# Model-based multilocus, genomic, and phenotypic integration reveals species assignment inconsistencies and sampling blind spots across Asian ecozones in Dicroglossidae

**DOI:** 10.1371/journal.pone.0360243

**Published:** 2026-10-23

**Authors:** Siti N. Othman, Yuran Dong, Boyang Yu, Tae Eun Um, Dallin B. Kohler, Vishal Kumar Prasad, Deyatima Ghosh, Xiaoli Zhang, Jiechen Wang, Amaël Borzée

**Affiliations:** 1 Laboratory of Animal Behaviour and Conservation, College of Life Sciences, Nanjing Forestry University, Nanjing, China; 2 The Co-Innovation Center for Sustainable Forestry in Southern China, College of Biology and the Environment, Nanjing Forestry University, Nanjing, China; 3 Jiangsu Provincial Key Laboratory for Advanced Remote Sensing and Geographic Information Technology, Key Laboratory for Land Satellite Remote Sensing Applications of Ministry of Natural Resources, School of Geography and Ocean Science, Nanjing University, Nanjing, Jiangsu, China; 4 Laboratory of Animal Behaviour and Conservation, College of Ecology and the Environment, Nanjing Forestry University, Nanjing, China; 5 School of Environment and Sustainability, Indian Institute for Human Settlements, Bengaluru, Karnataka, India; 6 School of Natural Sciences and Engineering, National Institute of Advanced Studies, Bengaluru, Karnataka, India; 7 Jiangsu Center for Collaborative Innovation in Geographical Information Resource Development and Application, Nanjing, China; CONICET: Consejo Nacional de Investigaciones Cientificas y Tecnicas, ARGENTINA

## Abstract

Widespread amphibians often show inconsistent species assignments in public databases, complicating accurate range-wide biodiversity mapping and obscuring gaps in taxonomic resolution. We assessed three widespread cryptic Asian Dicroglossid genera (*Fejervarya*, *Hoplobatrachus*, *Quasipaa*) across Indomalayan and Palearctic ecozones, where diffuse boundaries, taxonomic uncertainty, and exotic introduction impact biodiversity patterns. Using a hierarchical, scenario-based species delimitation framework based on publicly available molecular datasets and targeted new data, we evaluated 18 species-delimitation scenarios across 14 nominal taxa, with GenBank-based assignment as a control model. All scenarios were based on shared multilocus Dicroglossidae datasets (up to 517 sequence entries), integrating GenBank data with targeted sampling across two ecozones. The best-supported scenario was further refined using genome-derived SNPs from heterogeneous data sources (GBS, RNA-seq, and WGS/WCS), together with a binary morphological trait. Across analyses, the best-supported Model A1 showed a lower MLE than GenBank-based assignments, indicating non-random taxonomic mismatches between updated local classifications and public database entries. The model delineated 17 dicroglossid clades, including a single *Hoplobatrachus* lineage in East Asia. Diverging over 19 Mya, *Fejervarya* showed pronounced cryptic structure, with two deeply divergent clades recovered within the *F. limnocharis, F. kawamurai*, and *F. multistriata* species complexes, while *Quasipaa* displayed deep divergences. Integration of ecological niche modelling and bioacoustic analyses for a selected sympatric pair at the ecozone boundary further showed differences in predicted habitat associations and selected acoustic traits between *F. kawamurai* [Clade II] and *F. multistriata* [Clade I], despite substantial range overlap. *Fejervarya multistriata* was associated with coastal lowlands, whereas *F. kawamurai* was more widely distributed inland. Our integrative framework highlights regions with high mismatch under dense sampling (e.g., the Yangtze-Huai transition and East Asian mainland), and regions with mismatch under limited sampling, particularly insular systems (e.g., Taiwan and Hainan), defining priorities for future genomic surveys.

## Introduction

Conservation and research efforts are predominantly directed toward range-restricted [1], and charismatic species [2,3], leaving widespread taxa comparatively understudied. This imbalance, driven by their perceived complexity [4,5] and low conservation priority [6,7], contributes to persistent taxonomic uncertainty, including incorrect species assignments and unresolved boundaries. These limitations propagate inaccuracies in species distributions, identities, phylogeography [8], behaviour, ecological traits, and population estimates [9]. Particularly in East Asia, the transition zone between the Palearctic and Indomalayan ecozones [10], harbours species with unclear boundaries due to the overlapping faunal distributions [11]. For example, the Huai River and Jiangsu Plains in the Yangtze Delta represent key transitional areas between the two ecozones, where the distributions of many amphibian species overlap [12], shaped in part by glacial dynamics during the ice ages [13]. Resolving taxonomic uncertainty in such transitional landscapes requires model systems that are both widely distributed across these ecozones and representative of taxonomic challenges posed by morphological conservatism and cryptic diversity.

Public sequence databases, particularly GenBank (https://www.ncbi.nlm.nih.gov/genbank/), remain essential for large-scale biodiversity research, providing broad geographic coverage and an initial layer for species identification. However, reliance on these data without integrative validation may obscure cryptic structure and lead to inconsistent taxonomic resolution. To address this, we focus on three Asian Dicroglossidae genera (*Quasipaa*, *Fejervarya* and *Hoplobatrachus*) distributed across the Southeastern Palearctic and Eastern Indomalaya [14,15]. Our focal Dicroglossidae clades comprised 15 species [16], of which 13 are recognised in GenBank annotations. This discrepancy reflects the unresolved status of populations from mainland China labelled as *H. rugulosus* but corresponding to the distinct lineage *H. chinensis* [17,18], and the recent description of *F. zhoushanensis* [19] The focal taxa included widespread generalists such as *F. limnocharis* and *H. rugulosus*, occupying diverse habitats from agricultural wetlands and rice paddies to urban ponds [20,21], as well as saline-tolerant species like *F. cancrivora* and *F. moodiei*, capable of surviving high salinities in both adults and larvae [22,23]. The group also included habitat specialists, such as *Q. spinosa*, restricted to rocky montane streams [24], and *F. sakishimensis*, endemic to Taiwan and the southern Ryukyu Islands [25]. Several focal taxa, including *H. “chinensis” rugulosus*, *Q. spinosa* and *F. cancrivora*, are heavily traded for food in parts of East and Southeast Asia despite wildlife consumption restrictions [1,26–28]. Their morphological conservatism and frequent sympatry contribute to cryptic diversity and unresolved taxonomy, making them ideal models for integrative species delimitation. Therefore, aligning species identities with geographic ranges is essential for refining range-wide biodiversity mapping in widespread groups such as Dicroglossidae [29–31].

While independent, genome-wide sampling has proven effective in resolving range-wide biodiversity patterns across Asian ecozones, e.g., *Duttaphrynus melanostictus* [32] initial mapping using public sequence data provides a critical foundation for identifying geographic gaps, lineage structure, and priority regions for future, targeted sampling across Asian ecozones. As marker-based phylogenetic analyses of Dicroglossidae are often based on regionally focused datasets [33–35] and cannot readily compare populations across Asian ecozones, we employed an integrative, scenario-based delimitation framework with model testing to resolve cryptic structure and test species boundaries, while assessing the consistency of GenBank-based assignments. Recognising that genetic data derived from multilocus datasets may be insufficient to resolve shallow divergences at ecozone boundaries, our framework incorporated ecological niche modelling and bioacoustic analyses for selected clades to strengthen species delimitation and address morphological conservatism in anurans [36,37].

We grouped all scenario-based delimitation models into four categories: (a) allopatry versus sympatry, (b) latitudinal climatic gradients, (c) anthropogenic introduction, and (d) homology versus homoplasy. Accordingly, we divided this study into two main objectives: (1) to evaluate alternative lineage-assignment and species delimitation hypotheses within three Dicroglossid genera across the Eastern Indomalaya and Southeastern Palearctic using integrative species delimitation approaches, and genome-informed data. This includes the evaluation of *Hoplobatrachus* populations at the northern edge of their range on the East Asian mainland, a region where trade-related introductions may have contributed to complex lineage patterns. (2) To further assess the lineage boundaries within the widespread and sympatric *Fejervarya* species complexes in East Asia (*F. kawamurai*, *F. multistriata*, and *F. limnocharis*) through comparative phylogeographic analysis, complemented by ecological niche modelling and bioacoustics evidence. We further focused on three key *Fejervarya* species complexes across their ranges, including the Yangtze Plain, an ecological transition zone between the Indomalaya and Palearctic, where the cryptic *F. kawamurai* and *F. multistriata* occur in sympatry.

## Methods

### Field specimens, bioacoustics and public sequences data sampling

To construct a geographically broad framework for species delimitation simulation, we assembled publicly available molecular datasets as the primary data source for a geographically broad, scenario-based species delimitation framework, complemented by targeted field sampling at a key ecozone boundary. Field sampling focused on 11 opportunistic roadkill samples of Dicroglossidae (*Fejervarya* sp. = 10*, Hoplobatrachus* sp. = 1; GenBank accession numbers in S1 Table of S1 File), from five sites in Jiangsu, China: site 1 (31.978°N, 118.942°E), site 2 (33.135°N, 119.497°E), site 3 (33.081°N, 119.569°E), site 4 (34.156°N, 118.546°E) and site 5 (33.127°N, 118.802°E). We selected the Jiangsu Plains as a focal sampling area as this area represents the transition between the Southeastern Palearctic and the Northeastern Indomalaya. All specimens used for tissue sampling were collected after being found dead as roadkills, therefore no live animals were euthanised for tissue collection, and anaesthesia, analgesia, and procedures to alleviate suffering were not applicable. We stored all samples collected in 100% ethanol at −20 °C within 24 hours of collection until DNA extraction.

Given the cryptic nature in morphology between the sympatric clades of *F. kawamurai* and *F. multistriata* distributed across the Eastern Yangtze River Basin within the Southeastern Palearctic, we sampled the advertisement calls of *Fejervarya* from Nanjing (32.075°N, 118.806°E). Although the population could not be confidently assigned to either clade of *F. kawamurai* or *F. multistriata* in the field based on morphology alone, our preliminary verification included: (i) DNA sequencing of four representative individuals from the sampling site using the control region universal marker (see phylogenetic tree in S1 Fig of S1 File), and (ii) cross-checking phylogenetic placement with our species delimitation results. DNA samples were obtained non-lethally by swabbing the frogs, after which individuals were released at the sampling site. Only recordings meeting these verification criteria (*N* = 6; coordinates in S2 Table of S1 File) were retained for analysis. We recorded calls using a linear PCM recorder (Tascam DR–40X; Santa Fe Springs, CA, USA), paired with a unidirectional handheld microphone (Sennheiser MKE 600; Germany). All tissues and acoustics sampling in this study were conducted under the guidelines of Institutional Animal Care and Use Committee (IACUC) of Nanjing Forestry University, China (Approval Number: 2022014).

To extend geographic and taxonomic coverage, we compiled publicly available sequence data from GenBank and the NCBI Sequence Read Archive (SRA). The multilocus dataset comprised eight loci (4,044 bp) and 517 taxon entries used for species tree inference. All public records were curated and cross-checked prior to downstream analyses, with targeted field samples included for validation at a key distributional boundary (S3 Table in S1 File).

### DNA extraction and PCR amplification

We extracted genomic DNA from the tissues using the modified protocol of Chellex-100 method [38]. In total, we amplified eight multi-locus fragments, consisting of two ribosomal DNA sequences: 12S rRNA and 16S rRNA; three mitochondrial fragments: Control Region (*CR*), Cytochrome b (Cyt*b*), cytochrome C oxidase subunit I (*COI*); and three partial nuclear fragments: tyrosinase (*TYR*), recombination activating 1 (*RAG1*) and rhodopsin (*Rho*). Details of the primers used for PCR amplification, including sequence, length, and source, are provided in S4 Table in S1 File.

In a 20 µL reaction per tube, we performed standard Polymerase Chain Reactions (PCR) using the extracted DNAs as the templates (35–50 ng/μL). Each PCR reaction contained 10 μl I − 5TM High-Fidelity Master Mix (MCLAB, California, USA), 0.125 μM of each primer, and 1 μl of DNA template. Lastly, we added ddH_2_O to make up the final volume. We amplified the PCR reactions using Applied Biosystems MiniAmp Thermal Cycler (California, USA), following a standard PCR protocol using the following thermal profiles: (1) 5 min at 90 °C for pre-denaturation; (2) 1 min at 95 °C for denaturation; (3) 30 sec at specific temperatures for annealing, (4) 1 min at 72 °C for elongation, with the stages 2–4 repeated for 35 cycles; and (5) a final elongation at 72 °C for 5 min. We ran all PCR amplicons on 1.5% agarose gel, and visualized the final products with BluEye^TM^ transilluminator (Suzhou, China). We sent all the amplicons for purification, and sequencing to obtain both forward and reverse reads, using an ABI platform (Tsingke Biotechnology Co., Ltd, Beijing, PR China).

### Integrative species delimitation scenarios, model testing and mapping

We evaluated 18 species delimitation models, comprising 16 alternative hypotheses, one GenBank-based control (C1), and the final selected model (A1), derived from biogeographic, climatic, anthropogenic, and morphological evidence to test competing hypotheses of lineage boundaries. Datasets were structured hierarchically to allow iterative refinement as additional taxa and hypotheses were incorporated, and organised into preliminary (Pre) and revised (Rev) model sets, with the control (C1) and a final selected model (A1) representing the best-supported hypothesis (see details in S5 and S6 Tables of S1 File). Species trees for each scenario were reconstructed using an eight-locus multilocus dataset (4,044 bp), including up to 517 taxon entries, under a multispecies coalescent framework implemented in StarBEAST2, applying a relaxed lognormal clock model [39]. For coherence, we evaluated all models under identical analytical settings. We ran species trees using independent MCMC runs sampling, with convergence assessed based on effective sample sizes (ESS > 200), and Maximum Clade Credibility trees generated after discarding burn-in (full calibration priors, parameter settings and convergence diagnostics provided in the Supplementary Methods).

We evaluated the relative support of competing delimitation models using a Bayes factor delimitation (BFD) framework, based on marginal likelihood estimation obtained through nested sampling, independently using the NS model test package [40] embedded in BEAST v.2.6.7 [41]. Model fit was assessed using marginal likelihood estimates (MLE) and Bayes factors, with higher MLE values indicating better support. Comparative model fit between the GenBank-based control (C1) and the best-supported model (A1), based on both multilocus and genome-wide datasets, is reported in S6 Table in S1 File. Full details of model implementation and parameter settings are provided in the Supplementary Methods**.**

Finally, we compared current GenBank taxonomic labels retained in Model C1 against the best-supported species assignments recovered under Model A1 using a curated dataset of 392 verified entries (S3 Table in S1 File). We checked each record against voucher information, GenBank metadata, and original sources where necessary, then classified as either a match or mismatch depending on whether the GenBank label agreed with the Model A1 assignment. We tested whether mismatches deviated from random expectation using a chi-square test, with significance assessed at p < 0.05. We summarised the resulting counts by species pair and region (https://github.com/OthmanSN/Sampling_hotspots_dowbstream_analyses), and visualised them using an alluvial plot generated in R v.4.2.3 [42].

### Enrichment of morphology and genome-wide data into best-supported model

We refined the best-supported multilocus Model A1 by integrating genome-wide SNP data derived from heterogeneous sources (S7 Table in S1 File), and a matrix of binary morphological trait representing diagnostic characters across major species complexes, to provide an additional line of evidence for species delimitation (S8 Table in S1 File). Given the public genomic datasets comprised GBS, RNA-seq and WGS/ WCS data generated using different sequencing strategies, restriction-enzyme and library-preparation information was not consistently available across datasets. Consequently, a uniform de novo assembly could not be applied. We therefore used a common reference-based framework to standardise sequence recovery across the heterogenous datasets. Because the genomic analysis was conducted within a single Asian Dicroglossidae phylogenetic framework, we aligned the reads to the *Nanorana parkeri* genome (GCF_000935625.1), which was used as a common family-level reference across the focal genera, and provides a chromosome-level assembly with high scaffold continuity. We aligned reads with reference genome using BWA-MEM v.0.7.17 [43]. For GBS datasets, SNP calling was performed using the Stacks v.2.66 pipeline [44]. For Rna-seq and WGS/WCS datasets, we processed alignments using the GATK v.4.6 germline variant discovery pipeline [45]. Resulted VCF files were subsequently converted into NEXUS format for downstream species tree inference in BEAST pipeline (see loci information in S9 Table of S1 File). This reference-based approach also generated genomic-locus alignments compatible with the StarBEAST2 framework, which accommodates sequence alignments derived from independent genomic loci such as RAD-seq and exons [41]. Further details of raw reads processing and downstream analysis are provided in the Supplementary Methods.

Following genomic processing and filtering, we assessed data completeness directly from the genomic alignments retained for Model A1. For each sample, we recorded the number of retained loci and quantified the number and proportion of missing or ambiguous characters (N and?) relative to the total alignment length. To evaluate variation among the heterogenous public datasets, we summarised these metrics at the sample and locus-recovery levels, and recorded the original sequencing data source (GBS, RNA-seq, or WGS/WCS). Although locus recovery differed among datasets (S10 Table in S1 File), with 70 loci retained for RNA-seq samples, 64 for GBS samples, and 13 for WGS/WCS samples, median sequence-level missingness was 1.54% (S2 Fig in S1 File). All 33 genomic samples were retained because each sample contained data across the full set of loci retained for its respective dataset.

Finally, we integrated the genomic and morphological data within the same StarBEAST2 analytical framework used for the multilocus models to refine topology and divergence estimates under the best-supported Model A1.

### Calibration of species trees and final model inference

Divergence times were estimated for all species trees under a multispecies coalescent framework implemented in StarBEAST2, applying a relaxed lognormal clock model [39]. In the absence of fossil evidence for the focal genera, we applied a secondary calibration to constrain the root age of Dicroglossidae in East Asia, based on published estimates [46], placing its basal divergence between the Late Palaeocene and Early Eocene (c. 59 Mya; 95% HPD: 55–66 Mya). This calibration was implemented as a normal prior to anchor divergence time estimation and is consistent with broader hypotheses of post-Cretaceous–Paleogene anuran diversification [47], and the temporal framework recently evaluated for Northeast Asian anurans [48]**.** For the genome-wide refined Model A1, additional secondary calibration points derived from previous mitochondrial-based estimates [46,49] were applied as normal priors, representing key divergence events within *Hoplobatrachus*, *Fejervarya*, and *Quasipaa* lineages. These calibrations were used to refine divergence time estimates under the final model (see supporting information for the calibration enforcement details in S1 File).

We conducted final analyses under a fossilised birth-death (FBD) speciation prior [50] in StarBEAST RLC v.2.6.3 [39,41]. For robustness, we conducted an initial MCMC run of 500 million generations, sampling every 50,000 generations. To improve effective sample sizes across parameters, we performed two additional independent runs of 250 million generations each (resampling every 2,500 generations) and one independent run of 50 million generations that included the morphological matrix (resampling every 500 generations), yielding a total accumulated sampling effort of 1.05 billion generations. The reduced logging interval in the latter runs increased the number of retained samples without extending runtime. All runs were conducted on separate workstations, and the resulting trees were combined in LogCombiner v.2.6.3 [41], with resampling every 2,500 generations and discarding the first 10% of samples as burn-in. Convergence and effective sample sizes (ESS > 200) of key parameters were jointly assessed in Tracer v.1.7 [51].

### Adjusted Ecological Niche Modelling with species delimitation

To evaluate whether taxonomic discordance identified in geographic mapping corresponded to ecological differentiation in a key transition zone, we combined best-supported species delimitation model with a maximum entropy algorithm (MaxEnt) to examine ecological differentiation between two clades in Yangtze River Basin (*F. kawamurai* and *F. multistriata*), selected due to their sympatry and crypticity at the Indomalayan-Palearctic ecozone boundary. Considering the uncertainty and high crypticity in distribution, genetics, and morphology between the clades of *F. multistriata* and *F. kawamurai* in mainland East Asia, we relied on presence-only data as our sampling assumption. We collected occurrence data for *F. kawamurai* and *F. multistriata* through both field surveys and the Global Biodiversity Information Facility (GBIF.org). We minimised potential misidentifications in open-source records, particularly for cryptic taxa, by removing entries lacking essential metadata, excluding points outside the revised range, and verifying photographic vouchers where available. For *F. multistriata*, we used a validated occurrence dataset integrating our field survey data with vetted GBIF records and remote-sensing variables [52], to generate high-resolution habitat suitability models. This prior validation reinforces the ecological plausibility and reliability of the occurrence data used in this study.

We carried out the field surveys within each cell of a 10 km grid square overlayed across the western Jiangsu Plains, starting from the Yangtze River continuing 100 km towards the north. In addition, we conducted opportunistic surveys in suitable habitat at the known northern limit of *Fejervarya* in the Palearctic [53]. We determined the occurrences of the species during the peak of breeding season, through aural surveys conducted at each site for 10 minutes [54]. From GBIF, we obtained additional occurrence data, comprising 2,076 records for *F. kawamurai* (https://doi.org/10.15468/dl.n889nn, accessed on 11 October 2023) and 1,427 records for *F. multistriata* (https://doi.org/10.15468/dl.tdd63a, accessed on 11 October 2023). We filtered the records to remove erroneous occurrences based on the best-supported species delimitation model. To reduce spatial sampling bias, we applied a 10 km thinning procedure [55], resulting in 286 occurrences for *F. kawamurai* and 597 for *F. multistriata*.

We obtained 19 bioclimatic variables from WorldClim v.2 (www.worldclim.org) [56], at 30” resolution and an elevation layer from the ASTER GDEM v.3 dataset [57]. All layers were projected to WGS 1984 projection system in ArcGIS v.10.8 [58]. To prevent overfitting caused by multicollinearity among environmental variables [59], we excluded specific variables to ensure that their correlations did not exceed 0.7 (|r| < 0.7), using ‘cor’ function in R package ‘stats’ v.4.4.2 [60], with the final variable set provided in the Supplementary Information (S11 and S12 Tables and S3 Fig of S1 File).

We implemented Ecological niche models in MaxEnt v.3.4.1 using default settings, with cross-validation, 10 replicates, 10,000 background points, and a 75/25 training–testing split [61]. Model performance was evaluated using AUC and TSS [62]. We classified habitat suitability using two thresholds: minimum training presence (minPred), as a permissive threshold, and maximum training sensitivity plus specificity (MTSS), as a more conservative threshold that reduces background bias [63–65]. Full model settings and evaluation procedures are provided in the S1 File.

### Bioacoustics analysis and statistical evaluation of advertisement calls

To test the delineation suggested by the optimal Model A1 for *F. kawamurai* and *F. multistriata* in the Yangtze River Basin, we conducted a bioacoustic analysis comparing the advertisement calls of the two clades. For the recorded advertisement calls, we employed a sampling rate of 44.1 kHz with a 16‑bit resolution [66], and analysed the recorded advertisement calls using Raven Pro v.1.5 [67]. We standardised the extraction of call variables for statistical analyses following the acoustic properties described in the literature of *F. multistriata* [68,69]. For each individual, we measured the snout-vent length (SVL) and recorded ambient temperature during sampling. For sonograms and oscillograms, we extracted mean dominant frequency (Hz), maximum and minimum frequency (Hz), call rates (call/min), call duration (s), call interval (s), and pulse rate (pulse/s). We then compared all sampled advertisement calls representing putative *F. kawamurai* Clade II with those of a population representing putative *F. multistriata* Clade I, distributed in Shanghai (*N* = 11; S13 Table in S1 File), following previous observations [68].

We first evaluated the influence of SVL and temperature on call parameters using regression models. Our models showed that temperature influenced call duration and call interval, and SVL influenced dominant frequency. Subsequently, we tested the normality of distribution for the data using the Shapiro-Wilk test, which confirmed that all data met normality assumptions (p > 0.05), so transformation was unnecessary. To account for variance heterogeneity, we also applied Welch’s analysis of variance (Welch’s ANOVA) to the call duration, which confirmed species differences. With species as the factor, we analysed overall multivariate differences in call structure between clades using a multivariate analysis of variance (MANOVA). Although the MANOVA was not fully significant, it suggested that most of the variation came from call duration and call interval. Based on these results, we further conducted follow-up analyses of covariance (ANCOVAs), adjusting call duration and call interval for temperature and dominant frequency for SVL, while leaving other traits unadjusted. All statistical analyses were conducted in R v.4.2.3 [42], performed using the packages “car” [70], “emmenas” [71], “ggplot2” [72] and “patchwork” [73].

## Results

Across scenario-based delimitation analyses, we identified consistent lineage structure across Asian ecozones, with Model A1 providing the best-supported framework for distributional mapping (Fig 1). Compared to the GenBank-based assignment (Model C1), Model A1 revealed a structured pattern of taxonomic mismatch that deviated from random expectation, with lineage partitions broadly aligning with geographic boundaries, particularly within *Fejervarya* (Fig 2).

**Fig 1:**
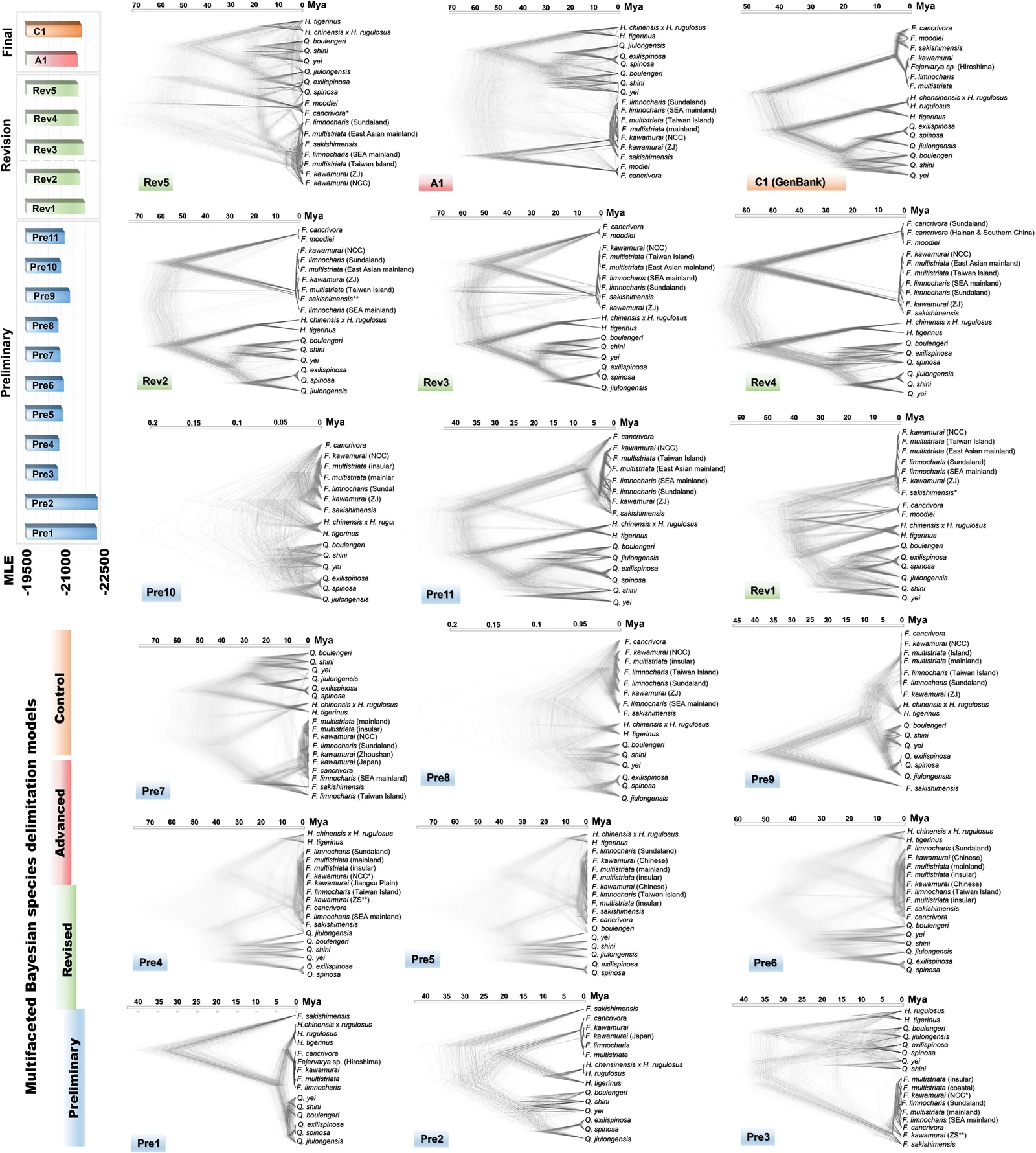
Hierarchical evaluation of 18 competing species-delimitation models, progressing from preliminary and revised models to the final best-supported Model A1. The topology for each model corresponds to the scenarios described in S5–S6 Tables in S1 File. All integrative Bayesian species delimitation models were tested using Bayes Factor Delimitation and nested sampling, including: (a) preliminary models (Pre–Pre11), designated to test the major clades of Asian Dicroglossidae based on geographic, climatic, and life history factors; (b) revised models (Rev1–Rev5), designated to test trade-associated clades (e.g., *F. cancrivora*) and isolated clades (e.g., *F. sakishimensis* from the Ryukyu Islands) into the best-supported preliminary model. Final Model A1 emerged as the best-supported delimitation model across GenBank-assigned Model C1 and the other alternative, integrating scenarios across clades from preliminary and revised stages, consistent with species boundaries and recent updates from the literature. The bar charts on the left show the marginal likelihood estimation (MLE) values per model, values for each model, with colours indicating model stages (see legend).

**Fig 2:**
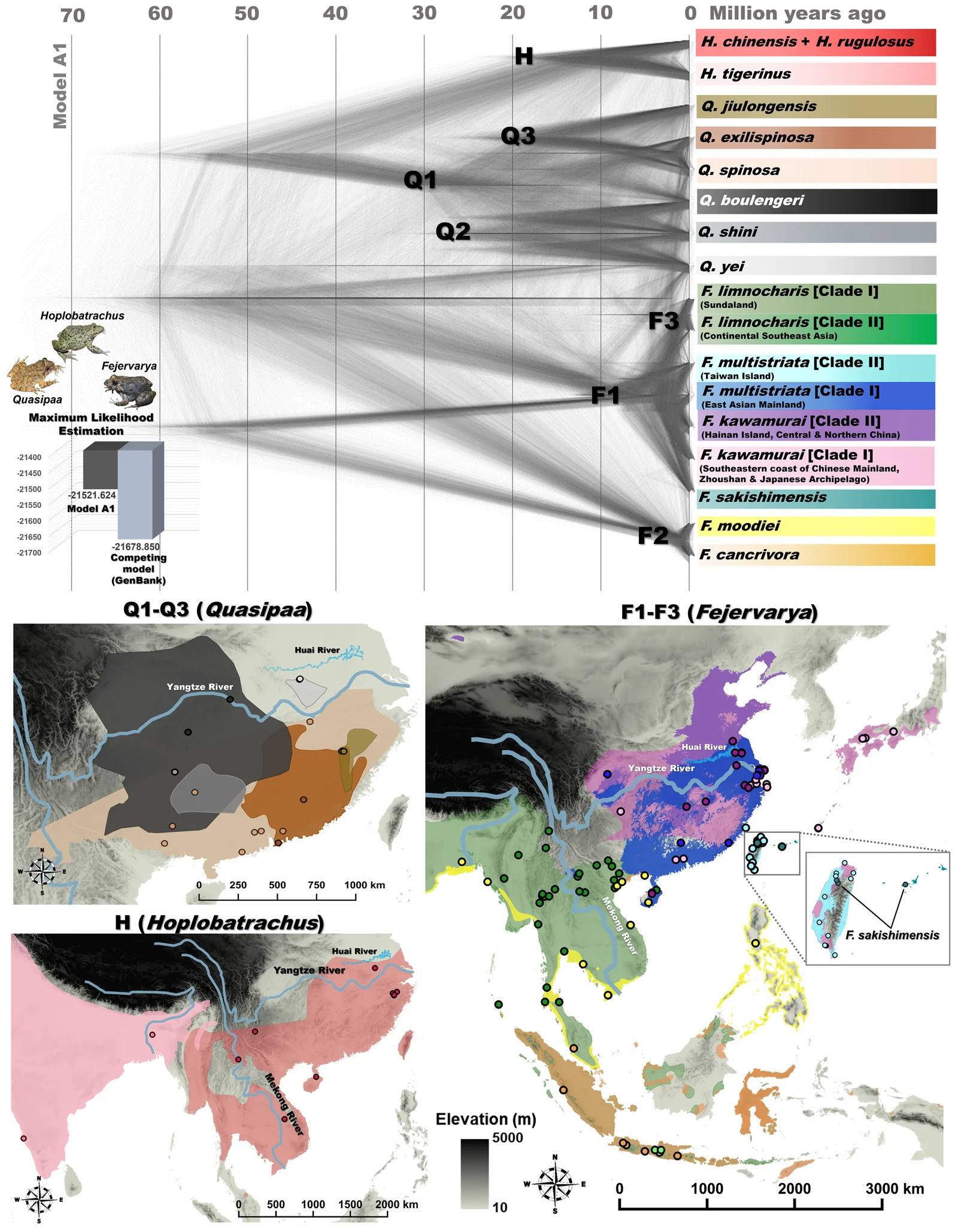
The best-supported species delimitation for Dicroglossids in the Southeastern Palearctic and Eastern Indomalaya inferred from common multilocus markers. The topology of Dicroglossids is derived from species trees generated using the best-supported species delimitation Model A1. Each genus is represented by major clade Q (*Quasipaa*), major clade H (*Hoplobatrachus*) and major clade F (*Fejervarya*). Species ranges are primarily rely on shapefiles provided by the IUCN [89], except for all clades of *F. kawamurai* and *F. multistriata* occurring sympatrically across the northern Indomalaya and southeastern Palearctic in mainland East Asia, presented using thresholded ENM ranges extracted from this study. All species range colours correspond to the colours of each clade within the species tree. Clades I represents the type locality lineage, and Clades II represents the diverged lineage. The elevation base map is generated by the authors from Shuttle Radar Topography Mission (SRTM) elevation data [92], obtained through DIVA-GIS. Species-range shapefiles are obtained from the IUCN [74], except for the *F. kawamurai* and *F. multistriata* ranges generated from the ENM analyses in this study. Major river layers are derived from Natural Earth 1:110 m physical vector data [93], whereas the Huai River is derived from the China 1:250,000 National Fundamental Geographic Database [94]. Frog photographs are taken by S.N.O and A.B.

Genome-wide refinement and divergence time estimates further clarified evolutionary relationships (Fig 3). Focusing on a sympatric lineage pair at the Yangtze-Huai ecozone boundary, ecological niche and acoustic analyses provided complementary evidence for differentiation between lineages despite geographic overlap (Fig 4).

**Fig 3:**
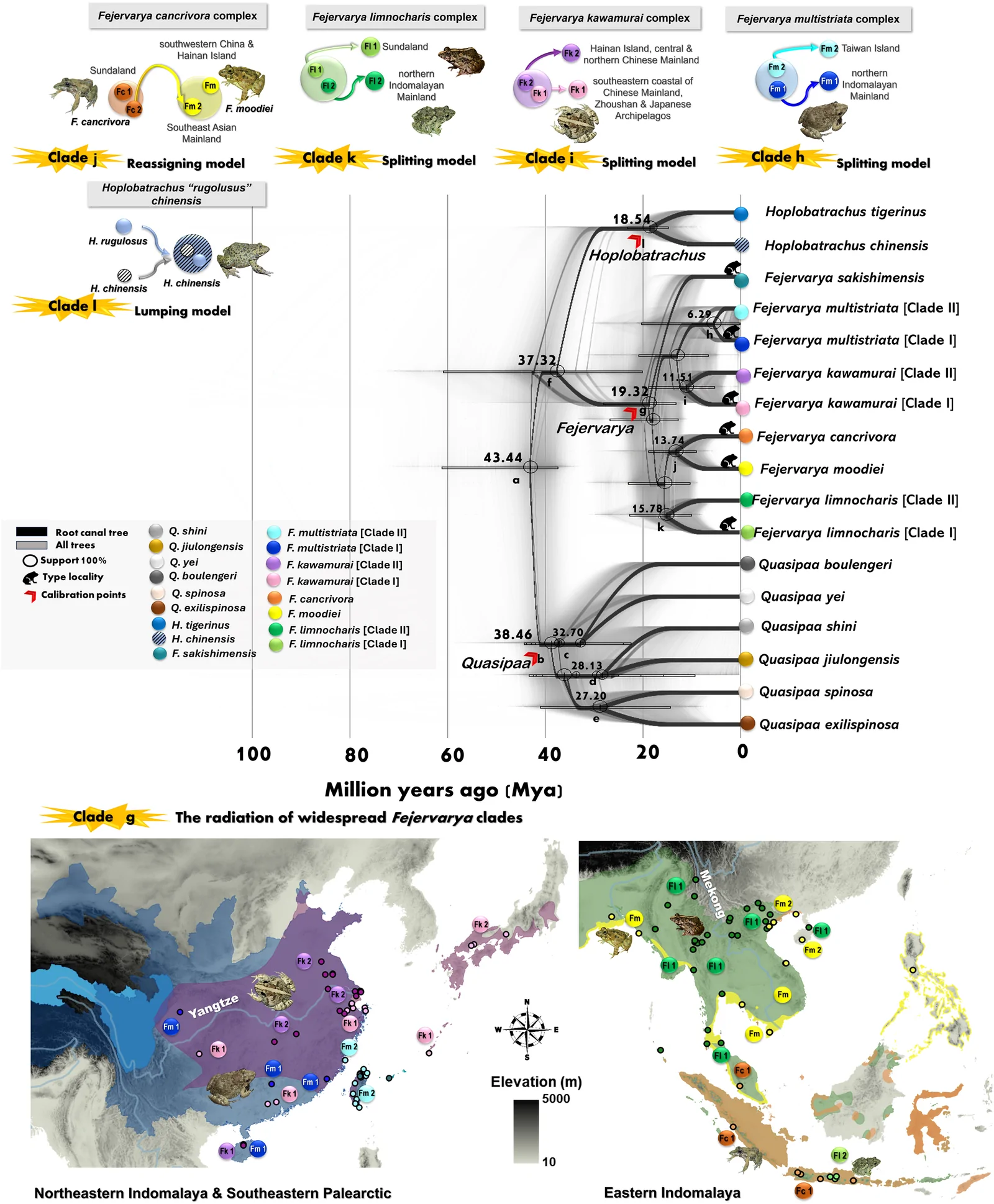
Refined species tree and divergence dating under the best-supported Model A1 using genome-wide and multilocus data. (A) Species trees reconstructed using genome-wide and multilocus data derived from the best-supported Model A1. The black branches represent the maximum clade credibility tree (root canal), indicating the consensus topology. The grey branches at background represent the posterior distribution of consensus trees, highlighting uncertainty and variation across the species trees. The support values for the tree nodes, ranging from 26% to 100%, are indicated by the size of the circles. Divergence estimates and calibration points are shown at each node, with corresponding divergence times and 95% HPD intervals for clades and nodes (a–n) provided in S15 Table in S1 File. (B) Taxonomic recommendations based on splitting, reassigning, and lumping mechanisms in species delimitation focused on cryptic clades within Dicroglossidae, particularly in the *Hoplobatrachus* and *Fejervarya* genera, are discussed. Species boundaries in the maps highlighted the continuous divergent of *Fejervarya* in the Eastern Indomalaya and Southeastern Palearctic regions, based on best-supported Model A1**.** The elevation base map is generated by the authors from Shuttle Radar Topography Mission (SRTM) elevation data [92], obtained through DIVA-GIS. Species-range shapefiles are obtained from the IUCN [74], except for the *F. kawamurai* and *F. multistriata* ranges generated from the ENM analyses in this study. River layers are derived from Natural Earth 1:110 m physical vector data [93]. Frog photographs are taken by S.N.O and A.B.

**Fig 4:**
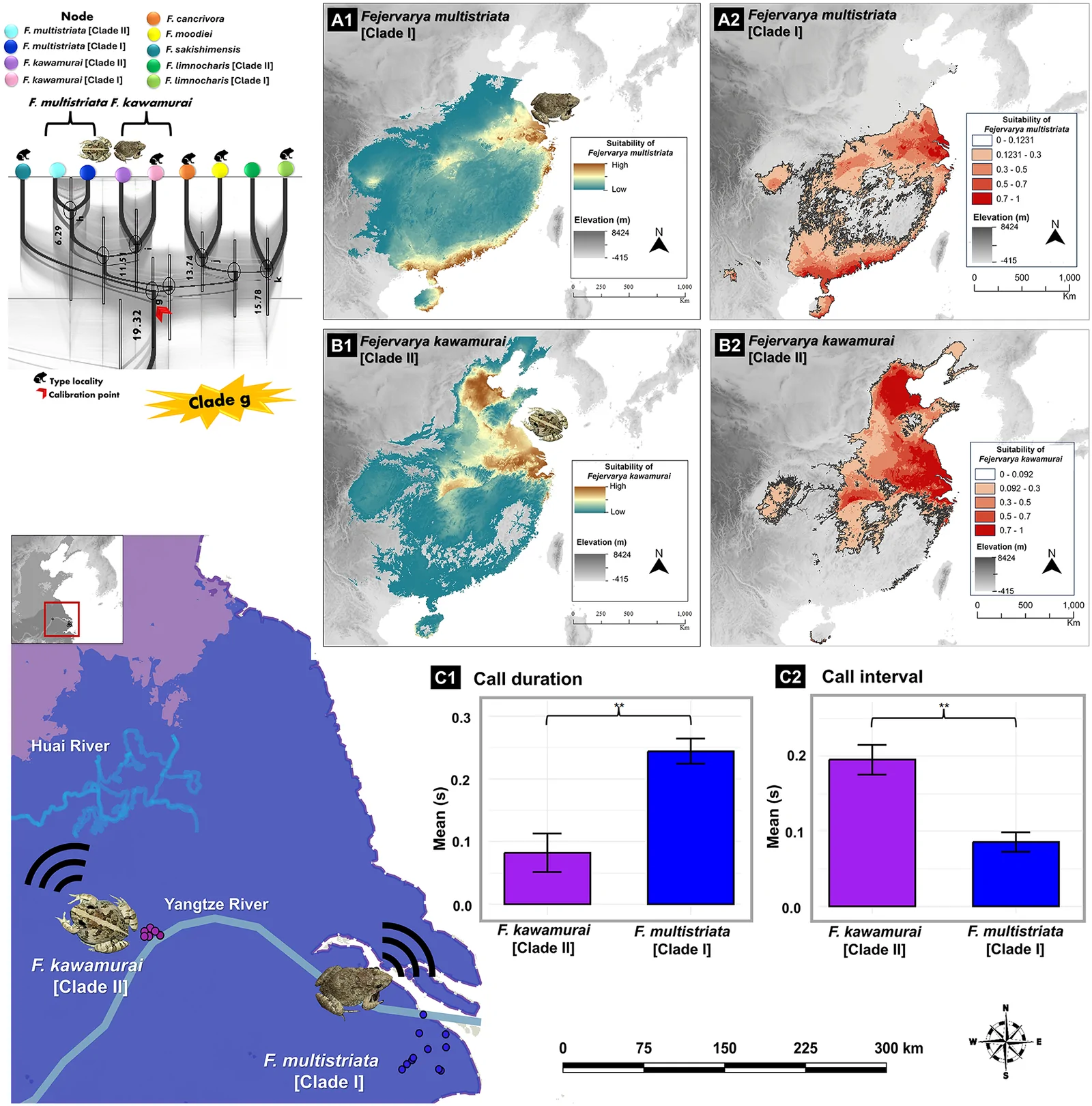
Ecological and phenotypic variation across ecozone boundaries relevant to species delimitation in selected cryptic and sympatric *Fejervarya* clades in the Yangtze Basin. We evaluated species boundaries between Clade I of *F. multistriata* and Clade II of *F. kawamurai*, with substantial range overlap across Central and Eastern China, within the transitional zone between the Palearctic and Indomalaya. Presence-only habitat suitability models were developed using two different thresholds, minPred and MTSS. The coloured areas represent habitat suitability on a continuous scale for the models, with thresholds set at a minPred value of 0.00819 for *F. multistriata* (A1) and 0.01319 for *F. kawamurai* (B1), and at an MTSS value of 0.1231 for *F. multistriata* (A2) and 0.092 for *F. kawamurai* (B2), with the latter thresholds marked by black lines. ANCOVA revealed significant interspecific differences in two advertisement call traits, call duration (C1: F(1, 12.3) = 7.26, p = 0.019) and call interval (C2: F(1, 15) = 13.12, p = 0.0012), after adjusting for temperature (S15 and S16 Tables in S1 File). Base map data were obtained from Shuttle Radar Topography Mission (SRTM) elevation data obtained through DIVA-GIS. Species-range polygons of *F. kawamurai* and *F. multistriata* ranges generated from the ENM analyses. The Yangtze River layer is derived from Natural Earth 1:110m physical vector data [92], whereas the Huai River is derived from the China 1:250,000 National Fundamental Geographic Database [94]. Frog photographs are taken by S.N.O and A.B.

Regional comparisons of assignment patterns further highlighted geographic structure, with mismatches concentrated in ecotonal mainland regions, notably the Yangtze-Huai transition where co-occurring lineages were detected. Insular systems such as Taiwan and Hainan also showed overlapping species occurrences despite lower sampling, providing distinct contexts in which assignment mismatches were evident (Fig 5).

**Fig 5:**
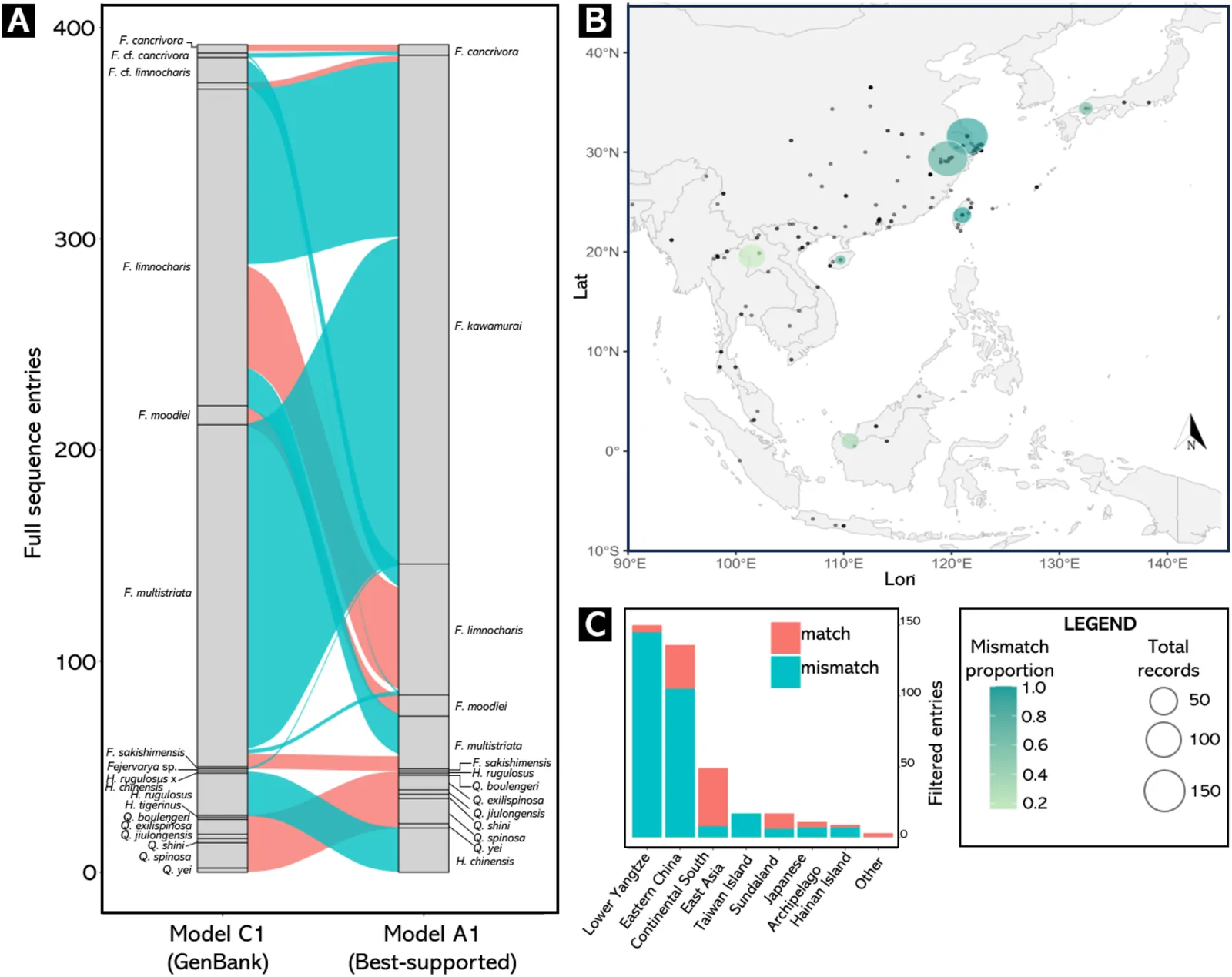
Geographic patterns of taxonomic reassignment and mismatch across Dicroglossidae datasets. (A) Alluvial plot showing reassignment of GenBank-based species assignments (Model C1) to the best-supported species delimitation model (Model A1), based on 393 verified sequence entries (S18 Table in S1 File). Flows represent the redistribution of sequence entries among taxa under the revised framework. (B) Geographic distribution of mismatch records between GenBank assignments and Model A1 based on the filtered entries. Black points indicate all georeferenced sequence entries, while circles represent regional summaries of mismatch-only records. Circle size corresponds to the total number of entries per region, and colour indicates mismatch proportion (see legend). Circle positions are based on the median geographic coordinates (centroids) of sampling localities within each region. (C) Bar chat represents regional summary of filtered entries, showing counts of matching and mismatching assignments used to define sampling priorities. Regions with high mismatch proportions and low sampling density highlight potential priorities for future genomic sampling. Map is generated in R v.4.2.3 [42] using Natural Earth base map data [95].

### Lineage structure across ecozones

Model A1 emerged as the best-supported species delimitation model, showing improved fit relative to the GenBank-based assignment (Model C1) based on marginal likelihood estimates (MLE: −21,521.624 vs −21,678.850; S6 Table in S1 File and Fig 2) and Bayes factors (BF: 314.452; S6 Table in S1 File). This result indicated that incorporating biological and ecological context improves species delimitation relative to reliance on GenBank labels alone. Additionally, Model A1 recovered consistent lineage structure across the three focal Dicroglossidae genera, broadly aligning with geographic structure and species boundaries recognised in regional taxonomic studies and distributional data [74]. In *Fejervarya*, multiple widespread species complexes (e.g., *F. limnocharis*, *F. kawamurai*, and *F. multistriata*) were each resolved into two geographically structured lineages (Clade F; Fig 2), broadly corresponding to mainland and insular divisions across Southeast and East Asia. Similarly, *F. cancrivora* and *F. moodiei* formed distinct lineages associated with Sundaic and mainland Southeast Asia distributions, respectively, while *Hoplobatrachus* was recovered as a single lineage of *H. chinensis* across mainland East Asia (Clade H; Fig 2). In contrast, *Quasipaa* showed deep and well-resolved divergence among species, with no evidence of cryptic subdivision (Clade Q; Fig 2).

### Genome-wide refinement and divergence-time estimation

Genome-wide integration provided further support for Model A1 relative to GenBank-based Model C1 (S14 Table in S1 File), consistent with multilocus results. Genome-refined Model A1 also achieved a higher marginal likelihood estimate (MLE: −26,636.676), than the control model C1 (MLE: −26,753.096), and Jeffrey’s Bayes Factor (e^116.42^; e > 100) provided decisive support for our model, reinforcing the lineage structure recovered under Model A1. In addition, incorporation of genome-wide SNP data improved phylogenetic resolution and resolved conflicting relationships among genera. In particular, *Hoplobatrachus* was recovered as more closely related to *Fejervarya*, contrasting with the multilocus tree where it grouped with *Quasipaa* (Fig 3).

The species tree dated the basal divergence of the three Dicroglossidae genera on the Asian mainland to c. 43.4 Mya, since Eocene (Clade a; Fig 3; see 95% High Posterior Density (HPD) interval for estimated ages in S15 Table of S1 File). The *Quasipaa* lineage emerged in the Middle Eocene, c. 38.46 Mya (Clade b; Fig 3), and subsequently diverged into a clade containing *Q. boulengeri* and *Q. yei* in central to southern China, c. 32.70 Mya (Clade c, Fig 3). This was followed by divergence of *Q. jiulongensis* and *Q. shini*, c. 28.13 Mya (Clade d; Fig 3), and sister clades *Q. spinosa* and *Q. exilispinosa* in Southern to Southeastern China, c. 27.20 Mya (Clade e; Fig 3).

The *Hoplobatrachus* stem lineage arose in the Early Miocene, c. 18.54 Mya (Clade l; Fig 3), giving rise to *H. tigerinus* (South and mainland Southeast Asia), and *H. chinensis*. The latter initially diverged into two clades in continental East Asia (type locality: presumably the vicinity of Canton, China) and Southeast Asia (referred to as *H. rugolosus*, type locality: Kap Shui Mun, Lantau I., Hongkong, China), which later merged into a single lineage, likely due to trade-mediated translocation (Fig 2).

In contrast, *Fejervarya* showed a continuous pattern of lineage diversification, with all three major species complexes diverging from their respective type locality lineages during the Early to Middle Miocene (c. 19.32–6.29 Mya; Fig 3). The *Fejervarya* assemblage emerged in the Early Miocene (c. 19.32 Mya; Fig 3), followed by diversification across the biogeographic separation between Indomalayan and Southern Palearctic lineages. The *F. cancrivora* and *F. limnocharis* species complexes originated during the Middle Miocene, c. 15.78 Mya to c. 13.74 Mya (nodes j and k; Fig 3), respectively. The *F. cancrivora* complex originated in sothern Sundaland/ Sundaic, encompassing southern Thailand, the Malay Peninsula, Sumatra, Borneo, Java (type locality), and surrounding smaller islands. This was followed by the formation of *F. moodiei* in continental Southeast Asia, covering Thailand, northern Vietnam, the Philippines (type locality), and Hainan Island, c. 0.85 Mya (based on multilocus time tree; Fig 2). *Fejervarya sakishimensis*, distributed in the Ryukyu Islands (type locality) and northern Taiwan, represented a distinct clade that originated c. 19.31 Mya (Fig 3), and is closely related to *F. moodiei* and *F. cancrivora*. The *F. limnocharis* complex diverged into two lineages c. 15.78 Mya (node k; Fig 3), comprising Clade I restricted to Sundaland (including Indonesia, Peninsular Malaysia, and Borneo), and Clade II distributed across continental Southeast Asia (Fig 2).

Diversification within the Southeastern Palearctic involved the emergence of *F. multistriata* and *F. kawamurai* at the transition between Oligocene and Miocene, c. 13.35 Mya (Fig 3). The *F. kawamurai* complex originated c. 11.51 Mya (Clade i; Fig 3), with Clade I distributed across southeastern coastal China, including Guangzhou, Zhoushan, the Japanese Archipelago (type locality), and Taiwan Island. Clade II, in contrast, was found across central and eastern mainland China, extending as far north as Beijing, with divergence estimated at c. 2.14 Mya (Fig 2). The *F. multistriata* complex originated c. 6.29 Mya (Clade h; Fig 3), with a subsequent diversification c. 2.00–3.00 Mya (Fig 2), temporally overlapping with Pleistocene climatic fluctuations in the Palearctic, although a direct association was not tested here. Clade I widely distributed across central and eastern coastal China, while Clade II restricted to Taiwan Island, c. 1.95 Mya (3.09–0.71 Mya; Fig 2).

### Ecological Niche Modelling for the two most cryptic *Fejervarya*

Ecological niche modelling revealed differences in predicted habitat associations within a broadly overlapping area of suitable habitat between the two cryptic clades *F. kawamurai* and *F. multistriata* at the boundary between the Northeastern Indomalayan and Southern Palearctic ecozones (Fig 4). Both models proved reliable in predicting performances (based on AUC > 0.8 and TSS > 0.6 [75]). For *F. kawamurai*, the models using both minPred and MTSS thresholds were characterised by an AUC of 0.969 ± 0.019 (Fig 4A1–4A2), and 0.968 ± 0.017 (Fig 4B2). For the *F. multistriata* models, the average test AUCs for the replicate runs were 0.967 ± 0.080 (Fig 4B1) and 0.966 ± 0.011 (Fig 4A2). We obtained distinct minPred thresholds delineating the minimum predicted probability of suitability for one set of models of both species. *F. multistriata* had a minPred value of 0.00819 (Fig 4) and *F. kawamurai* had a minPred value of 0.01319; Fig 4A1–4B1). The TSS values were considered good for the models of both *Fejervarya* species, with a TSS value of 0.838 for *F. multistriata* and a value of 0.867 for *F. kawamurai* (Fig 4A2–4B2).

We identified differences in the combination of key variables that contributed to the suitability habitat of both species. In addition, for each species, these key variable combinations were quite consistent across the two different thresholds. For *F. multistriata*, the precipitation of the driest month (Bio 14) and mean diurnal range (Bio 2) collectively explained >78% of the suitable habitat for *F. multistriata* (S11 and S12 Tables in S1 File). Meanwhile, elevation had a significant impact on the modelling of *F. kawamurai*, with the combination of elevation and Bio 14 accounting for 80.3% of the ecological requirements for one model, and the combination of elevation and annual mean temperature (Bio 1) accounting for 81.6% of the ecological requirements for other.

Binary projections from the minPred threshold showed large, but structured overlap. The total area covered for suitable habitat of *F. kawamurai* was 1,870,774.17 km^2^ and for *F. multistriata* was 2,091,013.23 km^2^, with an overlap of 1,570,405.79 km^2^ (Fig 4). Within this broadly overlapping area, the relative distribution of predicted habitat suitability nevertheless differed spatially. Potential suitable habitat of *F. multistriata* was highest around the Chinese coastal areas of present-day Jiangsu, Shanghai, Zhejiang, Fujian, Guangdong, Guangxi, and Hainan. The northernmost range of *F. multistriata* within the Southeastern Palearctic extended until Jiangsu and Anhui plains (Fig 4A1–4B1). However, using the stricter threshold based on MTSS, the results for both clades indicated that: (a) the highly suitable habitats for *F. multistriata* [Clade I] were concentrated across the central coastal region of China, encompassing Jiangsu, Zhejiang, and Fujian, (b) although both clades are observed in the Jiangsu Plains, *F. kawamurai* was predominantly distributed along both the northern and southern areas of the Yangtze Basin, while *F. multistriata* had a more extensive presence in the southern portion, and (c) the northern boundary of the distribution of *F. multistriata* in the Palearctic realm was restricted to the vicinity of the northern Huai River (Fig 4A2–4B2). Both thresholds clearly indicated that suitable habitats for *F. kawamurai* were found farther north than for *F. multistriata*, extending into the northern portion of the Yangtze Basin, and reaching into Shandong, Hebei and Beijing (Fig 4A1–4B1).

In the Indomalaya realm, the suitable habitat for *F. kawamurai* was primarily restricted to the present-day Central Chinese mainland, ranging west and encompassing areas of present-day Sichuan and Chongqing (Fig 4A2–4B2). Both *F. kawamurai* and *F. multistriata* clades exhibited habitat suitability on Hainan Island, although *F. kawamurai* demonstrated more limited suitability than *F. multistriata* (Fig 4A2–4B2).

### Acoustic variation between the two most cryptic *Fejervarya*

While multivariate analyses suggested no broad-scale acoustic separation, call duration and call interval consistently differentiated the two clades, indicating early-stage signal divergence despite overlap in other acoustic properties. Although sample sizes were limited (S13 Table in S1 File), the divergence in these temporal traits was consistent across analyses and remained significant after accounting for temperature effects.

MANOVA indicated that overall acoustic profiles of *F. kawamurai* [Clade II] from the Jiangsu Plain (*N* = 6) and *F. multistriata* [Clade I] from Shanghai (*N* = 11) broadly overlapped (Wilks’ λ = 0.67, F (5,11) = 1.08, p = 0.42; S16 Table in S1 File). Thus, no clear separation was detected when all traits were considered together. In contrast, the analyses of individual call parameters revealed two temporal traits with consistent divergence (S16 Table in S1 File). Call duration was significantly longer in *F. multistriata* than in *F. kawamurai* (Welch’s ANOVA: F (1,11) = 40.65, p < 0.001; temperature-corrected F (1, 12.3) = 7.26, p = 0.019), averaging 0.16 s longer per call, with a minor temperature’s effect (around 0.015 s per °C; S16 Table in S1 File; see C1; Fig 4). Call interval also showed significant interspecific divergence (ANCOVA: β = −0.11, p = 0.0012; S17 Table in S1 File), with *F. multistriata* exhibiting a mean interval about 0.11 s shorter than *F. kawamurai*, and this difference remained independent of temperature effect (see C2; Fig 4). Other traits showed no clear species differences (S17 Table in S1 File), as call rate did not differ between species (p-value = 0.99), pulse rate showed only a marginal species effect (p-value = 0.069) but was significantly influenced by temperature (β = +4.40, p-value = 0.025), and the dominant frequency was unrelated to either species or body size (all p-values > 0.55).

### Geographic patterns of taxonomic mismatch

Across the 393 verified sequence entries, taxonomic assignment and its mismatch with GenBank data showed clear differences among regions (Fig 5). Assignments to *Fejervarya kawamurai* and *F. multistriata* under Model A1 were concentrated in eastern China, particularly within the Lower Yangtze region, whereas *F. limnocharis* was more common in Mainland Southeast Asia and Sundaic regions. Other taxa were restricted to insular and peripheral regions, including Japanese Archipelago, Taiwan, and Hainan Islands (Fig 5A).

Mismatch was highest in the Lower Yangtze region (0.97) and Taiwan (1.00), and remained high across eastern China mainland (0.77) and Hainan (0.78; S18 Table in S1 File and Fig 5B and 5C), where sampling density was highest. In contrast, Mainland Southeast Asia showed the lowest mismatch (0.16), while Sundaic Southeast Asia (0.35) and Japan (0.64) showed intermediate levels, although these regions were represented by comparatively fewer entries. Overall, mismatch proportions ranged from 0.16 to 1.00, indicating consistent regional differences in taxonomic assignment.

## Discussion

We applied an integrative species delimitation framework that combines genome-wide and multilocus data with a morphology matrix, incorporating nearly all available open-access GenBank records of Dicroglossidae from the Southeastern Palearctic and Eastern Indomalaya. For the cryptic sympatric lineages in the Yangtze Plain, we further integrated ecological niche models and bioacoustic evidence. This multi-layered approach allowed us to evaluate the concordance between diverse lines of evidence and existing annotations, providing a broad baseline for future taxonomic revisions in these ecozones.

### Combined effects of allopatric divergence, cryptic sympatry, and anthropogenic influence

Allopatric divergence, cryptic diversity and anthropogenic activities may contribute to the patterns of taxonomic and geographic structure in Dicroglossidae frogs in the Southeastern Palearctic and Eastern Indomalaya. Similar interplay has been documented in *Pelophylax* water frogs, where diverged lineages in East Asia have come into secondary contact through human-mediated translocations, resulting in hybrid zones and cryptic sympatry [76,77]. Geographic segregation, shaped by ecological preferences, remains a major factor influencing the distribution of the Indomalayan *Fejervarya*, and to some extent *Quasipaa*, in the Southeastern Palearctic and Eastern Indomalaya (Fig 2). Splitting and clustering patterns in the topology of the best-supported and refined Model A1 (Fig 3) are consistent with patterns of both allopatry and cryptic sympatry. Allopatric divergence is evident in the landscape-based split within *F. multistriata* that separates Taiwan Island from continental populations, and in the deep divergence detected among *Quasipaa* species, which is notably greater than that observed in *Fejervarya* or *Hoplobatrachus*. These patterns are broadly consistent with the evolutionary framework recently reported for East Asian anurans [48]. Contrary to the previously reported dominance of *F. multistriata* in the East Asian mainland [78], cryptic sympatry is indicated by the overlap between *F. kawamurai* and *F. multistriata* lineages in the Eastern Yangtze Basin (Fig 4), although syntopy remains to be confirmed with site-level voucher or call data in future studies.

However, anthropogenic impacts are also a concern for insular populations of *Fejervarya* (Fig 4). Similarly, human activities pose a risk to the genetic integrity of closely related clades such as *Q. spinosa* and *Q. exilispinosa* which have economic value for trade [79], and occur in overlapping distributions in Southern and Southeastern China (Fig 2). In the Eastern Yangtze Basin, human-modified habitats such as paddy fields host sympatric populations of *F. kawamurai* [Clade II] and *F. multistriata* [Clade I], highlighting the role of agricultural landscapes in enhancing contact zones between cryptic taxa (Figs 2 and 3). Likewise, we observed overlapping ranges between the two subspecies on islands such as Hainan, a subtropical island that naturally supports diversity of amphibian assemblages [80], including *Fejervarya* [24] with four species, *F. limnocharis*, *F. moodiei*, *F. multistriata* and *F. kawamurai* occurring there (Figs 3 and 4). While these species are part of the island’s native fauna [19,23,24,31], our ecological niche models indicate that the continental clade of *F. multistriata* [Clade I] has a broad suitable habitat on Hainan, whereas the Yangtze clade of *F. kawamurai* [Clade II] shows suitable habitat only at the southern tip of the island under thresholded predictions (Fig 4). This ecological mismatch suggests that the two lineages were likely allopatric in origin, with the presence of *F. kawamurai* on Hainan possibly reflecting human-mediated dispersal. Further sampling and genetic checks of *F. kawamurai* populations on Hainan are needed to clarify their status.

Unlike the other two Dicroglossidae genera, the genetic structure of East Asian *Hoplobatrachus* clades may have been influenced by anthropogenic factors. Anthropogenic activities, such as trading and human introductions through farming and mercy release may have facilitated the movement of clades within species, and among intra-populations, especially in the central Chinese mainland. Here, our best-supported species delimitation model resulted in a single clade merging the introgressed clade of *H. chinensis* x *H. rugulosus* and the non introgressed clade of *H. rugulosus* across the totality of the range of the species, including the Jiangsu plain (Model A1; Fig 1).

### Geographic inconsistencies in taxonomic assignment and sampling priorities

The geographic pattern of mismatch observed (Fig 5), aligns with the distribution of closely related lineages across East and Southeast Asia (detailed taxonomic interpretation, type localities and holotypes are provided in the Supplementary Information). High mismatch in eastern China, particularly in the Lower Yangtze region (Fig 5B), likely reflects areas where multiple lineages co-occur or are geographically proximate, increasing the likelihood of inconsistent naming in public databases and legacy sequence records [31]. In contrast, lower mismatch in Mainland Southeast Asia may reflect regions where assignments have been applied more consistently across available sequences, despite relatively lower sampling density compared to eastern China. Intermediate mismatch levels in insular regions, including Japan and Sundaic Southeast Asia, may result from geographically restricted lineages, island-associated divergence patterns, and uneven sampling coverage (Fig 5B and 5C).

Trade-mediated signals are more apparent in the *Hoplobatrachus* as clade structure across East Asia suggests potential connectivity within a single regional unit (Fig 3). Although no direct signal of trade-mediated mixing is evident from the present data for *Fejervarya*, previous studies have documented human-mediated movement and genetic mixing in amphibians across Southeast and East Asia [26,81]. Similar processes have been reported in other regions beyond the focal ecozones, including island systems and major shipping hubs, where amphibian species have been introduced or translocated outside their native ranges, such as the spread of *Fejervarya* spp. to non-native localities (e.g., Guam and parts of Papua New Guinea [82]). These observations suggest that anthropogenic translocation remains a plausible but untested factor, particularly in insular systems.

### Future improvements and conservation efforts

Morphological identification alone remains ineffective for distinguishing the two most closely related *Fejervarya* species in the Yangtze Basin, as they share key identification characters, such as the absence of a marginal membrane on the fifth toe [24,53]. Our fine-scale assessment of lineage structure of Yangtze Basin *Fejervarya*, integrating ecological niche models and bioacoustics, further characterises the overlapping ranges of *F. multistriata* and *F. kawamurai* in the Yangtze Basin (Fig 4). Bioacoustic analyses revealed that *F. kawamurai* calls are shorter in duration and longer in interval compared to *F. multistriata* (Fig 4), showing acoustic differentiation similar to those reported between the *F. limnocharis* clades in Indomalaya [83]. Ecological niche modelling further indicates that the suitable habitat for *F. kawamurai* extends into the North China Plain, including Henan, Hebei, and Shandong, as well as the northern parts of Jiangsu and Anhui, extending northward to Beijing (Fig 4). A subsequent remote sensing-based ecological niche modelling study using our delimited *F. multistriata* dataset recovered broadly congruent patterns of suitable habitat [52], highlighting the potential for integrating high-resolution environmental data, with genetically validated occurrence records in future conservation assessments, especially for cryptic and widespread taxa.

In the context of ecological characteristics, the distribution of *F. kawamurai* is mostly influenced by elevation and precipitation in the driest month (S12 Table in S1 File), and this is apparent by the broad habitat preferences of the species, ranging from the north of the Yangtze River to mountainous areas of Xi’an, however, despite this range, it remains restricted by the maximum elevation of 2,000 meters [53]. The distribution of *F. multistriata* is linked most strongly to precipitation of the driest month and mean diurnal temperature range (S11 Table and Fig S2 in S1 File), aligning with tropical and subtropical climates prevalent in Southern China. Nevertheless, the substantial overlap in predicted suitable habitat indicates differences in habitat associations within broadly overlapping distributions, rather than strong ecological divergence (Fig 4). Although the overall acoustic difference was not significant, call duration and call interval differed between the two species (Fig 4C), suggesting differences in specific acoustic variables, and a potential role of mating-call differences in reducing interspecific competition and facilitating coexistence [84]. Further geographic and individual sampling will be required to determine the consistency of these differences across populations.

In the context of conservation, our results help clarify lineage assignments in traded and cryptic species, especially when these locally abundant species are vulnerable due to overexploitation or misidentification. Notably, abundant species such as *F. cancrivora* and *F. limnocharis* in Southeast Asia and Southern China were overexploited due to demands for food [28] and traditional medicine [85], promoting the introduction of exotic clade, elevated the risk of genetic loss by blurring their species boundaries [1,8]. While prioritising nationally endemic and rare species for effective species conservation management [86], efforts should also be directed towards locally abundant species [87], given the insufficient understanding of their distribution and population trends in East Asia [88]. Understanding sympatric yet cryptic species is especially crucial for conservation efforts because it helps identify areas where multiple species may compete for identical local resources or face similar threats. Conservation strategies in the two ecozones need to consider how sympatric species, such as the highly cryptic *F. multistriata* [Clade I], whose range is more limited north of the Huai River and is highly concentrated to the southeastern and central coastal landscapes in China, and *F. kawamurai* [Clade II], which is the more generalist species occurring from the north to the south of the Yangtze Basin, interact and coexist in their shared habitats. The lineage distributions recovered under Model A1 differ from the revised IUCN species distribution ranges, particularly for the three *Fejervarya* clades occurring in the East Asian mainland: *F. kawamurai*, *F. multistriata*, and *F. limnocharis* [89]. The lineage interpretation recovered under Model A1 however, aligns with local studies and is congruent with a combination of taxonomic recommendations for *Fejervarya* within the Eastern Indomalaya [29,31,35,90,91].

### Limitations of the study

We acknowledge that direct voucher re-verification is ideal, but it is often not feasible for older or inaccessible material. In such cases, we document potential inconsistencies transparently. All metadata for the samples used in this study, including GenBank accession numbers, voucher IDs, country of origin, and source publications, were carefully cross-checked against the original studies. While most records were consistent with their reported provenance and phylogenetic placement, a few required annotations to clarify potential discrepancies (S18 Table in S1 File).

Sampling coverage further contributes to the inconsistency pattern observed in the open-based species assignments. Regions with high sequence availability, such as eastern China, showed both high assignment frequency and elevated mismatch (Fig 5), indicating that increased sampling can also amplify inconsistencies when taxonomic frameworks are not uniformly applied. In contrast, insular regions, including Taiwan, Japan, and parts of maritime Southeast Asia, remain comparatively underrepresented, yet often show high mismatch proportions, highlighting priorities for targeted sampling. These mismatches, particularly where they coincide with low sampling density, underscore the need for expanded genome-wide datasets to resolve lineage identity and improve consistency in public databases. Together, these patterns demonstrate how uneven data coverage and regional lineage structure jointly shape the reliability of taxonomic assignments across large-scale sequence datasets.

The genomic component also has limitations associated with the heterogeneous public datasets. The use of a common family-level reference may have influenced initial locus recovery because of the phylogenetic distance between *Nanorana parkeri* and the focal taxa. Accordingly, we interpret the SRA-derived genomic loci as complementary evidence rather than independent evidence defining species limits. Support for Model A1 was established from the multilocus analyses before its evaluation under the total-evidence framework.

## Supporting information

S1 File Supporting information. Supplementary methods, results, discussion, figures, and tables, including S1–S3 Figs and S1–S18 Tables.
(DOCX)
