# Supplementary material for: Model-based multilocus, genomic, and phenotypic integration reveals species assignment inconsistencies and sampling blind spots across Asian ecozones in Dicroglossidae
Source: PLoS One. 2026 Oct 23;21(10):e0360243. doi: 10.1371/journal.pone.0360243 (PMC13693946; doi:10.1371/journal.pone.0360243)
Supplement: Supplementary file 1 [file pone.0360243.s001.docx]

**S1 File. Supplementary Information**

**Supplementary Methods**

**Dataset composition and** **aims**

To resolve unclear species boundaries within Dicroglossidae across the Indomalayan and Palearctic ecozones, we designed 18 alternative species delimitation scenarios based on an eight-locus multilocus dataset (4,044 bp; Table S8), including a GenBank-based assignment (Model C1) was retained as a control for comparison. Analyses were conducted on curated multilocus data, with up to 517 sequence entries included across scenarios. Dataset 1 (Pre1–Pre11; *N* putative species = 14) served as the preliminary test, while Dataset 2 (Rev1–Rev5; *N* putative species = 15) formed the final analysis against a control dataset (Model C1) that retained original GenBank species assignments without taxonomic revision. Model C1 served as a baseline for evaluating whether our scenarios-driven reassignment, improved species delimitation accuracy.

The increase in taxa from Dataset 1 to Dataset 2 reflects a shift in focus, quality control, and updates from newly available public data. We collected Dataset 1 during the initial phase, focusing on the placement of the northernmost *Fejervarya* population in the Jiangsu Plain (*F. kawamurai* vs. *F. multistriata*), the distributional limits between the shallowly divergent *F. multistriata* and *F. kawamurai* complexes, and relationships within the *F. limnocharis* complex, spanning a wide latitudinal range across the Indomalaya, from tropical to subtropical zones [1]. Dataset 2 incorporated additional data ad hoc, including 12 *F. moodiei* sequences from northern Vietnam, to clarify the validity of *F. cancrivora*, one of the most internationally traded species in the genus [2], on Hainan Island and in southern China [3]. Additionally, we examined the taxonomic assignment of *Hoplobatrachus* populations on the Chinese mainland to either non-introgressed *H. rugulosus* lineages or *H. chinensis* x *H. rugulosus* introgressed lineages [4].

**Species delimitation models: mechanisms and rationales**

We defined our models/scenarios a priori from biogeographic, climatic, anthropogenic, and morphological patterns, cross-checking them against systematic treatments and recent taxonomic revisions [4–7], and incorporating distributional context from open-access sources such as the IUCN Red List [8]. In addition, morphometric and meristic criteria such as small tympanum, head, limb and foot size, Snout-vent-length, tibia length, eyes and tympanum diameters, and nostril-to-tympanum distance (S8 Table), that are especially meaningful for *Fejervarya* [3,5,9,10], were included in the evaluation of species assignment, alongside multilocus phylogenies to evaluate whether similarity reflected shared ancestry or convergence, informing splitting vs. clustering outcomes.

All 18 species delimitation models explicitly centered on splitting versus clustering of lineages, and taxonomic reassignment. Our main scenarios addressed four recurrent uncertainties, arranged from northernmost to southernmost Dicroglossidae clades within the two focal ecozones: (a) examining whether the northernmost *Fejervarya* population in the Jiangsu Plain, at the margin of the Yangtze Basin, clusters with *F. kawamurai* or with *F. multistriata*, thereby clarifying the distributional boundary between these two morphologically similar complexes; (b) splitting versus clustering within the *F. limnocharis* complex, which spans a wide latitudinal range across the Indomalaya, from tropical to subtropical zones; (c) taxonomic reassignment between *F. moodiei* and *F. cancrivora* across Hainan Island, the East Asian mainland, the Southeast Asian mainland, and Sundaland; and (d) splitting versus clustering of *Hoplobatrachus* populations on the East Asian mainland, assigning them to either non-introgressed *H. rugulosus* lineages or *H. chinensis* x *H. rugulosus* introgressed lineages. While the preliminary scope also included *Quasipaa*, the species of the genus were delimited with deep divergences, and thus received less emphasis in the final model comparisons.

Each model aligned with one or more delimitation rationales: (i) allopatry and sympatry patterns, by testing segregation between continental and island populations and between Southeast Asian mainland versus Sundaland, or testing segregation between Taiwan and Ryukyu versus continental East Asian clades (Pre1, Pre2, Pre4, Rev1, Pre6–Pre8); (ii) latitudinal climatic gradients, by examining widespread species such as *F. limnocharis* across climatic transitions, from tropic to subtropic in Indomalaya (Pre3, Pre5, Pre9, Rev2, Rev3); (iii) anthropogenic introduction and trade-mediated dispersal, by assessing whether *F. cancrivora/F. moodiei* clade in Hainan reflects human-assisted introduction, and testing if *H. rugulosus* should be treated as a single non-introgressed clade or split to account for introgressed lineages (Pre10, Pre11, Rev4, Rev5); and (iv) homology versus homoplasy, by evaluating whether morphologically similar but genetically distinct clades warrant merging or splitting (Pre10, Pre11, Rev4, Rev5, A1). Refinement of the most strongly supported model resulted in this section with 33 genome-wide data (Dataset 3) is described in later subtopic.

**Comparative BFD analysis of species-delimitation scenario**

Following the scenario-testing framework described above, we reconstructed calibrated species trees for each competing model using multilocus datasets 1 and 2, which comprised eight unlinked loci (4,044 bp) from 405 samples, yielding up to 517 sequence entries in StarBEAST. Species tree reconstruction was conducted in StarBeast2 v.2.6.7 [11] under a relaxed lognormal molecular clock within a multispecies coalescent framework, with input .xml files generated in BEAUti v.2 [11]. Next, we imported and processed our alignment dataset under the SpeciestreeRLC template and determined the data partition for each locus with the bModelTest site model [12], integrated into the toolbox of the software. We defined the ploidy level for each locus (2.0 for diploid and 0.5 for haploid) and fixed the population to the default parameter using an analytical population size integration model.

We conducted the dating estimation analysis in StarBEAST RLC v.2.6.7 [13], and sampled for two independent MCMC runs (10 million iterations) with convergence diagnosed in (ESS > 200). The Maximum Clade Credibility (MCC) tree was constructed after discarding 50% of posterior trees, providing a robust evolutionary framework. Species boundary maps were reconstructed using IUCN range data and paleo-atlas projections to infer biogeographic patterns. For the multilocus datasets, we applied a Yule prior to model species diversification, whereas for the genome-wide refinement dataset we implemented a fossilised birth-death (FBD) tree prior as the final divergence dating analysis. To establish a consistent phylogenetic framework for evaluating alternative species assignments and topological constraints, we evaluated 18 comparative species delimitation models (from Pre1 to Pre11, from Rev1 to Rev5, and between C1 and A1) under identical settings of StarBeast2 v.2.6.7 [11].

We assessed the relative support of all the 18 competing topologies using nested sampling method in a Bayesian computation [14]. To do so, we further analysed all datasets independently using the NS model test package [15] embedded in BEAST v.2.6.7 [11]. We resampled each NS analysis for 10,000 replicates, with the particle count for sampling set to 14. We evaluated each topology model using the Marginal Likelihood Estimation (MLE) values and Bayes Factor (S6 Table), generated by the NS log analyser in BEAST v.2.6.7 [11]. Higher MLE values indicate better model fit, and positive Bayes Factors were used to assess relative support, with ranking procedures described later. Model fit from the concatenated SNP supermatrix comparisons of C1 versus A1, based on both multilocus and genome-wide datasets, is reported in S14 Table.

**Integration of morphology and genome-wide data into existing best-supported model**

We integrated a genome-wide sampling strategy to enhance species-level resolution and refine both the delimited tree and its calibration times, building on the most strongly-supported multilocus dataset (Model A1; Tables S8-S9). To achieve this, we assembled a genome-wide dataset of 33 samples representing *Quasipaa*, *Fejervarya*, and *Hoplobatrachus* covering within the focal ecozones. We retrieved raw sequence data from the NCBI Sequence Read Archive (SRA), selecting a combination of genotyping-by-sequencing (GBS), whole-genome/whole-cell shotgun (WGS/WCS), and transcriptome (RNA-seq) libraries. Overall, within *Quasipaa*, we selected 10 GBS-derived SNP sequences of *Q. boulengeri* and incorporated three WGS/WCS sequences representing *Q. boulengeri*, *Q. shini*, *Q. spinosa*. We also included two transcriptomic sequences of *Q. spinosa*. Within *Fejervarya*, we assembled 17 samples, including three WGS/WCS libraries from *F. kawamurai*, *F. limnocharis*, and *F. multistriata*, along with 17 RNA-seq libraries representing *F. cancrivora*, *F. kawamurai*, *F. limnocharis*, and *F. multistriata*. Finally, we added three RNA-seq libraries of *H. rugulosus* to represent *Hoplobatrachus* (S7 Table). We aligned all genome-wide data to the *Nanorana parkeri* reference genome, <https://www.ncbi.nlm.nih.gov/datasets/genome/GCF_000935625.1/>, a representative for the family Dicroglossidae with chromosome-level assembly with high scaffold continuity.

We filtered for quality and trimmed the adapters for the raw reads using fastp v.0.23.4 [16]. For GBS data, we aligned reads with BWA-MEM v.0.7.17 [17] and called SNPs using the Stacks v.2.66 pipeline [18]. For WGS/WCS data, we aligned the reads to the reference genome using BWA-MEM and processed the resulting alignment with the GATK v4.6 germline variant discovery pipeline [19]. We sorted and indexed the resulting Binary Alignment/Map (BAM) files using SAMtools v.1.17 [20] and marked PCR duplicates with Picard v.3.1.1 [21]. For RNA-seq data, we aligned reads using HISAT2 v.2.2.1 [22], which supports spliced alignments. We sorted the resulting BAM files, marked the duplicates, and processed intron-spanning reads with GATK v.4.6 using “SplitNCigarReads” function [19]. Under the same GATK pipeline, we called variants using “HaplotypeCaller” function in genomic variant call format (GVCF) mode. We then combined all GVCFs with “CombineGVCFs” function, and performed joint genotyping with “GenotypeGVCFs” function. We filtered all VCF output files obtained from the GBS, WGS/WCS, and RNA-seq pipelines using VCFtools v.0.1.16 [23] and BCFtools v.1.17 [24], retaining only biallelic SNPs with lesser than 10% missing data, and a minor allele frequency more than 0.05. We then sorted, compressed, and indexed the filtered SNP datasets using BCFtools and Tabix v.1.17 [25].

To prepare input for species tree estimation, we subdivided genetic variants into putatively unlinked loci using a sliding window approach in VCFtools v.0.1.16 [23], with a 500-bp window for nucleotide diversity calculations. Here, we retained only the windows containing at least 15 variants to reduce noise while preserving sufficient phylogenetic signal for accurate species tree inference [26,27] and extracted these variants from each retained window using chromosome and base pair coordinates. We then converted the locus-specific VCFs into NEXUS format in batch using vcf2phylip v.2.0 [28], and retained only informative loci with complete data and sufficient taxon representation for downstream species tree analysis. Finally, including eight multilocus markers, we extracted 155 unlinked loci from genome-wide datasets comprising 70 RNA-seq loci, 13 WGS/WCS loci, and 64 GBS-derived SNP loci, totalling 6,983 sites, and each locus containing an average of 20 variants.

In parallel, we constructed a morphological character matrix encompassed diagnostic features of *Fejervarya* species within three major complexes spanning the Southeastern Palearctic and Eastern Indomalaya, from published studies to provide an additional line of evidence preceding the application of genome-wide markers. We coded 19 morphological and morphometric traits previously emphasised in *Fejervarya* taxonomy, including tympanum size, head proportions, limb length, foot size, and other body size–related traits (see S8 Table for complete list and references) in binary format, with “1” denoting the presence of a trait and “0” denoting its absence in a species. The resulting morphological matrix was converted into NEXUS format and integrated with the genome-wide dataset in StarBEAST analyses, allowing us to jointly evaluate morphological and genomic signals within the framework of our best-supported model (Model A1).

**Calibration of species trees divergence-time**

To calibrate each species tree across the 18 models, we did not rely on primary fossil constraints due to the lack of fossil evidence for the focal genera. Instead, we applied a secondary calibration to constrain the root age of Dicroglossidae in East Asia, based on published estimates placing its basal divergence between the Late Palaeocene and Early Eocene (c. 59 HPD 95%: 55–66 Mya; Chen et al. 2017). This timeframe aligns with the broader hypothesis of a major radiation of anurans following the Cretaceous-Paleogene (K-Pg) extinction, c. 65 Mya [30], providing a cross-check for the plausibility of the root age. We assigned all three calibrations to normal distributions due to the absence of direct fossil constraints. We did not assign other clades to absolute ages, but they were grouped as monophyletic to help stabilise topology without imposing temporal constraints. The mean, sigma, offset, and 5%–95% quantile values for each calibration, along with full parameterisation details, are provided later.

Species trees were reconstructed in StarBEAST2 under a multispecies coalescent framework, applying a relaxed lognormal clock model (RLC [13]). For each locus, substitution rates were estimated within the species tree framework, with a random local clock model and a gamma prior (α = 0.5, β = 2.0). For ribosomal and mitochondrial loci, an initial clock rate of 3.5 × 10⁻⁹ substitutions per site per year was applied based on previous estimates for eastern Palearctic anurans [31]. A Yule process was used as the tree prior with default parameter settings.

For each tested multilocus species tree, we performed two independent analyses on the .xml file for the species tree dataset previously generated, using the 20 million iterations of Markov Chain Monte Carlo (MCMC) procedure using Beast v.2.6.7 [11]. The adequacy of the MCMC sampling was assessed using Tracer v.1.7 [13], for the effective sample size (ESS) values of each parameter to be above 200. We assembled all generated trees in LogCombiner v.2.6.1 [11] and summarized them with a Maximum Clade Credibility (MCC) tree after discarding 50% of the trees with a posterior probability limit of 0.5 using Tree Annotator v.2.6.3 [11]. Finally, we reconstructed the species boundary maps with the ranges downloaded from the IUCN Red List of Threatened Species for each species and with the projection of the clades’ composition based on our best-supported species tree on the paleo-atlas dataset adapted from Matthews et al. [32] using QGIS v.2.1.8 [33].

In addition, for the genome-wide refinement of the best-supported Model A1 species tree, we revised the dating calibration by exploring alternative secondary calibration points derived from previous mitochondrial-based estimates [29,34], applied as normal priors: (1) the divergence between *Hoplobatrachus rugulosus* and *H. tigerinus* (mean = 6.5, σ = 2.5, offset = 10.0); (2) the emergence of *Fejervarya* in Southeast and East Asia (mean = 6.5, σ = 2.5, offset = 10.0); and (3) the stem emergence of continental East Asian *Quasipaa* (mean = 18, σ = 3, offset = 20.0). These calibrations were used to assess the robustness of divergence estimates under alternative temporal constraints (S15 Table for the detailed calibration points).

For the final analyses, we used a relaxed lognormal clock model and a fossilised birth-death (FBD) speciation prior [35] in StarBEAST RLC v.2.6.3 [11,13]. We conducted an initial MCMC run of 500 million generations, sampling every 50,000 generations. To improve effective sample sizes across parameters, we performed two additional independent runs of 250 million generations each (resampling every 2,500 generations) and one independent run of 50 million generations that included the morphological matrix (resampling every 500 generations), yielding a total accumulated sampling effort of 1.05 billion generations. The reduced logging interval in the latter runs increased the number of retained samples without extending runtime. All runs were conducted on separate workstations, and the resulting trees were combined in LogCombiner v.2.6.3 [11], with resampling every 2,500 generations and discarding the first 10% of samples as burn-in. Convergence and effective sample sizes (ESS) of key parameters were jointly assessed in Tracer v.1.7 [36].

**Model evaluations with MLE and BF**

Based on the Marginal Likelihood Estimation (MLE) values, we compared all 18 species trees and selected the topology model that generated the highest MLE value and positive Bayes Factor (BF). The calculation of the BF value for each model is based on the equation:

$${BF=2 \times[(MLE of Model}_{x}) -({MLE of Model}_{0})],$$

Model*_x_* to represents any selected model and Model*_0_* represents the competing or default model. Here, we considered the default model to be the species tree containing taxa assigned under their original epithets in GenBank, in comparison to the other 17 competing species delimitation models.

**Model comparison of supermatrix SNP dataset: control GenBank-based Model C1 versus optimal Model A1**

After integrating supermatrix SNP of We validated our best-supported species tree Model A1, against the control model based on GenBank assignments Model C1 (Fig. 1), using model selection with path sampling [17] and Bayes Factor analysis of marginal likelihoods [37]. To obtain the marginal likelihood estimation for the two competing topologies, we specified the parameters in the GSS editor under BEAST v.2.7.3 [11] for generalized stepping-stone sampling [38], with an alpha value of 0.3, and 50 number of steps, a chain length of 1 million, and a 10% burn-in percentage.

**Adjusted Ecological Niche Modelling with species delimitation**

We combined species delimitation model and a maximum-entropy algorithm (MaxEnt) to predict the suitable habitat of the two most cryptic clades that currently, publicly labelled as *F. limnocharis* in GenBank annotation (Table S6), *F. kawamurai* and *F. multistriata*, as they are expected to be partially sympatric at the boundary between the Northeastern Indomalaya and Southern Palearctic ecozones. Considering the uncertainty and high crypticity in distribution, genetics, and morphology between the clades of *F. multistriata* and *F. kawamurai* in mainland East Asia, we relied on presence-only data as our sampling assumption. We collected occurrence data for *F. kawamurai* and *F. multistriata* through both field surveys and the Global Biodiversity Information Facility (GBIF.org). We minimised potential misidentifications in open-source records, particularly for cryptic taxa, by removing entries lacking essential metadata, excluding points outside the revised range, and verifying photographic vouchers where available. For *F. multistriata*, we used an occurrence dataset integrating our field survey data with vetted GBIF records and remote-sensing variables to produce high-resolution habitat suitability models. This prior validation [39], reinforces the ecological plausibility and reliability of the occurrence data used in this study.

We carried out the field surveys within each cell of a 10 km grid square overlayed across the western Jiangsu Plains, starting from the Yangtze River continuing 100 km towards the north. In addition, we conducted opportunistic surveys in suitable habitat at the known northern limit of *Fejervarya* in the Palearctic [7]. We determined the occurrences of the species through aural surveys conducted at each site for 10 minutes, a tested protocol valid for the detection of calling amphibians in this landscape [40]. We conducted all surveys during the peak breeding season of the species. From GBIF, we obtained 2,076 occurrences data for *F. kawamurai* (DOI: 10.15468/dl.n889nn, accessed on 11 October 2023) and 1,427 presence records for *F. multistriata* (DOI: 10.15468/dl.tdd63a, accessed on 11 October 2023). As the two cryptic species are also sympatric, we then delimited the erroneous occurrences in both datasets based on our best-supported species delimitation model produced in previous section. To reduce the impact of spatial sampling bias, we applied a 10 km thinning procedure for the spatial occurrences [41] using the “spatially rarefy occurrence data for SDM” tool in ArcGIS v.10.8 [42]. The final dataset contained 286 occurrences of *F. kawamurai* and 597 occurrences of *F. multistriata*.

We then downloaded 19 bioclimatic variables from WorldClim v.2 (www.worldclim.org, Fick and Hijmans, 2017) at 30” resolution and an elevation layer from the ASTER GDEM v.3 dataset [44], all projected to WGS 1984 projection system in ArcGIS v.10.8 [42]. Next, independently for the datasets of both species, we extracted the environmental variable at each point of occurrence and calculated the correlation amongst all variables. To prevent overfitting caused by multicollinearity among environmental variables [45], we excluded specific variables to ensure that their correlations did not exceed 0.7 (|r| < 0.7), using ‘cor’ function in R package ‘stats’ v.4.4.2 [46]. We retained the following variables: mean diurnal temperature range (Bio 2), isothermality (Bio 3), maximum temperature of warmest month (Bio 5), mean temperature of wettest quarter (Bio 8), mean temperature of warmest quarter (Bio 10), annual precipitation (Bio 12), precipitation of driest month (Bio 14), precipitation seasonality (Bio 15) and elevation (S3 Fig.).

We set each ecological model using the default parameters in Maxent v.3.4.1 [47]. We ran a jackknife analysis to measure variable importance and included all samples in the background in the advanced parameter settings to later generate the true skill statistic (TSS) value to evaluate the accuracy of each model. The ‘replicated run type’ was set to ‘Crossvalidate’ and output files were set to the Cloglog format. We randomly allocated 75% of the data for model training and 25% for testing in all runs. Each model was run with 10,000 background points and 10 replicates. We assessed the performance under present climate conditions using the Area Under the Receiver Operating Curve (AUC), as AUC is a threshold independent measure of predictive accuracy based only on the ranking of location [45]. Our models were considered having excellent discriminatory ability with all AUC values exceeding 0.8 [48]. We also estimated the accuracy of the models using the TSS methods [49], which provides a single value based on the maximum value of the F-measure, which is a metric commonly used to evaluate the accuracy of binary classification models [50]. We ran two separate models using the same settings and visualized them using different threshold values. The first used the minimum predicted value of training data as the threshold (minPred), a method designed specifically for this type of model, adapted from previous literature [50,51]. The minPred values represent the minimum value of all cells in the prediction area and depended on the prevalence of focal *Fejervarya* species in the area and the variables used by the models. We classified the habitat into unsuitable (<minPred) and suitable habitat (>minPred). For the second, we classified the MaxEnt output rasters using the maximum training sensitivity plus specificity (MTSS) threshold, also an ideal threshold because it minimises the influence of background data and is a stricter minimum threshold than minPred [52].

**Supplementary Results**

**Multilocus-based divergence time estimation**

Using multilocus data, we estimated the origin of Dicroglossidae in eastern Indomalaya to the Eocene, c. 54.05 Mya (Fig. 2). This was followed by the divergence between *Quasipaa* and *Hoplobatrachus*, c. 47.56 Mya (HPD 95% 63.09–32.17 Mya; Fig. 2). In the later period, the stem clade of eastern Indomalayan *Quasipaa* emerged during the transition between the Late Oligocene and Early Miocene, c. 25.58 Mya (33.11–17.24 Mya; see Q1 in Fig. 2). Here, we estimated the split between two major groups within the eastern Indomalayan *Quasipaa*, represented by the emergence of a clade containing *Q. boulengeri*, *Q. shini*, and *Q. yei* in central China c. 21.48 Mya (see Q2 in Fig. 2), followed by the emergence of a much younger clade containing *Q. jiulongensis*, *Q. spinosa,* and *Q. exilispinosa* c. 16.54 Mya (25.19–9.48 Mya; see Q3 in Fig. 2). Later, the stem clade of *Hoplobatrachus* emerged during the Middle Miocene, c.11.03 Mya (HPD 95%: 19.92–4.33 Mya; see H in Fig. 2). This stem clade clustered *H. tigerinus*, which was restricted to South Asia and the Southeast Asian Mainland, and *H. rugulosus*, which originated from continental Southeast Asia but then emerged in mainland East Asia (labelled here as *H. chinensis* in Fig. 2).

In the southeastern Palearctic and eastern Indomalaya realms, the genus *Fejervarya*, representing the youngest group within Dicroglossidae, emerged as clade assemblages during the Late Miocene, c. 7.14 Mya (12.08–2.24 Mya; see F1 in Fig. 2). The clade assemblages of *Fejervarya* were represented by the divergence between *F. sakishimensis*, occurring in the Ryukyu Islands and northern portion of Taiwan Island, and a clade containing three *Fejervarya* species complexes that emerged across East Asia and Southeast Asia since the Pliocene, c. 3.72 Mya (7.96–1.96 Mya; see clade assemblages labelled as F1; Fig. 2). The three major *Fejervarya* species complexes were nested as follows: (a) Clade I of *F. kawamurai* was distributed in southeastern coastal China such as Guangzhou, Zhoushan, the Japanese Archipelago, and Taiwan Island, and Clade II distributed across the central and eastern regions of the Chinese Mainland, extending up to Beijing (c. 2.14); (b) Clade I of *F. multistriata* was restricted to Taiwan Island, while Clade II of *F. multistriata* is locally widespread in the central mainland and central to eastern coastal regions of China, with Clade II of *F. multistriata* having diverged from Clade II of *F. kawamurai* c. 1.95 Mya (3.09–0.71 Mya; Fig. 2); (c) the *F. limnocharis* species complex emerged across Southeast Asia, diverging into two clades: Clade I, restricted to Sundaland, covering Indonesia and its smaller islands, Peninsular Malaysia, and Borneo, and Clade II occurring across continental Southeast Asia (Fig. 2).

The diversification of the two independent *Fejervarya* lineages in the eastern Indomalaya resulted in the formation of the *F. cancrivora* and *F. limnocharis* species complexes between the Middle Pleistocene and Holocene. The late radiation within the eastern Indomalayan *Fejervarya* was indicated by the emergence of the *F. cancrivora* species complex in the southern Sundaic (covering the Southern Thailand, Malay Peninsula, Sumatra, Borneo, Java, and the surrounding smaller islands), and the formation of the closely related species *F. moodiei* in continental Southeast Asia (Thailand, the northern region of Vietnam, Philippines) and Hainan Island, c. 0.85 Mya (1.77–0.02 Mya; see F2 in Fig. 2). Subsequently, the *F. limnocharis* complex formed and diverged into two lineages, c. 0.55 Mya (1.42–0.02 Mya; see F3 in Fig. 2). One lineage was prevalent in the southern Sundaic region, while the other lineage extended across continental Southeast Asia, reaching as far as Southern China.

**Supplementary Discussion**

**Taxonomic interpretation based on lineage delimitation patterns in Dicroglossidae**

Here, we treated public sequence records as starting points for evaluation rather than definitive authorities. We do not assume that GenBank annotations are inherently correct or incorrect, and we do not enforce any taxonomic changes, instead, we assessed how well the available data align with existing labels and highlight cases that may warrant closer review.

Under our optimal species delimitation model (A1), non-introgressed and introgressed *Hoplobatrachus chinensis* form a single clade (see lumping model in Fig. 3), consistent with phylogeographic evidence suggesting that *H. chinensis* in East Asia may include multiple cryptic lineages [4]. While we did not directly assess admixture, prior studies have documented trade-mediated genetic mixing in related *H. rugulosus* [53], through imports from Thailand and Vietnam for farming [2], suggesting that both international and domestic trade may contribute to lineage blurring in farmed and wild populations in China. Taxonomically, we support the recognition of the *Hoplobatrachus* populations in Mainland China as *Hoplobatrachus* *chinensis,* based on the principal of priority for *“Rana” chinensis* Osbeck, 1765, in comparison to the year of description for *“Rana” rugulosus* Wiegmann, 1834. We suggest that despite prior disagreement concerning the validity of *H. chinensis* [56], our integrative species delimitation reliably support the validity of *H*. *chinensis*, and this clade corresponds to the similar clade previously identified as “*H.* cf. *rugulosus*” [6].

The time-calibrated species trees from the refined Model A1 date the divergence of the *Fejervarya* superclade to the Early Miocene, c. 19.0 Mya, leading to the radiation of three major species complexes and their respective subclades across Indomalaya and Palearctic ecozones (nodes c and h; Fig. 3). *Fejervarya* superclade diverged into distinct clades, with estimated divergence times suggesting: (a) the splitting of the *F. kawamurai* species complex into two clades in East Asia c. 11.51 Mya (node i; Fig. 3). Clade I occurs in the Japanese Archipelago (type locality: Hiroshima, Japan), Southern China (e.g., Guizhou), the southeastern to the central coast of China (from Guangzhou to Shanghai), and the Zhoushan Archipelago. Clade II of this species occurs in central China (e.g., Henan, Zhejiang, central to southern Jiangsu) and Hainan Island. However, some populations of *F. kawamurai* in the eastern portion of the Zhoushan Archipelago (Kuzhu and Huangxing) cluster within Clade II (Table S8). Overlap between the two *F. kawamurai* clades has been reported previously [57], though labelled under the epithet “*F. limnocharis*” in GenBank. Even more than a decade after the last taxonomic revision covering *F. kawamurai* and *F. limnocharis* [5], there remains a need for greater consistency in species name application across the literature. Our revised species boundaries with phenotypic and ecological modelling indicate that *F. kawamurai* predominates in the East Asian mainland north of the lower Yangtze, extending inland across the Jiangsu Plain and adjacent floodplains, and occurring in both coastal and inland wetlands (Fig. 3). We therefore encourage consistent application of *F. kawamurai* for these mainland clades, particularly when interpreting or citing studies on morphology [58], ecology [59], life history [60–62] and evolution [59] of the species.

Recently, Tong et al. (2025) described *F. zhoushanensis* sp. nov. (type locality 122.11° E, 30.371° N) for Clade I populations from the Zhoushan (Fig. 3). Their GBS phylogeny grouped Pujiang, Pinghu, and Beilun with Zhoushan, whereas our broader dataset supported the clustering of some Pinghu individuals in Clade II, indicating two coexisting clades in Zhejiang. As their study did not compare Zhoushan clade to the Japanese *F. kawamurai* lineage from the type locality (Hiroshima, type locality 34.383° N, 132.700° E, alt. 200 m a.s.l), the only prior cross-regional genetic comparisons used allozymes [63] and Cyt*b* [57]. To address this, we incorporated all publicly available Japanese *F. kawamurai* data, including nine multilocus GenBank accessions, one WCS, and one RNA-seq data, into our refined model, testing two species delimitation scenarios: a model with three clades that separates Hiroshima, Clade I (Zhoushan), and Clade II, and a model with two clades that groups Zhoushan with Hiroshima as Clade I against Clade II. Optimal Model A1 supported the latter, with a higher MLE than the three-clade hypothesis (Pre1; Fig. 1). Nevertheless, the forthcoming public GBS dataset of Tong et al. (2025) can be integrated into our ready-made dataset for comprehensive reassessment.

(b) The splitting of *F. multistriata* species complex into two diverged clades occurred c. 6.29 Mya (node h; Fig. 3). Clade I is distributed in southern Mainland China (Sichuan), with a concentration in southeastern (Guangdong and Fujian), including Hong Kong (type locality) to central coastal China (Shanghai), and is locally abundant in Hainan Island. Moreover, our adjusted ecological niche modelling and calls analysis revealed the presence of *F. multistriata* [Clade I] in the east of the Yangtze River Basin, with the northern limit of the species associated with the extent of the Huai River (Fig. 4). The species also shows higher suitability along the central coastal region of China to the south, contrasting with the occurrence of *F. kawamurai* that is more generally distributed. Clade II of *F. kawamurai* was distributed further north than *F. multistriata*, covering areas from Jiangsu, Shandong, and Beijing along the west coast of the Yellow Sea (Fig. 4). Clade II is distributed from the southern to eastern part of Taiwan Island (Yilan, Pintung, Hualien) and some part of the Ryukyu Islands, such as Okinawa. Some *Fejervarya* presently labelled as “*F. limnocharis*” in GenBank distributed in Taiwan Island are instead clustered within Clade I of *F. multistriata* (Table S8).

(c) The splitting of *F. cancrivora* from *F. moodiei*, c. 13.74 Mya (node j; Fig. 3). *Fejervarya cancrivora* clade is limited to the Sundaland regions encompassing Indonesia and its smaller islands (type locality: Java; Dubois and Ohler, 2000), Peninsular Malaysia and Borneo, and merged with its synonymised clade previously known as “*F. raja*” [65], distributed across Southern Thailand (Fig. 3). *F. moodiei* is distributed in the Philippines (type locality: Manila, Luzon, Taylor, 1920; McCoy and Richmond, 1966), Southern China including Guangdong and Guangxi, the northeastern coast of Hainan Island, Vietnam, along the Gulf of Thailand, and Peninsular Malaysia (Fig. 3). The *F. cancrivora* clade also shared genetic homogeneity with *F. moodiei* distributed in the western Indomalaya, including the Andaman and Nicobar Islands, Bangladesh, Odisha [68,69], and extending to West Bengal in Eastern India [70]. Despite trade of the species from Southeast Asia to East Asia [71], the delimited species boundaries clarify the exotic clade of *F. cancrivora* on Hainan Island and along the coastline of Southern China [72], which based on the currently available sequences on GenBank clustered within the *F. moodiei* clade (Fig. 3). This aligns with previous findings that classified the species complex into three types based on allozymes, ecological, and morphological traits: the large-type as *F. cancrivora* in the Eastern Indomalaya, the mangrove-type as *F. moodiei* in the northern Indomalaya, and an undescribed Sulawesi-type *Fejervarya* species [73], although, the Sulawesian clade was not included in the present study. Apparently, both of these *Fejervarya* clades inhabit mangroves [3], but *F. cancrivora* is extremely tolerant of saline conditions [74] and is more of a generalist as it can also be found in rice paddy fields in Sundaland [75].

(d) The splitting of the most generic species complex, *F. limnocharis*, into two clades in the Indomalaya, c. 15.78 Mya (node k; Fig. 3). Previously, Kotaki et al., (2008) resolved three haplotype clades within *F. limnocharis* in continental Southeast Asia and northern Sundaic regions (including Peninsular Malaysia and Borneo), focusing on local-scale genetic structure. Haplotype 1 spanned both continental and Sundaic populations, indicating that their units were not strictly allopatric. Haplotype 2 was more localised to the continent, whereas haplotype 3 overlapped with haplotype 1 along the Thailand-Myanmar border (Pilok), further illustrating incomplete geographic segregation. As these haplotypes remain annotated as *F. limnocharis* in GenBank, their inclusion is congruent with our broader framework. By avoiding the risk of over splitting or misinterpreting local haplotype variation as species-level divergence, our multilocus, ecozone-wide analyses revealed that these haplotypes segregate across two species-level clades (Clade I and Clade II). Clade I is prevalent in Southern Sundaic, covering Indonesia and its small islands, Java, Peninsular Malaysia and Borneo (Sarawak, Kalimantan), southern Thailand such as Ranong, Nakhon Si Thammarat, and extending into central Thailand such as Saraburi. Clade II is distributed in southern China, including Yunnan, and prevalent across the Southeast Asian mainland, covering Myanmar, Vietnam, Laos, and Central and Northern Thailand, such as the Nam Lang River, Pang Ma Pha (Sappong), Chiang Mai, and Chao Phraya River Basin, and extending to the northern coast of the Andaman Sea (Fig. 3). Some individuals clustered within Clade I were also distributed in the continental portion of Southeast Asia, such as northwestern Vietnam (Sapa), Phongsaly (boundary between Vietnam and Laos), and central Thailand (Bangkok), as well as Hainan Island. Moreover, there are two holotypes associated with Clade I: (a) Holotype: RMNH 4287 (neotype); type locality: not stated, but from context clearly Java, Indonesia [64]; and (b) Holotype: ZSIC 17282; type locality: Kuching, Sarawak, Malaysia [64]. Considering the geographic segregation within some lineages in Clade I, and a recent split between Clade I and Clade II in the Indomalaya, it is likely that many genetic and morphological characteristics are still shared by the lineages within Clade I. We recommend a careful taxonomic update for the populations in the Southern Sundaic to ensure correct assignment between *Fejervarya limnocharis limnocharis* and *Fejervarya limnocharis wasl* and to clarify whether there is a subspecies boundary between them.

**Supplementary Figures**

**S1 Fig. Phylogeny inferred from mitochondrial control region (*CR*) sequences across 112 taxa.** The orange-highlighted clade represents four individuals from our bioacoustic sampling site, which cluster within the cryptic *Fejervarya kawamurai*-*F. multistriata* complex of the Yangtze Basin.

**S2 Fig. Sample-level sequence missingness across genomic datasets retained for Model A1.** Bars show the percentage of missing or ambiguous characters (N and ?) within the genomic alignments retained for each of the 33 samples. Samples are grouped according to the number of retained loci (70, 64, and 13 loci), corresponding to RNA-seq, GBS, and WGS/WCS datasets, respectively. The vertical dashed line indicates the overall median sequence-level missingness across all samples (1.54%). Horizontal dotted lines separate the three locus-recovery groups.

**S3 Fig. Correlation results for reserved environment variables from 20 ecological variables tested for Ecological Niche Modelling.** We exclude specific variables to ensure that their correlations did not exceed 0.7 (|r| < 0.7).

**Supporting tables**

**S1 Table. Entire sampling information of Dicroglossids distributed in targeted area and sequences leveraged from GenBank.** The accession numbers of 825 sequences represented by 486 Dicroglossidae individuals distributed across Eastern Indomalaya and Southeastern Palearctic. Correction in the epithet labelling matched to the model and species boundary.

| This study | *Genbank* | Voucher | Locality | Lat | Lon | 12S rRNA | 16S rRNA | *Cytb* | *COI* | D-loop | *TYR* | *RAG1* | *Rho* | Reference | |
| --- | --- | --- | --- | --- | --- | --- | --- | --- | --- | --- | --- | --- | --- | --- | --- |
| *Fejervarya kawamurai* (Clade II) | *Fejervarya kawamurai* | 21Fmc001 | Southeastern Jiangsu, China | 31.978 | 118.942 | PP855682 | PP854435 | PP866703 | PP886254 | PP866701 |  | PP883015 | PP883022 | This study |  |
| *Fejervarya kawamurai* (Clade II) | *Fejervarya kawamurai* | 21Fmc002 | Southeastern Jiangsu, China | 31.978 | 118.942 | PP855683 | PP854433 | PP866704 | PP886255 | PP866700 |  | PP883019 | PP883029 | This study |  |
| *Fejervarya kawamurai* (Clade II) | *Fejervarya kawamurai* | 21Fmc003 | Southeastern Jiangsu, China | 31.978 | 118.942 | PP855684 | PP854428 | PP866705 | PP886256 | PP866698 | PP889508 | PP883020 | PP883025 | This study |  |
| *Fejervarya kawamurai* (Clade II) | *Fejervarya kawamurai* | 22Fmc001 | Central Jiangsu, China | 33.135 | 119.497 | PP855685 | PP854431 | PP866706 | PP886257 | PP866699 | PP889507 | PP883018 | PP883024 | This study |  |
| *Fejervarya kawamurai* (Clade II) | *Fejervarya kawamurai* | 22Fmc002 | Central Jiangsu, China | 33.135 | 119.497 | PP855690 | PP854429 | PP866707 | PP886258 | PP866694 | PP889510 | PP883017 | PP883030 | This study |  |
| *Fejervarya kawamurai* (Clade II) | *Fejervarya kawamurai* | 22Fmc003 | Central Jiangsu, China | 33.081 | 119.569 | PP855686 | PP854436 | PP866708 | PP886259 | PP866695 | PP889509 | PP883014 | PP883023 | This study |  |
| *Fejervarya kawamurai* (Clade II) | *Fejervarya kawamurai* | 22Fmc004 | Northern Jiangsu, China | 34.156 | 118.546 | PP855687 | PP854434 | PP866709 | PP886260 | PP866696 |  | PP883021 | PP883026 | This study |  |
| *Fejervarya kawamurai* (Clade II) | *Fejervarya kawamurai* | 22Fmc005 | Northern Jiangsu, China | 34.156 | 118.546 | PP855688 | PP854432 | PP866710 | PP886261 | PP866697 |  | PP883013 | PP883027 | This study |  |
| *Fejervarya kawamurai* (Clade II) | *Fejervarya kawamurai* | 22Fmc006 | Northern Jiangsu, China | 34.156 | 118.546 | PP855689 | PP854430 | PP866711 | PP886262 | PP866702 | PP889511 | PP883016 | PP883028 | This study |  |
| *Fejervarya kawamurai* (Clade II) | *Hynobius chinensis* | 22Hic001 | Northern Jiangsu, China | 33.127 | 118.802 |  |  |  |  |  |  | PP883012 | PP883031 | This study |  |
| *Fejervarya kawamurai* (Clade II) | *Fejervarya limnocharis* | HNFL01 | Hainan Island |  |  |  |  | MK317496 |  |  |  |  |  | Yang et al. (2022) |  |
| *Fejervarya kawamurai* (Clade II) | *Fejervarya limnocharis* | HNFL02 | Hainan Island |  |  |  |  | MK317497 |  |  |  |  |  | Yang et al. (2022) |  |
| *Fejervarya kawamurai* (Clade II) | *Fejervarya limnocharis* | HNFL03 | Hainan Island |  |  |  |  | MK317498 |  |  |  |  |  | Yang et al. (2022) |  |
| *Fejervarya kawamurai* (Clade II) | *Fejervarya limnocharis* | HNLY01 | Henan, China |  |  |  |  | MK317507 |  |  |  |  |  | Yang et al. (2022) |  |
| *Fejervarya kawamurai* (Clade II) | *Fejervarya limnocharis* | HNXY05 | Henan, China |  |  |  |  | MK317520 |  |  |  |  |  | Yang et al. (2022) |  |
| *Fejervarya kawamurai* (Clade II) | *Fejervarya limnocharis* | SHLC15 | Shanghai,China |  |  |  |  | MK317538 |  |  |  |  |  | Yang et al. (2022) |  |
| *Fejervarya kawamurai* (Clade II) | *Fejervarya limnocharis* | ZJDQ05 | Zhejiang,China |  |  |  |  |  |  | MK317549 |  |  |  | Yang et al. (2022) |  |
| *Fejervarya kawamurai* (Clade II) | *Fejervarya limnocharis* | ZJDQ09 | Zhejiang,China |  |  |  |  |  |  | MK317550 |  |  |  | Yang et al. (2022) |  |
| *Fejervarya kawamurai* (Clade II) | *Fejervarya limnocharis* | ZJJX04 | Zhejiang,China | 30.46 | 120.45 |  |  |  |  | MK317552 |  |  |  | Yang et al. (2022) |  |
| *Fejervarya kawamurai* (Clade II) | *Fejervarya limnocharis* | ZJJX10 | Zhejiang,China | 30.46 | 120.45 |  |  | MK317553 |  | MK317553 |  |  |  | Yang et al. (2022) |  |
| *Fejervarya kawamurai* (Clade II) | *Fejervarya limnocharis* | ZJLX05 | Zhejiang,China | 29.3642 | 119.8111 |  |  | MK317566 |  | MK317566 |  |  |  | Yang et al. (2022) |  |
| *Fejervarya kawamurai* (Clade II) | *Fejervarya limnocharis* | ZJLX06 | Zhejiang,China | 29.3642 | 119.8111 |  |  |  |  | MK317567 |  |  |  | Yang et al. (2022) |  |
| *Fejervarya kawamurai* (Clade II) | *Fejervarya limnocharis* | ZJLX08 | Zhejiang,China | 29.3642 | 119.8111 |  |  |  |  | MK317568 |  |  |  | Yang et al. (2022) |  |
| *Fejervarya kawamurai* (Clade II) | *Fejervarya limnocharis* | ZJLX09 | Zhejiang,China | 29.3642 | 119.8111 |  |  |  |  | MK317569 |  |  |  | Yang et al. (2022) |  |
| *Fejervarya kawamurai* (Clade II) | *Fejervarya limnocharis* | ZJLX10 | Zhejiang,China | 29.3642 | 119.8111 |  |  |  |  | MK317570 |  |  |  | Yang et al. (2022) |  |
| *Fejervarya kawamurai* (Clade II) | *Fejervarya limnocharis* | ZJLX12 | Zhejiang,China | 29.3642 | 119.8111 |  |  |  |  | MK317571 |  |  |  | Yang et al. (2022) |  |
| *Fejervarya kawamurai* (Clade II) | *Fejervarya limnocharis* | ZJLX14 | Zhejiang,China | 29.3642 | 119.8111 |  |  |  |  | MK317572 |  |  |  | Yang et al. (2022) |  |
| *Fejervarya kawamurai* (Clade II) | *Fejervarya limnocharis* | ZJLX15 | Zhejiang,China | 29.3642 | 119.8111 |  |  |  |  | MK317573 |  |  |  | Yang et al. (2022) |  |
| *Fejervarya kawamurai* (Clade II) | *Fejervarya limnocharis* | ZJNB04 | Zhejiang,China | 29.8999 | 121.8719 |  |  | MK316688 |  | MK317577 |  |  |  | Yang et al. (2022) |  |
| *Fejervarya kawamurai* (Clade II) | *Fejervarya limnocharis* | ZJPH02 | Zhejiang,China | 30.6396 | 121.0488 |  |  | MK317586 |  |  |  | MK317309 |  | Yang et al. (2022) |  |
| *Fejervarya kawamurai* (Clade II) | *Fejervarya limnocharis* | ZJPH03 | Zhejiang,China | 30.6396 | 121.0488 |  |  |  |  | MK317587 |  |  |  | Yang et al. (2022) |  |
| *Fejervarya kawamurai* (Clade II) | *Fejervarya limnocharis* | ZJPH04 | Zhejiang,China | 30.6396 | 121.0488 |  |  |  |  | MK317588 |  |  |  | Yang et al. (2022) |  |
| *Fejervarya kawamurai* (Clade II) | *Fejervarya limnocharis* | ZJPH05 | Zhejiang,China | 30.6396 | 121.0488 |  |  |  |  | MK317589 |  |  |  | Yang et al. (2022) |  |
| *Fejervarya kawamurai* (Clade II) | *Fejervarya limnocharis* | ZJPJ01 | Zhejiang,China | 29.4847 | 120.0118 |  |  | MK317593 |  |  |  | MK317310 |  | Yang et al. (2022) |  |
| *Fejervarya kawamurai* (Clade II) | *Fejervarya limnocharis* | ZJPJ02 | Zhejiang,China | 29.4847 | 120.0118 |  |  | MK317594 |  |  |  | MK317311 |  | Yang et al. (2022) |  |
| *Fejervarya kawamurai* (Clade II) | *Fejervarya limnocharis* | ZJPJ03 | Zhejiang,China | 29.4847 | 120.0118 |  |  | MK317595 |  |  |  | MK317312 |  | Yang et al. (2022) |  |
| *Fejervarya kawamurai* (Clade II) | *Fejervarya limnocharis* | ZJPJ04 | Zhejiang,China | 29.4847 | 120.0118 |  |  | MK317596 |  |  |  | MK317313 |  | Yang et al. (2022) |  |
| *Fejervarya kawamurai* (Clade II) | *Fejervarya limnocharis* | ZJPJ05 | Zhejiang,China | 29.4847 | 120.0118 |  |  | MK317597 |  |  |  | MK317314 |  | Yang et al. (2022) |  |
| *Fejervarya kawamurai* (Clade II) | *Fejervarya limnocharis* | ZJPJ09 | Zhejiang,China | 29.4847 | 120.0118 |  |  |  |  | MK317598 |  |  |  | Yang et al. (2022) |  |
| *Fejervarya kawamurai* (Clade II) | *Fejervarya limnocharis* | ZJPJ11 | Zhejiang,China | 29.4847 | 120.0118 |  |  | MK317599 |  |  |  | MK317315 |  | Yang et al. (2022) |  |
| *Fejervarya kawamurai* (Clade II) | *Fejervarya limnocharis* | ZJPJ15 | Zhejiang,China | 29.4847 | 120.0118 |  |  |  |  | MK317601 |  |  |  | Yang et al. (2022) |  |
| *Fejervarya kawamurai* (Clade II) | *Fejervarya limnocharis* | ZSHX06 | Huangxing, Zhoushan, China | 30.1989 | 122.6412 |  |  | MK317644 |  |  |  | MK317327 |  | Yang et al. (2022) |  |
| *Fejervarya kawamurai* (Clade II) | *Fejervarya limnocharis* | ZSKZ08 | Kuzhu,Zhoushan, China | 29.9928 | 122.2436 |  |  | MK317661 |  |  |  | MK317330 |  | Yang et al. (2022) |  |
| *Fejervarya kawamurai* (Clade I) | *Fejervarya limnocharis* | GZBY01 | Guangzhou,China | 23.2857 | 113.3058 |  |  | MK317476 |  |  |  | MK317232 |  | Yang et al. (2022) |  |
| *Fejervarya kawamurai* (Clade I) | *Fejervarya limnocharis* | GZBY02 | Guangzhou,China | 23.2857 | 113.3058 |  |  | MK317477 |  |  |  | MK317233 |  | Yang et al. (2022) |  |
| *Fejervarya kawamurai* (Clade I) | *Fejervarya limnocharis* | GZBY04 | Guangzhou,China | 23.2857 | 113.3058 |  |  | MK317479 |  |  |  | MK317235 |  | Yang et al. (2022) |  |
| *Fejervarya kawamurai* (Clade I) | *Fejervarya limnocharis* | GZBY05 | Guangzhou,China | 23.2857 | 113.3058 |  |  | MK317480 |  |  |  | MK317236 |  | Yang et al. (2022) |  |
| *Fejervarya kawamurai* (Clade I) | *Fejervarya limnocharis* | HNXY05 | Shanghai,China | 32.1469 | 114.091 |  |  | MK317520 |  |  |  | MK317274 |  | Yang et al. (2022) |  |
| *Fejervarya kawamurai* (Clade I) | *Fejervarya limnocharis* | ZJPH12 | Zhejiang,China | 30.6396 | 121.0488 |  |  |  |  | MK317591 |  |  |  | Yang et al. (2022) |  |
| *Fejervarya kawamurai* (Clade I) | *Fejervarya limnocharis* | ZSBQ11 | Baiquan, Zhoushan, China | 30.0567 | 122.1578 |  |  | MK317604 |  |  |  | MK317316 |  | Yang et al. (2022) |  |
| *Fejervarya kawamurai* (Clade I) | *Fejervarya limnocharis* | ZSDD03 | Daidong, Zhoushan, China | 30.2902 | 122.1947 |  |  | MK317606 |  |  |  | MK317317 |  | Yang et al. (2022) |  |
| *Fejervarya kawamurai* (Clade I) | *Fejervarya limnocharis* | ZSDF01 | Dongfu, Zhoushan, China | 30.1442 | 122.7575 |  |  |  |  | MK317611 |  |  |  | Yang et al. (2022) |  |
| *Fejervarya kawamurai* (Clade I) | *Fejervarya limnocharis* | ZSDF03 | Dongfu, Zhoushan, China | 30.1442 | 122.7575 |  |  | MK317613 |  |  |  | MK317318 |  | Yang et al. (2022) |  |
| *Fejervarya kawamurai* (Clade I) | *Fejervarya limnocharis* | ZSDH02 | Donghai, Zhoushan, China | 30.7083 | 122.4992 |  |  | MK317614 |  |  |  | MK317319 |  | Yang et al. (2022) |  |
| *Fejervarya kawamurai* (Clade I) | *Fejervarya limnocharis* | ZSDJ12 | Daijia, Zhoushan, China | 30.0198 | 122.0332 |  |  | MK317620 |  |  |  | MK317320 |  | Yang et al. (2022) |  |
| *Fejervarya kawamurai* (Clade I) | *Fejervarya limnocharis* | ZSGJ01 | Zhejiang, China | 29.8833 | 121.9823 |  |  | MK317627 |  |  |  | MK317322 |  | Yang et al. (2022) |  |
| *Fejervarya kawamurai* (Clade I) | *Fejervarya limnocharis* | ZSGL04 | Ganlan,Zhoushan, China | 30.1121 | 122.1305 |  |  | MK317633 |  |  |  | MK317323 |  | Yang et al. (2022) |  |
| *Fejervarya kawamurai* (Clade I) | *Fejervarya limnocharis* | ZSGQ06 | Gouqi, Zhoushan, China | 30.7162 | 122.7724 |  |  | MK317635 |  |  |  | MK317324 |  | Yang et al. (2022) |  |
| *Fejervarya kawamurai* (Clade I) | *Fejervarya limnocharis* | ZSHL03 | Huanglong, Zhoushan, China | 30.728 | 122.8146 |  |  | MK316704 |  |  |  | MK317325 |  | Yang et al. (2022) |  |
| *Fejervarya kawamurai* (Clade I) | *Fejervarya limnocharis* | ZSHN01 | Huaniao, Zhoushan, China | 30.8474 | 122.6917 |  |  | MK317638 |  |  |  | MK317326 |  | Yang et al. (2022) |  |
| *Fejervarya kawamurai* (Clade I) | *Fejervarya limnocharis* | ZSHX06 | Huangxing, Zhoushan, China | 30.1989 | 122.6412 |  |  | MK317644 |  |  |  | MK317327 |  | Yang et al. (2022) |  |
| *Fejervarya kawamurai* (Clade I) | *Fejervarya limnocharis* | ZSJH01 | Jihu, Zhoushan, China | 30.7182 | 122.4624 |  |  | MK317646 |  |  |  | MK317328 |  | Yang et al. (2022) |  |
| *Fejervarya kawamurai* (Clade I) | *Fejervarya limnocharis* | ZSKZ08 | Kuzhu, Zhoushan, China | 29.9928 | 122.2436 |  |  | MK317661 |  |  |  | MK317330 |  | Yang et al. (2022) |  |
| *Fejervarya kawamurai* (Clade I) | *Fejervarya limnocharis* | ZSMZH01 | Miaozihu, Zhoushan, China | 30.1976 | 122.6792 |  |  | MK317666 |  |  |  | MK317332 |  | Yang et al. (2022) |  |
| *Fejervarya kawamurai* (Clade I) | *Fejervarya limnocharis* | ZSMZH02 | Miaozihu, Zhoushan, China | 30.1976 | 122.6792 |  |  | MK317667 |  |  |  | MK317333 |  | Yang et al. (2022) |  |
| *Fejervarya kawamurai* (Clade I) | *Fejervarya limnocharis* | ZSMZH03 | Miaozihu, Zhoushan, China | 30.1976 | 122.6792 |  |  | MK317668 |  |  |  | MK317334 |  | Yang et al. (2022) |  |
| *Fejervarya kawamurai* (Clade I) | *Fejervarya limnocharis* | ZSNP03 | Nanpu, Zhoushan, China | 30.2667 | 122.1385 |  |  | MK317679 |  |  |  | MK317335 |  | Yang et al. (2022) |  |
| *Fejervarya kawamurai* (Clade I) | *Fejervarya limnocharis* | ZSQB04 | Qingbang, Zhoushan, China | 30.1995 | 122.7069 |  |  |  |  | MK317687 |  |  |  | Yang et al. (2022) |  |
| *Fejervarya kawamurai* (Clade I) | *Fejervarya limnocharis* | ZSQS02 | Qushan, Zhoushan, China | 30.4371 | 122.3516 |  |  | MK317690 |  |  |  | MK317338 |  | Yang et al. (2022) |  |
| *Fejervarya kawamurai* (Clade I) | *Fejervarya limnocharis* | ZSSN05 | Shengshan, Zhoushan, China | 30.7239 | 122.811 |  |  | MK317701 |  |  |  | MK317340 |  | Yang et al. (2022) |  |
| *Fejervarya kawamurai* (Clade I) | *Fejervarya limnocharis* | ZSSN09 | Sannong, Zhoushan, China | 30.4454 | 122.2938 |  |  | MK317698 |  |  |  | MK317339 |  | Yang et al. (2022) |  |
| *Fejervarya kawamurai* (Clade I) | *Fejervarya limnocharis* | ZSSS05 | Sannong, Zhoushan, China | 30.4454 | 122.2938 |  |  | MK317701 |  |  |  | MK317340 |  | Yang et al. (2022) |  |
| *Fejervarya kawamurai* (Clade I) | *Fejervarya limnocharis* | ZSTT06 | Tiantu,Zhoushan, China | 30.4386 | 122.41 |  |  |  |  |  |  | MK317341 |  | Yang et al. (2022) |  |
| *Fejervarya kawamurai* (Clade I) | *Fejervarya limnocharis* | ZSXYS01 | Xiaoyangshan,Zhoushan, China | 30.6411 | 122.0611 |  |  | MK317706 |  |  |  | MK317342 |  | Yang et al. (2022) |  |
| *Fejervarya kawamurai* (Clade I) | *Fejervarya limnocharis* | ZSYD09 | Yandun,Zhoushan, China | 30.136 | 122.0198 |  |  | MK317714 |  |  |  | MK317343 |  | Yang et al. (2022) |  |
| *Fejervarya kawamurai* (Clade I) | *Fejervarya limnocharis* | ZSZM14 | Zhanmao,Zhoushan, China | 30.0476 | 122.2564 |  |  | MK317716 |  |  |  | MK317345 |  | Yang et al. (2022) |  |
| *Fejervarya kawamurai* (Clade I) | *Fejervarya limnocharis* | Partial genome | Zunyi city, Guizhou, China | 27.7083 | 106.9200 | MH087466 | MH087466 | MH087466 | MH087466 | MH087466 |  |  |  | Cheng et al. (2014) |  |
| *Fejervarya kawamurai* (Clade II) | *Fejervarya limnocharis* | ZJLX05 | Zhejiang, China | 29.3642 | 119.8111 |  |  | MK317566 |  |  | MK317793 | MK317302 | MK317419 | Yang et al. (2022) |  |
| *Fejervarya kawamurai* (Clade II) | *Fejervarya limnocharis* | ZJLX06 | Zhejiang, China | 29.3642 | 119.8111 |  |  |  |  | MK317567 |  |  |  | Yang et al. (2022) |  |
| *Fejervarya kawamurai* (Clade II) | *Fejervarya limnocharis* | ZJLX08 | Zhejiang, China | 29.3642 | 119.8111 |  |  | MK317568 |  |  | MK317794 | MK317303 | MK317420 | Yang et al. (2022) |  |
| *Fejervarya kawamurai* (Clade II) | *Fejervarya limnocharis* | ZJLX09 | Zhejiang, China | 29.3642 | 119.8111 |  |  | MK317569 |  |  | MK317795 | MK317304 | MK317421 | Yang et al. (2022) |  |
| *Fejervarya kawamurai* (Clade II) | *Fejervarya limnocharis* | ZJLX12 | Zhejiang, China | 29.3642 | 119.8111 |  |  |  |  | MK317571 |  |  |  | Yang et al. (2022) |  |
| *Fejervarya kawamurai* (Clade II) | *Fejervarya limnocharis* | ZJLX14 | Zhejiang, China | 29.3642 | 119.8111 |  |  |  |  | MK317572 |  |  |  | Yang et al. (2022) |  |
| *Fejervarya kawamurai* (Clade II) | *Fejervarya limnocharis* | ZJLX15 | Zhejiang, China | 29.3642 | 119.8111 |  |  |  |  | MK317573 |  |  |  | Yang et al. (2022) |  |
| *Fejervarya kawamurai* (Clade II) | *Fejervarya limnocharis* | ZJPH02 | Zhejiang, China | 30.6396 | 121.0488 |  |  | MK317586 |  |  | MK317800 | MK317309 | MK317423 | Yang et al. (2022) |  |
| *Fejervarya kawamurai* (Clade II) | *Fejervarya limnocharis* | ZJPH03 | Zhejiang, China | 30.6396 | 121.0488 |  |  |  |  | MK317587 |  |  |  | Yang et al. (2022) |  |
| *Fejervarya kawamurai* (Clade II) | *Fejervarya limnocharis* | ZJPH04 | Zhejiang, China | 30.6396 | 121.0488 |  |  |  |  | MK317588 |  |  |  | Yang et al. (2022) |  |
| *Fejervarya kawamurai* (Clade II) | *Fejervarya limnocharis* | ZJPH05 | Zhejiang, China | 30.6396 | 121.0488 |  |  |  |  | MK317589 |  |  |  | Yang et al. (2022) |  |
| *Fejervarya sakishimensis* | *Fejervarya sakishimensis* | Jp1 | Iriomote Island, Japan |  |  | AB488863 | AB488886 |  |  |  | AB489004 |  | AB489025 | Kotaki et al. (2010) |  |
| *Fejervarya limnocharis* (Clade II) | *Fejervarya limnocharis* | CAS 235484 | Krung Thep Mahanakhon (Bangkok), Thailand |  |  | MK621345 | MK621395 |  |  |  |  |  |  | [10] |  |
| *Fejervarya limnocharis* (Clade II) | *Fejervarya limnocharis* | CAS 215116 | Shanjiang village, just S of Liuku, Yunnan, China |  |  | MK621338 | MK621388 |  |  |  |  |  |  | Köhler et al., (2019) |  |
| *Fejervarya limnocharis* (Clade II) | *Fejervarya limnocharis* | CAS 228131 | Shanjiang village, just S of Liuku, Yunnan, China |  |  | MK621343 | MK621393 |  |  |  |  |  |  | Köhler et al., (2019) |  |
| *Fejervarya limnocharis* (Clade II) | *Fejervarya limnocharis* | CAS 228129 | Liuku bridge, Yunnan, China |  |  | MK621342 | MK621392 |  |  |  |  |  |  | Köhler et al., (2019) |  |
| *Fejervarya limnocharis* (Clade II) | *Fejervarya limnocharis* | CAS 230367 | Myitkyina Township, Pidaung Wildlife Sanctuary, near Ma-Li-Hka Village, Myanmar |  |  | MK958590 | MK958571 |  |  |  |  |  |  | Köhler et al., (2019) |  |
| *Fejervarya limnocharis* (Clade II) | *Fejervarya limnocharis* | CAS 234814 | Chin: Kanpatlat Township, in Kanpatlat town, Myanmar |  |  | MK958591 | MK958572 |  |  |  |  |  |  | Köhler et al., (2019) |  |
| *Fejervarya limnocharis* (Clade II) | *Fejervarya limnocharis* | CAS 234815 | Chin, Kanpatlat Township, in Kanpatlat town, Myanmar |  |  | MK958573 |  |  |  |  |  |  |  | Köhler et al., (2019) |  |
| *Fejervarya limnocharis* (Clade II) | *Fejervarya limnocharis* | CAS 235276 | Kanpatlat Township, Let Mon Village, Myanmar |  |  | MK621394 | MK621344 |  |  |  |  |  |  | Köhler et al., (2019) |  |
| *Fejervarya limnocharis* (Clade II) | *Fejervarya limnocharis* | SMF 104127 | Mae Hong Son, Pangmapha, Ban Soppong, Thailand |  |  | MK621398 | MK621348 |  |  |  |  |  |  | Kotaki et al. (2010) |  |
| *Fejervarya limnocharis* (Clade II) | *Fejervarya limnocharis* | Thai-1 | Ranong, Thailand |  |  |  |  |  |  |  | AB277351 | AB277333 |  | Kotaki et al. (2010) |  |
| *Fejervarya limnocharis* (Clade II) | *Fejervarya limnocharis* | Thai-2 | Tha Ton, Thailand |  |  |  |  |  |  |  |  | AB277334 |  | Kotaki et al. (2010) |  |
| *Fejervarya limnocharis* (Clade II) | *Fejervarya limnocharis* | Thai-3 | Mae Hong Son, Thailand |  |  |  |  |  |  |  |  | AB277335 |  | Kotaki et al. (2010) |  |
| *Fejervarya limnocharis* (Clade II) | *Fejervarya limnocharis* | Thai-4 | Nakhon Si Thammarat, Thailand |  |  |  |  |  |  |  |  | AB277336 |  | Kotaki et al. (2010) |  |
| *Fejervarya limnocharis* (Clade II) | *Fejervarya limnocharis* | SMF 104128 | Mae Hong Son, Pangmapha, Ban Soppong, Thailand |  |  | MK621399 | MK621349 |  |  |  |  |  |  | Kotaki et al. (2010) |  |
| *Fejervarya limnocharis* (Clade II) | *Fejervarya limnocharis* | SMF 104131 | Pangmapha, near Ban Nam Rin, Thailand |  |  | MK621349 | MK621399 |  |  |  |  |  |  | Koehler et al. (2019) |  |
| *Fejervarya limnocharis* (Clade II) | *Fejervarya limnocharis* | SMF 105119 | Ridge north of viewpoint between Pangmapha and Pai, Thailand |  |  | MK958574 |  |  |  |  |  |  |  | Koehler et al. (2019) |  |
| *Fejervarya limnocharis* (Clade II) | *Fejervarya limnocharis* | SMF 105120 | Ridge north of viewpoint between Pangmapha and Pai, Thailand |  |  | MK958575 | MK958592 |  |  |  |  |  |  | Koehler et al. (2019) |  |
| *Fejervarya limnocharis* (Clade II) | *Fejervarya limnocharis* | SMF 105121 | Pangmapha district, at NamLang River, Thailand |  |  | MK958576 |  |  |  |  |  |  |  | Koehler et al. (2019) |  |
| *Fejervarya limnocharis* (Clade II) | *Fejervarya limnocharis* | TZ25 | North Vietnam |  |  |  | AF285211 |  |  |  |  |  |  | Ziegler et al. (2000) |  |
| *Fejervarya limnocharis* (Clade II) | *Fejervarya limnocharis* | TZ98105 | North Vietnam |  |  |  | AF285212 |  |  |  |  |  |  | Ziegler et al. (2000) |  |
| *Fejervarya limnocharis* (Clade II) | *Fejervarya limnocharis* | 1999.5721 | Lao Cai, Sapa, Vietnam | 22° 19' N | 103° 47' E |  |  |  | KR087652 |  |  |  |  | Grosjean et al. (2015) |  |
| *Fejervarya limnocharis* (Clade II) | *Fejervarya limnocharis* | 2004.0208 | Phongsali, Buon Tai, Laos | 21° 23' 00" N | 101° 58' 00" E |  | KR827751 |  | KR087660 |  |  |  |  | Grosjean et al. (2015) |  |
| *Fejervarya limnocharis* (Clade II) | *Fejervarya limnocharis* | 2004.0309 | Phongsali, Nathen, Laos | 21° 24' N | 101° 58' E |  | KR827753 |  | KR087662 |  |  |  |  | Grosjean et al. (2015) |  |
| *Fejervarya limnocharis* (Clade II) | *Fejervarya limnocharis* | 2006.2411 | Louangphrabang, Ban Sop Khao, Laos |  |  |  | KR827749 |  | KR087658 |  |  |  |  | Grosjean et al. (2015) |  |
| *Fejervarya limnocharis* (Clade II) | *Fejervarya limnocharis* | K3037 | Chiang Mai, Doi Chiang Dao, Thailand |  |  |  |  |  | KR087653 |  |  |  |  | Grosjean et al. (2015) |  |
| *Fejervarya limnocharis* (Clade II) | *Fejervarya limnocharis* | K3085 | Louangphrabang, Ban Sop Khao, Laos |  |  |  | KR827748 |  | KR087657 |  |  |  |  | Grosjean et al. (2015) |  |
| *Fejervarya kawamurai* (Clade II) | *Fejervarya multistriata* | FM_HN_01 | Northern China |  |  |  |  |  | KY385701 |  |  |  |  | Wang et al. (2016), direct submission |  |
| *Fejervarya kawamurai* (Clade II) | *Fejervarya multistriata* | FM_HN_02 | Northern China |  |  |  |  |  | KY385702 |  |  |  |  | Wang et al. (2016), direct submission |  |
| *Fejervarya kawamurai* (Clade II) | *Fejervarya multistriata* | FM_HN_03 | Northern China |  |  |  |  |  | KY385703 |  |  |  |  | Wang et al. (2016), direct submission |  |
| *Fejervarya kawamurai* (Clade II) | *Fejervarya multistriata* | FM_HN_04 | Northern China |  |  |  |  |  | KY385704 |  |  |  |  | Wang et al. (2016), direct submission |  |
| *Fejervarya kawamurai* (Clade II) | *Fejervarya multistriata* | FM_HN_05 | Northern China |  |  |  |  |  | KY385705 |  |  |  |  | Wang et al. (2016), direct submission |  |
| *Fejervarya kawamurai* (Clade II) | *Fejervarya multistriata* | FM_HN_06 | Northern China |  |  |  |  |  | KY385706 |  |  |  |  | Wang et al. (2016), direct submission |  |
| *Fejervarya kawamurai* (Clade II) | *Fejervarya multistriata* | FM_HN_07 | Northern China |  |  |  |  |  | KY385707 |  |  |  |  | Wang et al. (2016), direct submission |  |
| *Fejervarya kawamurai* (Clade II) | *Fejervarya multistriata* | FM_HN_08 | Northern China |  |  |  |  |  | KY385708 |  |  |  |  | Wang et al. (2016), direct submission |  |
| *Fejervarya kawamurai* (Clade II) | *Fejervarya multistriata* | FM_HN_09 | Northern China |  |  |  |  |  | KY385709 |  |  |  |  | Wang et al. (2016), direct submission |  |
| *Fejervarya kawamurai* (Clade II) | *Fejervarya multistriata* | FM_HN_10 | Northern China |  |  |  |  |  | KY385710 |  |  |  |  | Wang et al. (2016), direct submission |  |
| *Fejervarya kawamurai* (Clade II) | *Fejervarya multistriata* | MZ44_MZ | Chongming, Shanghai, China |  |  |  |  | MT254032 |  |  |  |  |  | Ding and Chen (2021), direct submission |  |
| *Fejervarya kawamurai* (Clade II) | *Fejervarya multistriata* | ZWc_1113_BZ | Chongming, Shanghai, China |  |  |  |  | MT254033 |  |  |  |  |  | Ding and Chen (2021), direct submission |  |
| *Fejervarya kawamurai* (Clade II) | *Fejervarya multistriata* | ZWc_1121_BZ | Chongming, Shanghai, China |  |  |  |  | MT253947 |  |  |  |  |  | Ding and Chen (2021), direct submission |  |
| *Fejervarya kawamurai* (Clade II) | *Fejervarya multistriata* | ZWc_13111_MZ | Chongming, Shanghai, China |  |  |  |  | MT253947 |  |  |  |  |  | Ding and Chen (2021), direct submission |  |
| *Fejervarya kawamurai* (Clade II) | *Fejervarya multistriata* | ZWc_1317_MZ | Chongming, Shanghai, China |  |  |  |  | MT253954 |  |  |  |  |  | Ding and Chen (2021), direct submission |  |
| *Fejervarya kawamurai* (Clade II) | *Fejervarya multistriata* | ZWc_1343_MZ | Chongming, Shanghai, China |  |  |  |  | MT253949 |  |  |  |  |  | Ding and Chen (2021), direct submission |  |
| *Fejervarya kawamurai* (Clade II) | *Fejervarya multistriata* | ZWc_1345_MZ | Chongming, Shanghai, China |  |  |  |  | MT253948 |  |  |  |  |  | Ding and Chen (2021), direct submission |  |
| *Fejervarya kawamurai* (Clade II) | *Fejervarya multistriata* | ZWc_1346_MZ | Chongming, Shanghai, China |  |  |  |  | MT253953 |  |  |  |  |  | Ding and Chen (2021), direct submission |  |
| *Fejervarya kawamurai* (Clade II) | *Fejervarya multistriata* | ZWc_1516a_MT | Chongming, Shanghai, China |  |  |  |  | MT254037 |  |  |  |  |  | Ding and Chen (2021), direct submission |  |
| *Fejervarya kawamurai* (Clade II) | *Fejervarya multistriata* | ZWc_1527_MZH | Chongming, Shanghai, China |  |  |  |  | MT254046 |  |  |  |  |  | Ding and Chen (2021), direct submission |  |
| *Fejervarya kawamurai* (Clade II) | *Fejervarya multistriata* | ZWc_1611b_BBY | Chongming, Shanghai, China |  |  |  |  | MT254023 |  |  |  |  |  | Ding and Chen (2021), direct submission |  |
| *Fejervarya kawamurai* (Clade II) | *Fejervarya multistriata* | ZWc_1621_BBY | Chongming, Shanghai, China |  |  |  |  | MT254034 |  |  |  |  |  | Ding and Chen (2021), direct submission |  |
| *Fejervarya kawamurai* (Clade II) | *Fejervarya multistriata* | ZWc_1622b_BBY | Chongming, Shanghai, China |  |  |  |  | MT254031 |  |  |  |  |  | Ding and Chen (2021), direct submission |  |
| *Fejervarya kawamurai* (Clade II) | *Fejervarya multistriata* | ZWc_1643b_BBY | Chongming, Shanghai, China |  |  |  |  | MT253967 |  |  |  |  |  | Ding and Chen (2021), direct submission |  |
| *Fejervarya kawamurai* (Clade II) | *Fejervarya multistriata* | ZWc_1646b_BBY | Chongming, Shanghai, China |  |  |  |  | MT254049 |  |  |  |  |  | Ding and Chen (2021), direct submission |  |
| *Fejervarya kawamurai* (Clade II) | *Fejervarya multistriata* | ZWc_1819b_DP | Chongming, Shanghai, China |  |  |  |  | MT253946. |  |  |  |  |  | Ding and Chen (2021), direct submission |  |
| *Fejervarya kawamurai* (Clade II) | *Fejervarya multistriata* | ZWc_1826_DP | Chongming, Shanghai, China |  |  |  |  | MT254044 |  |  |  |  |  | Ding and Chen (2021), direct submission |  |
| *Fejervarya kawamurai* (Clade II) | *Fejervarya multistriata* | ZWc_BBY29_BBY | Chongming, Shanghai, China |  |  |  |  | MT254041 |  |  |  |  |  | Ding and Chen (2021), direct submission |  |
| *Fejervarya kawamurai* (Clade II) | *Fejervarya multistriata* | ZWc_BHQ07_DT | Chongming, Shanghai, China |  |  |  |  | MT254035 |  |  |  |  |  | Ding and Chen (2021), direct submission |  |
| *Fejervarya kawamurai* (Clade II) | *Fejervarya multistriata* | ZWc_BHQ12_DT | Chongming, Shanghai, China |  |  |  |  | MT254039 |  |  |  |  |  | Ding and Chen (2021), direct submission |  |
| *Fejervarya kawamurai* (Clade II) | *Fejervarya multistriata* | ZWc_BHQ14_DT | Chongming, Shanghai, China |  |  |  |  | MT254040 |  |  |  |  |  | Ding and Chen (2021), direct submission |  |
| *Fejervarya kawamurai* (Clade II) | *Fejervarya multistriata* | ZWc_BZ09_BZ | Chongming, Shanghai, China |  |  |  |  | MT254042 |  |  |  |  |  | Ding and Chen (2021), direct submission |  |
| *Fejervarya kawamurai* (Clade II) | *Fejervarya multistriata* | ZWc_BZ10_BZ | Chongming, Shanghai, China |  |  |  |  | MT254043 |  |  |  |  |  | Ding and Chen (2021), direct submission |  |
| *Fejervarya kawamurai* (Clade II) | *Fejervarya multistriata* | ZWc_BZ13_BZ | Chongming, Shanghai, China |  |  |  |  | MT254045 |  |  |  |  |  | Ding and Chen (2021), direct submission |  |
| *Fejervarya kawamurai* (Clade II) | *Fejervarya multistriata* | ZWc_BZ23_BZ | Chongming, Shanghai, China |  |  |  |  | MT254048. |  |  |  |  |  | Ding and Chen (2021), direct submission |  |
| *Fejervarya kawamurai* (Clade II) | *Fejervarya multistriata* | ZWc_DL01_DL | Chongming, Shanghai, China |  |  |  |  | MT254011 |  |  |  |  |  | Ding and Chen (2021), direct submission |  |
| *Fejervarya kawamurai* (Clade II) | *Fejervarya multistriata* | ZWc_DL02_DL | Chongming, Shanghai, China |  |  |  |  | MT254047 |  |  |  |  |  | Ding and Chen (2021), direct submission |  |
| *Fejervarya kawamurai* (Clade II) | *Fejervarya multistriata* | ZWc_DL03_DL | Chongming, Shanghai, China |  |  |  |  | MT254012 |  |  |  |  |  | Ding and Chen (2021), direct submission |  |
| *Fejervarya kawamurai* (Clade II) | *Fejervarya multistriata* | ZWc_DL11_DL | Chongming, Shanghai, China |  |  |  |  | MT254015 |  |  |  |  |  | Ding and Chen (2021), direct submission |  |
| *Fejervarya kawamurai* (Clade II) | *Fejervarya multistriata* | ZWc_DL17_DL | Chongming, Shanghai, China |  |  |  |  | MT254018 |  |  |  |  |  | Ding and Chen (2021), direct submission |  |
| *Fejervarya kawamurai* (Clade II) | *Fejervarya multistriata* | ZWc_DL18_DL | Chongming, Shanghai, China |  |  |  |  | MT254026 |  |  |  |  |  | Ding and Chen (2021), direct submission |  |
| *Fejervarya kawamurai* (Clade II) | *Fejervarya multistriata* | ZWc_DT12_DT | Chongming, Shanghai, China |  |  |  |  | MT253963 |  |  |  |  |  | Ding and Chen (2021), direct submission |  |
| *Fejervarya kawamurai* (Clade II) | *Fejervarya multistriata* | ZWc_DT2_DT | Chongming, Shanghai, China |  |  |  |  | MT253961 |  |  |  |  |  | Ding and Chen (2021), direct submission |  |
| *Fejervarya kawamurai* (Clade II) | *Fejervarya multistriata* | ZWc_DT5_DT | Chongming, Shanghai, China |  |  |  |  | MT254027 |  |  |  |  |  | Ding and Chen (2021), direct submission |  |
| *Fejervarya kawamurai* (Clade II) | *Fejervarya multistriata* | ZWc_DT8_DT | Chongming, Shanghai, China |  |  |  |  | MT253962 |  |  |  |  |  | Ding and Chen (2021), direct submission |  |
| *Fejervarya kawamurai* (Clade II) | *Fejervarya multistriata* | ZWc_HS14_HS | Chongming, Shanghai, China |  |  |  |  | MT254028 |  |  |  |  |  | Ding and Chen (2021), direct submission |  |
| *Fejervarya kawamurai* (Clade II) | *Fejervarya multistriata* | ZWc_HS16_HS | Chongming, Shanghai, China |  |  |  |  | MT254029 |  |  |  |  |  | Ding and Chen (2021), direct submission |  |
| *Fejervarya kawamurai* (Clade II) | *Fejervarya multistriata* | ZWc_HS17_HS | Chongming, Shanghai, China |  |  |  |  | MT254030 |  |  |  |  |  | Ding and Chen (2021), direct submission |  |
| *Fejervarya kawamurai* (Clade II) | *Fejervarya multistriata* | ZWc_MT02_MT | Chongming, Shanghai, China |  |  |  |  | MT253955 |  |  |  |  |  | Ding and Chen (2021), direct submission |  |
| *Fejervarya kawamurai* (Clade II) | *Fejervarya multistriata* | ZWc_MT09_MT | Chongming, Shanghai, China |  |  |  |  | MT253956 |  |  |  |  |  | Ding and Chen (2021), direct submission |  |
| *Fejervarya kawamurai* (Clade II) | *Fejervarya multistriata* | ZWc_MT10_MT | Chongming, Shanghai, China |  |  |  |  | MT253957 |  |  |  |  |  | Ding and Chen (2021), direct submission |  |
| *Fejervarya kawamurai* (Clade II) | *Fejervarya multistriata* | ZWc_MT13_MT | Chongming, Shanghai, China |  |  |  |  | MT253959 |  |  |  |  |  | Ding and Chen (2021), direct submission |  |
| *Fejervarya kawamurai* (Clade II) | *Fejervarya multistriata* | ZWc_MT16_MT | Chongming, Shanghai, China |  |  |  |  | MT253959 |  |  |  |  |  | Ding and Chen (2021), direct submission |  |
| *Fejervarya kawamurai* (Clade II) | *Fejervarya multistriata* | ZWc_MT18_MT | Chongming, Shanghai, China |  |  |  |  | MT253960 |  |  |  |  |  | Ding and Chen (2021), direct submission |  |
| *Fejervarya kawamurai* (Clade II) | *Fejervarya multistriata* | ZWc_MZ01_MZ | Chongming, Shanghai, China |  |  |  |  | MT253965 |  |  |  |  |  | Ding and Chen (2021), direct submission |  |
| *Fejervarya kawamurai* (Clade II) | *Fejervarya multistriata* | ZWc_MZ04_MZ | Chongming, Shanghai, China |  |  |  |  | MT253965 |  |  |  |  |  | Ding and Chen (2021), direct submission |  |
| *Fejervarya kawamurai* (Clade II) | *Fejervarya multistriata* | ZWc_MZ06_MZ | Chongming, Shanghai, China |  |  |  |  | MT253966 |  |  |  |  |  | Ding and Chen (2021), direct submission |  |
| *Fejervarya kawamurai* (Clade II) | *Fejervarya multistriata* | ZWc_MZ09_MZ | Chongming, Shanghai, China |  |  |  |  | MT253968 |  |  |  |  |  | Ding and Chen (2021), direct submission |  |
| *Fejervarya kawamurai* (Clade II) | *Fejervarya multistriata* | ZWc_MZ10_MZ | Chongming, Shanghai, China |  |  |  |  | MT253969 |  |  |  |  |  | Ding and Chen (2021), direct submission |  |
| *Fejervarya kawamurai* (Clade II) | *Fejervarya multistriata* | _ZWc_MZ11_MZ | Chongming, Shanghai, China |  |  |  |  | MT253970 |  |  |  |  |  | Ding and Chen (2021), direct submission |  |
| *Fejervarya kawamurai* (Clade II) | *Fejervarya multistriata* | ZWc_MZ13_MZ | Chongming, Shanghai, China |  |  |  |  | MT253971 |  |  |  |  |  | Ding and Chen (2021), direct submission |  |
| *Fejervarya kawamurai* (Clade II) | *Fejervarya multistriata* | ZWc_MZ14_MZ | Chongming, Shanghai, China |  |  |  |  | MT253972 |  |  |  |  |  | Ding and Chen (2021), direct submission |  |
| *Fejervarya kawamurai* (Clade II) | *Fejervarya multistriata* | ZWc_MZ15_MZ | Chongming, Shanghai, China |  |  |  |  | MT253973 |  |  |  |  |  | Ding and Chen (2021), direct submission |  |
| *Fejervarya kawamurai* (Clade II) | *Fejervarya multistriata* | ZWc_MZ16_MZ | Chongming, Shanghai, China |  |  |  |  | MT253974 |  |  |  |  |  | Ding and Chen (2021), direct submission |  |
| *Fejervarya kawamurai* (Clade II) | *Fejervarya multistriata* | ZWc_MZ21_MZ | Chongming, Shanghai, China |  |  |  |  | MT253974 |  |  |  |  |  | Ding and Chen (2021), direct submission |  |
| *Fejervarya kawamurai* (Clade II) | *Fejervarya multistriata* | ZWc_MZ22_MZ | Chongming, Shanghai, China |  |  |  |  | MT253975 |  |  |  |  |  | Ding and Chen (2021), direct submission |  |
| *Fejervarya kawamurai* (Clade II) | *Fejervarya multistriata* | ZWc_MZ23_MZ | Chongming, Shanghai, China |  |  |  |  | MT253976 |  |  |  |  |  | Ding and Chen (2021), direct submission |  |
| *Fejervarya kawamurai* (Clade II) | *Fejervarya multistriata* | ZWc_MZ26_MZ | Chongming, Shanghai, China |  |  |  |  | MT253977 |  |  |  |  |  | Ding and Chen (2021), direct submission |  |
| *Fejervarya kawamurai* (Clade II) | *Fejervarya multistriata* | ZWc_MZ30_MZ | Chongming, Shanghai, China |  |  |  |  | MT254036 |  |  |  |  |  | Ding and Chen (2021), direct submission |  |
| *Fejervarya kawamurai* (Clade II) | *Fejervarya multistriata* | ZWc_MZH15_MZH | Chongming, Shanghai, China |  |  |  |  | MT254038 |  |  |  |  |  | Ding and Chen (2021), direct submission |  |
| *Fejervarya kawamurai* (Clade II) | *Fejervarya multistriata* | ZWc_QJX26_QXJ | Chongming, Shanghai, China |  |  |  |  | MT254019 |  |  |  |  |  | Ding and Chen (2021), direct submission |  |
| *Fejervarya kawamurai* (Clade II) | *Fejervarya multistriata* | ZWc_QJX31_QXJ | Chongming, Shanghai, China |  |  |  |  | MT254020 |  |  |  |  |  | Ding and Chen (2021), direct submission |  |
| *Fejervarya kawamurai* (Clade II) | *Fejervarya multistriata* | ZWc_QXJ37_QXJ | Chongming, Shanghai, China |  |  |  |  | MT254022 |  |  |  |  |  | Ding and Chen (2021), direct submission |  |
| *Fejervarya kawamurai* (Clade II) | *Fejervarya multistriata* | ZWc_QXJ46_QXJ | Chongming, Shanghai, China |  |  |  |  | MT254024 |  |  |  |  |  | Ding and Chen (2021), direct submission |  |
| *Fejervarya kawamurai* (Clade II) | *Fejervarya multistriata* | ZWd1102_BZ | Chongming, Shanghai, China |  |  |  |  |  |  | MT235073 |  |  |  | Ding and Chen (2021), direct submission |  |
| *Fejervarya kawamurai* (Clade II) | *Fejervarya multistriata* | ZWd1103_BZ | Chongming, Shanghai, China |  |  |  |  |  |  | MT235074 |  |  |  | Ding and Chen (2021), direct submission |  |
| *Fejervarya kawamurai* (Clade II) | *Fejervarya multistriata* | ZWd1117_BZ | Chongming, Shanghai, China |  |  |  |  |  |  | MT235075 |  |  |  | Ding and Chen (2021), direct submission |  |
| *Fejervarya kawamurai* (Clade II) | *Fejervarya multistriata* | ZWd1135_BZ | Chongming, Shanghai, China |  |  |  |  |  |  | MT235077 |  |  |  | Ding and Chen (2021), direct submission |  |
| *Fejervarya kawamurai* (Clade II) | *Fejervarya multistriata* | ZWd1211_BZ | Chongming, Shanghai, China |  |  |  |  |  |  | MT235078 |  |  |  | Ding and Chen (2021), direct submission |  |
| *Fejervarya kawamurai* (Clade II) | *Fejervarya multistriata* | ZWd1215_DP | Chongming, Shanghai, China |  |  |  |  |  |  | MT235101 |  |  |  | Ding and Chen (2021), direct submission |  |
| *Fejervarya kawamurai* (Clade II) | *Fejervarya multistriata* | ZWd1231_BZ | Chongming, Shanghai, China |  |  |  |  |  |  | MT235079 |  |  |  | Ding and Chen (2021), direct submission |  |
| *Fejervarya kawamurai* (Clade II) | *Fejervarya multistriata* | ZWd1244_BZ | Chongming, Shanghai, China |  |  |  |  |  |  | MT235080 |  |  |  | Ding and Chen (2021), direct submission |  |
| *Fejervarya kawamurai* (Clade II) | *Fejervarya multistriata* | ZWd1339_MZ | Chongming, Shanghai, China |  |  |  |  |  |  | MT235107 |  |  |  | Ding and Chen (2021), direct submission |  |
| *Fejervarya kawamurai* (Clade II) | *Fejervarya multistriata* | ZWd1343_MZ | Chongming, Shanghai, China |  |  |  |  |  |  | MT235113 |  |  |  | Ding and Chen (2021), direct submission |  |
| *Fejervarya kawamurai* (Clade II) | *Fejervarya multistriata* | ZWd1515_MT | Chongming, Shanghai, China |  |  |  |  |  |  | MT235099 |  |  |  | Ding and Chen (2021), direct submission |  |
| *Fejervarya kawamurai* (Clade II) | *Fejervarya multistriata* | ZWd1516_MT | Chongming, Shanghai, China |  |  |  |  |  |  | MT235176 |  |  |  | Ding and Chen (2021), direct submission |  |
| *Fejervarya kawamurai* (Clade II) | *Fejervarya multistriata* | ZWd1525_MT | Chongming, Shanghai, China |  |  |  |  |  |  | MT235165 |  |  |  | Ding and Chen (2021), direct submission |  |
| *Fejervarya kawamurai* (Clade II) | *Fejervarya multistriata* | ZWd1548a_MZH | Chongming, Shanghai, China |  |  |  |  |  |  | MT235096 |  |  |  | Ding and Chen (2021), direct submission |  |
| *Fejervarya kawamurai* (Clade II) | *Fejervarya multistriata* | ZWd1613_BBY | Chongming, Shanghai, China |  |  |  |  |  |  | MT235083 |  |  |  | Ding and Chen (2021), direct submission |  |
| *Fejervarya kawamurai* (Clade II) | *Fejervarya multistriata* | ZWd1617b_BBY | Chongming, Shanghai, China |  |  |  |  |  |  | MT235201 |  |  |  | Ding and Chen (2021), direct submission |  |
| *Fejervarya kawamurai* (Clade II) | *Fejervarya multistriata* | ZWd1619b_BBY | Chongming, Shanghai, China |  |  |  |  |  |  | MT235193 |  |  |  | Ding and Chen (2021), direct submission |  |
| *Fejervarya kawamurai* (Clade II) | *Fejervarya multistriata* | ZWd1621b_BBY | Chongming, Shanghai, China |  |  |  |  |  |  | MT235164 |  |  |  | Ding and Chen (2021), direct submission |  |
| *Fejervarya kawamurai* (Clade II) | *Fejervarya multistriata* | ZWd1634_BBY | Chongming, Shanghai, China |  |  |  |  |  |  | MT235089 |  |  |  | Ding and Chen (2021), direct submission |  |
| *Fejervarya kawamurai* (Clade II) | *Fejervarya multistriata* | ZWd1641b_BBY | Chongming, Shanghai, China |  |  |  |  |  |  | MT235069 |  |  |  | Ding and Chen (2021), direct submission |  |
| *Fejervarya kawamurai* (Clade II) | *Fejervarya multistriata* | ZWd1642b_BBY | Chongming, Shanghai, China |  |  |  |  |  |  | MT235091 |  |  |  | Ding and Chen (2021), direct submission |  |
| *Fejervarya kawamurai* (Clade II) | *Fejervarya multistriata* | ZWd1647b_BBY | Chongming, Shanghai, China |  |  |  |  |  |  | MT235091 |  |  |  | Ding and Chen (2021), direct submission |  |
| *Fejervarya kawamurai* (Clade II) | *Fejervarya multistriata* | ZWd1813b_DP | Chongming, Shanghai, China |  |  |  |  |  |  | MT235162 |  |  |  | Ding and Chen (2021), direct submission |  |
| *Fejervarya kawamurai* (Clade II) | *Fejervarya multistriata* | ZWd1814_DP | Chongming, Shanghai, China |  |  |  |  |  |  | MT235117 |  |  |  | Ding and Chen (2021), direct submission |  |
| *Fejervarya kawamurai* (Clade II) | *Fejervarya multistriata* | ZWd1814b_DP | Chongming, Shanghai, China |  |  |  |  |  |  | MT235118 |  |  |  | Ding and Chen (2021), direct submission |  |
| *Fejervarya kawamurai* (Clade II) | *Fejervarya multistriata* | ZWd1818_DP | Chongming, Shanghai, China |  |  |  |  |  |  | MT235120 |  |  |  | Ding and Chen (2021), direct submission |  |
| *Fejervarya kawamurai* (Clade II) | *Fejervarya multistriata* | ZWd1818b_DP | Chongming, Shanghai, China |  |  |  |  |  |  | MT235121 |  |  |  | Ding and Chen (2021), direct submission |  |
| *Fejervarya kawamurai* (Clade II) | *Fejervarya multistriata* | ZWd1819sb_DP | Chongming, Shanghai, China |  |  |  |  |  |  | MT235123 |  |  |  | Ding and Chen (2021), direct submission |  |
| *Fejervarya kawamurai* (Clade II) | *Fejervarya multistriata* | ZWd1842_DP | Chongming, Shanghai, China |  |  |  |  |  |  | MT235124 |  |  |  | Ding and Chen (2021), direct submission |  |
| *Fejervarya kawamurai* (Clade II) | *Fejervarya multistriata* | ZWdBBY09_BBY | Chongming, Shanghai, China |  |  |  |  |  |  | MT235211 |  |  |  | Ding and Chen (2021), direct submission |  |
| *Fejervarya kawamurai* (Clade II) | *Fejervarya multistriata* | ZWdBBY29_BBY | Chongming, Shanghai, China |  |  |  |  |  |  | MT235167 |  |  |  | Ding and Chen (2021), direct submission |  |
| *Fejervarya kawamurai* (Clade II) | *Fejervarya multistriata* | ZWdBZ03_BZ | Chongming, Shanghai, China |  |  |  |  |  |  | MT235203 |  |  |  | Ding and Chen (2021), direct submission |  |
| *Fejervarya kawamurai* (Clade II) | *Fejervarya multistriata* | ZWdBZ18_BZ | Chongming, Shanghai, China |  |  |  |  |  |  | MT235177 |  |  |  | Ding and Chen (2021), direct submission |  |
| *Fejervarya kawamurai* (Clade II) | *Fejervarya multistriata* | ZWdBZ22_BZ | Chongming, Shanghai, China |  |  |  |  |  |  | MT235180 |  |  |  | Ding and Chen (2021), direct submission |  |
| *Fejervarya kawamurai* (Clade II) | *Fejervarya multistriata* | ZWdCX03_CX | Chongming, Shanghai, China |  |  |  |  |  |  | MT235180 |  |  |  | Ding and Chen (2021), direct submission |  |
| *Fejervarya kawamurai* (Clade II) | *Fejervarya multistriata* | ZWdCX16_CX | Chongming, Shanghai, China |  |  |  |  |  |  | MT235184 |  |  |  | Ding and Chen (2021), direct submission |  |
| *Fejervarya kawamurai* (Clade II) | *Fejervarya multistriata* | ZWdCXD1033_CX | Chongming, Shanghai, China |  |  |  |  |  |  | MT235103 |  |  |  | Ding and Chen (2021), direct submission |  |
| *Fejervarya kawamurai* (Clade II) | *Fejervarya multistriata* | ZWdDL01_DL | Chongming, Shanghai, China |  |  |  |  |  |  | MT235132 |  |  |  | Ding and Chen (2021), direct submission |  |
| *Fejervarya kawamurai* (Clade II) | *Fejervarya multistriata* | ZWdDL04_DL | Chongming, Shanghai, China |  |  |  |  |  |  | MT235134 |  |  |  | Ding and Chen (2021), direct submission |  |
| *Fejervarya kawamurai* (Clade II) | *Fejervarya multistriata* | ZWdDL06_DL | Chongming, Shanghai, China |  |  |  |  |  |  | MT235136 |  |  |  | Ding and Chen (2021), direct submission |  |
| *Fejervarya kawamurai* (Clade II) | *Fejervarya multistriata* | ZWdDL07_DL | Chongming, Shanghai, China |  |  |  |  |  |  | MT235137 |  |  |  | Ding and Chen (2021), direct submission |  |
| *Fejervarya kawamurai* (Clade II) | *Fejervarya multistriata* | ZWdDL09_DL | Chongming, Shanghai, China |  |  |  |  |  |  | MT235138 |  |  |  | Ding and Chen (2021), direct submission |  |
| *Fejervarya kawamurai* (Clade II) | *Fejervarya multistriata* | ZWdDL14_DL | Chongming, Shanghai, China |  |  |  |  |  |  | MT235141 |  |  |  | Ding and Chen (2021), direct submission |  |
| *Fejervarya kawamurai* (Clade II) | *Fejervarya multistriata* | ZWdDL15_DL | Chongming, Shanghai, China |  |  |  |  |  |  | MT235142 |  |  |  | Ding and Chen (2021), direct submission |  |
| *Fejervarya kawamurai* (Clade II) | *Fejervarya multistriata* | ZWdDL17_DL | Chongming, Shanghai, China |  |  |  |  |  |  | MT235161 |  |  |  | Ding and Chen (2021), direct submission |  |
| *Fejervarya kawamurai* (Clade II) | *Fejervarya multistriata* | ZWdDL18_DL | Chongming, Shanghai, China |  |  |  |  |  |  | MT235144 |  |  |  | Ding and Chen (2021), direct submission |  |
| *Fejervarya kawamurai* (Clade II) | *Fejervarya multistriata* | ZWdDP12_DP | Chongming, Shanghai, China |  |  |  |  |  |  | MT235158 |  |  |  | Ding and Chen (2021), direct submission |  |
| *Fejervarya kawamurai* (Clade II) | *Fejervarya multistriata* | ZWdDP18_DP | Chongming, Shanghai, China |  |  |  |  |  |  | MT235159 |  |  |  | Ding and Chen (2021), direct submission |  |
| *Fejervarya kawamurai* (Clade II) | *Fejervarya multistriata* | ZWdDT2_DT | Chongming, Shanghai, China |  |  |  |  |  |  | MT235160 |  |  |  | Ding and Chen (2021), direct submission |  |
| *Fejervarya kawamurai* (Clade II) | *Fejervarya multistriata* | ZWdHS001_HS | Chongming, Shanghai, China |  |  |  |  |  |  | MT235090 |  |  |  | Ding and Chen (2021), direct submission |  |
| *Fejervarya kawamurai* (Clade II) | *Fejervarya multistriata* | ZWdHS037_HS | Chongming, Shanghai, China |  |  |  |  |  |  | MT235200 |  |  |  | Ding and Chen (2021), direct submission |  |
| *Fejervarya kawamurai* (Clade II) | *Fejervarya multistriata* | ZWdHS12_HS | Chongming, Shanghai, China |  |  |  |  |  |  | MT235188 |  |  |  | Ding and Chen (2021), direct submission |  |
| *Fejervarya kawamurai* (Clade II) | *Fejervarya multistriata* | ZWdHS17_HS | Chongming, Shanghai, China |  |  |  |  |  |  | MT235190 |  |  |  | Ding and Chen (2021), direct submission |  |
| *Fejervarya kawamurai* (Clade II) | *Fejervarya multistriata* | ZWdMT02_MT | Chongming, Shanghai, China |  |  |  |  |  |  | MT235125 |  |  |  | Ding and Chen (2021), direct submission |  |
| *Fejervarya kawamurai* (Clade II) | *Fejervarya multistriata* | ZWdMT03_MT | Chongming, Shanghai, China |  |  |  |  |  |  | MT235126 |  |  |  | Ding and Chen (2021), direct submission |  |
| *Fejervarya kawamurai* (Clade II) | *Fejervarya multistriata* | ZWdMT08_MT | Chongming, Shanghai, China |  |  |  |  |  |  | MT235127 |  |  |  | Ding and Chen (2021), direct submission |  |
| *Fejervarya kawamurai* (Clade II) | *Fejervarya multistriata* | ZWdMT09_MT | Chongming, Shanghai, China |  |  |  |  |  |  | MT235128 |  |  |  | Ding and Chen (2021), direct submission |  |
| *Fejervarya kawamurai* (Clade II) | *Fejervarya multistriata* | ZWdMT13_MT | Chongming, Shanghai, China |  |  |  |  |  |  | MT235130 |  |  |  | Ding and Chen (2021), direct submission |  |
| *Fejervarya kawamurai* (Clade II) | *Fejervarya multistriata* | ZWdMT18_MT | Chongming, Shanghai, China |  |  |  |  |  |  | MT235135 |  |  |  | Ding and Chen (2021), direct submission |  |
| *Fejervarya kawamurai* (Clade II) | *Fejervarya multistriata* | ZWdMZ06_MZ | Chongming, Shanghai, China |  |  |  |  |  |  | MT235194 |  |  |  | Ding and Chen (2021), direct submission |  |
| *Fejervarya kawamurai* (Clade II) | *Fejervarya multistriata* | ZWdMZ11_MZ | Chongming, Shanghai, China |  |  |  |  |  |  | MT235196 |  |  |  | Ding and Chen (2021), direct submission |  |
| *Fejervarya kawamurai* (Clade II) | *Fejervarya multistriata* | ZWdMZ13_MZ | Chongming, Shanghai, China |  |  |  |  |  |  | MT235198 |  |  |  | Ding and Chen (2021), direct submission |  |
| *Fejervarya kawamurai* (Clade II) | *Fejervarya multistriata* | ZWdMZ14_MZ | Chongming, Shanghai, China |  |  |  |  |  |  | MT235199 |  |  |  | Ding and Chen (2021), direct submission |  |
| *Fejervarya kawamurai* (Clade II) | *Fejervarya multistriata* | ZWdMZ26_MZ | Chongming, Shanghai, China |  |  |  |  |  |  | MT235202 |  |  |  | Ding and Chen (2021), direct submission |  |
| *Fejervarya kawamurai* (Clade II) | *Fejervarya multistriata* | ZWdMZ30_MZ | Chongming, Shanghai, China |  |  |  |  |  |  | MT235204 |  |  |  | Ding and Chen (2021), direct submission |  |
| *Fejervarya kawamurai* (Clade II) | *Fejervarya multistriata* | ZWdMZ32_MZ | Chongming, Shanghai, China |  |  |  |  |  |  | MT235205 |  |  |  | Ding and Chen (2021), direct submission |  |
| *Fejervarya kawamurai* (Clade II) | *Fejervarya multistriata* | ZWdMZ35_MZ | Chongming, Shanghai, China |  |  |  |  |  |  | MT235206 |  |  |  | Ding and Chen (2021), direct submission |  |
| *Fejervarya kawamurai* (Clade II) | *Fejervarya multistriata* | ZWdMZ46_MZ | Chongming, Shanghai, China |  |  |  |  |  |  | MT235213 |  |  |  | Ding and Chen (2021), direct submission |  |
| *Fejervarya kawamurai* (Clade II) | *Fejervarya multistriata* | ZWdMZ47_MZ | Chongming, Shanghai, China |  |  |  |  |  |  | MT235214 |  |  |  | Ding and Chen (2021), direct submission |  |
| *Fejervarya kawamurai* (Clade II) | *Fejervarya multistriata* | ZWdMZH04_MZH | Chongming, Shanghai, China |  |  |  |  |  |  | MT235146 |  |  |  | Ding and Chen (2021), direct submission |  |
| *Fejervarya kawamurai* (Clade II) | *Fejervarya multistriata* | ZWdMZH09_MZH | Chongming, Shanghai, China |  |  |  |  |  |  | MT235166 |  |  |  | Ding and Chen (2021), direct submission |  |
| *Fejervarya kawamurai* (Clade II) | *Fejervarya multistriata* | ZWdMZH16_MZH | Chongming, Shanghai, China |  |  |  |  |  |  | MT235148 |  |  |  | Ding and Chen (2021), direct submission |  |
| *Fejervarya kawamurai* (Clade II) | *Fejervarya multistriata* | ZWdQXJ43_QXJ | Chongming, Shanghai, China |  |  |  |  |  |  | MT235155 |  |  |  | Ding and Chen (2021), direct submission |  |
| *Fejervarya kawamurai* (Clade II) | *Fejervarya multistriata* | ZWd1819_DP | Chongming, Shanghai, China |  |  |  |  |  |  | MT235122 |  |  |  | Ding and Chen (2021), direct submission |  |
| *Fejervarya kawamurai* (Clade II) | *Fejervarya multistriata* | ZWdBHQ06_DT | Chongming, Shanghai, China |  |  |  |  |  |  | MT235171 |  |  |  | Ding and Chen (2021), direct submission |  |
| *Fejervarya kawamurai* (Clade II) | *Fejervarya multistriata* | ZWdMZ36_MZ | Chongming, Shanghai, China |  |  |  |  |  |  | MT235207 |  |  |  | Ding and Chen (2021), direct submission |  |
| *Fejervarya multistriata* (Clade II) | *Fejervarya limnocharis* | 15083 | Taiwan Province, Taichung City, Taichung |  |  |  |  |  | MH034192 |  |  |  |  | Jang-Liaw and Chao (2008) |  |
| *Fejervarya multistriata* (Clade II) | *Fejervarya limnocharis* | 17691 | Lienchiang County, Nangan Township, Fuwoling, Taiwan Island | 26.156 | 119.947 |  |  |  | MH034196 |  |  |  |  | Jang-Liaw and Chao (2008) |  |
| *Fejervarya multistriata* (Clade II) | *Fejervarya limnocharis* | 17744 |  | 25.268 | 121.551 |  |  |  | MH034190 |  |  |  |  | Jang-Liaw and Chao (2008) |  |
| *Fejervarya multistriata* (Clade I) | *Fejervarya limnocharis* | 17620 | Guangdong, Shaoguan City, Juyuan County, Tianjingshan Forestry Station, China | 24.698 | 112.988 |  |  |  | MH034194 |  |  |  |  | Jang-Liaw and Chao (2008) |  |
| *Fejervarya multistriata* (Clade I) | *Fejervarya limnocharis* | 17674 | Fujian, Xiamen City, Xiamen City, Xiamen  University, China | 24.442 | 118.099 |  |  |  | MH034195 |  |  |  |  | Jang-Liaw and Chao (2008) |  |
| *Fejervarya multistriata* (Clade II) | *Fejervarya limnocharis* | 18178 | Taiwan Province, Pintung County, Jiadung Township, Hsiaputou | 22.4092 | 120.561 |  |  |  | MH034191 |  |  |  |  | Jang-Liaw and Chao (2008) |  |
| *Fejervarya multistriata* (Clade II) | *Fejervarya limnocharis* | 18238 | Taiwan Province, Yilan County, Toucheng Township, Toucheng | 24.893 | 121.856 |  |  |  | MH034197 |  |  |  |  | Jang-Liaw and Chao (2008) |  |
| *Fejervarya multistriata* (Clade II) | *Fejervarya limnocharis* | JL73-2-1 | Taiwan: Taiwan Province, Yilan County, Nan-ao | 24.4388 | 121.764 |  |  |  | MH034202 |  |  |  |  | Jang-Liaw and Chao (2008) |  |
| *Fejervarya multistriata* (Clade II) | *Fejervarya limnocharis* | JL73-2-2 | Taiwan: Taiwan Province, Yilan County, Nan-ao | 24.4388 | 121.764 |  |  |  |  |  |  |  |  | Jang-Liaw and Chao (2008) |  |
| *Fejervarya multistriata* (Clade II) | *Fejervarya limnocharis* | JL106 | Taiwan: Taiwan Province, Tainan City, Liouying  District, Liouying | 23.261 | 120.378 |  |  |  | MH034204 |  |  |  |  | Jang-Liaw and Chao (2008) |  |
| *Fejervarya multistriata* (Clade II) | *Fejervarya limnocharis* | JL66-2 | Taiwan: Taiwan Province, Pingtung County, Sandimen County, Sandimen | 22.7519 | 120.636 |  |  |  | MH034201 |  |  |  |  | Jang-Liaw and Chao (2008) |  |
| *Fejervarya multistriata* (Clade II) | *Fejervarya limnocharis* | JL-19-2 | Taiwan Province, Hualien County, Shoufeng Township, Shoufeng | 23.8823 | 121.568 |  |  |  | MH034200 |  |  |  |  | Jang-Liaw and Chao (2008) |  |
| *Fejervarya limnocharis* (Clade II) | *Fejervarya limnocharis* | G024 | Hainan Island, China |  |  |  | KR827744 |  | KR087654 |  |  |  |  | Grosjean et al. (2015) |  |
| *Fejervarya limnocharis* (Clade II) | *Fejervarya limnocharis* | JL02-1 | Chubu, Shizuoka Prefecture, Central, Japan | 34.988 | 138.299 |  |  |  | MH034198 |  |  |  |  | Jang-Liaw and Chao (2008) |  |
| *Fejervarya limnocharis* (Clade II) | *Fejervarya multistriata* | NMNSF15460 | Taiwan Province, Pingtung County, Manjhou Township, Chiupeng | 22.1033 | 120.847 |  |  |  | MH034193 |  |  |  |  | Jang-Liaw and Chao (2008) |  |
| *Fejervarya limnocharis* (Clade I) | *Fejervarya multistriata* | fm_1 | Possibly central China (Shaanxi) |  |  |  |  |  | OL762406 |  |  |  |  | Wang (2021), direct submission |  |
| *Fejervarya limnocharis* (Clade I) | *Fejervarya limnocharis* | TAD-P992 | Thailand: Phangnga, Namtok Raman Forest Park |  |  |  | KR827738 |  | KR087650 |  |  |  |  | Grosjean et al. (2015) |  |
| *Fejervarya kawamurai* (Clade II) | *Fejervarya limnocharis* |  | Japan: Higashihiroshima |  |  |  | AB070732 | AB296095 |  |  |  |  |  | Djong et al. (2007) |  |
| *Fejervarya limnocharis* (Clade I) | *Fejervarya limnocharis* | USNM:Herp:595381 | Viet Nam: Trang An Landscape Complex, south  central extent of Trang An Natural Landscape Complex |  |  |  | OM387117 |  | OM420388 |  |  |  |  | Miller et al. (2022), direct submission |  |
| *Fejervarya limnocharis* (Clade I) | *Fejervarya limnocharis* | USNM:Herp:595383 | iet Nam: Du Gia Forest, Bac Me, 3.2 km E of  Khau Ca Reserve (straight-line distance) |  |  |  | OM387145 |  | OM420416 |  |  |  |  | Miller et al. (2022), direct submission |  |
| *Fejervarya multistriata* (Clade II) | *Fejervarya limnocharis* | H001 | Taiwan |  |  | HQ170489 | HQ226055 | HQ289763 |  | HQ289782 |  |  |  | Chang and Liu (2010), direct submission |  |
| *Fejervarya multistriata* (Clade II) | *Fejervarya limnocharis* | H002 | Taiwan Island |  |  | HQ170490 | HQ226056 | HQ289764 |  | HQ289783 |  |  |  | Chang and Liu (2010), direct submission |  |
| *Fejervarya multistriata* (Clade II) | *Fejervarya limnocharis* | H005 | Taiwan Island |  |  | HQ170493 | HQ226059 | HQ289767 |  | HQ289786 |  |  |  | Chang and Liu (2010), direct submission |  |
| *Fejervarya multistriata* (Clade II) | *Fejervarya limnocharis* | H011 | Taiwan Island |  |  | HQ170499 | HQ226065 | HQ289773 |  | HQ289792 |  |  |  | Chang and Liu (2010), direct submission |  |
| *Fejervarya multistriata* (Clade II) | *Fejervarya limnocharis* | H012 | Taiwan Island |  |  | HQ170500 | HQ226066 | HQ289774 |  | HQ289793 |  |  |  | Chang and Liu (2010), direct submission |  |
| *Fejervarya multistriata* (Clade II) | *Fejervarya limnocharis* | H013 | Taiwan Island |  |  | HQ170501 | HQ226067 | HQ289775 |  | HQ289794 |  |  |  | Chang and Liu (2010), direct submission |  |
| *Fejervarya kawamurai* (Clade II) | *Fejervarya multistrata* | GZBY01 | Guangzhou,China |  |  |  |  | MK317476 |  |  | MK317725 | MK317232 | MK317351 | Yang et al. (2023) |  |
| *Fejervarya kawamurai* (Clade II) | *Fejervarya multistrata* | GZBY02 | Guangzhou,China |  |  |  |  | MK317477 |  |  | MK317726 | MK317233 | MK317352 | Yang et al. (2023) |  |
| *Fejervarya kawamurai* (Clade II) | *Fejervarya multistrata* | GZBY04 | Guangzhou,China |  |  |  |  | MK317479 |  |  | MK317728 | MK317235 | MK317354 | Yang et al. (2023) |  |
| *Fejervarya kawamurai* (Clade II) | *Fejervarya multistrata* | GZBY05 | Guangzhou,China |  |  |  |  | MT254032 |  |  |  |  |  | Yang et al. (2023) |  |
| *Fejervarya multistriata* (Clade I) | *Fejervarya multistrata* | mtDNA | Santai County, Mianyang City, Sichuan, China | 105°7′49.89 | 31°10′41.55 | MN987553 | MN987553 | MN987553 | MN987553 | MN987553 |  |  |  | Jiang et al. (2020) |  |
| *Fejervarya multistriata* (Clade I) | *Fejervarya multistrata* | ZWd1339 | Chongming, Shanghai, China |  |  |  |  |  |  | MT235107 |  |  |  | Ding and Chen (2021) |  |
| *Fejervarya multistriata* (Clade I) | *Fejervarya multistrata* | MZ44_MZ | Chongming, Shanghai, China |  |  |  |  | MT254032 |  |  |  |  |  | Ding and Chen (2021) |  |
| *Fejervarya cancrivora* | *Fejervarya cancrivora* | full mtDNA | Beihai, Guangxi, China |  |  | NC_012647 | NC_012647 | NC_012647 | NC_012647 | NC_012647 |  |  |  | ren et al. (2009) |  |
| *Fejervarya cancrivora* | *Fejervarya cancrivora* | MS-2009 (MS-can-Mal) | Selangor, Malaysia |  |  |  |  |  |  |  | AB500255 |  |  | Kotaki et al. (2010) |  |
| *Fejervarya cancrivora* | *Fejervarya cancrivora* | Jiadong-13 | Jiadong, China |  |  |  | EU435283 |  |  |  |  |  |  | Kung et al. (2008), direct submission |  |
| *Fejervarya cancrivora* | *Fejervarya cancrivora* | FCL-16S-1 | Cianjur, Java, Indonesia |  |  |  | AB444684 |  |  |  |  |  |  | Kurniawan et al. (2010) |  |
| *Fejervarya cancrivora* | *Fejervarya cancrivora* | FCL-16S-2 | West Sumatra, Padang, Indonesia |  |  |  | AB444685 |  |  |  |  |  |  | Kurniawan et al. (2010) |  |
| *Fejervarya cancrivora* | *Fejervarya cancrivora* | FCL-16S-7 | Central Java, Banyumas, Indonesia |  |  |  | AB444690 | AB444700 |  |  |  |  |  | Kurniawan et al. (2010) |  |
| *Fejervarya moodiei* | *Fejervarya moodiei* | IEBR A.5085 | Nam Dinh, Vietnam |  |  |  | ON870696 | ON936022 |  |  |  |  |  | Chung et al. (2022) |  |
| *Fejervarya moodiei* | *Fejervarya moodiei* | IEBR A.5089 | Nam Dinh, Vietnam |  |  |  | ON870700 | ON936023 |  |  |  |  |  | Chung et al. (2022) |  |
| *Fejervarya moodiei* | *Fejervarya moodiei* | IEBR A.5091 | Nam Dinh, Vietnam |  |  |  | ON870702 | ON936024 |  |  |  |  |  | Chung et al. (2022) |  |
| *Fejervarya moodiei* | *Fejervarya moodiei* | IEBR A.5097 | Thua Thien Hue, Vietnam |  |  |  | ON870707 | ON936028 |  |  |  |  |  | Chung et al. (2022) |  |
| *Fejervarya moodiei* | *Fejervarya moodiei* | IEBR A.5096 | Thua Thien Hue, Vietnam |  |  |  | ON870706 | ON936026 |  |  |  |  |  | Chung et al. (2022) |  |
| *Fejervarya moodiei* | *Fejervarya moodiei* | IEBR A.5098 | Hai Phong, Vietnam |  |  |  | ON870708 | ON936031 |  |  |  |  |  | Chung et al. (2022) |  |
| *Fejervarya moodiei* | *Fejervarya moodiei* | IEBR A.5099 | Hai Phong, Vietnam |  |  |  | ON870709 | ON936032 |  |  |  |  |  | Chung et al. (2022) |  |
| *Fejervarya moodiei* | *Fejervarya moodiei* | IEBR A.5101 | Ca Mau, Vietnam |  |  |  | ON870711 | ON936029 |  |  |  |  |  | Chung et al. (2022) |  |
| *Fejervarya moodiei* | *Fejervarya moodiei* | CG9CaMau | Ca Mau, Vietnam |  |  |  | ON870713 |  |  |  |  |  |  | Chung et al. (2022) |  |
| *Hoplobatrachus chinensis* | *Hoplobatrachus rugulosus* | full mtDNA | Jinhua, Zhejiang, China |  |  | JX181763 | JX181763 | JX181763 | JX181763 | JX181763 |  |  |  | Yu et al. (2015) |  |
| *Hoplobatrachus chinensis* | *Hoplobatrachus rugulosus* | full mtDNA | Anhui, China |  |  | KC196066 | KC196066 | KC196066 | KC196066 | KC196066 |  |  |  | Pan et al. (2012) |  |
| *Hoplobatrachus chinensis* | *Hoplobatrachus chinensis x rugulosus* | full mtDNA | Jinhua, Zhejiang | 29°21′39 | 119°57′58 | NC_042258 | NC_042258 | NC_042258 | NC_042258 | NC_042258 |  |  |  | Cai et al. (2018) |  |
| *Hoplobatrachus chinensis* | *Hoplobatrachus rugulosus* | JHBT-B4 | the Bendi farm in Jinhua |  |  |  |  | KC818470 |  |  |  |  |  | Yu et al. (2015) |  |
| *Hoplobatrachus chinensis* | *Hoplobatrachus rugulosus* | JHBT-B3 | the Bendi farm in Jinhua |  |  |  |  | KC818469 |  |  |  |  |  | Yu et al. (2015) |  |
| *Hoplobatrachus chinensis* | *Hoplobatrachus rugulosus* | JHBT-B2 | the Bendi farm in Jinhua |  |  |  |  | KC818468 |  |  |  |  |  | Yu et al. (2015) |  |
| *Hoplobatrachus chinensis* | *Hoplobatrachus rugulosus* | JHBT-B1 | the Bendi farm in Jinhua |  |  |  |  | KC818467 |  |  |  |  |  | Yu et al. (2015) |  |
| *Hoplobatrachus chinensis* | *Hoplobatrachus rugulosus* | FMNH 255191 | Laos: Champasak Province, Mounlapamok District, Dong Khanthung National Biodiversity Conservation Area |  |  |  |  |  |  |  |  |  | DQ283842 | Frost et al. (2017) |  |
| *Hoplobatrachus chinensis* | *Hoplobatrachus rugulosus* | HN1 | the Hainan farm in Jinhua |  |  |  |  | KC818461 |  |  | KJ637243 | KJ637281 | KJ637300 | Yu et al. (2015) |  |
| *Hoplobatrachus chinensis* | *Hoplobatrachus rugulosus* | HN2 | the Hainan farm in Jinhua, China |  |  |  |  | KC818460 |  |  | KJ637244 | KJ637282 | KJ637301 | Yu et al. (2015) |  |
| *Hoplobatrachus chinensis* | *Hoplobatrachus rugulosus* | HN3 | the Hainan farm in Jinhua, China |  |  |  |  | KC818462 |  |  |  |  |  | Yu et al. (2015) |  |
| *Hoplobatrachus chinensis* | *Hoplobatrachus rugulosus* | HW1 | the Huwawa farm in Jinhua, China |  |  |  |  | KC818463 |  |  |  |  |  | Yu et al. (2015) |  |
| *Hoplobatrachus chinensis* | *Hoplobatrachus rugulosus* | HW5 | the Huwawa farm in Jinhua, China |  |  |  |  | KC818464 |  |  |  |  |  | Yu et al. (2015) |  |
| *Hoplobatrachus chinensis* | *Hoplobatrachus rugulosus* | HW6 | the Huwawa farm in Jinhua, China |  |  |  |  | KC818463 |  |  |  |  |  | Yu et al. (2015) |  |
| *Hoplobatrachus chinensis* | *Hoplobatrachus rugulosus* | THW1 | the Huwawa farm in Jinhua, China |  |  |  |  | KC818464 |  |  |  |  |  | Yu et al. (2015) |  |
| *Hoplobatrachus chinensis* | *Hoplobatrachus rugulosus* | NW1 | the Niuwa farm in Jinhua, China |  |  |  |  | KC818465 |  |  |  |  |  | Yu et al. (2015) |  |
| *Hoplobatrachus chinensis* | *Hoplobatrachus rugulosus* | NW2 | the Niuwa farm in Jinhua, China |  |  |  |  | KC818466 |  |  |  |  |  | Yu et al. (2015) |  |
| *Hoplobatrachus chinensis* | *Hoplobatrachus rugulosus* | JHBT-W7 | the Wu farm in Jinhua, China |  |  |  |  | KC818471 |  |  |  |  |  | Yu et al. (2015) |  |
| *Hoplobatrachus chinensis* | *Hoplobatrachus rugulosus* | JHBT-W8 | the Wu farm in Jinhua, China |  |  |  |  | KC818472 |  |  |  |  | KJ637312 | Yu et al. (2015) |  |
| *Hoplobatrachus chinensis* | *Hoplobatrachus rugulosus* | WZ2 | Jinhua, Zhejiang, China |  |  |  |  | KC818475 |  |  |  |  |  | Yu et al. (2015) |  |
| *Hoplobatrachus chinensis* | *Hoplobatrachus rugulosus* | WZ3 | Jinhua, Zhejiang, China |  |  |  |  | KC818476 |  |  |  |  |  | Yu et al. (2015) |  |
| *Hoplobatrachus tigerinus* | *Hoplobatrachus tigerinus* | full mtDNA | Banglandesh: Mymensingh, China |  |  | NC_014581 | NC_014581 | NC_014581 | NC_014581 | NC_014581 |  |  |  | Alam et al. (2010) |  |
| *Quasipaa spinosa* | *Quasipaa spinosa* | KIZ021611 | Yuanbaoshan, Guangxi, China |  |  |  |  | MT678127 |  |  | MT678354 | MT678406 | MT678311 | Yan et al. (2021) |  |
| *Quasipaa shini* | *Quasipaa shini* | KIZ021613 | Yuanbaoshan, Guangxi, China |  |  |  |  | MT678128 |  |  | MT678355 | MT678407 |  | Yan et al. (2021) |  |
| *Quasipaa spinosa* | *Quasipaa spinosa* | KIZ022191 | Fulong, Guangxi, China |  |  |  |  | MT678129 |  |  | MT678356 | MT678408 | MT678312 | Yan et al. (2021) |  |
| *Quasipaa yei* | *Quasipaa yei* | KIZ02340 | China: Shangcheng, Henan |  |  |  |  |  |  |  | MT678357 | MT678409 | MT678313 | Yan et al. (2021) |  |
| *Quasipaa yei* | *Quasipaa yei* | KIZ02412 | China: Shangcheng, Henan |  |  |  |  | MT678134 |  |  | MT678360 | MT678412 | MT678316 | Yan et al. (2021) |  |
| *Quasipaa boulengeri* | *Quasipaa boulengeri* | KIZ03107 | China: Youyang, Chongqing |  |  |  |  |  |  |  | MT678362 | MT678414 |  | Yan et al. (2021) |  |
| *Quasipaa spinosa* | *Quasipaa spinosa* | KIZ04714 | China: Huizhou, Guangdong |  |  |  |  | MT678162 |  |  | MT678369 | MT678421 | MT678323 | Yan et al. (2021) |  |
| *Quasipaa jiulongensis* | *Quasipaa jiulongensis* | KIZ08918 | Wuyishan, Fujian, China |  |  |  |  | MT678192 |  |  | MT678380 | MT678430 |  | Yan et al. (2021) |  |
| *Quasipaa exilispinosa* | *Quasipaa exilispinosa* | KIZ08932 | Wuyishan, Fujian, China |  |  |  |  | MT678196 |  |  | MT678383 | MT678433 | MT678334 | Yan et al. (2021) |  |
| *Quasipaa spinosa* | *Quasipaa spinosa* | KIZ08997 | Daiyunshan, Fujian, China |  |  |  |  | MT678218 |  |  | MT678384 | MT678434 | MT678335 | Yan et al. (2021) |  |
| *Quasipaa spinosa* | *Quasipaa spinosa* | KIZ09096 | Qixingzhen, Guangdong, China |  |  |  |  | MT678224 |  |  | MT678385 | MT678435 | MT678336 | Yan et al. (2021) |  |
| *Quasipaa spinosa* | *Quasipaa spinosa* | KIZ09282 | Chongzuo, Guangxi, China |  |  |  |  | MT678226 |  |  | MT678387 | MT678437 | MT678338 | Yan et al. (2021) |  |
| *Quasipaa spinosa* | *Quasipaa spinosa* | KIZ09706 | Yangjiang, Guangdong, China |  |  |  |  | MT678231 |  |  | MT678389 | MT678439 | MT678339 | Yan et al. (2021) |  |
| *Quasipaa spinosa* | *Quasipaa spinosa* | KIZJX0709008 | Lushan, Jiangxi, China |  |  |  |  | MT678238 |  |  | MT678397 | MT678447 | MT678347 | Yan et al. (2021) |  |
| *Quasipaa spinosa* | *Quasipaa spinosa* | KIZYPX18607 | Xinying, Guangdong, China |  |  |  |  | MT678244 |  |  | MT678399 | MT678449 | MT678349 | Yan et al. (2021) |  |
| *Quasipaa exilispinosa* | *Quasipaa exilispinosa* | KIZ01575 | Damaoshan, Hongkong |  |  |  |  | MT678126 |  |  | MT724034 | MT723998 | MT724008 | Yan et al. (2021) |  |
| *Quasipaa exilispinosa* | *Quasipaa exilispinosa* | KIZ07219 | Xunwu, Jiangxi, China |  |  |  |  | MT678179 |  |  | MT724035 | MT723999 | MT724009 | Yan et al. (2021) |  |
| *Quasipaa exilispinosa* | *Quasipaa exilispinosa* | KIZ08934 | Wuyishan, Fujian, China |  |  |  |  | MT678198 |  |  | MT724038 |  | MT724012 | Yan et al. (2021) |  |
| *Quasipaa exilispinosa* | *Quasipaa exilispinosa* | KIZ08935 | Wuyishan, Fujian, China |  |  |  |  | MT678199 |  |  | MT724039 | MT724002 | MT724013 | Yan et al. (2021) |  |
| *Quasipaa exilispinosa* | *Quasipaa exilispinosa* | KIZ08936 | Wuyishan, Fujian, China |  |  |  |  | MT678200 |  |  | MT724040 | MT724003 | MT724014 | Yan et al. (2021) |  |
| *Quasipaa exilispinosa* | *Quasipaa exilispinosa* | KIZ08938 | Wuyishan, Fujian, China |  |  |  |  | MT678202 |  |  | MT724042 | MT724004 | MT724016 | Yan et al. (2021) |  |
| *Quasipaa jiulongensis* | *Quasipaa jiulongensis* | KIZ08920 | Wuyishan, Fujian, China |  |  |  |  | MT678193 |  |  | MT678381 | MT678431 | MT678332 | Yan et al. (2021) |  |
| *Quasipaa shini* | *Quasipaa shini* | KIZ02397 | Kaili, Guizhou, China |  |  |  |  | JX676600 |  |  | MT678359 | MT678411 | MT678315 | Yan et al. (2021) |  |
| *Quasipaa spinosa* | *Quasipaa spinosa* | KIZ09282 | Chongzuo, Guangxi, China |  |  |  |  | MT678226 |  |  | MT678387 | MT678437 | MT678338 | Yan et al. (2021) |  |
| *Quasipaa spinosa* | *Quasipaa spinosa* | KIZC21 | Huangshanpo, Anhui |  |  |  |  | MT678233 |  |  | MT678391 | MT678441 | MT678341 | Yan et al. (2021) |  |
| *Quasipaa spinosa* | *Quasipaa spinosa* | KIZ021611 | China: Yuanbaoshan, Guangxi |  |  | 12S |  | MT678127 |  |  | MT678354 | MT678406 | MT678311 | Yan et al. (2021) |  |
| *Fejervarya kawamurai* (Clade I) | *Fejervarya kawamurai* | HiroshimaShiga | Shiga, Japan |  |  |  |  | LC667355 |  |  |  |  |  | Kakehashi et al. (2022) |  |
| *Fejervarya kawamurai* (Clade I) | *Fejervarya kawamurai* | Karasuma | Shiga, Japan |  |  |  |  | LC667356 |  |  |  |  |  | Kakehashi et al. (2022) |  |
| *Fejervarya kawamurai* (Clade I) | *Fejervarya limnocharis* | JL02_1 | Chubu, Shizuoka Prefecture, Central Region, Shizuoka City, Shizuoka, Japan | 34.9884 | 138.299 |  |  |  | MH034198 |  |  |  |  | Kakehashi et al. (2022) |  |
| *Fejervarya kawamurai* (Clade I) | *Fejervarya limnocharis* | Okin | Okinawa, Japan |  |  | AB070726 |  |  |  |  |  |  |  | Sumida et al. (2002) |  |
| *Fejervarya kawamurai* (Clade I) | *Fejervarya limnocharis* | Okin_1_ | Okinawa, Japan |  |  |  | AB070733 |  |  |  |  |  |  | Sumida et al. (2002) |  |
| *Fejervarya kawamurai* (Clade I) | *Fejervarya limnocharis* | Okin_2_ | Okinawa, Japan |  |  |  | AB070734 |  |  |  |  |  |  | Sumida et al. (2002) |  |
| *Fejervarya kawamurai* (Clade I) | *Fejervarya limnocharis* | japo_higa | Higasihiroshima, Kagamiyama, Japan |  |  |  |  | AB296095 |  |  |  |  |  | Djong et al. (2007) |  |
| *Fejervarya kawamurai* (Clade I) | *Fejervarya limnocharis* | japo_hiro | Hiroshima, Japan |  |  |  |  | AB296096 |  |  |  |  |  | Djong et al. (2007) |  |
| *Fejervarya kawamurai* (Clade I) | *Fejervarya* sp. | Hiroshima_1 | Hiroshima, Japan |  |  | AB488864 | AB488887 | AB488832 |  |  | AB489005 | AB488965 | AB489026 | Sumida et al. (2002) |  |
| *Fejervarya multistriata* (Clade II) | *Fejervarya limnocharis* | G024 | Hainan Island, China |  |  |  | KR827744 | AB296094 | KR087654 |  |  |  |  | Grosjean et al. (2015) |  |
| *Fejervarya multistriata* (Clade II) | *Fejervarya multistriata* | HS12001 | Santai County, Mianyang City, Sichuan province, China | 105°7′49.89 | 31°10′41.55 | MN987553 | MN987553 | MN987553 | MN987553 | MN987553 |  |  |  | Jiang et al. (2020) |  |
| *Fejervarya multistriata* (Clade II) | *Fejervarya multistriata* | Husa | Husa, China |  |  | AB488861 | AB488884 | AB488828 |  |  | AB500265 | AB500228 | AB500257 | Kotaki et al. (2010) |  |
| *Fejervarya multistriata* (Clade II) | *Fejervarya multistriata* | zjbj3 | Ji’an city, Jiangxi Province, China |  |  | NC_029754 | NC_029754 | NC_029754 | NC_029754 | NC_029754 |  |  |  | Huang and Tu (2016) |  |
| *Fejervarya limnocharis* (Clade I) | *Fejervarya limnocharis* | 16SF15 | West Kalimantan |  |  |  | KU840567 |  |  |  |  |  |  | Goutte et al. (2016) |  |
| *Fejervarya limnocharis* (Clade I) | *Fejervarya limnocharis* | 16SSG18 | Sabah, Borneo |  |  |  | KU840568 |  |  |  |  |  |  | Goutte et al. (2016) |  |
| *Fejervarya limnocharis* (Clade I) | *Fejervarya limnocharis* | FMNH261984 | Laos |  |  |  | GU934327 |  | GU934370 |  | GU934455 |  |  | Inger and Stuart (2010) |  |
| *Fejervarya limnocharis* (Clade I) | *Fejervarya limnocharis* | MVZ226347 | Southern China |  |  |  |  |  |  |  |  | HM163580 |  | Che et al. (2010) |  |
| *Fejervarya limnocharis* (Clade I) | *Fejervarya limnocharis* | Malay | Malaysia |  |  |  | AB277301 |  |  |  |  |  |  | Kotaki et al. (2008) |  |
| *Fejervarya limnocharis* (Clade I) | *Fejervarya limnocharis* | NA0083 | Sarawak, Malaysia |  |  |  | EU604201 |  |  |  |  |  |  | Zainuddin et al. (2008) |  |
| *Fejervarya limnocharis* (Clade I) | *Fejervarya limnocharis* | NA0084 | Sarawak, Malaysia |  |  |  | EU604200 |  |  |  |  |  |  | Zainuddin et al. (2008) |  |
| *Fejervarya limnocharis* (Clade I) | *Fejervarya limnocharis* | Rano | Ranong, Thailand |  |  | AB162442 | AB162445 |  |  |  |  |  |  | Sumida et al. (2007) |  |
| *Fejervarya limnocharis* (Clade I) | *Fejervarya limnocharis* | SCUM04H001 | Hainan Island, China |  |  | DQ458239 | DQ458253 |  |  |  | DQ458286 |  | DQ458271 | Che et al. (2007) |  |
| *Fejervarya limnocharis* (Clade I) | *Fejervarya limnocharis* | SH0034 | Borneo |  |  | MW007341 |  |  |  |  |  |  |  | Flury et al. (2022) |  |
| *Fejervarya limnocharis* (Clade I) | *Fejervarya limnocharis* | SNP0261 | Sarawak, Malaysia |  |  |  | EU604202 |  |  |  |  |  |  | Zainuddin et al. (2008) |  |
| *Fejervarya limnocharis* (Clade I) | *Fejervarya limnocharis* | Thai 1 | Nakhon Si Thammarat, Thailand |  |  |  |  |  |  |  | AB277347 |  |  | Kotaki et al. (2008) |  |
| *Fejervarya limnocharis* (Clade I) | *Fejervarya limnocharis* | Thai 5 | Sanam Chaiket, Thailand |  |  |  |  |  |  |  |  | AB277337 |  | Kotaki et al. (2008) |  |
| *Fejervarya limnocharis* (Clade I) | *Fejervarya limnocharis* | Thai 6 | Bangkok, Thailand |  |  |  |  |  |  |  |  | AB277338 |  | Kotaki et al. (2008) |  |
| *Fejervarya limnocharis* (Clade I) | *Fejervarya limnocharis* | Thai 7 | Sara Buri, Thailand |  |  |  |  |  |  |  |  | AB277339 |  | Kotaki et al. (2008) |  |
| *Fejervarya limnocharis* (Clade I) | *Fejervarya limnocharis* | SH13_027 | Borneo |  |  | MW007340 |  |  |  |  |  |  |  | Flury et al. (2022) |  |
| *Fejervarya limnocharis* (Clade I) | *Fejervarya* cf. *limnocharis* | lim 1 | Java, Indonesia |  |  |  |  | AB488811 |  |  |  | AB500227 |  | Kotaki et al. (2010) |  |
| *Fejervarya limnocharis* (Clade I) | *Fejervarya* cf. *limnocharis* | lim 10 | Sapa, Vietnam |  |  |  |  | AB488826 |  |  |  |  |  | Kotaki et al. (2010) |  |
| *Fejervarya limnocharis* (Clade I) | *Fejervarya* cf. *limnocharis* | lim 11 | Phongsaly, Laos |  |  |  |  | AB488827 |  |  |  |  |  | Kotaki et al. (2010) |  |
| *Fejervarya limnocharis* (Clade I) | *Fejervarya* cf. *limnocharis* | lim 12 | Cambodia |  |  |  |  | AB488833 |  |  |  |  |  | Kotaki et al. (2010) |  |
| *Fejervarya limnocharis* (Clade I) | *Fejervarya* cf. *limnocharis* | lim 2 | Java, Indonesia |  |  |  |  | AB488812 |  |  |  |  |  | Kotaki et al. (2010) |  |
| *Fejervarya limnocharis* (Clade I) | *Fejervarya* cf. *limnocharis* | lim 3 | Kuala Lumpur, Malaysia |  |  |  |  | AB488815 |  |  |  |  |  | Kotaki et al. (2010) |  |
| *Fejervarya limnocharis* (Clade I) | *Fejervarya* cf. *limnocharis* | lim 4 | Kuala Lumpur, Malaysia |  |  |  |  | AB488816 |  |  |  |  |  | Kotaki et al. (2010) |  |
| *Fejervarya limnocharis* (Clade I) | *Fejervarya* cf. *limnocharis* | lim 5 | Ranong, Thailand |  |  |  |  | AB488818 |  |  |  |  |  | Kotaki et al. (2010) |  |
| *Fejervarya limnocharis* (Clade I) | *Fejervarya* cf. *limnocharis* | lim 6 | Tha Ton, Thailand |  |  |  |  | AB488819 |  |  |  |  |  | Kotaki et al. (2010) |  |
| *Fejervarya limnocharis* (Clade I) | *Fejervarya* cf. *limnocharis* | lim 8 | Phang Nga, Thailand |  |  |  |  | AB488824 |  |  |  |  |  | Kotaki et al. (2010) |  |
| *Fejervarya limnocharis* (Clade I) | *Fejervarya* cf. *limnocharis* | lim 9 | Phang Nga, Thailand |  |  |  |  | AB488825 |  |  |  |  |  | Kotaki et al. (2010) |  |
| *Fejervarya limnocharis* (Clade I) | *Fejervarya* cf. *limnocharis* | limno | Java, Indonesia |  |  | AB277285 | AB277302 |  |  |  | AB277354 | AB277341 |  | Kotaki et al. (2010) |  |

**S2 Table. Call properties of *Fejervarya kawamurai* (putative Yangtze clade) sampled from Nanjing.** For each of six males, 10 consecutive calls were recorded within a one-minute recording period to assess the stereotypy of acoustic traits.

| **Species (replicate)** | **Call Duration (s)** | **Call period (s)** | **Call repetition rate/s** | **Inter-call interval (s)** | **Pulses per call** | **Pulse rate (pulse/s)** | **Dominant frequency (Hz)** | **Pulse duration (s)** |
| --- | --- | --- | --- | --- | --- | --- | --- | --- |
| *Fejervarya kawamurai* male 1 call 1 | 0.079 | 0.281 | 3.559 | 0.202 | 7.000 | 86.957 | 2928.500 | 0.011 |
| *Fejervarya kawamurai* male 1 call 2 | 0.075 | 0.250 | 4.000 | 0.175 | 7.000 | 90.909 | 2928.500 | 0.011 |
| *Fejervarya kawamurai* male 1 call 3 | 0.081 | 0.240 | 4.167 | 0.159 | 7.000 | 85.714 | 2928.500 | 0.011 |
| *Fejervarya kawamurai* male 1 call 4 | 0.080 | 0.160 | 6.250 | 0.080 | 7.000 | 88.235 | 2971.600 | 0.011 |
| *Fejervarya kawamurai* male 1 call 5 | 0.083 | 0.164 | 6.098 | 0.081 | 7.000 | 88.235 | 1421.200 | 0.011 |
| *Fejervarya kawamurai* male 1 call 6 | 0.081 | 0.160 | 6.250 | 0.079 | 7.000 | 86.957 | 1421.200 | 0.011 |
| *Fejervarya kawamurai* male 1 call 7 | 0.083 | 0.164 | 6.098 | 0.081 | 7.000 | 88.235 | 2971.600 | 0.011 |
| *Fejervarya kawamurai* male 1 call 8 | 0.084 | 0.168 | 5.952 | 0.084 | 7.000 | 84.507 | 2971.600 | 0.011 |
| *Fejervarya kawamurai* male 1 call 9 | 0.085 | 0.263 | 3.802 | 0.178 | 7.000 | 86.957 | 2971.600 | 0.011 |
| *Fejervarya kawamurai* male 1 call 10 | 0.086 | 0.266 | 3.759 | 0.180 | 8.000 | 89.744 | 1421.200 | 0.011 |
| *Fejervarya kawamurai* male 2 call 1 | 0.106 | 0.225 | 4.444 | 0.119 | 9.000 | 86.022 | 1421.200 | 0.012 |
| *Fejervarya kawamurai* male 2 call 2 | 0.101 | 0.224 | 4.464 | 0.123 | 9.000 | 87.912 | 1421.200 | 0.011 |
| *Fejervarya kawamurai* male 2 call 3 | 0.105 | 0.263 | 3.802 | 0.158 | 10.000 | 89.109 | 1378.100 | 0.012 |
| *Fejervarya kawamurai* male 2 call 4 | 0.109 | 0.275 | 3.636 | 0.166 | 10.000 | 86.538 | 1378.100 | 0.012 |
| *Fejervarya kawamurai* male 2 call 5 | 0.104 | 0.274 | 3.650 | 0.170 | 9.000 | 85.106 | 1378.100 | 0.012 |
| *Fejervarya kawamurai* male 2 call 6 | 0.111 | 0.266 | 3.759 | 0.155 | 10.000 | 87.379 | 1378.100 | 0.012 |
| *Fejervarya kawamurai* male 2 call 7 | 0.107 | 0.312 | 3.205 | 0.205 | 9.000 | 86.957 | 1378.100 | 0.011 |
| *Fejervarya kawamurai* male 2 call 8 | 0.104 | 0.618 | 1.618 | 0.514 | 9.000 | 86.022 | 1378.100 | 0.011 |
| *Fejervarya kawamurai* male 2 call 9 | 0.093 | 0.260 | 3.846 | 0.167 | 8.000 | 84.337 | 1421.200 | 0.011 |
| *Fejervarya kawamurai* male 2 call 10 | 0.107 | 0.275 | 3.636 | 0.168 | 9.000 | 84.211 | 1378.100 | 0.012 |
| *Fejervarya kawamurai* male 3 call 1 | 0.063 | 0.314 | 3.185 | 0.251 | 6.000 | 90.909 | 1378.100 | 0.011 |
| *Fejervarya kawamurai* male 3 call 2 | 0.081 | 0.421 | 2.375 | 0.340 | 7.000 | 86.957 | 1335.100 | 0.011 |
| *Fejervarya kawamurai* male 3 call 3 | 0.064 | 0.383 | 2.611 | 0.319 | 6.000 | 89.286 | 1335.100 | 0.011 |
| *Fejervarya kawamurai* male 3 call 4 | 0.077 | 0.308 | 3.247 | 0.231 | 7.000 | 89.552 | 1378.100 | 0.011 |
| *Fejervarya kawamurai* male 3 call 5 | 0.092 | 0.306 | 3.268 | 0.214 | 8.000 | 88.608 | 1421.200 | 0.011 |
| *Fejervarya kawamurai* male 3 call 6 | 0.088 | 0.329 | 3.040 | 0.241 | 8.000 | 89.744 | 1378.100 | 0.012 |
| *Fejervarya kawamurai* male 3 call 7 | 0.087 | 0.337 | 2.967 | 0.250 | 8.000 | 89.744 | 1378.100 | 0.011 |
| *Fejervarya kawamurai* male 3 call 8 | 0.077 | 0.506 | 1.976 | 0.429 | 7.000 | 89.552 | 1335.100 | 0.011 |
| *Fejervarya kawamurai* male 3 call 9 | 0.081 | 0.495 | 2.020 | 0.414 | 7.000 | 89.552 | 1378.100 | 0.011 |
| *Fejervarya kawamurai* male 3 call 10 | 0.078 | 0.318 | 3.145 | 0.240 | 7.000 | 89.552 | 1335.100 | 0.011 |
| *Fejervarya kawamurai* male 4 call 1 | 0.105 | 0.284 | 3.521 | 0.179 | 9.000 | 85.106 | 1292.000 | 0.012 |
| *Fejervarya kawamurai* male 4 call 2 | 0.107 | 0.283 | 3.534 | 0.176 | 9.000 | 84.211 | 1292.000 | 0.012 |
| *Fejervarya kawamurai* male 4 call 3 | 0.111 | 0.381 | 2.625 | 0.270 | 10.000 | 87.379 | 1292.000 | 0.012 |
| *Fejervarya kawamurai* male 4 call 4 | 0.107 | 0.290 | 3.448 | 0.183 | 9.000 | 85.106 | 1292.000 | 0.011 |
| *Fejervarya kawamurai* male 4 call 5 | 0.108 | 0.343 | 2.915 | 0.235 | 9.000 | 85.106 | 1292.000 | 0.012 |
| *Fejervarya kawamurai* male 4 call 6 | 0.109 | 0.266 | 3.759 | 0.157 | 9.000 | 86.022 | 1292.000 | 0.011 |
| *Fejervarya kawamurai* male 4 call 7 | 0.112 | 0.302 | 3.311 | 0.190 | 10.000 | 86.538 | 1292.000 | 0.011 |
| *Fejervarya kawamurai* male 4 call 8 | 0.109 | 0.304 | 3.289 | 0.195 | 9.000 | 86.022 | 1292.000 | 0.012 |
| *Fejervarya kawamurai* male 4 call 9 | 0.111 | 0.280 | 3.571 | 0.169 | 10.000 | 87.379 | 1292.000 | 0.012 |
| *Fejervarya kawamurai* male 4 call 10 | 0.115 | 0.349 | 2.865 | 0.234 | 10.000 | 84.112 | 1292.000 | 0.012 |
| *Fejervarya kawamurai* male 5 call 1 | 0.092 | 0.336 | 2.976 | 0.244 | 10.000 | 129.870 | 3014.600 | 0.008 |
| *Fejervarya kawamurai* male 5 call 2 | 0.095 | 0.243 | 4.115 | 0.147 | 8.000 | 100.000 | 1378.100 | 0.011 |
| *Fejervarya kawamurai* male 5 call 3 | 0.084 | 0.245 | 4.082 | 0.160 | 9.000 | 108.434 | 1464.300 | 0.011 |
| *Fejervarya kawamurai* male 5 call 4 | 0.125 | 0.233 | 4.292 | 0.131 | 12.000 | 114.286 | 1421.200 | 0.011 |
| *Fejervarya kawamurai* male 5 call 5 | 0.098 | 0.251 | 3.984 | 0.148 | 9.000 | 123.288 | 1421.200 | 0.012 |
| *Fejervarya kawamurai* male 5 call 6 | 0.083 | 0.243 | 4.115 | 0.156 | 8.000 | 123.077 | 3014.600 | 0.011 |
| *Fejervarya kawamurai* male 5 call 7 | 0.112 | 0.248 | 4.032 | 0.143 | 10.000 | 107.527 | 3057.700 | 0.012 |
| *Fejervarya kawamurai* male 5 call 8 | 0.094 | 0.244 | 4.098 | 0.152 | 11.000 | 90.909 | 1335.100 | 0.012 |
| *Fejervarya kawamurai* male 5 call 9 | 0.125 | 0.263 | 3.802 | 0.138 | 12.000 | 117.647 | 1464.300 | 0.011 |
| *Fejervarya kawamurai* male 5 call 10 | 0.116 | 0.243 | 4.115 | 0.127 | 9.000 | 97.826 | 1464.300 | 0.011 |
| *Fejervarya kawamurai* male 6 call 1 | 0.109 | 0.234 | 4.274 | 0.125 | 11.000 | 105.769 | 3057.700 | 0.011 |
| *Fejervarya kawamurai* male 6 call 2 | 0.112 | 0.233 | 4.292 | 0.121 | 10.000 | 96.154 | 3057.700 | 0.013 |
| *Fejervarya kawamurai* male 6 call 3 | 0.106 | 0.247 | 4.049 | 0.141 | 9.000 | 103.448 | 1378.100 | 0.007 |
| *Fejervarya kawamurai* male 6 call 4 | 0.112 | 0.255 | 3.922 | 0.143 | 9.000 | 96.774 | 3100.800 | 0.007 |
| *Fejervarya kawamurai* male 6 call 5 | 0.110 | 0.256 | 3.906 | 0.146 | 10.000 | 104.167 | 3014.600 | 0.011 |
| *Fejervarya kawamurai* male 6 call 6 | 0.110 | 0.259 | 3.861 | 0.149 | 10.000 | 101.010 | 1378.100 | 0.011 |
| *Fejervarya kawamurai* male 6 call 7 | 0.109 | 0.383 | 2.611 | 0.274 | 9.000 | 90.909 | 3057.700 | 0.008 |
| *Fejervarya kawamurai* male 6 call 8 | 0.100 | 0.375 | 2.667 | 0.275 | 9.000 | 101.124 | 2971.600 | 0.010 |
| *Fejervarya kawamurai* male 6 call 9 | 0.094 | 0.276 | 3.623 | 0.182 | 9.000 | 103.448 | 2971.600 | 0.011 |
| *Fejervarya kawamurai* male 6 call 10 | 0.096 | 0.266 | 3.759 | 0.170 | 9.000 | 116.883 | 2971.600 | 0.013 |

**S3 Table. Verified voucher information and origin of specimens used in species delimitation analyses.** For each specimen obtained from primarily from GenBank, we provide taxon label, voucher code (when available), locality and coordinates, GenBank accession number(s), and reference. This table ensures traceability of samples and supports the validation of taxonomic assignments in our multilocus and genome-wide framework.

| **Species assignment of Model A1** | **Taxon label in GenBank** | **Voucher** | **Locality** | **Lat** | **Lon** | **Country** | **Voucher status** | **Notes on identity** | **Source** |
| --- | --- | --- | --- | --- | --- | --- | --- | --- | --- |
| *Fejervarya kawamurai* (Clade II) | *Fejervarya limnocharis* | HNFL01 | Fuluo, Hainan Island | 18.5988 | 108.7235 | China | Verified | Labelled as *F. limnocharis* in GenBank, but phylogenetically clusters within *F. kawamurai* [Clade II] | Yang et al. (2022) |
| *Fejervarya kawamurai* (Clade II) | *Fejervarya limnocharis* | HNFL02 | Fuluo, Hainan Island | 18.5988 | 108.7235 | China | Verified | Labelled as *F. limnocharis* in GenBank, but phylogenetically clusters within *F. kawamurai* [Clade II] | Yang et al. (2022) |
| *Fejervarya kawamurai* (Clade II) | *Fejervarya limnocharis* | HNFL03 | Fuluo, Hainan Island | 18.5988 | 108.7235 | China | Verified | Labelled as *F. limnocharis* in GenBank, but phylogenetically clusters within *F. kawamurai* [Clade II] | Yang et al. (2022) |
| *Fejervarya kawamurai* (Clade II) | *Fejervarya limnocharis* | HNLY01 | Luoyang, Henan | 34.6196 | 112.4541 | China | Verified | Labelled as *F. limnocharis* in GenBank, but phylogenetically clusters within *F. kawamurai* [Clade II] | Yang et al. (2022) |
| *Fejervarya kawamurai* (Clade II) | *Fejervarya limnocharis* | HNXY05 | Xinyang, Henan | 32.1649 | 114.0911 | China | Verified | Labelled as *F. limnocharis* in GenBank, but phylogenetically clusters within *F. kawamurai* [Clade II] | Yang et al. (2022) |
| *Fejervarya kawamurai* (Clade II) | *Fejervarya limnocharis* | SHLC15 | Luchao, Shanghai | 30.8818 | 121.8359 | China | Verified | Labelled as *F. limnocharis* in GenBank, but phylogenetically clusters within *F. kawamurai* [Clade II] | Yang et al. (2022) |
| *Fejervarya kawamurai* (Clade II) | *Fejervarya limnocharis* | ZJDQ05 | Hangzhou, Zhejiang | 30.46 | 120.45 | China | Verified | Labelled as *F. limnocharis* in GenBank, but phylogenetically clusters within *F. kawamurai* [Clade II] | Yang et al. (2022) |
| *Fejervarya kawamurai* (Clade II) | *Fejervarya limnocharis* | ZJDQ09 | Hangzhou, Zhejiang | 30.46 | 120.45 | China | Verified | Labelled as *F. limnocharis* in GenBank, but phylogenetically clusters within *F. kawamurai* [Clade II] | Yang et al. (2022) |
| *Fejervarya kawamurai* (Clade II) | *Fejervarya limnocharis* | ZJJX04 | Hangzhou, Zhejiang | 30.46 | 120.45 | China | Verified | Labelled as *F. limnocharis* in GenBank, but phylogenetically clusters within *F. kawamurai* [Clade II] | Yang et al. (2022) |
| *Fejervarya kawamurai* (Clade II) | *Fejervarya limnocharis* | ZJJX10 | Hangzhou, Zhejiang | 30.46 | 120.45 | China | Verified | Labelled as *F. limnocharis* in GenBank, but phylogenetically clusters within *F. kawamurai* [Clade II] | Yang et al. (2022) |
| *Fejervarya kawamurai* (Clade II) | *Fejervarya limnocharis* | ZJLX05 | Jinhua, Zhejiang | 29.3642 | 119.8111 | China | Verified | Labelled as *F. limnocharis* in GenBank, but phylogenetically clusters within *F. kawamurai* [Clade II] | Yang et al. (2022) |
| *Fejervarya kawamurai* (Clade II) | *Fejervarya limnocharis* | ZJLX06 | Jinhua, Zhejiang | 29.3642 | 119.8111 | China | Verified | Labelled as *F. limnocharis* in GenBank, but phylogenetically clusters within *F. kawamurai* [Clade II] | Yang et al. (2022) |
| *Fejervarya kawamurai* (Clade II) | *Fejervarya limnocharis* | ZJLX08 | Jinhua, Zhejiang | 29.3642 | 119.8111 | China | Verified | Labelled as *F. limnocharis* in GenBank, but phylogenetically clusters within *F. kawamurai* [Clade II] | Yang et al. (2022) |
| *Fejervarya kawamurai* (Clade II) | *Fejervarya limnocharis* | ZJLX09 | Jinhua, Zhejiang | 29.3642 | 119.8111 | China | Verified | Labelled as *F. limnocharis* in GenBank, but phylogenetically clusters within *F. kawamurai* [Clade II] | Yang et al. (2022) |
| *Fejervarya kawamurai* (Clade II) | *Fejervarya limnocharis* | ZJLX10 | Jinhua, Zhejiang | 29.3642 | 119.8111 | China | Verified | Labelled as *F. limnocharis* in GenBank, but phylogenetically clusters within *F. kawamurai* [Clade II] | Yang et al. (2022) |
| *Fejervarya kawamurai* (Clade II) | *Fejervarya limnocharis* | ZJLX12 | Jinhua, Zhejiang | 29.3642 | 119.8111 | China | Verified | Labelled as *F. limnocharis* in GenBank, but phylogenetically clusters within *F. kawamurai* [Clade II] | Yang et al. (2022) |
| *Fejervarya kawamurai* (Clade II) | *Fejervarya limnocharis* | ZJLX14 | Jinhua, Zhejiang | 29.3642 | 119.8111 | China | Verified | Labelled as *F. limnocharis* in GenBank, but phylogenetically clusters within *F. kawamurai* [Clade II] | Yang et al. (2022) |
| *Fejervarya kawamurai* (Clade II) | *Fejervarya limnocharis* | ZJLX15 | Jinhua, Zhejiang | 29.3642 | 119.8111 | China | Verified | Labelled as *F. limnocharis* in GenBank, but phylogenetically clusters within *F. kawamurai* [Clade II] | Yang et al. (2022) |
| *Fejervarya kawamurai* (Clade II) | *Fejervarya limnocharis* | ZJNB04 | Beilun, Zhejiang | 29.8999 | 121.8719 | China | Verified | Labelled as *F. limnocharis* in GenBank, but phylogenetically clusters within *F. kawamurai* [Clade II] | Yang et al. (2022) |
| *Fejervarya kawamurai* (Clade II) | *Fejervarya limnocharis* | ZJPH02 | Pinghu, Zhejiang | 30.6396 | 121.0488 | China | Verified | Labelled as *F. limnocharis* in GenBank, but phylogenetically clusters within *F. kawamurai* [Clade II] | Yang et al. (2022) |
| *Fejervarya kawamurai* (Clade II) | *Fejervarya limnocharis* | ZJPH03 | Pinghu, Zhejiang | 30.6396 | 121.0488 | China | Verified | Labelled as *F. limnocharis* in GenBank, but phylogenetically clusters within *F. kawamurai* [Clade II] | Yang et al. (2022) |
| *Fejervarya kawamurai* (Clade II) | *Fejervarya limnocharis* | ZJPH04 | Pinghu, Zhejiang | 30.6396 | 121.0488 | China | Verified | Labelled as *F. limnocharis* in GenBank, but phylogenetically clusters within *F. kawamurai* [Clade II] | Yang et al. (2022) |
| *Fejervarya kawamurai* (Clade II) | *Fejervarya limnocharis* | ZJPH05 | Pinghu, Zhejiang | 30.6396 | 121.0488 | China | Verified | Labelled as *F. limnocharis* in GenBank, but phylogenetically clusters within *F. kawamurai* [Clade II] | Yang et al. (2022) |
| *Fejervarya kawamurai* (Clade II) | *Fejervarya limnocharis* | ZJPJ01 | Jinhua, Zhejiang | 29.4847 | 120.0118 | China | Verified | Labelled as *F. limnocharis* in GenBank, but phylogenetically clusters within *F. kawamurai* [Clade II] | Yang et al. (2022) |
| *Fejervarya kawamurai* (Clade II) | *Fejervarya limnocharis* | ZJPJ02 | Jinhua, Zhejiang | 29.4847 | 120.0118 | China | Verified | Labelled as *F. limnocharis* in GenBank, but phylogenetically clusters within *F. kawamurai* [Clade II] | Yang et al. (2022) |
| *Fejervarya kawamurai* (Clade II) | *Fejervarya limnocharis* | ZJPJ03 | Jinhua, Zhejiang | 29.4847 | 120.0118 | China | Verified | Labelled as *F. limnocharis* in GenBank, but phylogenetically clusters within *F. kawamurai* [Clade II] | Yang et al. (2022) |
| *Fejervarya kawamurai* (Clade II) | *Fejervarya limnocharis* | ZJPJ04 | Jinhua, Zhejiang | 29.4847 | 120.0118 | China | Verified | Labelled as *F. limnocharis* in GenBank, but phylogenetically clusters within *F. kawamurai* [Clade II] | Yang et al. (2022) |
| *Fejervarya kawamurai* (Clade II) | *Fejervarya limnocharis* | ZJPJ05 | Jinhua, Zhejiang | 29.4847 | 120.0118 | China | Verified | Labelled as *F. limnocharis* in GenBank, but phylogenetically clusters within *F. kawamurai* [Clade II] | Yang et al. (2022) |
| *Fejervarya kawamurai* (Clade II) | *Fejervarya limnocharis* | ZJPJ09 | Jinhua, Zhejiang | 29.4847 | 120.0118 | China | Verified | Labelled as *F. limnocharis* in GenBank, but phylogenetically clusters within *F. kawamurai* [Clade II] | Yang et al. (2022) |
| *Fejervarya kawamurai* (Clade II) | *Fejervarya limnocharis* | ZJPJ11 | Jinhua, Zhejiang | 29.4847 | 120.0118 | China | Verified | Labelled as *F. limnocharis* in GenBank, but phylogenetically clusters within *F. kawamurai* [Clade II] | Yang et al. (2022) |
| *Fejervarya kawamurai* (Clade II) | *Fejervarya limnocharis* | ZJPJ15 | Jinhua, Zhejiang | 29.4847 | 120.0118 | China | Verified | Labelled as *F. limnocharis* in GenBank, but phylogenetically clusters within *F. kawamurai* [Clade II] | Yang et al. (2022) |
| *Fejervarya kawamurai* (Clade II) | *Fejervarya limnocharis* | ZSHX06 | Huangxing, Zhoushan | 30.1989 | 122.6412 | China | Verified | Labelled as *F. limnocharis* in GenBank, but phylogenetically clusters within *F. kawamurai* [Clade II] | Yang et al. (2022) |
| *Fejervarya kawamurai* (Clade II) | *Fejervarya limnocharis* | ZSKZ08 | Kuzhu,Zhoushan | 29.9928 | 122.2436 | China | Verified | Labelled as *F. limnocharis* in GenBank, but phylogenetically clusters within *F. kawamurai* [Clade II] | Yang et al. (2022) |
| *Fejervarya kawamurai* (Clade I) | *Fejervarya limnocharis* | GZBY01 | Guangzhou | 23.2857 | 113.3058 | China | Verified | Labelled as *F. limnocharis* in GenBank, but phylogenetically clusters within *F. kawamurai* [Clade II] | Yang et al. (2022) |
| *Fejervarya kawamurai* (Clade I) | *Fejervarya limnocharis* | GZBY02 | Guangzhou | 23.2857 | 113.3058 | China | Verified | Labelled as *F. limnocharis* in GenBank, but phylogenetically clusters within *F. kawamurai* [Clade II] | Yang et al. (2022) |
| *Fejervarya kawamurai* (Clade I) | *Fejervarya limnocharis* | GZBY04 | Guangzhou | 23.2857 | 113.3058 | China | Verified | Labelled as *F. limnocharis* in GenBank, but phylogenetically clusters within *F. kawamurai* [Clade II] | Yang et al. (2022) |
| *Fejervarya kawamurai* (Clade I) | *Fejervarya limnocharis* | GZBY05 | Guangzhou | 23.2857 | 113.3058 | China | Verified | Labelled as *F. limnocharis* in GenBank, but phylogenetically clusters within *F. kawamurai* [Clade II] | Yang et al. (2022) |
| *Fejervarya kawamurai* (Clade I) | *Fejervarya limnocharis* | HNXY05 | Shanghai | 32.1469 | 114.091 | China | Verified | Labelled as *F. limnocharis* in GenBank, but phylogenetically clusters within *F. kawamurai* [Clade II] | Yang et al. (2022) |
| *Fejervarya kawamurai* (Clade I) | *Fejervarya limnocharis* | ZJPH12 | Shaoxing, Zhejiang | 30.6396 | 121.0488 | China | Verified | Labelled as *F. limnocharis* in GenBank, but phylogenetically clusters within *F. kawamurai* [Clade II] | Yang et al. (2022) |
| *Fejervarya kawamurai* (Clade I) | *Fejervarya limnocharis* | ZSBQ11 | Baiquan, Zhoushan | 30.0567 | 122.1578 | China | Verified | Labelled as *F. limnocharis* in GenBank, but phylogenetically clusters within *F. kawamurai* [Clade II] | Yang et al. (2022) |
| *Fejervarya kawamurai* (Clade I) | *Fejervarya limnocharis* | ZSDD03 | Daidong, Zhoushan | 30.2902 | 122.1947 | China | Verified | Labelled as *F. limnocharis* in GenBank, but phylogenetically clusters within *F. kawamurai* [Clade II] | Yang et al. (2022) |
| *Fejervarya kawamurai* (Clade I) | *Fejervarya limnocharis* | ZSDF01 | Dongfu, Zhoushan | 30.1442 | 122.7575 | China | Verified | Labelled as *F. limnocharis* in GenBank, but phylogenetically clusters within *F. kawamurai* [Clade II] | Yang et al. (2022) |
| *Fejervarya kawamurai* (Clade I) | *Fejervarya limnocharis* | ZSDF03 | Dongfu, Zhoushan | 30.1442 | 122.7575 | China | Verified | Labelled as *F. limnocharis* in GenBank, but phylogenetically clusters within *F. kawamurai* [Clade II] | Yang et al. (2022) |
| *Fejervarya kawamurai* (Clade I) | *Fejervarya limnocharis* | ZSDH02 | Donghai, Zhoushan | 30.7083 | 122.4992 | China | Verified | Labelled as *F. limnocharis* in GenBank, but phylogenetically clusters within *F. kawamurai* [Clade II] | Yang et al. (2022) |
| *Fejervarya kawamurai* (Clade I) | *Fejervarya limnocharis* | ZSDJ12 | Daijia, Zhoushan | 30.0198 | 122.0332 | China | Verified | Labelled as *F. limnocharis* in GenBank, but phylogenetically clusters within *F. kawamurai* [Clade II] | Yang et al. (2022) |
| *Fejervarya kawamurai* (Clade I) | *Fejervarya limnocharis* | ZSGJ01 | Shaoxing, Zhejiang | 29.8833 | 121.9823 | China | Verified | Labelled as *F. limnocharis* in GenBank, but phylogenetically clusters within *F. kawamurai* [Clade II] | Yang et al. (2022) |
| *Fejervarya kawamurai* (Clade I) | *Fejervarya limnocharis* | ZSGL04 | Ganlan,Zhoushan | 30.1121 | 122.1305 | China | Verified | Labelled as *F. limnocharis* in GenBank, but phylogenetically clusters within *F. kawamurai* [Clade II] | Yang et al. (2022) |
| *Fejervarya kawamurai* (Clade I) | *Fejervarya limnocharis* | ZSGQ06 | Gouqi, Zhoushan | 30.7162 | 122.7724 | China | Verified | Labelled as *F. limnocharis* in GenBank, but phylogenetically clusters within *F. kawamurai* [Clade II] | Yang et al. (2022) |
| *Fejervarya kawamurai* (Clade I) | *Fejervarya limnocharis* | ZSHL03 | Huanglong, Zhoushan | 30.728 | 122.8146 | China | Verified | Labelled as *F. limnocharis* in GenBank, but phylogenetically clusters within *F. kawamurai* [Clade II] | Yang et al. (2022) |
| *Fejervarya kawamurai* (Clade I) | *Fejervarya limnocharis* | ZSHN01 | Huaniao, Zhoushan | 30.8474 | 122.6917 | China | Verified | Labelled as *F. limnocharis* in GenBank, but phylogenetically clusters within *F. kawamurai* [Clade II] | Yang et al. (2022) |
| *Fejervarya kawamurai* (Clade I) | *Fejervarya limnocharis* | ZSHX06 | Huangxing, Zhoushan | 30.1989 | 122.6412 | China | Verified | Labelled as *F. limnocharis* in GenBank, but phylogenetically clusters within *F. kawamurai* [Clade II] | Yang et al. (2022) |
| *Fejervarya kawamurai* (Clade I) | *Fejervarya limnocharis* | ZSJH01 | Jihu, Zhoushan | 30.7182 | 122.4624 | China | Verified | Labelled as *F. limnocharis* in GenBank, but phylogenetically clusters within *F. kawamurai* [Clade II] | Yang et al. (2022) |
| *Fejervarya kawamurai* (Clade I) | *Fejervarya limnocharis* | ZSKZ08 | Kuzhu, Zhoushan | 29.9928 | 122.2436 | China | Verified | Labelled as *F. limnocharis* in GenBank, but phylogenetically clusters within *F. kawamurai* [Clade II] | Yang et al. (2022) |
| *Fejervarya kawamurai* (Clade I) | *Fejervarya limnocharis* | ZSMZH01 | Miaozihu, Zhoushan | 30.1976 | 122.6792 | China | Verified | Labelled as *F. limnocharis* in GenBank, but phylogenetically clusters within *F. kawamurai* [Clade II] | Yang et al. (2022) |
| *Fejervarya kawamurai* (Clade I) | *Fejervarya limnocharis* | ZSMZH02 | Miaozihu, Zhoushan | 30.1976 | 122.6792 | China | Verified | Labelled as *F. limnocharis* in GenBank, but phylogenetically clusters within *F. kawamurai* [Clade II] | Yang et al. (2022) |
| *Fejervarya kawamurai* (Clade I) | *Fejervarya limnocharis* | ZSMZH03 | Miaozihu, Zhoushan | 30.1976 | 122.6792 | China | Verified | Labelled as *F. limnocharis* in GenBank, but phylogenetically clusters within *F. kawamurai* [Clade II] | Yang et al. (2022) |
| *Fejervarya kawamurai* (Clade I) | *Fejervarya limnocharis* | ZSNP03 | Nanpu, Zhoushan | 30.2667 | 122.1385 | China | Verified | Labelled as *F. limnocharis* in GenBank, but phylogenetically clusters within *F. kawamurai* [Clade II] | Yang et al. (2022) |
| *Fejervarya kawamurai* (Clade I) | *Fejervarya limnocharis* | ZSQB04 | Qingbang, Zhoushan | 30.1995 | 122.7069 | China | Verified | Labelled as *F. limnocharis* in GenBank, but phylogenetically clusters within *F. kawamurai* [Clade II] | Yang et al. (2022) |
| *Fejervarya kawamurai* (Clade I) | *Fejervarya limnocharis* | ZSQS02 | Qushan, Zhoushan, | 30.4371 | 122.3516 | China | Verified | Labelled as *F. limnocharis* in GenBank, but phylogenetically clusters within *F. kawamurai* [Clade II] | Yang et al. (2022) |
| *Fejervarya kawamurai* (Clade I) | *Fejervarya limnocharis* | ZSSN05 | Shengshan, Zhoushan | 30.7239 | 122.811 | China | Verified | Labelled as *F. limnocharis* in GenBank, but phylogenetically clusters within *F. kawamurai* [Clade II] | Yang et al. (2022) |
| *Fejervarya kawamurai* (Clade I) | *Fejervarya limnocharis* | ZSSN09 | Sannong, Zhoushan | 30.4454 | 122.2938 | China | Verified | Labelled as *F. limnocharis* in GenBank, but phylogenetically clusters within *F. kawamurai* [Clade II] | Yang et al. (2022) |
| *Fejervarya kawamurai* (Clade I) | *Fejervarya limnocharis* | ZSSS05 | Sannong, Zhoushan | 30.4454 | 122.2938 | China | Verified | Labelled as *F. limnocharis* in GenBank, but phylogenetically clusters within *F. kawamurai* [Clade II] | Yang et al. (2022) |
| *Fejervarya kawamurai* (Clade I) | *Fejervarya limnocharis* | ZSTT06 | Tiantu,Zhoushan | 30.4386 | 122.41 | China | Verified | Labelled as *F. limnocharis* in GenBank, but phylogenetically clusters within *F. kawamurai* [Clade II] | Yang et al. (2022) |
| *Fejervarya kawamurai* (Clade I) | *Fejervarya limnocharis* | ZSXYS01 | Xiaoyangshan,Zhoushan | 30.6411 | 122.0611 | China | Verified | Labelled as *F. limnocharis* in GenBank, but phylogenetically clusters within *F. kawamurai* [Clade II] | Yang et al. (2022) |
| *Fejervarya kawamurai* (Clade I) | *Fejervarya limnocharis* | ZSYD09 | Yandun,Zhoushan | 30.136 | 122.0198 | China | Verified | Labelled as *F. limnocharis* in GenBank, but phylogenetically clusters within *F. kawamurai* [Clade II] | Yang et al. (2022) |
| *Fejervarya kawamurai* (Clade I) | *Fejervarya limnocharis* | ZSZM14 | Zhanmao,Zhoushan | 30.0476 | 122.2564 | China | Verified | Labelled as *F. limnocharis* in GenBank, but phylogenetically clusters within *F. kawamurai* [Clade II] | Yang et al. (2022) |
| *Fejervarya kawamurai* (Clade I) | *Fejervarya kawamurai* | Full mtDNA | Zunyi city, Guizhou | 27.7083 | 106.92 | China | Verified | Consistent with current labelling, *F. kawamurai,* but clustered with Clade II | Cheng et al. (2014) |
| *Fejervarya kawamurai* (Clade II) | *Fejervarya limnocharis* | ZJLX05 | Jinhua, Zhejiang | 29.3642 | 119.8111 | China | Verified | Labelled as *F. limnocharis* in GenBank, but phylogenetically clusters within *F. kawamurai* [Clade II] | Yang et al. (2022) |
| *Fejervarya kawamurai* (Clade II) | *Fejervarya limnocharis* | ZJLX06 | Zhejiang, China | 29.3642 | 119.8111 | China | Verified | Labelled as *F. limnocharis* in GenBank, but phylogenetically clusters within *F. kawamurai* [Clade II] | Yang et al. (2022) |
| *Fejervarya kawamurai* (Clade II) | *Fejervarya limnocharis* | ZJLX08 | Zhejiang, China | 29.3642 | 119.8111 | China | Verified | Labelled as *F. limnocharis* in GenBank, but phylogenetically clusters within *F. kawamurai* [Clade II] | Yang et al. (2022) |
| *Fejervarya kawamurai* (Clade II) | *Fejervarya limnocharis* | ZJLX09 | Zhejiang, China | 29.3642 | 119.8111 | China | Verified | Labelled as *F. limnocharis* in GenBank, but phylogenetically clusters within *F. kawamurai* [Clade II] | Yang et al. (2022) |
| *Fejervarya kawamurai* (Clade II) | *Fejervarya limnocharis* | ZJLX12 | Zhejiang, China | 29.3642 | 119.8111 | China | Verified | Labelled as *F. limnocharis* in GenBank, but phylogenetically clusters within *F. kawamurai* [Clade II] | Yang et al. (2022) |
| *Fejervarya kawamurai* (Clade II) | *Fejervarya limnocharis* | ZJLX14 | Zhejiang, China | 29.3642 | 119.8111 | China | Verified | Labelled as *F. limnocharis* in GenBank, but phylogenetically clusters within *F. kawamurai* [Clade II] | Yang et al. (2022) |
| *Fejervarya kawamurai* (Clade II) | *Fejervarya limnocharis* | ZJLX15 | Zhejiang, China | 29.3642 | 119.8111 | China | Verified | Labelled as *F. limnocharis* in GenBank, but phylogenetically clusters within *F. kawamurai* [Clade II] | Yang et al. (2022) |
| *Fejervarya kawamurai* (Clade II) | *Fejervarya limnocharis* | ZJPH02 | Zhejiang, China | 30.6396 | 121.0488 | China | Verified | Labelled as *F. limnocharis* in GenBank, but phylogenetically clusters within *F. kawamurai* [Clade II] | Yang et al. (2022) |
| *Fejervarya kawamurai* (Clade II) | *Fejervarya limnocharis* | ZJPH03 | Zhejiang, China | 30.6396 | 121.0488 | China | Verified | Labelled as *F. limnocharis* in GenBank, but phylogenetically clusters within *F. kawamurai* [Clade II] | Yang et al. (2022) |
| *Fejervarya kawamurai* (Clade II) | *Fejervarya limnocharis* | ZJPH04 | Zhejiang, China | 30.6396 | 121.0488 | China | Verified | Labelled as *F. limnocharis* in GenBank, but phylogenetically clusters within *F. kawamurai* [Clade II] | Yang et al. (2022) |
| *Fejervarya kawamurai* (Clade II) | *Fejervarya limnocharis* | ZJPH05 | Zhejiang, China | 30.6396 | 121.0488 | China | Verified | Labelled as *F. limnocharis* in GenBank, but phylogenetically clusters within *F. kawamurai* [Clade II] | Yang et al. (2022) |
| *Fejervarya sakishimensis* | *Fejervarya sakishimensis* | Jp1 | Iriomote Island, Japan |  |  | Japan | Verified | GPS is not available but vouchers were cross-checked against their original published sources | Kotaki et al. (2010) |
| *Fejervarya limnocharis* (Clade II) | *Fejervarya limnocharis* | CAS 235484 | Krung Thep Mahanakhon (Bangkok) |  |  | Thailand | Verified | GPS is not available but vouchers were cross-checked against their original published sources | Koehler et al. (2019) |
| *Fejervarya limnocharis* (Clade II) | *Fejervarya limnocharis* | CAS 215116 | Shanjiang village, just S of Liuku, Yunnan |  |  | China | Verified | GPS is not available but vouchers were cross-checked against their original published sources | Koehler et al. (2019) |
| *Fejervarya limnocharis* (Clade II) | *Fejervarya limnocharis* | CAS 228131 | Shanjiang village, just S of Liuku, Yunnan |  |  | China | Verified | GPS is not available but vouchers were cross-checked against their original published sources | Koehler et al. (2019) |
| *Fejervarya limnocharis* (Clade II) | *Fejervarya limnocharis* | CAS 228129 | Liuku bridge, Yunnan |  |  | China | Verified | GPS is not available but vouchers were cross-checked against their original published sources | Koehler et al. (2019) |
| *Fejervarya limnocharis* (Clade II) | *Fejervarya limnocharis* | CAS 230367 | Township, Pidaung Wildlife Sanctuary, near Ma-Li-Hka Village | |  | Myanmar | Verified | GPS is not available but vouchers were cross-checked against their original published sources | Koehler et al. (2019) |
| *Fejervarya limnocharis* (Clade II) | *Fejervarya limnocharis* | CAS 234814 | Chin: Kanpatlat Township, in Kanpatlat |  |  | Myanmar | Verified | GPS is not available but vouchers were cross-checked against their original published sources | Koehler et al. (2019) |
| *Fejervarya limnocharis* (Clade II) | *Fejervarya limnocharis* | CAS 234815 | Chin: Kanpatlat Township, in Kanpatlat |  |  | Myanmar | Verified | GPS is not available but vouchers were cross-checked against their original published sources | Koehler et al. (2019) |
| *Fejervarya limnocharis* (Clade II) | *Fejervarya limnocharis* | CAS 235276 | Kanpatlat Township, Let Mon Village |  |  | Myanmar | Verified | GPS is not available but vouchers were cross-checked against their original published sources | Kotaki et al. (2010) |
| *Fejervarya limnocharis* (Clade II) | *Fejervarya limnocharis* | SMF 104127 | Mae Hong Son, Pangmapha, Ban Soppong |  |  | Thailand | Verified | GPS is not available but vouchers were cross-checked against their original published sources | Kotaki et al. (2010) |
| *Fejervarya limnocharis* (Clade II) | *Fejervarya limnocharis* | Thai-1 | Ranong |  |  | Thailand | Verified | GPS is not available but vouchers were cross-checked against their original published sources | Kotaki et al. (2010) |
| *Fejervarya limnocharis* (Clade II) | *Fejervarya limnocharis* | Thai-2 | Tha Ton |  |  | Thailand | Verified | GPS is not available but vouchers were cross-checked against their original published sources | Kotaki et al. (2010) |
| *Fejervarya limnocharis* (Clade II) | *Fejervarya limnocharis* | Thai-3 | Mae Hong Son |  |  | Thailand | Verified | GPS is not available but vouchers were cross-checked against their original published sources | Koehler et al. (2019) |
| *Fejervarya limnocharis* (Clade II) | *Fejervarya limnocharis* | Thai-4 | Nakhon Si Thammarat |  |  | Thailand | Verified | GPS is not available but vouchers were cross-checked against their original published sources | Koehler et al. (2019) |
| *Fejervarya limnocharis* (Clade II) | *Fejervarya limnocharis* | SMF 104128 | Mae Hong Son, Pangmapha, Ban Soppong |  |  | Thailand | Verified | GPS is not available but vouchers were cross-checked against their original published sources | Koehler et al. (2019) |
| *Fejervarya limnocharis* (Clade II) | *Fejervarya limnocharis* | SMF 104131 | Pangmapha, near Ban Nam Rin, Thailand |  |  | Thailand | Verified | GPS is not available but vouchers were cross-checked against their original published sources | Koehler et al. (2019) |
| *Fejervarya limnocharis* (Clade II) | *Fejervarya limnocharis* | SMF 105119 | Ridge north of viewpoint between Pangmapha and Pai, Thailand |  |  | Thailand | Verified | GPS is not available but vouchers were cross-checked against their original published sources | Ziegler et al. (2000) |
| *Fejervarya limnocharis* (Clade II) | *Fejervarya limnocharis* | SMF 105120 | Ridge north of viewpoint between Pangmapha and Pai, Thailand |  |  | Thailand | Verified | GPS is not available but vouchers were cross-checked against their original published sources | Ziegler et al. (2000) |
| *Fejervarya limnocharis* (Clade II) | *Fejervarya limnocharis* | SMF 105121 | Pangmapha district, at NamLang River, Thailand |  |  | Thailand | Verified | GPS is not available but vouchers were cross-checked against their original published sources | Miller et al. (2022), unpublished |
| *Fejervarya limnocharis* (Clade II) | *Fejervarya limnocharis* | TZ25 | North Vietnam |  |  | Vietnam | Verified | GPS is not available but vouchers were cross-checked against their original published sources | Grosjean et al. (2015) |
| *Fejervarya limnocharis* (Clade II) | *Fejervarya limnocharis* | TZ98105 | North Vietnam |  |  | Vietnam | Verified | GPS is not available but vouchers were cross-checked against their original published sources | Grosjean et al. (2015) |
| *Fejervarya limnocharis* (Clade II) | *Fejervarya limnocharis* |  | Northern Karsh forest, Vietnam |  |  | Vietnam | Verified | GPS is not available but vouchers were cross-checked against their original published sources | Grosjean et al. (2015) |
| *Fejervarya limnocharis* (Clade II) | *Fejervarya limnocharis* | 1999.5721 | Lao Cai, Sapa | 22.3167 | 103.7833 | Vietnam | Verified | Location and vouchers were cross-checked against their original published sources | Grosjean et al. (2015) |
| *Fejervarya limnocharis* (Clade II) | *Fejervarya limnocharis* | 2004.0208 | Phongsali, Buon Tai, Laos | 21.3833 | 101.9667 | Laos | Verified | Location and vouchers were cross-checked against their original published sources | Grosjean et al. (2015) |
| *Fejervarya limnocharis* (Clade II) | *Fejervarya limnocharis* | 2004.0309 | Phongsali, Nathen, Laos | 21.4 | 101.9667 | Laos | Verified | Location and vouchers were cross-checked against their original published sources | Grosjean et al. (2015) |
| *Fejervarya limnocharis* (Clade II) | *Fejervarya limnocharis* | 2006.2411 | Louangphrabang, Ban Sop Khao |  |  | Laos | Verified | Location and vouchers were cross-checked against their original published sources | Grosjean et al. (2015) |
| *Fejervarya limnocharis* (Clade II) | *Fejervarya limnocharis* | K3037 | Chiang Mai, Doi Chiang Dao |  |  | Thailand | Verified | Location and vouchers were cross-checked against their original published sources | Grosjean et al. (2015) |
| *Fejervarya limnocharis* (Clade II) | *Fejervarya limnocharis* | K3085 | Louangphrabang, Ban Sop Khao |  |  | Laos | Verified | Location and vouchers were cross-checked against their original published sources | Grosjean et al. (2015) |
| *Fejervarya kawamurai* (Clade II) | *Fejervarya multistriata* | FM_HN_01 | Northern Chinese Mainland |  |  | China | Verified | Precise locality unavailable, broadly assigned as Northern China (likely mainland), from original source of depositor | Wang et al. (2016), direct submission |
| *Fejervarya kawamurai* (Clade II) | *Fejervarya multistriata* | FM_HN_02 | Northern Chinese Mainland |  |  | China | Verified | Precise locality unavailable, broadly assigned as Northern China (likely mainland), from original source of depositor | Wang et al. (2016), direct submission |
| *Fejervarya kawamurai* (Clade II) | *Fejervarya multistriata* | FM_HN_03 | Northern Chinese Mainland |  |  | China | Verified | Precise locality unavailable, broadly assigned as Northern China (likely mainland), from original source of depositor | Wang et al. (2016), direct submission |
| *Fejervarya kawamurai* (Clade II) | *Fejervarya multistriata* | FM_HN_04 | Northern Chinese Mainland |  |  | China | Verified | Precise locality unavailable, broadly assigned as Northern China (likely mainland), from original source of depositor | Wang et al. (2016), direct submission |
| *Fejervarya kawamurai* (Clade II) | *Fejervarya multistriata* | FM_HN_05 | Northern Chinese Mainland |  |  | China | Verified | Precise locality unavailable, broadly assigned as Northern China (likely mainland), from original source of depositor | Wang et al. (2016), direct submission |
| *Fejervarya kawamurai* (Clade II) | *Fejervarya multistriata* | FM_HN_06 | Northern Chinese Mainland |  |  | China | Verified | Precise locality unavailable, broadly assigned as Northern China (likely mainland), from original source of depositor | Wang et al. (2016), direct submission |
| *Fejervarya kawamurai* (Clade II) | *Fejervarya multistriata* | FM_HN_07 | Northern Chinese Mainland |  |  | China | Verified | Precise locality unavailable, broadly assigned as Northern China (likely mainland), from original source of depositor | Wang et al. (2016), direct submission |
| *Fejervarya kawamurai* (Clade II) | *Fejervarya multistriata* | FM_HN_08 | Northern Chinese Mainland |  |  | China | Verified | Precise locality unavailable, broadly assigned as Northern China (likely mainland), from original source of depositor | Wang et al. (2016), direct submission |
| *Fejervarya kawamurai* (Clade II) | *Fejervarya multistriata* | FM_HN_09 | Northern Chinese Mainland |  |  | China | Verified | Precise locality unavailable, broadly assigned as Northern China (likely mainland), from original source of depositor | Wang et al. (2016), direct submission |
| *Fejervarya kawamurai* (Clade II) | *Fejervarya multistriata* | FM_HN_10 | Northern Chinese Mainland |  |  | China | Verified | Precise locality unavailable, broadly assigned as Northern China (likely mainland), from original source of depositor | Wang et al. (2016), direct submission |
| *Fejervarya kawamurai* (Clade II) | *Fejervarya multistriata* | MZ44_MZ | Chongming, Shanghai |  |  | China | Verified | Voucher verified with locality name in GenBank metadata (no coordinates available) | Ding and Chen (2021), direct submission |
| *Fejervarya kawamurai* (Clade II) | *Fejervarya multistriata* | ZWc_1113_BZ | Chongming, Shanghai |  |  | China | Verified | Voucher verified with locality name in GenBank metadata (no coordinates available) | Ding and Chen (2021), direct submission |
| *Fejervarya kawamurai* (Clade II) | *Fejervarya multistriata* | ZWc_1121_BZ | Chongming, Shanghai |  |  | China | Verified | Voucher verified with locality name in GenBank metadata (no coordinates available) | Ding and Chen (2021), direct submission |
| *Fejervarya kawamurai* (Clade II) | *Fejervarya multistriata* | ZWc_13111_MZ | Chongming, Shanghai |  |  | China | Verified | Voucher verified with locality name in GenBank metadata (no coordinates available) | Ding and Chen (2021), direct submission |
| *Fejervarya kawamurai* (Clade II) | *Fejervarya multistriata* | ZWc_1317_MZ | Chongming, Shanghai |  |  | China | Verified | Voucher verified with locality name in GenBank metadata (no coordinates available) | Ding and Chen (2021), direct submission |
| *Fejervarya kawamurai* (Clade II) | *Fejervarya multistriata* | ZWc_1343_MZ | Chongming, Shanghai |  |  | China | Verified | Voucher verified with locality name in GenBank metadata (no coordinates available) | Ding and Chen (2021), direct submission |
| *Fejervarya kawamurai* (Clade II) | *Fejervarya multistriata* | ZWc_1345_MZ | Chongming, Shanghai |  |  | China | Verified | Voucher verified with locality name in GenBank metadata (no coordinates available) | Ding and Chen (2021), direct submission |
| *Fejervarya kawamurai* (Clade II) | *Fejervarya multistriata* | ZWc_1346_MZ | Chongming, Shanghai |  |  | China | Verified | Voucher verified with locality name in GenBank metadata (no coordinates available) | Ding and Chen (2021), direct submission |
| *Fejervarya kawamurai* (Clade II) | *Fejervarya multistriata* | ZWc_1516a_MT | Chongming, Shanghai |  |  | China | Verified | Voucher verified with locality name in GenBank metadata (no coordinates available) | Ding and Chen (2021), direct submission |
| *Fejervarya kawamurai* (Clade II) | *Fejervarya multistriata* | ZWc_1527_MZH | Chongming, Shanghai |  |  | China | Verified | Voucher verified with locality name in GenBank metadata (no coordinates available) | Ding and Chen (2021), direct submission |
| *Fejervarya kawamurai* (Clade II) | *Fejervarya multistriata* | ZWc_1611b_BBY | Chongming, Shanghai |  |  | China | Verified | Voucher verified with locality name in GenBank metadata (no coordinates available) | Ding and Chen (2021), direct submission |
| *Fejervarya kawamurai* (Clade II) | *Fejervarya multistriata* | ZWc_1621_BBY | Chongming, Shanghai |  |  | China | Verified | Voucher verified with locality name in GenBank metadata (no coordinates available) | Ding and Chen (2021), direct submission |
| *Fejervarya kawamurai* (Clade II) | *Fejervarya multistriata* | ZWc_1622b_BBY | Chongming, Shanghai |  |  | China | Verified | Voucher verified with locality name in GenBank metadata (no coordinates available) | Ding and Chen (2021), direct submission |
| *Fejervarya kawamurai* (Clade II) | *Fejervarya multistriata* | ZWc_1643b_BBY | Chongming, Shanghai |  |  | China | Verified | Voucher verified with locality name in GenBank metadata (no coordinates available) | Ding and Chen (2021), direct submission |
| *Fejervarya kawamurai* (Clade II) | *Fejervarya multistriata* | ZWc_1646b_BBY | Chongming, Shanghai |  |  | China | Verified | Voucher verified with locality name in GenBank metadata (no coordinates available) | Ding and Chen (2021), direct submission |
| *Fejervarya kawamurai* (Clade II) | *Fejervarya multistriata* | ZWc_1819b_DP | Chongming, Shanghai |  |  | China | Verified | Voucher verified with locality name in GenBank metadata (no coordinates available) | Ding and Chen (2021), direct submission |
| *Fejervarya kawamurai* (Clade II) | *Fejervarya multistriata* | ZWc_1826_DP | Chongming, Shanghai |  |  | China | Verified | Voucher verified with locality name in GenBank metadata (no coordinates available) | Ding and Chen (2021), direct submission |
| *Fejervarya kawamurai* (Clade II) | *Fejervarya multistriata* | ZWc_BBY29_BBY | Chongming, Shanghai |  |  | China | Verified | Voucher verified with locality name in GenBank metadata (no coordinates available) | Ding and Chen (2021), direct submission |
| *Fejervarya kawamurai* (Clade II) | *Fejervarya multistriata* | ZWc_BHQ07_DT | Chongming, Shanghai |  |  | China | Verified | Voucher verified with locality name in GenBank metadata (no coordinates available) | Ding and Chen (2021), direct submission |
| *Fejervarya kawamurai* (Clade II) | *Fejervarya multistriata* | ZWc_BHQ12_DT | Chongming, Shanghai |  |  | China | Verified | Voucher verified with locality name in GenBank metadata (no coordinates available) | Ding and Chen (2021), direct submission |
| *Fejervarya kawamurai* (Clade II) | *Fejervarya multistriata* | ZWc_BHQ14_DT | Chongming, Shanghai |  |  | China | Verified | Voucher verified with locality name in GenBank metadata (no coordinates available) | Ding and Chen (2021), direct submission |
| *Fejervarya kawamurai* (Clade II) | *Fejervarya multistriata* | ZWc_BZ09_BZ | Chongming, Shanghai |  |  | China | Verified | Voucher verified with locality name in GenBank metadata (no coordinates available) | Ding and Chen (2021), direct submission |
| *Fejervarya kawamurai* (Clade II) | *Fejervarya multistriata* | ZWc_BZ10_BZ | Chongming, Shanghai |  |  | China | Verified | Voucher verified with locality name in GenBank metadata (no coordinates available) | Ding and Chen (2021), direct submission |
| *Fejervarya kawamurai* (Clade II) | *Fejervarya multistriata* | ZWc_BZ13_BZ | Chongming, Shanghai |  |  | China | Verified | Voucher verified with locality name in GenBank metadata (no coordinates available) | Ding and Chen (2021), direct submission |
| *Fejervarya kawamurai* (Clade II) | *Fejervarya multistriata* | ZWc_BZ23_BZ | Chongming, Shanghai |  |  | China | Verified | Voucher verified with locality name in GenBank metadata (no coordinates available) | Ding and Chen (2021), direct submission |
| *Fejervarya kawamurai* (Clade II) | *Fejervarya multistriata* | ZWc_DL01_DL | Chongming, Shanghai |  |  | China | Verified | Voucher verified with locality name in GenBank metadata (no coordinates available) | Ding and Chen (2021), direct submission |
| *Fejervarya kawamurai* (Clade II) | *Fejervarya multistriata* | ZWc_DL02_DL | Chongming, Shanghai |  |  | China | Verified | Voucher verified with locality name in GenBank metadata (no coordinates available) | Ding and Chen (2021), direct submission |
| *Fejervarya kawamurai* (Clade II) | *Fejervarya multistriata* | ZWc_DL03_DL | Chongming, Shanghai |  |  | China | Verified | Voucher verified with locality name in GenBank metadata (no coordinates available) | Ding and Chen (2021), direct submission |
| *Fejervarya kawamurai* (Clade II) | *Fejervarya multistriata* | ZWc_DL11_DL | Chongming, Shanghai |  |  | China | Verified | Voucher verified with locality name in GenBank metadata (no coordinates available) | Ding and Chen (2021), direct submission |
| *Fejervarya kawamurai* (Clade II) | *Fejervarya multistriata* | ZWc_DL17_DL | Chongming, Shanghai |  |  | China | Verified | Voucher verified with locality name in GenBank metadata (no coordinates available) | Ding and Chen (2021), direct submission |
| *Fejervarya kawamurai* (Clade II) | *Fejervarya multistriata* | ZWc_DL18_DL | Chongming, Shanghai |  |  | China | Verified | Voucher verified with locality name in GenBank metadata (no coordinates available) | Ding and Chen (2021), direct submission |
| *Fejervarya kawamurai* (Clade II) | *Fejervarya multistriata* | ZWc_DT12_DT | Chongming, Shanghai |  |  | China | Verified | Voucher verified with locality name in GenBank metadata (no coordinates available) | Ding and Chen (2021), direct submission |
| *Fejervarya kawamurai* (Clade II) | *Fejervarya multistriata* | ZWc_DT2_DT | Chongming, Shanghai |  |  | China | Verified | Voucher verified with locality name in GenBank metadata (no coordinates available) | Ding and Chen (2021), direct submission |
| *Fejervarya kawamurai* (Clade II) | *Fejervarya multistriata* | ZWc_DT5_DT | Chongming, Shanghai |  |  | China | Verified | Voucher verified with locality name in GenBank metadata (no coordinates available) | Ding and Chen (2021), direct submission |
| *Fejervarya kawamurai* (Clade II) | *Fejervarya multistriata* | ZWc_DT8_DT | Chongming, Shanghai |  |  | China | Verified | Voucher verified with locality name in GenBank metadata (no coordinates available) | Ding and Chen (2021), direct submission |
| *Fejervarya kawamurai* (Clade II) | *Fejervarya multistriata* | ZWc_HS14_HS | Chongming, Shanghai |  |  | China | Verified | Voucher verified with locality name in GenBank metadata (no coordinates available) | Ding and Chen (2021), direct submission |
| *Fejervarya kawamurai* (Clade II) | *Fejervarya multistriata* | ZWc_HS16_HS | Chongming, Shanghai |  |  | China | Verified | Voucher verified with locality name in GenBank metadata (no coordinates available) | Ding and Chen (2021), direct submission |
| *Fejervarya kawamurai* (Clade II) | *Fejervarya multistriata* | ZWc_HS17_HS | Chongming, Shanghai |  |  | China | Verified | Voucher verified with locality name in GenBank metadata (no coordinates available) | Ding and Chen (2021), direct submission |
| *Fejervarya kawamurai* (Clade II) | *Fejervarya multistriata* | ZWc_MT02_MT | Chongming, Shanghai |  |  | China | Verified | Voucher verified with locality name in GenBank metadata (no coordinates available) | Ding and Chen (2021), direct submission |
| *Fejervarya kawamurai* (Clade II) | *Fejervarya multistriata* | ZWc_MT09_MT | Chongming, Shanghai |  |  | China | Verified | Voucher verified with locality name in GenBank metadata (no coordinates available) | Ding and Chen (2021), direct submission |
| *Fejervarya kawamurai* (Clade II) | *Fejervarya multistriata* | ZWc_MT10_MT | Chongming, Shanghai |  |  | China | Verified | Voucher verified with locality name in GenBank metadata (no coordinates available) | Ding and Chen (2021), direct submission |
| *Fejervarya kawamurai* (Clade II) | *Fejervarya multistriata* | ZWc_MT13_MT | Chongming, Shanghai |  |  | China | Verified | Voucher verified with locality name in GenBank metadata (no coordinates available) | Ding and Chen (2021), direct submission |
| *Fejervarya kawamurai* (Clade II) | *Fejervarya multistriata* | ZWc_MT16_MT | Chongming, Shanghai |  |  | China | Verified | Voucher verified with locality name in GenBank metadata (no coordinates available) | Ding and Chen (2021), direct submission |
| *Fejervarya kawamurai* (Clade II) | *Fejervarya multistriata* | ZWc_MT18_MT | Chongming, Shanghai |  |  | China | Verified | Voucher verified with locality name in GenBank metadata (no coordinates available) | Ding and Chen (2021), direct submission |
| *Fejervarya kawamurai* (Clade II) | *Fejervarya multistriata* | ZWc_MZ01_MZ | Chongming, Shanghai |  |  | China | Verified | Voucher verified with locality name in GenBank metadata (no coordinates available) | Ding and Chen (2021), direct submission |
| *Fejervarya kawamurai* (Clade II) | *Fejervarya multistriata* | ZWc_MZ04_MZ | Chongming, Shanghai |  |  | China | Verified | Voucher verified with locality name in GenBank metadata (no coordinates available) | Ding and Chen (2021), direct submission |
| *Fejervarya kawamurai* (Clade II) | *Fejervarya multistriata* | ZWc_MZ06_MZ | Chongming, Shanghai |  |  | China | Verified | Voucher verified with locality name in GenBank metadata (no coordinates available) | Ding and Chen (2021), direct submission |
| *Fejervarya kawamurai* (Clade II) | *Fejervarya multistriata* | ZWc_MZ09_MZ | Chongming, Shanghai |  |  | China | Verified | Voucher verified with locality name in GenBank metadata (no coordinates available) | Ding and Chen (2021), direct submission |
| *Fejervarya kawamurai* (Clade II) | *Fejervarya multistriata* | ZWc_MZ10_MZ | Chongming, Shanghai |  |  | China | Verified | Voucher verified with locality name in GenBank metadata (no coordinates available) | Ding and Chen (2021), direct submission |
| *Fejervarya kawamurai* (Clade II) | *Fejervarya multistriata* | _ZWc_MZ11_MZ | Chongming, Shanghai |  |  | China | Verified | Voucher verified with locality name in GenBank metadata (no coordinates available) | Ding and Chen (2021), direct submission |
| *Fejervarya kawamurai* (Clade II) | *Fejervarya multistriata* | ZWc_MZ13_MZ | Chongming, Shanghai |  |  | China | Verified | Voucher verified with locality name in GenBank metadata (no coordinates available) | Ding and Chen (2021), direct submission |
| *Fejervarya kawamurai* (Clade II) | *Fejervarya multistriata* | ZWc_MZ14_MZ | Chongming, Shanghai |  |  | China | Verified | Voucher verified with locality name in GenBank metadata (no coordinates available) | Ding and Chen (2021), direct submission |
| *Fejervarya kawamurai* (Clade II) | *Fejervarya multistriata* | ZWc_MZ15_MZ | Chongming, Shanghai |  |  | China | Verified | Voucher verified with locality name in GenBank metadata (no coordinates available) | Ding and Chen (2021), direct submission |
| *Fejervarya kawamurai* (Clade II) | *Fejervarya multistriata* | ZWc_MZ16_MZ | Chongming, Shanghai |  |  | China | Verified | Voucher verified with locality name in GenBank metadata (no coordinates available) | Ding and Chen (2021), direct submission |
| *Fejervarya kawamurai* (Clade II) | *Fejervarya multistriata* | ZWc_MZ21_MZ | Chongming, Shanghai |  |  | China | Verified | Voucher verified with locality name in GenBank metadata (no coordinates available) | Ding and Chen (2021), direct submission |
| *Fejervarya kawamurai* (Clade II) | *Fejervarya multistriata* | ZWc_MZ22_MZ | Chongming, Shanghai |  |  | China | Verified | Voucher verified with locality name in GenBank metadata (no coordinates available) | Ding and Chen (2021), direct submission |
| *Fejervarya kawamurai* (Clade II) | *Fejervarya multistriata* | ZWc_MZ23_MZ | Chongming, Shanghai |  |  | China | Verified | Voucher verified with locality name in GenBank metadata (no coordinates available) | Ding and Chen (2021), direct submission |
| *Fejervarya kawamurai* (Clade II) | *Fejervarya multistriata* | ZWc_MZ26_MZ | Chongming, Shanghai |  |  | China | Verified | Voucher verified with locality name in GenBank metadata (no coordinates available) | Ding and Chen (2021), direct submission |
| *Fejervarya kawamurai* (Clade II) | *Fejervarya multistriata* | ZWc_MZ30_MZ | Chongming, Shanghai |  |  | China | Verified | Voucher verified with locality name in GenBank metadata (no coordinates available) | Ding and Chen (2021), direct submission |
| *Fejervarya kawamurai* (Clade II) | *Fejervarya multistriata* | ZWc_MZH15_MZH | Chongming, Shanghai |  |  | China | Verified | Voucher verified with locality name in GenBank metadata (no coordinates available) | Ding and Chen (2021), direct submission |
| *Fejervarya kawamurai* (Clade II) | *Fejervarya multistriata* | ZWc_QJX26_QXJ | Chongming, Shanghai |  |  | China | Verified | Voucher verified with locality name in GenBank metadata (no coordinates available) | Ding and Chen (2021), direct submission |
| *Fejervarya kawamurai* (Clade II) | *Fejervarya multistriata* | ZWc_QJX31_QXJ | Chongming, Shanghai |  |  | China | Verified | Voucher verified with locality name in GenBank metadata (no coordinates available) | Ding and Chen (2021), direct submission |
| *Fejervarya kawamurai* (Clade II) | *Fejervarya multistriata* | ZWc_QXJ37_QXJ | Chongming, Shanghai |  |  | China | Verified | Voucher verified with locality name in GenBank metadata (no coordinates available) | Ding and Chen (2021), direct submission |
| *Fejervarya kawamurai* (Clade II) | *Fejervarya multistriata* | ZWc_QXJ46_QXJ | Chongming, Shanghai |  |  | China | Verified | Voucher verified with locality name in GenBank metadata (no coordinates available) | Ding and Chen (2021), direct submission |
| *Fejervarya kawamurai* (Clade II) | *Fejervarya multistriata* | ZWd1102_BZ | Chongming, Shanghai |  |  | China | Verified | Voucher verified with locality name in GenBank metadata (no coordinates available) | Ding and Chen (2021), direct submission |
| *Fejervarya kawamurai* (Clade II) | *Fejervarya multistriata* | ZWd1103_BZ | Chongming, Shanghai |  |  | China | Verified | Voucher verified with locality name in GenBank metadata (no coordinates available) | Ding and Chen (2021), direct submission |
| *Fejervarya kawamurai* (Clade II) | *Fejervarya multistriata* | ZWd1117_BZ | Chongming, Shanghai |  |  | China | Verified | Voucher verified with locality name in GenBank metadata (no coordinates available) | Ding and Chen (2021), direct submission |
| *Fejervarya kawamurai* (Clade II) | *Fejervarya multistriata* | ZWd1135_BZ | Chongming, Shanghai |  |  | China | Verified | Voucher verified with locality name in GenBank metadata (no coordinates available) | Ding and Chen (2021), direct submission |
| *Fejervarya kawamurai* (Clade II) | *Fejervarya multistriata* | ZWd1211_BZ | Chongming, Shanghai |  |  | China | Verified | Voucher verified with locality name in GenBank metadata (no coordinates available) | Ding and Chen (2021), direct submission |
| *Fejervarya kawamurai* (Clade II) | *Fejervarya multistriata* | ZWd1215_DP | Chongming, Shanghai |  |  | China | Verified | Voucher verified with locality name in GenBank metadata (no coordinates available) | Ding and Chen (2021), direct submission |
| *Fejervarya kawamurai* (Clade II) | *Fejervarya multistriata* | ZWd1231_BZ | Chongming, Shanghai |  |  | China | Verified | Voucher verified with locality name in GenBank metadata (no coordinates available) | Ding and Chen (2021), direct submission |
| *Fejervarya kawamurai* (Clade II) | *Fejervarya multistriata* | ZWd1244_BZ | Chongming, Shanghai |  |  | China | Verified | Voucher verified with locality name in GenBank metadata (no coordinates available) | Ding and Chen (2021), direct submission |
| *Fejervarya kawamurai* (Clade II) | *Fejervarya multistriata* | ZWd1339_MZ | Chongming, Shanghai |  |  | China | Verified | Voucher verified with locality name in GenBank metadata (no coordinates available) | Ding and Chen (2021), direct submission |
| *Fejervarya kawamurai* (Clade II) | *Fejervarya multistriata* | ZWd1343_MZ | Chongming, Shanghai |  |  | China | Verified | Voucher verified with locality name in GenBank metadata (no coordinates available) | Ding and Chen (2021), direct submission |
| *Fejervarya kawamurai* (Clade II) | *Fejervarya multistriata* | ZWd1515_MT | Chongming, Shanghai |  |  | China | Verified | Voucher verified with locality name in GenBank metadata (no coordinates available) | Ding and Chen (2021), direct submission |
| *Fejervarya kawamurai* (Clade II) | *Fejervarya multistriata* | ZWd1516_MT | Chongming, Shanghai |  |  | China | Verified | Voucher verified with locality name in GenBank metadata (no coordinates available) | Ding and Chen (2021), direct submission |
| *Fejervarya kawamurai* (Clade II) | *Fejervarya multistriata* | ZWd1525_MT | Chongming, Shanghai |  |  | China | Verified | Voucher verified with locality name in GenBank metadata (no coordinates available) | Ding and Chen (2021), direct submission |
| *Fejervarya kawamurai* (Clade II) | *Fejervarya multistriata* | ZWd1548a_MZH | Chongming, Shanghai |  |  | China | Verified | Voucher verified with locality name in GenBank metadata (no coordinates available) | Ding and Chen (2021), direct submission |
| *Fejervarya kawamurai* (Clade II) | *Fejervarya multistriata* | ZWd1613_BBY | Chongming, Shanghai |  |  | China | Verified | Voucher verified with locality name in GenBank metadata (no coordinates available) | Ding and Chen (2021), direct submission |
| *Fejervarya kawamurai* (Clade II) | *Fejervarya multistriata* | ZWd1617b_BBY | Chongming, Shanghai |  |  | China | Verified | Voucher verified with locality name in GenBank metadata (no coordinates available) | Ding and Chen (2021), direct submission |
| *Fejervarya kawamurai* (Clade II) | *Fejervarya multistriata* | ZWd1619b_BBY | Chongming, Shanghai |  |  | China | Verified | Voucher verified with locality name in GenBank metadata (no coordinates available) | Ding and Chen (2021), direct submission |
| *Fejervarya kawamurai* (Clade II) | *Fejervarya multistriata* | ZWd1621b_BBY | Chongming, Shanghai |  |  | China | Verified | Voucher verified with locality name in GenBank metadata (no coordinates available) | Ding and Chen (2021), direct submission |
| *Fejervarya kawamurai* (Clade II) | *Fejervarya multistriata* | ZWd1634_BBY | Chongming, Shanghai |  |  | China | Verified | Voucher verified with locality name in GenBank metadata (no coordinates available) | Ding and Chen (2021), direct submission |
| *Fejervarya kawamurai* (Clade II) | *Fejervarya multistriata* | ZWd1641b_BBY | Chongming, Shanghai |  |  | China | Verified | Voucher verified with locality name in GenBank metadata (no coordinates available) | Ding and Chen (2021), direct submission |
| *Fejervarya kawamurai* (Clade II) | *Fejervarya multistriata* | ZWd1642b_BBY | Chongming, Shanghai |  |  | China | Verified | Voucher verified with locality name in GenBank metadata (no coordinates available) | Ding and Chen (2021), direct submission |
| *Fejervarya kawamurai* (Clade II) | *Fejervarya multistriata* | ZWd1647b_BBY | Chongming, Shanghai |  |  | China | Verified | Voucher verified with locality name in GenBank metadata (no coordinates available) | Ding and Chen (2021), direct submission |
| *Fejervarya kawamurai* (Clade II) | *Fejervarya multistriata* | ZWd1813b_DP | Chongming, Shanghai |  |  | China | Verified | Voucher verified with locality name in GenBank metadata (no coordinates available) | Ding and Chen (2021), direct submission |
| *Fejervarya kawamurai* (Clade II) | *Fejervarya multistriata* | ZWd1814_DP | Chongming, Shanghai |  |  | China | Verified | Voucher verified with locality name in GenBank metadata (no coordinates available) | Ding and Chen (2021), direct submission |
| *Fejervarya kawamurai* (Clade II) | *Fejervarya multistriata* | ZWd1814b_DP | Chongming, Shanghai |  |  | China | Verified | Voucher verified with locality name in GenBank metadata (no coordinates available) | Ding and Chen (2021), direct submission |
| *Fejervarya kawamurai* (Clade II) | *Fejervarya multistriata* | ZWd1818_DP | Chongming, Shanghai |  |  | China | Verified | Voucher verified with locality name in GenBank metadata (no coordinates available) | Ding and Chen (2021), direct submission |
| *Fejervarya kawamurai* (Clade II) | *Fejervarya multistriata* | ZWd1818b_DP | Chongming, Shanghai |  |  | China | Verified | Voucher verified with locality name in GenBank metadata (no coordinates available) | Ding and Chen (2021), direct submission |
| *Fejervarya kawamurai* (Clade II) | *Fejervarya multistriata* | ZWd1819sb_DP | Chongming, Shanghai |  |  | China | Verified | Voucher verified with locality name in GenBank metadata (no coordinates available) | Ding and Chen (2021), direct submission |
| *Fejervarya kawamurai* (Clade II) | *Fejervarya multistriata* | ZWd1842_DP | Chongming, Shanghai |  |  | China | Verified | Voucher verified with locality name in GenBank metadata (no coordinates available) | Ding and Chen (2021), direct submission |
| *Fejervarya kawamurai* (Clade II) | *Fejervarya multistriata* | ZWdBBY09_BBY | Chongming, Shanghai |  |  | China | Verified | Voucher verified with locality name in GenBank metadata (no coordinates available) | Ding and Chen (2021), direct submission |
| *Fejervarya kawamurai* (Clade II) | *Fejervarya multistriata* | ZWdBBY29_BBY | Chongming, Shanghai |  |  | China | Verified | Voucher verified with locality name in GenBank metadata (no coordinates available) | Ding and Chen (2021), direct submission |
| *Fejervarya kawamurai* (Clade II) | *Fejervarya multistriata* | ZWdBZ03_BZ | Chongming, Shanghai |  |  | China | Verified | Voucher verified with locality name in GenBank metadata (no coordinates available) | Ding and Chen (2021), direct submission |
| *Fejervarya kawamurai* (Clade II) | *Fejervarya multistriata* | ZWdBZ18_BZ | Chongming, Shanghai |  |  | China | Verified | Voucher verified with locality name in GenBank metadata (no coordinates available) | Ding and Chen (2021), direct submission |
| *Fejervarya kawamurai* (Clade II) | *Fejervarya multistriata* | ZWdBZ22_BZ | Chongming, Shanghai |  |  | China | Verified | Voucher verified with locality name in GenBank metadata (no coordinates available) | Ding and Chen (2021), direct submission |
| *Fejervarya kawamurai* (Clade II) | *Fejervarya multistriata* | ZWdCX03_CX | Chongming, Shanghai |  |  | China | Verified | Voucher verified with locality name in GenBank metadata (no coordinates available) | Ding and Chen (2021), direct submission |
| *Fejervarya kawamurai* (Clade II) | *Fejervarya multistriata* | ZWdCX16_CX | Chongming, Shanghai |  |  | China | Verified | Voucher verified with locality name in GenBank metadata (no coordinates available) | Ding and Chen (2021), direct submission |
| *Fejervarya kawamurai* (Clade II) | *Fejervarya multistriata* | ZWdCXD1033_CX | Chongming, Shanghai |  |  | China | Verified | Voucher verified with locality name in GenBank metadata (no coordinates available) | Ding and Chen (2021), direct submission |
| *Fejervarya kawamurai* (Clade II) | *Fejervarya multistriata* | ZWdDL01_DL | Chongming, Shanghai |  |  | China | Verified | Voucher verified with locality name in GenBank metadata (no coordinates available) | Ding and Chen (2021), direct submission |
| *Fejervarya kawamurai* (Clade II) | *Fejervarya multistriata* | ZWdDL04_DL | Chongming, Shanghai |  |  | China | Verified | Voucher verified with locality name in GenBank metadata (no coordinates available) | Ding and Chen (2021), direct submission |
| *Fejervarya kawamurai* (Clade II) | *Fejervarya multistriata* | ZWdDL06_DL | Chongming, Shanghai |  |  | China | Verified | Voucher verified with locality name in GenBank metadata (no coordinates available) | Ding and Chen (2021), direct submission |
| *Fejervarya kawamurai* (Clade II) | *Fejervarya multistriata* | ZWdDL07_DL | Chongming, Shanghai |  |  | China | Verified | Voucher verified with locality name in GenBank metadata (no coordinates available) | Ding and Chen (2021), direct submission |
| *Fejervarya kawamurai* (Clade II) | *Fejervarya multistriata* | ZWdDL09_DL | Chongming, Shanghai |  |  | China | Verified | Voucher verified with locality name in GenBank metadata (no coordinates available) | Ding and Chen (2021), direct submission |
| *Fejervarya kawamurai* (Clade II) | *Fejervarya multistriata* | ZWdDL14_DL | Chongming, Shanghai |  |  | China | Verified | Voucher verified with locality name in GenBank metadata (no coordinates available) | Ding and Chen (2021), direct submission |
| *Fejervarya kawamurai* (Clade II) | *Fejervarya multistriata* | ZWdDL15_DL | Chongming, Shanghai |  |  | China | Verified | Voucher verified with locality name in GenBank metadata (no coordinates available) | Ding and Chen (2021), direct submission |
| *Fejervarya kawamurai* (Clade II) | *Fejervarya multistriata* | ZWdDL17_DL | Chongming, Shanghai |  |  | China | Verified | Voucher verified with locality name in GenBank metadata (no coordinates available) | Ding and Chen (2021), direct submission |
| *Fejervarya kawamurai* (Clade II) | *Fejervarya multistriata* | ZWdDL18_DL | Chongming, Shanghai |  |  | China | Verified | Voucher verified with locality name in GenBank metadata (no coordinates available) | Ding and Chen (2021), direct submission |
| *Fejervarya kawamurai* (Clade II) | *Fejervarya multistriata* | ZWdDP12_DP | Chongming, Shanghai |  |  | China | Verified | Voucher verified with locality name in GenBank metadata (no coordinates available) | Ding and Chen (2021), direct submission |
| *Fejervarya kawamurai* (Clade II) | *Fejervarya multistriata* | ZWdDP18_DP | Chongming, Shanghai |  |  | China | Verified | Voucher verified with locality name in GenBank metadata (no coordinates available) | Ding and Chen (2021), direct submission |
| *Fejervarya kawamurai* (Clade II) | *Fejervarya multistriata* | ZWdDT2_DT | Chongming, Shanghai |  |  | China | Verified | Voucher verified with locality name in GenBank metadata (no coordinates available) | Ding and Chen (2021), direct submission |
| *Fejervarya kawamurai* (Clade II) | *Fejervarya multistriata* | ZWdHS001_HS | Chongming, Shanghai |  |  | China | Verified | Voucher verified with locality name in GenBank metadata (no coordinates available) | Ding and Chen (2021), direct submission |
| *Fejervarya kawamurai* (Clade II) | *Fejervarya multistriata* | ZWdHS037_HS | Chongming, Shanghai |  |  | China | Verified | Voucher verified with locality name in GenBank metadata (no coordinates available) | Ding and Chen (2021), direct submission |
| *Fejervarya kawamurai* (Clade II) | *Fejervarya multistriata* | ZWdHS12_HS | Chongming, Shanghai |  |  | China | Verified | Voucher verified with locality name in GenBank metadata (no coordinates available) | Ding and Chen (2021), direct submission |
| *Fejervarya kawamurai* (Clade II) | *Fejervarya multistriata* | ZWdHS17_HS | Chongming, Shanghai |  |  | China | Verified | Voucher verified with locality name in GenBank metadata (no coordinates available) | Ding and Chen (2021), direct submission |
| *Fejervarya kawamurai* (Clade II) | *Fejervarya multistriata* | ZWdMT02_MT | Chongming, Shanghai |  |  | China | Verified | Voucher verified with locality name in GenBank metadata (no coordinates available) | Ding and Chen (2021), direct submission |
| *Fejervarya kawamurai* (Clade II) | *Fejervarya multistriata* | ZWdMT03_MT | Chongming, Shanghai |  |  | China | Verified | Voucher verified with locality name in GenBank metadata (no coordinates available) | Ding and Chen (2021), direct submission |
| *Fejervarya kawamurai* (Clade II) | *Fejervarya multistriata* | ZWdMT08_MT | Chongming, Shanghai |  |  | China | Verified | Voucher verified with locality name in GenBank metadata (no coordinates available) | Ding and Chen (2021), direct submission |
| *Fejervarya kawamurai* (Clade II) | *Fejervarya multistriata* | ZWdMT09_MT | Chongming, Shanghai |  |  | China | Verified | Voucher verified with locality name in GenBank metadata (no coordinates available) | Ding and Chen (2021), direct submission |
| *Fejervarya kawamurai* (Clade II) | *Fejervarya multistriata* | ZWdMT13_MT | Chongming, Shanghai |  |  | China | Verified | Voucher verified with locality name in GenBank metadata (no coordinates available) | Ding and Chen (2021), direct submission |
| *Fejervarya kawamurai* (Clade II) | *Fejervarya multistriata* | ZWdMT18_MT | Chongming, Shanghai |  |  | China | Verified | Voucher verified with locality name in GenBank metadata (no coordinates available) | Ding and Chen (2021), direct submission |
| *Fejervarya kawamurai* (Clade II) | *Fejervarya multistriata* | ZWdMZ06_MZ | Chongming, Shanghai |  |  | China | Verified | Voucher verified with locality name in GenBank metadata (no coordinates available) | Ding and Chen (2021), direct submission |
| *Fejervarya kawamurai* (Clade II) | *Fejervarya multistriata* | ZWdMZ11_MZ | Chongming, Shanghai |  |  | China | Verified | Voucher verified with locality name in GenBank metadata (no coordinates available) | Ding and Chen (2021), direct submission |
| *Fejervarya kawamurai* (Clade II) | *Fejervarya multistriata* | ZWdMZ13_MZ | Chongming, Shanghai |  |  | China | Verified | Voucher verified with locality name in GenBank metadata (no coordinates available) | Ding and Chen (2021), direct submission |
| *Fejervarya kawamurai* (Clade II) | *Fejervarya multistriata* | ZWdMZ14_MZ | Chongming, Shanghai |  |  | China | Verified | Voucher verified with locality name in GenBank metadata (no coordinates available) | Ding and Chen (2021), direct submission |
| *Fejervarya kawamurai* (Clade II) | *Fejervarya multistriata* | ZWdMZ26_MZ | Chongming, Shanghai |  |  | China | Verified | Voucher verified with locality name in GenBank metadata (no coordinates available) | Ding and Chen (2021), direct submission |
| *Fejervarya kawamurai* (Clade II) | *Fejervarya multistriata* | ZWdMZ30_MZ | Chongming, Shanghai |  |  | China | Verified | Voucher verified with locality name in GenBank metadata (no coordinates available) | Ding and Chen (2021), direct submission |
| *Fejervarya kawamurai* (Clade II) | *Fejervarya multistriata* | ZWdMZ32_MZ | Chongming, Shanghai |  |  | China | Verified | Voucher verified with locality name in GenBank metadata (no coordinates available) | Ding and Chen (2021), direct submission |
| *Fejervarya kawamurai* (Clade II) | *Fejervarya multistriata* | ZWdMZ35_MZ | Chongming, Shanghai |  |  | China | Verified | Voucher verified with locality name in GenBank metadata (no coordinates available) | Ding and Chen (2021), direct submission |
| *Fejervarya kawamurai* (Clade II) | *Fejervarya multistriata* | ZWdMZ46_MZ | Chongming, Shanghai |  |  | China | Verified | Voucher verified with locality name in GenBank metadata (no coordinates available) | Ding and Chen (2021), direct submission |
| *Fejervarya kawamurai* (Clade II) | *Fejervarya multistriata* | ZWdMZ47_MZ | Chongming, Shanghai |  |  | China | Verified | Voucher verified with locality name in GenBank metadata (no coordinates available) | Ding and Chen (2021), direct submission |
| *Fejervarya kawamurai* (Clade II) | *Fejervarya multistriata* | ZWdMZH04_MZH | Chongming, Shanghai |  |  | China | Verified | Voucher verified with locality name in GenBank metadata (no coordinates available) | Ding and Chen (2021), direct submission |
| *Fejervarya kawamurai* (Clade II) | *Fejervarya multistriata* | ZWdMZH09_MZH | Chongming, Shanghai |  |  | China | Verified | Voucher verified with locality name in GenBank metadata (no coordinates available) | Ding and Chen (2021), direct submission |
| *Fejervarya kawamurai* (Clade II) | *Fejervarya multistriata* | ZWdMZH16_MZH | Chongming, Shanghai |  |  | China | Verified | Voucher verified with locality name in GenBank metadata (no coordinates available) | Ding and Chen (2021), direct submission |
| *Fejervarya kawamurai* (Clade II) | *Fejervarya multistriata* | ZWdQXJ43_QXJ | Chongming, Shanghai |  |  | China | Verified | Voucher verified with locality name in GenBank metadata (no coordinates available) | Ding and Chen (2021), direct submission |
| *Fejervarya kawamurai* (Clade II) | *Fejervarya multistriata* | ZWd1819_DP | Chongming, Shanghai |  |  | China | Verified | Voucher verified with locality name in GenBank metadata (no coordinates available) | Ding and Chen (2021), direct submission |
| *Fejervarya kawamurai* (Clade II) | *Fejervarya multistriata* | ZWdBHQ06_DT | Chongming, Shanghai |  |  | China | Verified | Voucher verified with locality name in GenBank metadata (no coordinates available) | Ding and Chen (2021), direct submission |
| *Fejervarya kawamurai* (Clade II) | *Fejervarya multistriata* | ZWdMZ36_MZ | Chongming, Shanghai |  |  | China | Verified | Voucher verified with locality name in GenBank metadata (no coordinates available) | Ding and Chen (2021), direct submission |
| *Fejervarya multistriata* (Clade II) | *Fejervarya limnocharis* | 15083 | Taiwan Province, Taichung City, Taichung |  |  | Taiwan | Verified | Labelled as *F. limnocharis* in GenBank, but phylogenetically clusters within *F. multistriata* [Clade II] | Jang-Liaw and Chao (2008) |
| *Fejervarya multistriata* (Clade II) | *Fejervarya limnocharis* | 17691 | Lienchiang County, Nangan Township, Fuwoling, Taiwan | 26.156 | 119.947 | Taiwan | Verified | Labelled as *F. limnocharis* in GenBank, but phylogenetically clusters within *F. multistriata* [Clade II] | Jang-Liaw and Chao (2008) |
| *Fejervarya multistriata* (Clade II) | *Fejervarya limnocharis* | 17744 | New Taipei City, Shimen District, Jutsao | 25.268 | 121.551 | Taiwan | Verified | Labelled as *F. limnocharis* in GenBank, but phylogenetically clusters within *F. multistriata* [Clade II] | Jang-Liaw and Chao (2008) |
| *Fejervarya multistriata* (Clade I) | *Fejervarya limnocharis* | 17620 | Guangdong, Shaoguan City, Juyuan County, Tianjingshan Forestry Station | 24.698 | 112.988 | China | Verified | Labelled as *F. limnocharis* in GenBank, but phylogenetically clusters within *F. multistriata* [Clade II] | Jang-Liaw and Chao (2008) |
| *Fejervarya multistriata (Clade I)* | *Fejervarya limnocharis* | 17674 | Fujian, Xiamen City, Xiamen University | 24.442 | 118.099 | China | Verified | Labelled as *F. limnocharis* in GenBank, but phylogenetically clusters within *F. multistriata* [Clade II] | Jang-Liaw and Chao (2008) |
| *Fejervarya multistriata* (Clade II) | *Fejervarya limnocharis* | 18178 | Pintung County, Jiadung Township, Hsiaputou | 22.4092 | 120.561 | Taiwan | Verified | Labelled as *F. limnocharis* in GenBank, but phylogenetically clusters within *F. multistriata* [Clade II] | Jang-Liaw and Chao (2008) |
| *Fejervarya multistriata* (Clade II) | *Fejervarya limnocharis* | 18238 | Yilan County, Toucheng Township, Toucheng | 24.893 | 121.856 | Taiwan | Verified | Labelled as *F. limnocharis* in GenBank, but phylogenetically clusters within *F. multistriata* [Clade II] | Jang-Liaw and Chao (2008) |
| *Fejervarya multistriata* (Clade II) | *Fejervarya limnocharis* | JL73-2-1 | Yilan County, Nan-ao | 24.4388 | 121.764 | Taiwan | Verified | Labelled as *F. limnocharis* in GenBank, but phylogenetically clusters within *F. multistriata* [Clade II] | Jang-Liaw and Chao (2008) |
| *Fejervarya multistriata* (Clade II) | *Fejervarya limnocharis* | JL73-2-2 | Yilan County, Nan-ao | 24.4388 | 121.764 | Taiwan | Verified | Labelled as *F. limnocharis* in GenBank, but phylogenetically clusters within *F. multistriata* [Clade II] | Jang-Liaw and Chao (2008) |
| *Fejervarya multistriata* (Clade II) | *Fejervarya limnocharis* | JL106 | Tainan City, Liouying | 23.261 | 120.378 | Taiwan | Verified | Labelled as *F. limnocharis* in GenBank, but phylogenetically clusters within *F. multistriata* [Clade II] | Jang-Liaw and Chao (2008) |
| *Fejervarya multistriata* (Clade II) | *Fejervarya limnocharis* | JL66-2 | Taiwan Province, Pingtung County, Sandimen | 22.7519 | 120.636 | Taiwan | Verified | Labelled as *F. limnocharis* in GenBank, but phylogenetically clusters within *F. multistriata* [Clade II] | Grosjean et al. (2015) |
| *Fejervarya multistriata* (Clade II) | *Fejervarya limnocharis* | JL-19-2 | Hualien County, Shoufeng Township | 23.8823 | 121.568 | Taiwan | Verified | Labelled as *F. limnocharis* in GenBank, but phylogenetically clusters within *F. multistriata* [Clade II] | Jang-Liaw and Chao (2008) |
| *Fejervarya limnocharis* (Clade II) | *Fejervarya limnocharis* | G024 | Hainan Island, China |  |  | China | Verified | Labelled as *F. limnocharis* in GenBank, but phylogenetically clusters within *F. multistriata* [Clade II] | Jang-Liaw and Chao (2008) |
| *Fejervarya limnocharis* (Clade II) | *Fejervarya limnocharis* | JL02-1 | Chubu, Shizuoka Prefecture, Central | 34.988 | 138.299 | Japan | Verified | Labelled as *F. limnocharis* in GenBank, but phylogenetically clusters within *F. multistriata* [Clade II] | Grosjean et al. (2015) |
| *Fejervarya limnocharis* (Clade II) | *Fejervarya multistriata* | NMNSF15460 | Pingtung County, Manjhou Township, Chiupeng | 22.1033 | 120.847 | Taiwan | Verified | Labelled as *F. limnocharis* in GenBank, but phylogenetically clusters within *F. multistriata* [Clade II] | Djong et al. (2007) |
| *Fejervarya limnocharis* (Clade II) | *Fejervarya multistriata* | fm1 | N/a, but original depositor is from Xian |  |  | China | Verified | Geographic origin is unknown, however original source of depoistor in Xian, China, and the sequence clustered to *F.limnocharis* | Wang et al. (2021), direct submission |
| *Fejervarya limnocharis* (Clade II) | *Fejervarya limnocharis* | TAD-P992 | Phangnga, Namtok Raman Forest Park |  |  | Thailand | Verified | Geographic origin is verified from GenBank metadata | Chang and Liu (2010), direct submission |
| *Fejervarya limnocharis* (Clade II) | *Fejervarya limnocharis* | USNM:Herp:595381 | south central extent of Trang An Landscape Complex |  |  | Vietnam | Verified | Geographic origin is verified from GenBank metadata | Miller et al. (2022), direct submission |
| *Fejervarya limnocharis* (Clade II) | *Fejervarya limnocharis* | USNM:Herp:595383 | Du Gia Forest, Bac Me, 3.2 km of Khau Ca Reserve (straight-line distance) |  |  | Vietnam | Verified | Geographic origin is verified from GenBank metadata | Miller et al. (2022), direct submission |
| *Fejervarya multistriata* (Clade II) | *Fejervarya limnocharis* | H001 | Taiwan Island |  |  | Taiwan | Verified | Labelled as *F. limnocharis* in GenBank, but phylogenetically clusters within *F. multistriata* [Clade II] | Yang et al. (2023) |
| *Fejervarya multistriata* (Clade II) | *Fejervarya limnocharis* | H002 | Taiwan Island |  |  | Taiwan | Verified | Labelled as *F. limnocharis* in GenBank, but phylogenetically clusters within *F. multistriata* [Clade II] | Yang et al. (2023) |
| *Fejervarya multistriata* (Clade II) | *Fejervarya limnocharis* | H005 | Taiwan Island |  |  | Taiwan | Verified | Labelled as *F. limnocharis* in GenBank, but phylogenetically clusters within *F. multistriata* [Clade II] | Yang et al. (2023) |
| *Fejervarya multistriata* (Clade II) | *Fejervarya limnocharis* | H011 | Taiwan Island |  |  | Taiwan | Verified | Labelled as *F. limnocharis* in GenBank, but phylogenetically clusters within *F. multistriata* [Clade II] | Yang et al. (2023) |
| *Fejervarya multistriata* (Clade II) | *Fejervarya limnocharis* | H012 | Taiwan Island |  |  | Taiwan | Verified | Labelled as *F. limnocharis* in GenBank, but phylogenetically clusters within *F. multistriata* [Clade II] | Jiang et al. (2020) |
| *Fejervarya multistriata* (Clade II) | *Fejervarya limnocharis* | H013 | Taiwan Island |  |  | Taiwan | Verified | Labelled as *F. limnocharis* in GenBank, but phylogenetically clusters within *F. multistriata* [Clade II] | Jiang et al. (2020) |
| *Fejervarya kawamurai* (Clade II) | *Fejervarya multistrata* | GZBY01 | Guangzhou |  |  | China | Verified | consistent assignment between GenBank annotation and species delimitation | Ding and Chen (2021) |
| *Fejervarya kawamurai* (Clade II) | *Fejervarya multistrata* | GZBY02 | Guangzhou |  |  | China | Verified | consistent assignment between GenBank annotation and species delimitation | Ding and Chen (2021) |
| *Fejervarya kawamurai* (Clade II) | *Fejervarya multistrata* | GZBY04 | Guangzhou |  |  | China | Verified | consistent assignment between GenBank annotation and species delimitation | ren et al. (2009) |
| *Fejervarya kawamurai* (Clade II) | *Fejervarya multistrata* | GZBY05 | Guangzhou |  |  | China | Verified | consistent assignment between GenBank annotation and species delimitation | Kotaki et al. (2010) |
| *Fejervarya multistriata* (Clade I) | *Fejervarya multistrata* | mtDNA | Santai County, Mianyang City, Sichuan | 31.178 | 105.13 | China | Verified | consistent assignment between GenBank annotation and species delimitation | Kung et al. (2008), direct submission |
| *Fejervarya multistriata* (Clade I) | *Fejervarya multistrata* | ZWd1339 | Chongming, Shanghai |  |  | China | Verified | consistent assignment between GenBank annotation and species delimitation | Kurniawan et al. (2010) |
| *Fejervarya multistriata* (Clade I) | *Fejervarya multistrata* | MZ44_MZ | Chongming, Shanghai |  |  | China | Verified | consistent assignment between GenBank annotation and species delimitation | Kurniawan et al. (2010) |
| *Fejervarya cancrivora* | *Fejervarya* cf. *cancrivora* | full mtDNA | Beihai, Guangxi |  |  | China | Verified | Supported to be split from *F. moodiei* in our species delimitation model | Kurniawan et al. (2010) |
| *Fejervarya cancrivora* | *Fejervarya* cf. *cancrivora* | MS-2009 (MS-can-Mal) | Selangor |  |  | Malaysia | Verified | Supported to be split from *F. moodiei* in our species delimitation model | Kotaki et al. (2010) |
| *Fejervarya moodiei* | *Fejervarya cancrivora* | Jiadong-13 | Jiadong |  |  | China | Verified | consistent assignment, supported to be split from *F. moodiei* in our species delimitation model | Kotaki et al. (2010) |
| *Fejervarya cancrivora* | *Fejervarya cancrivora* | FCL-16S-1 | Cianjur, Java |  |  | Indonesia | Verified | consistent assignment, supported to be split from *F. moodiei* in our species delimitation model | Chung et al. (2022) |
| *Fejervarya cancrivora* | *Fejervarya cancrivora* | FCL-16S-2 | West Sumatra, Padang |  |  | Indonesia | Verified | consistent assignment, supported to be split from *F. moodiei* in our species delimitation model | Chung et al. (2022) |
| *Fejervarya cancrivora* | *Fejervarya cancrivora* | FCL-16S-7 | Central Java, Banyumas |  |  | Indonesia | Verified | consistent assignment, supported to be split from *F. moodiei* in our species delimitation model | Chung et al. (2022) |
| *Fejervarya moodiei* | *Fejervarya moodiei* | IEBR A.5085 | Nam Dinh |  |  | Vietnam | Verified | Voucher verified from original published article, different in Genbank | Chung et al. (2022) |
| *Fejervarya moodiei* | *Fejervarya moodiei* | IEBR A.5089 | Nam Dinh |  |  | Vietnam | Verified | Voucher verified from original published article, different in Genbank | Hoang et al. (2022) |
| *Fejervarya moodiei* | *Fejervarya moodiei* | IEBR A.5091 | Nam Dinh |  |  | Vietnam | Verified | Voucher verified from original published article, different in Genbank | Hoang et al. (2022) |
| *Fejervarya moodiei* | *Fejervarya moodiei* | IEBR A.5097 | Thua Thien Hue |  |  | Vietnam | Verified | Voucher verified from original published article, different in Genbank | Hoang et al. (2022) |
| *Fejervarya moodiei* | *Fejervarya moodiei* | IEBR A.5096 | Thua Thien Hue |  |  | Vietnam | Verified | Voucher verified from original published article, different in Genbank | Hoang et al. (2022) |
| *Fejervarya moodiei* | *Fejervarya moodiei* | IEBR A.5098 | Hai Phong |  |  | Vietnam | Verified | Voucher verified from original published article, different in Genbank | Hoang et al. (2022) |
| *Fejervarya moodiei* | *Fejervarya moodiei* | IEBR A.5099 | Hai Phong |  |  | Vietnam | Verified | Voucher verified from original published article, different in Genbank | Hoang et al. (2022) |
| *Fejervarya moodiei* | *Fejervarya moodiei* | IEBR A.5101 | Ca Mau |  |  | Vietnam | Verified | Voucher verified from original published article, different in Genbank | Hoang et al. (2022) |
| *Fejervarya moodiei* | *Fejervarya moodiei* | CG9CaMau | Ca Mau |  |  | Vietnam | Verified | Voucher verified from original published article, different in Genbank | Hoang et al. (2022) |
| *Hoplobatrachus chinensis* | *Hoplobatrachus rugulosus* | full mtDNA | Jinhua, Zhejiang |  |  |  | Verified | Source of sample is farm-based, and verified in Zhejiang | Hoang et al. (2022) |
| *Hoplobatrachus chinensis* | *Hoplobatrachus rugulosus* | full mtDNA | Anhui |  |  |  | Verified | Source of sample is farm-based, and verified in Zhejiang | Yu et al. (2015) |
| *Hoplobatrachus chinensis* | *Hoplobatrachus chinensis x rugulosus* | full mtDNA | Jinhua, Zhejiang | 29°21′39 | 119°57′58 | China | Verified | Source of sample is farm-based, and verified in Zhejiang | Pan et al. (2012) |
| *Hoplobatrachus chinensis* | *Hoplobatrachus rugulosus* | JHBT-B4 | the Bendi farm in Jinhua, Zhejiang |  |  | China | Verified | Source of sample is farm-based, and verified in Zhejiang | Cai et al. (2018) |
| *Hoplobatrachus chinensis* | *Hoplobatrachus rugulosus* | JHBT-B3 | the Bendi farm in Jinhua, Zhejiang |  |  | China | Verified | Source of sample is farm-based, and verified in Zhejiang | Yu et al. (2015) |
| *Hoplobatrachus chinensis* | *Hoplobatrachus rugulosus* | JHBT-B2 | the Bendi farm in Jinhua, Zhejiang |  |  | China | Verified | Source of sample is farm-based, and verified in Zhejiang | Yu et al. (2015) |
| *Hoplobatrachus chinensis* | *Hoplobatrachus rugulosus* | JHBT-B1 | the Bendi farm in Jinhua, Zhejiang |  |  | China | Verified | Source of sample is farm-based, and verified in Zhejiang | Yu et al. (2015) |
| *Hoplobatrachus chinensis* | *Hoplobatrachus rugulosus* | FMNH 255191 | Laos: Champasak Province, Mounlapamok District, Dong Khanthung National Biodiversity Conservation Area |  |  | Laos | Verified | Source of voucher was cross checked with original article | Yu et al. (2015) |
| *Hoplobatrachus chinensis* | *Hoplobatrachus rugulosus* | HN1 | the 'Hainan' farm in Jinhua, Zhejiang |  |  | China | Verified | Source of sample is farm-based, and verified in Zhejiang | Frost et al. (2017) |
| *Hoplobatrachus chinensis* | *Hoplobatrachus rugulosus* | HN2 | the 'Hainan' farm in Jinhua, Zhejiang |  |  | China | Verified | Source of sample is farm-based, and verified in Zhejiang | Yu et al. (2015) |
| *Hoplobatrachus chinensis* | *Hoplobatrachus rugulosus* | HN3 | the 'Hainan' farm in Jinhua, Zhejiang |  |  | China | Verified | Source of sample is farm-based, and verified in Zhejiang | Yu et al. (2015) |
| *Hoplobatrachus chinensis* | *Hoplobatrachus rugulosus* | HW1 | the Huwawa farm in Jinhua, Zhejiang |  |  | China | Verified | Source of sample is farm-based, and verified in Zhejiang | Yu et al. (2015) |
| *Hoplobatrachus chinensis* | *Hoplobatrachus rugulosus* | HW5 | the Huwawa farm in Jinhua, Zhejiang |  |  | China | Verified | Source of sample is farm-based, and verified in Zhejiang | Yu et al. (2015) |
| *Hoplobatrachus chinensis* | *Hoplobatrachus rugulosus* | HW6 | the Huwawa farm in Jinhua, Zhejiang |  |  | China | Verified | Source of sample is farm-based, and verified in Zhejiang | Yu et al. (2015) |
| *Hoplobatrachus chinensis* | *Hoplobatrachus rugulosus* | THW1 | the Huwawa farm in Jinhua, Zhejiang |  |  | China | Verified | Source of sample is farm-based, and verified in Zhejiang | Yu et al. (2015) |
| *Hoplobatrachus chinensis* | *Hoplobatrachus rugulosus* | NW1 | the Niuwa farm in Jinhua, Zhejiang |  |  | China | Verified | Source of sample is farm-based, and verified in Zhejiang | Yu et al. (2015) |
| *Hoplobatrachus chinensis* | *Hoplobatrachus rugulosus* | NW2 | the Niuwa farm in Jinhua, Zhejiang |  |  | China | Verified | Source of sample is farm-based, and verified in Zhejiang | Yu et al. (2015) |
| *Hoplobatrachus chinensis* | *Hoplobatrachus rugulosus* | JHBT-W7 | the Wu farm in Jinhua, Zhejiang |  |  | China | Verified | Source of sample is farm-based, and verified in Zhejiang | Yu et al. (2015) |
| *Hoplobatrachus chinensis* | *Hoplobatrachus rugulosus* | JHBT-W8 | the Wu farm in Jinhua, Zhejiang |  |  | China | Verified | Source of sample is farm-based, and verified in Zhejiang | Yu et al. (2015) |
| *Hoplobatrachus chinensis* | *Hoplobatrachus rugulosus* | WZ2 | Jinhua, Zhejiang |  |  | China | Verified |  | Yu et al. (2015) |
| *Hoplobatrachus chinensis* | *Hoplobatrachus rugulosus* | WZ3 | Jinhua, Zhejiang |  |  | China | Verified |  | Alam et al. (2010) |
| *Hoplobatrachus tigerinus* | *Hoplobatrachus tigerinus* | full mtDNA | Mymensingh |  |  | Bangladesh | Verified |  | Yan et al. (2021) |
| *Quasipaa spinosa* | *Quasipaa spinosa* | KIZ021611 | Yuanbaoshan, Guangxi |  |  | China | Verified | Source of voucher was cross checked with original article | Yan et al. (2021) |
| *Quasipaa shini* | *Quasipaa shini* | KIZ021613 | Yuanbaoshan, Guangxi |  |  | China | Verified | Source of voucher was cross checked with original article | Yan et al. (2021) |
| *Quasipaa spinosa* | *Quasipaa spinosa* | KIZ022191 | Fulong, Guangxi |  |  | China | Verified | Source of voucher was cross checked with original article | Yan et al. (2021) |
| *Quasipaa yei* | *Quasipaa yei* | KIZ02340 | Shangcheng, Henan |  |  | China | Verified | Source of voucher was cross checked with original article | Yan et al. (2021) |
| *Quasipaa yei* | *Quasipaa yei* | KIZ02412 | Shangcheng, Henan |  |  | China | Verified | Source of voucher was cross checked with original article | Yan et al. (2021) |
| *Quasipaa boulengeri* | *Quasipaa boulengeri* | KIZ03107 | Youyang, Chongqing |  |  | China | Verified | Source of voucher was cross checked with original article | Yan et al. (2021) |
| *Quasipaa spinosa* | *Quasipaa spinosa* | KIZ04714 | Huizhou, Guangdong |  |  | China | Verified | Source of voucher was cross checked with original article | Yan et al. (2021) |
| *Quasipaa jiulongensis* | *Quasipaa jiulongensis* | KIZ08918 | Wuyishan, Fujian |  |  | China | Verified | Source of voucher was cross checked with original article | Yan et al. (2021) |
| *Quasipaa exilispinosa* | *Quasipaa exilispinosa* | KIZ08932 | Wuyishan, Fujian |  |  | China | Verified | Source of voucher was cross checked with original article | Yan et al. (2021) |
| *Quasipaa spinosa* | *Quasipaa spinosa* | KIZ08997 | Daiyunshan, Fujian |  |  | China | Verified | Source of voucher was cross checked with original article | Yan et al. (2021) |
| *Quasipaa spinosa* | *Quasipaa spinosa* | KIZ09096 | Qixingzhen, Guangdong |  |  | China | Verified | Source of voucher was cross checked with original article | Yan et al. (2021) |
| *Quasipaa spinosa* | *Quasipaa spinosa* | KIZ09282 | Chongzuo, Guangxi |  |  | China | Verified | Source of voucher was cross checked with original article | Yan et al. (2021) |
| *Quasipaa spinosa* | *Quasipaa spinosa* | KIZ09706 | Yangjiang, Guangdong |  |  | China | Verified | Source of voucher was cross checked with original article | Yan et al. (2021) |
| *Quasipaa spinosa* | *Quasipaa spinosa* | KIZJX0709008 | Lushan, Jiangxi |  |  | China | Verified | Source of voucher was cross checked with original article | Yan et al. (2021) |
| *Quasipaa spinosa* | *Quasipaa spinosa* | KIZYPX18607 | Xinying, Guangdong |  |  | China | Verified | Source of voucher was cross checked with original article | Yan et al. (2021) |
| *Quasipaa exilispinosa* | *Quasipaa exilispinosa* | KIZ01575 | Damaoshan, Hongkong |  |  | Hong Kong, Special Administrative Region (SAR) of China | Verified | Source of voucher was cross checked with original article | Yan et al. (2021) |
| *Quasipaa exilispinosa* | *Quasipaa exilispinosa* | KIZ07219 | Xunwu, Jiangxi |  |  | China | Verified | Source of voucher was cross checked with original article | Yan et al. (2021) |
| *Quasipaa exilispinosa* | *Quasipaa exilispinosa* | KIZ08934 | Wuyishan, Fujian |  |  | China | Verified | Source of voucher was cross checked with original article | Yan et al. (2021) |
| *Quasipaa exilispinosa* | *Quasipaa exilispinosa* | KIZ08935 | Wuyishan, Fujian |  |  | China | Verified | Source of voucher was cross checked with original article | Yan et al. (2021) |
| *Quasipaa exilispinosa* | *Quasipaa exilispinosa* | KIZ08936 | Wuyishan, Fujian |  |  | China | Verified | Source of voucher was cross checked with original article | Yan et al. (2021) |
| *Quasipaa exilispinosa* | *Quasipaa exilispinosa* | KIZ08938 | Wuyishan, Fujian |  |  | China | Verified | Source of voucher was cross checked with original article | Yan et al. (2021) |
| *Quasipaa jiulongensis* | *Quasipaa jiulongensis* | KIZ08920 | Wuyishan, Fujian |  |  | China | Verified | Source of voucher was cross checked with original article | Yan et al. (2021) |
| *Quasipaa shini* | *Quasipaa shini* | KIZ02397 | Kaili, Guizhou |  |  | China | Verified | Source of voucher was cross checked with original article | Yan et al. (2021) |
| *Quasipaa spinosa* | *Quasipaa spinosa* | KIZ09282 | Chongzuo, Guangxi |  |  | China | Verified | Source of voucher was cross checked with original article | Yan et al. (2021) |
| *Quasipaa spinosa* | *Quasipaa spinosa* | KIZC21 | Huangshanpo, Anhui |  |  | China | Verified | Source of voucher was cross checked with original article | Yan et al. (2021) |
| *Quasipaa spinosa* | *Quasipaa spinosa* | KIZ021611 | China: Yuanbaoshan, Guangxi |  |  | China | Verified | Source of voucher was cross checked with original article | Yan et al. (2021) |
| *Fejervarya kawamurai* (Clade I) | *Fejervarya kawamurai* | HiroshimaShiga | Shiga, Japan |  |  | Japan | Verified | Source of voucher was cross checked with original article | Yan et al. (2021) |
| *Fejervarya kawamurai* (Clade I) | *Fejervarya kawamurai* | Karasuma | Shiga, Japan |  |  | Japan | Verified | Source of voucher was cross checked with original article | Kakehashi et al. (2022) |
| *Fejervarya kawamurai* (Clade I) | *Fejervarya limnocharis* | JL02_1 | Chubu, Shizuoka Prefecture, Central Region, Shizuoka City, Shizuoka | 34.9884 | 138.299 | Japan | Verified | Source of voucher was cross checked with original article | Kakehashi et al. (2022) |
| *Fejervarya kawamurai* (Clade I) | *Fejervarya limnocharis* | Okin | Okinawa |  |  | Japan | Verified | Source of voucher was cross checked with original article | Kakehashi et al. (2022) |
| *Fejervarya kawamurai* (Clade I) | *Fejervarya limnocharis* | Okin_1_ | Okinawa |  |  | Japan | Verified | Source of voucher was cross checked with original article | Sumida et al. (2002) |
| *Fejervarya kawamurai* (Clade I) | *Fejervarya limnocharis* | Okin_2_ | Okinawa, Japan |  |  | Japan | Verified | Source of voucher was cross checked with original article | Sumida et al. (2002) |
| *Fejervarya kawamurai* (Clade I) | *Fejervarya limnocharis* | japo_higa | Higasihiroshima, Kagamiyama, Japan |  |  | Japan | Verified | Source of voucher was cross checked with original article | Sumida et al. (2002) |
| *Fejervarya kawamurai* (Clade I) | *Fejervarya limnocharis* | japo_hiro | Hiroshima, Japan |  |  | Japan | Verified | Source of voucher was cross checked with original article | Djong et al. (2007) |
| *Fejervarya kawamurai* (Clade I) | *Fejervarya* sp. | Hiroshima_1 | Hiroshima, Japan |  |  | Japan | Verified | Source of voucher was cross checked with original article | Djong et al. (2007) |
| *Fejervarya multistriata* (Clade II) | *Fejervarya limnocharis* | G024 | Hainan Island, China |  |  | China | Verified | Source of voucher was cross checked with original article | Sumida et al. (2002) |
| *Fejervarya multistriata* (Clade II) | *Fejervarya multistriata* | HS12001 | Santai County, Mianyang City, Sichuan province, China | 31.1782 | 105.1305 | China | Verified | Source of voucher was cross checked with original article | Grosjean et al. (2015) |
| *Fejervarya multistriata* (Clade II) | *Fejervarya multistriata* | Husa | Husa, China |  |  | China | Verified | GPS is not available but vouchers and locality were cross-checked against their original published sources | Jiang et al. (2020) |
| *Fejervarya multistriata* (Clade II) | *Fejervarya multistriata* | zjbj3 | Ji’an city, Jiangxi Province, China |  |  | China | Verified | GPS is not available but vouchers and locality were cross-checked against their original published sources | Kotaki et al. (2010) |
| *Fejervarya limnocharis* (Clade I) | *Fejervarya limnocharis* | 16SF15 | West Kalimantan |  |  | Indonesia | Verified | GPS is not available but vouchers and locality were cross-checked against their original published sources | Huang and Tu (2016) |
| *Fejervarya limnocharis* (Clade I) | *Fejervarya limnocharis* | 16SSG18 | Sabah, Borneo |  |  | Malaysia | Verified | GPS is not available but vouchers and locality were cross-checked against their original published sources | Goutte et al. (2016) |
| *Fejervarya limnocharis* (Clade I) | *Fejervarya limnocharis* | FMNH 261984 | *No exact locality but verified ito be originated from Laos | |  | Laos | Verified | GPS is not available but vouchers and locality were cross-checked against their original published sources | Inger and Stuart (2010) |
| *Fejervarya limnocharis* (Clade I) | *Fejervarya limnocharis* | MVZ226347 | Southern Chinese Mainland |  |  | China | Verified | GPS is not available but vouchers and locality were cross-checked against their original published sources | Che et al. (2010) |
| *Fejervarya limnocharis* (Clade I) | *Fejervarya limnocharis* | Malay | Peninsular Malaysia |  |  | Malaysia | Verified | GPS is not available but vouchers and locality were cross-checked against their original published sources | Kotaki et al. (2008) |
| *Fejervarya limnocharis* (Clade I) | *Fejervarya limnocharis* | NA0083 | Sarawak |  |  | Malaysia | Verified | GPS is not available but vouchers and locality were cross-checked against their original published sources | Zainuddin et al. (2008) |
| *Fejervarya limnocharis* (Clade I) | *Fejervarya limnocharis* | NA0084 | Sarawak |  |  | Malaysia | Verified | GPS is not available but vouchers and locality were cross-checked against their original published sources | Zainuddin et al. (2008) |
| *Fejervarya limnocharis* (Clade I) | *Fejervarya limnocharis* | Rano | Ranong |  |  | Thailand | Verified | GPS is not available but vouchers and locality were cross-checked against their original published sources | Che et al. (2016) |
| *Fejervarya limnocharis* (Clade I) | *Fejervarya limnocharis* | SCUM04H001 | Hainan Island |  |  | China | Verified | GPS is not available but vouchers and locality were cross-checked against their original published sources | Flury et al. (2021) |
| *Fejervarya limnocharis* (Clade I) | *Fejervarya limnocharis* | SH0034 | Borneo |  |  |  | Verified | GPS is not available but vouchers and locality were cross-checked against their original published sources | Zainuddin et al. (2008) |
| *Fejervarya limnocharis* (Clade I) | *Fejervarya limnocharis* | SNP0261 | Sarawak, Malaysia |  |  |  | Verified | GPS is not available but vouchers and locality were cross-checked against their original published sources | Inger and Stuart (2010) |
| *Fejervarya limnocharis* (Clade I) | *Fejervarya limnocharis* | Thai 1 | Nakhon Si Thammarat, Thailand |  |  |  | Verified | GPS is not available but vouchers and locality were cross-checked against their original published sources | Kotaki et al. (2008) |
| *Fejervarya limnocharis* (Clade I) | *Fejervarya limnocharis* | Thai 5 | Sanam Chaiket, Thailand |  |  |  | Verified | GPS is not available but vouchers and locality were cross-checked against their original published sources | Kotaki et al. (2008) |
| *Fejervarya limnocharis* (Clade I) | *Fejervarya limnocharis* | Thai 6 | Bangkok, Thailand |  |  |  | Verified | GPS is not available but vouchers and locality were cross-checked against their original published sources | Kotaki et al. (2008) |
| *Fejervarya limnocharis* (Clade I) | *Fejervarya limnocharis* | Thai 7 | Sara Buri, Thailand |  |  |  | Verified | GPS is not available but vouchers and locality were cross-checked against their original published sources | Kotaki et al. (2008) |
| *Fejervarya limnocharis* (Clade I) | *Fejervarya limnocharis* | SH13_027 | Borneo |  |  |  | Verified | GPS is not available but vouchers and locality were cross-checked against their original published sources | Flury et al. (2022) |
| *Fejervarya limnocharis* (Clade I) | *Fejervarya* cf. *limnocharis* | lim 1 | Java, Indonesia |  |  |  | Verified | GPS is not available but vouchers and locality were cross-checked against their original published sources | Kotaki et al. (2010) |
| *Fejervarya limnocharis* (Clade I) | *Fejervarya* cf. *limnocharis* | lim 10 | Sapa, Vietnam |  |  |  | Verified | GPS is not available but vouchers and locality were cross-checked against their original published sources | Flurry et al. (2021) |
| *Fejervarya limnocharis* (Clade I) | *Fejervarya* cf. *limnocharis* | lim 11 | Phongsaly, Laos |  |  |  | Verified | GPS is not available but vouchers and locality were cross-checked against their original published sources | Kotaki et al. (2010) |
| *Fejervarya limnocharis* (Clade I) | *Fejervarya* cf. *limnocharis* | lim 12 | Cambodia |  |  |  | Verified | GPS is not available but vouchers and locality were cross-checked against their original published sources | Kotaki et al. (2010) |
| *Fejervarya limnocharis* (Clade I) | *Fejervarya* cf. *limnocharis* | lim 2 | Java, Indonesia |  |  |  | Verified | GPS is not available but vouchers and locality were cross-checked against their original published sources | Kotaki et al. (2010) |
| *Fejervarya limnocharis* (Clade I) | *Fejervarya* cf. *limnocharis* | lim 3 | Kuala Lumpur, Malaysia |  |  |  | Verified | GPS is not available but vouchers and locality were cross-checked against their original published sources | Kotaki et al. (2010) |
| *Fejervarya limnocharis* (Clade I) | *Fejervarya* cf. *limnocharis* | lim 4 | Kuala Lumpur, Malaysia |  |  |  | Verified | GPS is not available but vouchers and locality were cross-checked against their original published sources | Kotaki et al. (2010) |
| *Fejervarya limnocharis* (Clade I) | *Fejervarya* cf. *limnocharis* | lim 5 | Ranong, Thailand |  |  |  | Verified | GPS is not available but vouchers and locality were cross-checked against their original published sources | Kotaki et al. (2010) |
| *Fejervarya limnocharis* (Clade I) | *Fejervarya* cf. *limnocharis* | lim 6 | Tha Ton, Thailand |  |  |  | Verified | GPS is not available but vouchers and locality were cross-checked against their original published sources | Kotaki et al. (2010) |
| *Fejervarya limnocharis* (Clade I) | *Fejervarya* cf. *limnocharis* | lim 8 | Phang Nga, Thailand |  |  |  | Verified | GPS is not available but vouchers and locality were cross-checked against their original published sources | Kotaki et al. (2010) |
| *Fejervarya limnocharis* (Clade I) | *Fejervarya* cf. *limnocharis* | lim 9 | Phang Nga, Thailand |  |  |  | Verified | GPS is not available but vouchers and locality were cross-checked against their original published sources | Kotaki et al. (2010) |
| *Fejervarya limnocharis* (Clade I) | *Fejervarya* cf. *limnocharis* | limno | Java, Indonesia |  |  |  | Verified | GPS is not available but vouchers and locality were cross-checked against their original published sources | Kotaki et al. (2010) |

**S4 Table. Information of universal primers used for PCR amplification of selected gene fragments.** The annealing temperature for each primer was adjusted according to the PCR conditions.

| **Fragment** | **Primer** | **5'-3'** | **Estimate length (base pairs)** | **Source** | **PCR condition** |
| --- | --- | --- | --- | --- | --- |
| D-loop/ Control region (*CR*) | 327-L | CTGTCCATATCATGACTACTTG | 519-bp | Zhong et al. (2008) | 95 ◦C for 3 min, followed by 35 cycles of 95 ◦C for 30 s, 50 ◦C-60 ◦C (gradient condition) for 30 s and 72 ◦C for 40 s, and a final elongation at 72 ◦C for 7 min |
|  | 885-H | GGTCTTAGCTTGTAGAGAGGTC | 519-bp | Zhong et al. (2008) |  |
| Cytochrome b (Cyt*b*) | Glu-f | GAC TCT AAC CTG GAC CAA TAG | 500 bp | Zhang et al., (2008) | 95°C for 4 min,  35 cycles of 95°C for 40 s, 50°C for 45 s, 72°C for 1 min, and 1 cycle of 72°C for 7 min. |
|  | contr5'r | TAA ATT TAT GCT CTA TAC A | 500 bp | Zhang et al., (2008) |  |
| 12S rRNA | FS01 | AACGCTAAGATGAACCCTAAAAAGTTCT | 422 bp | Sumida et al., (2002) | 95 ◦C for 5 min, 30 cycles, of 94 °C at 1 min,  55 °C for 1 min, 72 °C for 1 min and a final elongation at 72 ◦C for 7 min |
|  | R16M1 | GGGTATCTAATCCCAGTTTG | 422 bp | Sumida et al., (2002) |  |
| 16S rRNA | F51 | CCCGCCTGTTTACCAAAAACAT | 566 bp | Sumida et al., (2002) | 95 ◦C for 5 min, 30 cycles, of 94 °C at 1 min,  55 °C for 1 min, 72 °C for 1 min and a final elongation at 72 ◦C for 7 min |
|  | R51 | GGTCTGAACTCAGATCACGTA | 566 bp | Sumida et al., (2002) |  |
| *RAG-1* | RAG1_Ex1_Fow1 | AAATWCTCRGAMTGGAAGTTYAARCT | 900 bp | Kotaki et al., (2008) | 95 ◦C for 5 min, followed by 35 cycles of 94 ◦C for 30 s, 50 ◦C-60 ◦C (gradient condition) for 30 s and 72 ◦C for 1 min, and a final elongation at 72 ◦C for 10 min |
|  | RAG1_Ex1_Rev1 | TCACCWYCTTCTTCYTTBTCDGCRAA | 900 bp | Kotaki et al., (2008) |  |
| *TYR* | Tyr 1A | AGGTCCTCTTRAGCAAGGAATG | 530 bp | Bossuyt and Milinkovitch, (2000) | 95 ◦C for 5 min, followed by 35 cycles of 94 ◦C for 30 s, 50 ◦C-60 ◦C (gradient condition) for 30 s and 72 ◦C for 1 min, and a final elongation at 72 ◦C for 7 min |
|  | Tyr 1E | GAGAAGAAAGAWGCTGGGCTGAG | 530 bp | Bossuyt and Milinkovitch, (2000) |  |
| Rhodopsin (*Rho*) | Rhod1A | ACCATGAACGGAACAGAAGGYCC | 316 bp | Bossuyt and Milinkovitch, (2000) | 95 ◦C for 5 min, followed by 35 cycles of 94 ◦C for 30 s, 50 ◦C-60 ◦C (gradient condition) for 30 s and 72 ◦C for 1 min, and a final elongation at 72 ◦C for 7 min |
|  | Rhod1C | CCAAGGGTAGCGAAGAARCCTTC | 316 bp | Bossuyt and Milinkovitch, (2000) |  |

**S5 Table. Summary of the 18 species delimitation scenarios (Pre1–Pre11, Rev1–Rev5, A1, and C1) tested in this study.** For each scenario, the table lists the proposed clade composition, number of taxa, putative species count, and guiding factor(s) used in model formulation. Guiding factors are classified as (i) biogeographic allopatric scenarios, (ii) latitudinal climatic gradients, or (iii) evolutionary processes (homology/homoplasy). Models Pre1–Pre11 were generated from multilocus datasets as preliminary scenarios, Rev1–Rev5 represent revised scenarios incorporating expanded taxon sampling and genome-wide calibration, A1 is the advanced final model, and C1 is the control model based on uncorrected GenBank assignments.

| **Scenario testing**  **(Clades of interest)** | **Model** | **Species delimitation scenario** |
| --- | --- | --- |
| (a) *F. kawamurai, F. multistriata*, and *F. limnocharis* complexes.  (b) *Hoplobatrachus chinensis* x *H. rugulosus* | Pre1 | (a) Uniting *F. limnocharis* and *F. multistriata* species complexes into a single unit and assigning *Fejervarya* distributed in the Jiangsu plains to *F. limnocharis.* |
|  |  | (b) Splitting *F. kawamurai* into two units: a Japanese Archipelago clade vs. a Chinese Mainland clade. |
|  |  | (c) Splitting *Hoplobatrachus* distributed in East Asian mainland into two units: a clade of *H. rugulosus* vs. a hybrid clade of *H. rugulosus* x *H. chinensis.* |
| (a) *F. kawamurai, F. multistriata*, and *F. limnocharis* complexes.  (b) *Hoplobatrachus chinensis* x *H. rugulosus* | Pre2 | (a) Uniting *F. limnocharis* and *F. multistriata* species complexes into a single unit and assigning *Fejervarya* distributed in the Jiangsu plains to *F. multistriata.* |
|  |  | (b) Splitting *F. kawamurai* into two units: Southeastern coastal + Zhoushan and Japanese Archipelagos vs. Central and Northern China. |
|  |  | (c) Splitting *Hoplobatrachus* distributed on the East Asian mainland into two units: a clade of *H. rugulosus* vs. a hybrid clade of *H. rugulosus* x *H. chinensis.* |
| (a) *F. kawamurai, F. multistriata*, and *F. limnocharis* complexes.  (b) *Hoplobatrachus chinensis* x *H. rugulosus* | Pre3 | (a) Splitting *F. kawamurai* into two units: Southeastern coastal + Zhoushan and Japanese Archipelagos vs. *Fejervarya* clade distributed across Central China and northern Yangtze River (from Jiangsu plains to Northern China). |
|  |  | (b) Splitting *F. limnocharis* into two units: Indomalaya mainland and Sundaland vs. Taiwan Island. |
|  |  | (c) Splitting two units of *F. multistriata*: East Asian mainland vs. insular East Asia. |
|  |  | (d) Uniting the *Hoplobatrachus* distributed in East Asian mainland into the single unit of *H. rugulosus* |
| (a) *F. kawamurai, F. multistriata*, and *F. limnocharis* complexes.  (b) *Hoplobatrachus chinensis* x *H. rugulosus* | Pre4 | (a) Splitting *F. kawamurai* into three units: Zhoushan and Japanese archipelagos clade vs. central and northern China clade vs. an independent clade distributed in Jiangsu Plain. |
|  |  | (b) Splitting *F. limnocharis* into three units: Taiwan Island vs. Sundaland vs. Indomalayan mainland (continental Southeast Asia). |
|  |  | (c) Splitting *F. multistriata* into two units: East Asian mainland vs. East Asian insular clades. |
|  |  | (d) Uniting *H. rugulosus* and the hybrid clade of *H. chinensis* x *H. rugulosus* distributed across the East Asian mainland into a single unit. |
| (a) *F. kawamurai, F. multistriata*, and *F. limnocharis* complexes.  (b) *Hoplobatrachus chinensis* x *H. rugulosus* | Pre5 | (a) Uniting *F. kawamurai* into a unit, merging clade originated from Jiangsu Plains within Southern Yangtze and Japanese Archipelago *F. kawamurai*. |
|  |  | (b) Splitting *F. limnocharis* into three clades; Taiwan Island, Sundaland and Myanmar. |
|  |  | (c) Splitting *F. multistriata* into two units: an East Asian Mainland vs. East Asian insular clade. |
|  |  | (d) A unit of *F. sakishimensis* restricted to the Ryukyu range. |
|  |  | (e) Uniting *H. chinensis* x *H. rugulosus* hybrid clade with pure *H. rugulosus* clade into a single unit in East Asian mainland. |
| (a) *F. kawamurai, F. multistriata*, and *F. limnocharis* complexes.  (b) *Hoplobatrachus chinensis* x *H. rugulosus* | Pre6 | (a) Splitting *F. kawamurai* into two units: a clade restricted to the Japanese Archipelago vs. a clade restricted to the East Asian mainland, with *Fejervarya* distributed in the Jiangsu plains assigned to this clade. |
|  |  | (b) Splitting *F. limnocharis* into three units: a clade on the Indomalayan mainland vs. a clade limited to Sundaland vs. a clade restricted to Taiwan Island. |
|  |  | (c) Splitting *F. multistriata* into two units: comprising a clade of Insular East Asia vs. a clade on the East Asian mainland. |
|  |  | (d) A unit of *F. sakishimensis* restricted to Ryukyu range. |
|  |  | (e) A unit of *F. cancrivora* across the Indomalayan Ecozone. |
|  |  | (f) Uniting *H. chinensis* x *H. rugulosus* hybrid clade with a pure *H. rugulosus* clade into a single unit on the East Asian mainland. |
| (a) *F. kawamurai, F. multistriata*, and *F. limnocharis* complexes.  (b) *Hoplobatrachus chinensis* x *H. rugulosus* | Pre7 | (a) Splitting *F. kawamurai* into three units: a clade restricted to Japanese Archipelago vs. a clade restricted to the Zhoushan Archipelago and coastal areas in Southeastern China vs. a clade distributed in central and northern China, with *Fejervarya* distributed in Jiangsu plains was assigned within this clade. |
|  |  | (b) Splitting *F. limnocharis* into three units: a widespread clade distributed across Indomalayan mainland vs. a clade restricted to Sundaland vs. a clade restricted to Taiwan Island. |
|  |  | (c) Splitting *F. multistriata* into two units: a clade restricted to insular East Asia vs. a widespread clade distributed in East Asian mainland. |
|  |  | (d) A unit of *F. sakishimensis* restricted to Ryukyu. |
|  |  | (e) A unit of *F. cancrivora* across the Indomalaya Ecozone. |
|  |  | (f) Uniting *H. chinensis* x *H. rugulosus* hybrid clade with pure *H. rugulosus* clade into a single unit in East Asian mainland. |
| (a) *F. kawamurai, F. multistriata*, and *F. limnocharis* complexes.  (b) *Hoplobatrachus chinensis* x *H. rugulosus* | Pre8 | a) Merging all *F. multistriata* into a single unit; |
|  |  | (b) Splitting *F. kawamurai* into two units of the Zhoushan and Japanese archipelagos vs. Central and northern mainland of China. |
|  |  | (c)Splitting *F. limnocharis* into three units: Taiwan Island vs. Sundaland vs. Southeast Asian mainland and southwestern China. |
|  |  | (d) a unit of *F. cancrivora*. |
|  |  | (e) A unit of *F. sakishimensis* |
| (a) *F. kawamurai, F. multistriata*, and *F. limnocharis* complexes.  (b) *Hoplobatrachus chinensis* x *H. rugulosus* | Pre9 | Splitting all species complexes into two units. |
|  |  | (a) Splitting *F. multistriata* complex into two units: a clade distributed in Taiwan Island vs. a clade distributed in the East Asian mainland. |
|  |  | (b) Splitting *F. limnocharis* complex into two units; a clade restricted to Taiwan Island and the Ryukyu Archipelagos vs. a clade distributed from Sundaland to mainland Ssoutheast Asia. |
|  |  | (c) Splitting *F. kawamurai* into two units: a clade distributed from coastal areas of southeastern China, the Zhoushan and Japanese Archipelagos vs. a clade distributed across the central and northern Chinese mainland. |
|  |  | (d) Uniting *H. chinensis* x *H. rugulosus* hybrid clade with pure *H. rugulosus* clade into a single unit in East Asian mainland. |
| (a) *F. kawamurai, F. multistriata*, and *F. limnocharis* complexes. | Pre10 | (a) Merging *F. limnocharis* distributed across East and Southeast Asian Mainland and Sundaland into one unit. |
|  |  | (b) Splitting *F. kawamurai* into two units: the Zhoushan and Japanese Archipelagos clade vs. central and northern China clade, with the Jiangsu population assigned under this clade. |
|  |  | (c) Splitting *F. multistriata* into two units: insular clades (Hainan and Taiwan) vs. the Chinese Mainland. |
|  |  | (d) Uniting *H. chinensis* x *H. rugulosus* hybrid clade with pure *H. rugulosus* clade into a single unit in mainland East Asia. |
| (a) *F. kawamurai, F. multistriata*, and *F. limnocharis* complexes. (b) *Hoplobatrachus chinensis* x *H. rugulosus* | Pre11 | (a) Splitting *F. multistriata* into three units: a clade distributed in Taiwan-Ryukyu Archipelagos vs. a clade distributed in Eastern and Central China vs. a clade distributed in northeastern coastal China. |
|  |  | (b) Splitting *F. kawamurai* into two units: Zhoushan and Japanese Archipelagos clade vs. Central and Northern China clade, with Jiangsu population included within this clade. |
|  |  | (c) Splitting *F. limnocharis* into two units: a widespread clade distributed across the Southeastern Asian mainland vs. a clade limited to Sundaland. |
|  |  | (d) Uniting *H. chinensis* x *H. rugulosus* hybrid clade with pure *H. rugulosus* clade into a single unit in East Asian mainland. |
| *F. sakishimensis* | Rev1 | A unit of *F. sakishimensis* restricted to Ryukyu Islands. |
| *F. sakishimensis* | Rev2 | A unit of *F. sakishimensis* distributed in the Ryukyu Archipelagos and Taiwan Island. |
| *Fejervarya cancrivora* complex | Rev3 | (a) Uniting *F. cancrivora* into a single unit contained *F. cancrivora* distributed in Sundaland and *Fejervarya* population distributed in Hainan and Southern China (presumed to be an introduced population of *F. cancrivora*). |
|  |  | (b) A single clade of *F. moodiei* distributed across the Southeast Asian mainland. |
| *Fejervarya cancrivora complex* | Rev4 | (a) Splitting *F. cancrivora* into three units: a clade of ‘*F. moodiei*’ distributed in South East Asian mainland vs. a clade of *F. cancrivora* restricted to Sundaland vs. a clade of *Fejervarya* sp. distributed in Hainan and Southern China (assigned as an independent unit). |
| *Fejervarya cancrivora complex* | Rev5 | (a) Splitting *F. cancrivora* into two units: a clade of *F. moodiei* distributed across the Southeast Asian mainland, including Fejervarya population distributed in Hainan and Southern China vs. a clade of *F. cancrivora* restricted to Sundaland. |
| Optimal model* | A1 | (a) Splitting F. limnocharis into two units: South East Asian mainland vs. Sundaland clades. |
|  |  | (b) Splitting *F. multistriata* into two units: a clade distributed in Chinese Mainland and Hainan Island vs. a clade distributed in Taiwan Island and some part of Ryukyu Islands such as Okinawa. |
|  |  | (c) Splitting *F. kawamurai* into two units: a clade distributed in Hainan Island, and from Central to Northern Yangtze (including Jiangsu Plains and Northern China) vs. a clade distributed to the Japanese Archipelago, Southeastern coastal of China (Guangzhou) and the Zhoushan Archipelago. |
|  |  | (d) A unit of *F. sakishimensis* distributed to Taiwan Island and Ryukyu Archipelagos. |
|  |  | (e)Splitting *F. cancrivora* into two units: a clade of *F. moodiei* distributed across the Southeast Asian mainland, including *Fejervarya* population distributed in Hainan and Southern China vs. a clade of *F. cancrivora* restricted to Sundaland. |
| Control model (Genbank assignment) | C1  (Control) | The default topology follows the current assignment in GenBank. |

**S6 Table.** **The rank of support for the 18 comparative species delimitation models of three genera of Dicroglossidae distributed across the astern Indomalaya and southeastern Palearctic.** The rank of support for the 18 models, derived from preliminary and final datasets, was determined based on Marginal Likelihood Error (MLE) and positive Bayes Factor (BF) values. These models were segregated into four main scenarios of species boundaries and clades arrangement (see the scenarios regarding the topologies in Fig. 1). Support for each model is ranked from largest to smallest MLE value. The best-supported model, indicated with (*), is used in Fig. 2 as the representative best-supported species tree.

| **Scenario testing**  **(Clades of interest)** | **Model** | **Marginal likelihood** | **sqrt (H/N)** | **Standard deviation (SD)** | **Information** | **Max ESS** | **Bayes Factor** | **Rank of support** |
| --- | --- | --- | --- | --- | --- | --- | --- | --- |
| (a) *F. kawamurai, F. multistriata*, and *F. limnocharis* complexes.  (b) *Hoplobatrachus chinensis* x *H. rugulosus* | Pre1 | -22294.35 | 10.386 | 10.551 | 1510.244 | 501.431 | -2149.41 | 10 |
| (a) *F. kawamurai, F. multistriata*, and *F. limnocharis* complexes.  (b) *Hoplobatrachus chinensis* x *H. rugulosus* | Pre2 | -22320.404 | 10.09 | 10.221 | 1425.419 | 714.115 | -52.108 | 11 |
| (a) *F. kawamurai, F. multistriata*, and *F. limnocharis* complexes.  (b) *Hoplobatrachus chinensis* x *H. rugulosus* | Pre3 | -20751.769 | 10.008 | 9.994 | 1402.156 | 813.887 | 14.926 | 1 |
| (a) *F. kawamurai, F. multistriata*, and *F. limnocharis* complexes.  (b) *Hoplobatrachus chinensis* x *H. rugulosus* | Pre4 | -20791.038 | 9.914 | 10.286 | 1376.022 | 1028.881 | -63.612 | 3 |
| (a) *F. kawamurai, F. multistriata*, and *F. limnocharis* complexes.  (b) *Hoplobatrachus chinensis* x *H. rugulosus* | Pre5 | -20930.209 | 10.496 | 10.477 | 1542.436 | 570.114 | -139.728 | 6 |
| (a) *F. kawamurai, F. multistriata*, and *F. limnocharis* complexes.  (b) *Hoplobatrachus chinensis* x *H. rugulosus* | Pre6 | -20977.189 | 10.734 | 10.728 | 1613.048 | 806.558 | -93.96 | 7 |
| (a) *F. kawamurai, F. multistriata*, and *F. limnocharis* complexes.  (b) *Hoplobatrachus chinensis* x *H. rugulosus* | Pre7 | -20838.234 | 10.226 | 10.098 | 1463.98 | 802.632 | -94.392 | 4 |
| (a) *F. kawamurai, F. multistriata*, and *F. limnocharis* complexes.  (b) *Hoplobatrachus chinensis* x *H. rugulosus* | Pre8 | -20759.232 | 9.992 | 10.131 | 1397.705 | 1014.617 | -14.926 | 2 |
| (a) *F. kawamurai, F. multistriata*, and *F. limnocharis* complexes.  (b) *Hoplobatrachus chinensis* x *H. rugulosus* | Pre9 | -21219.645 | 9.93 | 10 | 1380.506 | 855.834 | -428.316 | 9 |
| (a) *F. kawamurai, F. multistriata*, and *F. limnocharis* complexes.  (b) *Hoplobatrachus chinensis* x *H. rugulosus* | Pre10 | -20860.345 | 10.425 | 10.475 | 1521.6006 | 517.708 | -44.222 | 5 |
| (a) *F. kawamurai, F. multistriata*, and *F. limnocharis* complexes. (b) *Hoplobatrachus chinensis* x *H. rugulosus* | Pre11 | -21005.487 | 10.374 | 10.317 | 1506.614 | 822.678 | -56.596 | 8 |
| *F. sakishimensis* | Rev1 | -21817.09 | 10.225 | 10.194 | 1463.573 | 943.243 | -385.304 | 2 |
| *F. sakishimensis* | Rev2 | -21624.348 | 10.332 | 10.523 | 1494.525 | 801.261 | 385.304 | 1 |
| *Fejervarya cancrivora* complex | Rev3 | -21772.968 | 10.058 | 9.973 | 1416.255 | 1449.644 | -400.486 | 3 |
| *Fejervarya cancrivora* complex | Rev4 | -21572.725 | 10.206 | 10.171 | 1458.242 | 954.372 | -42.27 | 2 |
| *Fejervarya cancrivora* complex | Rev5 | -21551.59 | 10.154 | 10.256 | 1443.567 | 1019.487 | 42.27 | 1 |
| Optimal model* | A1 | -21521.624 | 10.221 | 10.288 | 1462.457 | 915.308 | 314.216 | 1 |
| Control model (Genbank assignment) | C1  (Control) | -21678.85 | 10.902 | 11.143 | 1664.068 | 733.858 | -314.216 | 2 |

**S7 Table. Metadata for the 33 genome-wide samples used in this study.** Columns indicate the Sequence Read Archive (SRA) accession, data source and type, geographic origin, species assignment under Model A1, and reference. These samples were merged to generate the final genome-wide dataset for Model A1.

| **Sequence Read Archive (SRA)** | **Source** | **Data type** | **Geographic origin** | **Species assignment in StarBEAST** | **Reference** |
| --- | --- | --- | --- | --- | --- |
| SRR11516790 | GBS | SNPs | Yan’e village, Dayi, Sichuan, China | *Q. boulengeri* | Yang et al., (2022) |
| SRR11516791 | GBS | SNPs | Yan’e village, Dayi, Sichuan, China | *Q. boulengeri* | Yang et al. (2022) |
| SRR11516793 | GBS | SNPs | Yan’e village, Dayi, Sichuan, China | *Q. boulengeri* | Yang et al. (2022) |
| SRR11516794 | GBS | SNPs | Yan’e village, Dayi, Sichuan, China | *Q. boulengeri* | Yang et al. (2022) |
| SRR11516795 | GBS | SNPs | Yan’e village, Dayi, Sichuan, China | *Q. boulengeri* | Yang et al. (2022) |
| SRR11516796 | GBS | SNPs | Yan’e village, Dayi, Sichuan, China | *Q. boulengeri* | Yang et al. (2022) |
| SRR11516801 | GBS | SNPs | Yan’e village, Dayi, Sichuan, China | *Q. boulengeri* | Yang et al. (2022) |
| SRR11516802 | GBS | SNPs | Yan’e village, Dayi, Sichuan, China | *Q. boulengeri* | Yang et al. (2022) |
| SRR1151680 | GBS | SNPs | Yan’e village, Dayi, Sichuan, China | *Q. boulengeri* | Yang et al. (2022) |
| SRR11516832 | GBS | SNPs | Yan’e village, Dayi, Sichuan, China | *Q. boulengeri* | Yang et al. (2022) |
| SRR4332936 | WGS/WCS | DNA-seq | Southern China | *F. kawamurai [Clade I]* | Kunming institute of Zoology, CAS, unpublished |
| SRR21116873 | WGS/WCS | DNA-seq | Peninsular Malaysia | *F. limnocharis [Clade I]* | Jahari et al., unpublished |
| SRR4332935 | WGS/WCS | DNA-seq | Southern China | *F. multistriata [Clade II]* | Kunming institute of Zoology |
| SRR3929655 | WGS/WCS | DNA-seq | Southern China | *Q. boulengeri* | Chengdu Institute of Biology, Chinese Academy of Sciences, unpublished |
| SRR4332938 | WGS/WCS | DNA-seq | Southern China | *Q. shini* | Kunming institute of Zoology, CAS, unpublished |
| SRR4332939 | WGS/WCS | DNA-seq | Eastern China | *Q. spinosa* | Kunming institute of Zoology, CAS, unpublished |
| SRR1553362 | RNA-seq | RNA-seq | Hainan Island, China | *F. moodiei* | Shao et al., (2015) |
| SRR1553365 | RNA-seq | RNA-seq | Hainan Island, China | *F. moodiei* | Shao et al. (2015) |
| SRR1553366 | RNA-seq | RNA-seq | Hainan Island, China | *F. moodiei* | Shao et al. (2015) |
| SRR1554295 | RNA-seq | RNA-seq | Hainan Island, China | *F. moodiei* | Shao et al. (2015) |
| DRR489649 | RNA-seq | RNA-seq | Mt. Futagami, Hiroshima, Japan (34°40’N, 132°70’E) | *F. kawamurai [Clade I]* | Lau et al., (2023) |
| SRR1554298 | RNA-seq | RNA-seq | Hainan Island, China | *F.limnocharis [Clade II]* | Shao et al. (2015) |
| SRR1554299 | RNA-seq | RNA-seq | Hainan Island, China | *F. limnocharis [Clade II]* | Shao et al. (2015) |
| SRS686785 | RNA-seq | RNA-seq | Hainan Island, China | *F.limnocharis [Clade II]* | Shao et al. (2015) |
| SRR18907483 | RNA-seq | RNA-seq | Hainan Island, China | *F. multistriata [Clade I]* | Shao et al. (2015) |
| SRR12516458 | RNA-seq | RNA-seq | Lishui University, Zhejiang | *H. rugulosus* | Tang et al., (2021) |
| SRR12516456 | RNA-seq | RNA-seq | Lishui University, Zhejiang | *H. rugulosus* | Tang et al. (2021) |
| SRR12516457 | RNA-seq | RNA-seq | Lishui University, Zhejiang | *H. rugulosus* | Tang et al. (2021) |
| SRR21148310 | RNA-seq | RNA-seq | Zunyi city, Guizhou Province, China | *Q. spinosa* | Wang et al., (2022) |
| SRR8648149 | RNA-seq | RNA-seq | Zunyi city, Guizhou Province, China | *Q.spinosa* | Wang et al. (2022) |
| SRR1554293 | RNA-seq | RNA-seq | Hainan Island, China | *F. limnocharis [Clade II]* | Shao et al. (2015) |
| SRR1554301 | RNA-seq | RNA-seq | Hainan Island, China | *F. limnocharis [Clade II]* | Shao et al. (2015) |
| SRR1554303 | RNA-seq | RNA-seq | Hainan Island, China | *F. limnocharis [Clade II]* | Shao et al. (2015) |

**S8 Table. Morphological characters available from the literature for *Fejervarya* species belonging to three major species complexes in the Southeastern Palearctic and Eastern Indomalaya.** The characters were scored in binary format, with “1” indicating the presence of a trait in a species and “0” indicating its absence, and subsequently converted to NEXUS format for integration into the StarBEAST dataset.

| Morphometric / Meristic trait | *F. kawamurai* | *F. sakishimensis* | *F. multistriata* | *F. moodiei* | *F. cancrivora* | *F. limnocharis* | Source |
| --- | --- | --- | --- | --- | --- | --- | --- |
| Small tympanum | 1 | 0 | 0 | 0 | 0 | 0 | Djong et al. (2011) |
| Small head | 1 | 0 | 0 | 0 | 0 | 0 | Djong et al. (2011) |
| Small and short forelimbs | 1 | 0 | 0 | 0 | 0 | 0 | Djong et al. (2011) |
| Small and short hindlimbs | 1 | 0 | 0 | 0 | 0 | 0 | Djong et al. (2011) |
| Small foot | 1 | 0 | 0 | 0 | 0 | 0 | Djong et al. (2011) |
| Short SVL | 1 | 0 | 0 | 0 | 0 | 0 | Djong et al. (2011) |
| Short tibia length | 1 | 0 | 0 | 0 | 0 | 0 | Djong et al. (2011) |
| Small eye length | 0 | 0 | 0 | 0 | 1 | 0 | Yodthong et al. (2019) |
| Small tympanum diameter | 0 | 0 | 0 | 0 | 1 | 0 | Yodthong et al. (2019) |
| Short nostril–tympanum distance | 0 | 0 | 0 | 0 | 1 | 0 | Yodthong et al. (2019) |
| SVL ♂ (mm) | 30.7–41.8 | 34–46 (Japan) | 35–48 | 44.5–74.9 | 58.6–77.2 | 37.8–46.2 | Djong et al. (2011); Köhler et al., (2019); Tong et al. (2025) |
| SVL ♀ (mm) | 36.8–48.7 | 38–50 (Japan) | 38–50 | 46.1–88.1 | 76.4–99.1 | 38.0–56.6 | Djong et al. (2011); Köhler et al., (2019); Tong et al. (2025) |
| Relative tibia length (SHL/SVL mean) | 0.43 | 0.54 | 0.50 | 0.52 | 0.52 | 0.48 | Köhler et al. (2019); Tong et al. (2025) |
| Relative foot length (FL/SVL mean) | 0.45 | 0.54 | 0.49 | 0.49 | 0.49 | 0.52 | Köhler et al. (2019); Tong et al. (2025) |
| Relative head width (HW/SVL mean) | 0.35 | 0.33 | 0.32 | 0.38 | 0.38 | 0.31 | Köhler et al. (2019); Tong et al. (2025) |
| Relative tympanum size (TYD/SVL mean) | 0.07 | 0.07 | 0.07 | 0.085 | 0.085 | 0.076 | Köhler et al. (2019); Tong et al. (2025) |
| Toe webbing formula (♂) | Weak | Moderate | Weak | Strong | Strong | Weak | Tong et al. (2025) |
| Toe webbing formula (♀) | Weak | Moderate | Weak | Strong | Strong | Weak | Tong et al. (2025) |
| Tubercles (metatarsal, subarticular) | Present | Present | Present | Strong | Strong | Weak | Tong et al. (2025) |

**S9 Table. Locus information and partitioning scheme used in StarBEAST under a Relaxed Lognormal Clock (RLC) model for species tree reconstruction.** The multilocus dataset was enriched with genome-wide data, including including RNA-seq-derived exons, SNP-based loci, and WGS data obtained from SRA entries in GenBank.

| **Modelling stage** | **Dataset** | **Locus** | **Ploidy** | **N taxa** | **Sites** | **data** |
| --- | --- | --- | --- | --- | --- | --- |
| Preliminary and revised | 1,2 & 3 | Cytochrome *b* | Haploid | 147 | 437 | nucleotide |
| Preliminary and revised | *1,2&3* | *RAG1* | Diploid | 81 | 849 | nucleotide |
| Preliminary and revised | 1,2&3 | Tyrosinase | Diploid | 109 | 524 | nucleotide |
| Preliminary and revised | 1,2&3 | 12S rRNA | Haploid | 72 | 420 | nucleotide |
| Preliminary and revised | 1,2&3 | Rhodopsin | Diploid | 102 | 279 | nucleotide |
| Revised | 2&3 | *COI* | Haploid | 59 | 578 | nucleotide |
| Preliminary and revised Preliminary and revised | 1,2&3 | Control region | Haploid | 120 | 476 | nucleotide |
| Preliminary and revised | 1,2&3 | 16S rRNA | Haploid | 136 | 481 | nucleotide |
| Final (refinement) | 3 | Genome-wide data using RNAseq exons and RADseq (147 split locus) | Diploid | 33 | 752,383 | nucleotide |

**S10 Table. Sample-level locus recovery and sequence completeness of SRA-derived genomic datasets retained for Model A1.** Sequencing data source indicates the original public dataset type (GBS, RNA-seq, or WGS/WCS). Missingness was calculated as the proportion of characters (coded as N or ?) across the genomic alignments retained for each sample. The alignment gaps (-) were not counted as missing characters.

| **Sample** | **SRA accession** | **Sequencing data source** | **Loci retained** | **Total alignment characters** | **Missing/ ambiguous characters (N/ ?)** | **Missingness (%)** | **Completeness (%)** |
| --- | --- | --- | --- | --- | --- | --- | --- |
| *Q. spinosa* | SRR21148310 | RNA-seq | 70 | 1,231 | 0 | 0 | 100 |
| *F. moodiei* | SRR1554295 | RNA-seq | 70 | 1,231 | 1 | 0.08 | 99.92 |
| *F. moodiei* | SRR1553366 | RNA-seq | 70 | 1,231 | 3 | 0.24 | 99.76 |
| *H. rugulosus* | SRR12516458 | RNA-seq | 70 | 1,231 | 5 | 0.41 | 99.59 |
| *F. limnocharis* | SRR1554298 | RNA-seq | 70 | 1,231 | 7 | 0.57 | 99.43 |
| *F. moodiei* | SRR1553365 | RNA-seq | 70 | 1,231 | 10 | 0.81 | 99.19 |
| *F. limnocharis* | SRR1554301 | RNA-seq | 70 | 1,231 | 13 | 1.06 | 98.94 |
| *F. limnocharis* | SRR1554293 | RNA-seq | 70 | 1,231 | 16 | 1.3 | 98.7 |
| *F. kawamurai* | DRR489649 | RNA-seq | 70 | 1,231 | 17 | 1.38 | 98.62 |
| *F. multistriata* | SRR18907483 | RNA-seq | 70 | 1,231 | 17 | 1.38 | 98.62 |
| *F. limnocharis* | SRR1554299 | RNA-seq | 70 | 1,231 | 19 | 1.54 | 98.46 |
| *H. rugulosus* | SRR12516457 | RNA-seq | 70 | 1,231 | 21 | 1.71 | 98.29 |
| *Q. spinosa* | SRR8648149 | RNA-seq | 70 | 1,231 | 22 | 1.79 | 98.21 |
| *F. limnocharis* | SRS686785 | RNA-seq | 70 | 1,231 | 23 | 1.87 | 98.13 |
| *H. rugulosus* | SRR12516456 | RNA-seq | 70 | 1,231 | 29 | 2.36 | 97.64 |
| *F. limnocharis* | SRR1554303 | RNA-seq | 70 | 1,231 | 38 | 3.09 | 96.91 |
| *F. moodiei* | SRR1553362 | RNA-seq | 70 | 1,231 | 46 | 3.74 | 96.26 |
| *Q. boulengeri* | SRR11516796 | GBS | 64 | 1,203 | 22 | 1.83 | 98.17 |
| *Q. boulengeri* | SRR11516791 | GBS | 64 | 1,203 | 24 | 2 | 98 |
| *Q. boulengeri* | SRR11516795 | GBS | 64 | 1,203 | 49 | 4.07 | 95.93 |
| *Q. boulengeri* | SRR11516802 | GBS | 64 | 1,203 | 54 | 4.49 | 95.51 |
| *Q. boulengeri* | SRR11516794 | GBS | 64 | 1,203 | 60 | 4.99 | 95.01 |
| *Q. boulengeri* | SRR11516832 | GBS | 64 | 1,203 | 80 | 6.65 | 93.35 |
| *Q. boulengeri* | SRR11516793 | GBS | 64 | 1,203 | 93 | 7.73 | 92.27 |
| *Q. boulengeri* | SRR11516790 | GBS | 64 | 1,203 | 94 | 7.81 | 92.19 |
| *Q. boulengeri* | SRR11516801 | GBS | 64 | 1,203 | 121 | 10.06 | 89.94 |
| *Q. boulengeri* | SRR11516803 | GBS | 64 | 1,203 | 154 | 12.8 | 87.2 |
| *F. kawamurai* | SRR4332936 | WGS/WCS | 13 | 505 | 0 | 0 | 100 |
| *F. limnocharis* | SRR21116873 | WGS/WCS | 13 | 505 | 0 | 0 | 100 |
| *F. multistriata* | SRR4332935 | WGS/WCS | 13 | 505 | 0 | 0 | 100 |
| *Q. boulengeri* | SRR3929655 | WGS/WCS | 13 | 505 | 0 | 0 | 100 |
| *Q. shini* | SRR4332938 | WGS/WCS | 13 | 505 | 0 | 0 | 100 |
| *Q. spinosa* | SRR4332939 | WGS/WCS | 13 | 505 | 0 | 0 | 100 |

**S11 Table.** **Contribution of environment variables for the prediction of species occurrences**. Percent contribution of environment variables for *Fejervarya multistriata* using presence-only data for sampling assumption in the modelling with minPred and MTSS thresholds, respectively.

| **Model prediction of species occurrence** | **Variable** | **Percent contribution** | **Permutation importance** |
| --- | --- | --- | --- |
| MinPred thresholding | Bio 14-precipitation of driest month | 59.8 | 17.8 |
|  | Bio 2-mean diurnal range | 20 | 10.7 |
|  | Elevation | 5 | 48.3 |
|  | Bio 8-mean temperature of wettest quarter | 3.9 | 5.9 |
|  | Bio 12-annual precipitation | 2.9 | 1.9 |
|  | Bio 5-maximum temperature of warmest month | 2.5 | 3.4 |
|  | Bio 10-mean temperature of warmest quarter | 1.9 | 0.4 |
|  | Bio 1-annual mean temperature | 1.9 | 2.4 |
|  | Bio 3-isothermality | 1.4 | 3.2 |
|  | Bio15-precipitation seasonality | 0.6 | 6.1 |
|  | Bio 14-precipitation of Driest Month | 58.3 | 25.2 |
| MTSS thresholding | Bio 2-Mean Diurnal Range | 20 | 17.5 |
|  | Elevation | 6.9 | 27.8 |
|  | Bio 1-Annual Mean Temperature | 3.6 | 1.2 |
|  | Bio 8-Mean Temperature of Wettest Quarter | 3.4 | 14.2 |
|  | Bio 12-Annual Precipitation | 3 | 1.7 |
|  | Bio 10-Mean Temperature of Warmest Quarter | 1.8 | 0 |
|  | Bio 5-Max Temperature of Warmest Month | 1.7 | 5.1 |
|  | Bio 3-Isothermality | 1.1 | 3.8 |
|  | Bio 15-Precipitation Seasonality | 0.3 | 3.5 |

**S12 Table.** **Contribution of environment variables for the prediction of species occurrences**. Percent contribution of environment variables for *Fejervarya kawamurai* using presence-only data for sampling assumption in the modelling with minPred and MTSS thresholds, respectively.

| **Presence-only data reliance** | **Variable** | **Percent contribution** | **Permutation importance** |
| --- | --- | --- | --- |
| MinPred thresholding | Bio 14 - Precipitation of driest month | 46.1 | 8.5 |
|  | Elevation | 34.2 | 65.5 |
|  | Bio 1 - Annual mean temperature | 7.6 | 0.9 |
|  | Bio 15 - Precipitation Seasonality | 5 | 5.1 |
|  | Bio 8 - Mean Temperature of Wettest Quarter | 2.7 | 4.5 |
|  | Bio 12 - Annual Precipitation | 2 | 13.4 |
|  | Bio 10 - Mean Temperature of Warmest Quarter | 1.8 | 0.8 |
|  | Bio 3 - Isothermality | 0.3 | 0.1 |
|  | Bio 2 - Mean Diurnal Range | 0.2 | 1.2 |
|  | Bio 5 - Max Temperature of Warmest Month | 0 | 0 |
| MTSS thresholding | Elevation | 71.8 | 9.1 |
|  | Bio 1 - Annual Mean Temperature | 9.8 | 12.4 |
|  | Bio 2 - Mean Diurnal Range | 5.8 | 26.2 |
|  | Bio 15 - Precipitation Seasonality | 5.2 | 6.4 |
|  | Bio 8 - Mean Temperature of Wettest Quarter | 3.7 | 33.6 |
|  | Bio 14 - Precipitation of Driest Month | 1.3 | 0.8 |
|  | Bio 3 - Isothermality | 1.2 | 3.1 |
|  | Bio 10 - Mean Temperature of Warmest Quarter | 0.9 | 6.9 |
|  | Bio 5 - Max Temperature of Warmest Month | 0.1 | 0 |
|  | Bio 12 - Annual Precipitation | 0.1 | 1.6 |

**S13 Table.** Mean call properties of two sympatric and cryptic *Fejervarya* clades. *Fejervarya kawamurai* from the Central Yangtze basin and *F. multistriata* from the Eastern Yangtze Basin in the Palearctic range.

| **Species** | **Lat** | **Lon** | **Locality** | **SVL (mm)** | **Mean call duration (s)** | **SE** | **Mean call interval (s)** | **SE** | **Mean call rate (call/min)** | **SE** | **Mean pulse rate (pulse/s)** | **SE** | **Mean dominant frequency (Hz)** | **SE** | **Temperature (^o^C)** |
| --- | --- | --- | --- | --- | --- | --- | --- | --- | --- | --- | --- | --- | --- | --- | --- |
| *Fejervarya kawamurai* | 32.0809 | 118.8135 | Xuanwu, Nanjing, Jiangsu | 44.430 | 0.109 | 0.001 | 0.130 | 0.017 | 299.607 | 22.966 | 87.645 | 0.603 | 2493.550 | 234.082 | 25.6 |
| *Fejervarya kawamurai* | 32.0809 | 118.8135 | Xuanwu, Nanjing, Jiangsu | 39.480 | 0.105 | 0.002 | 0.195 | 0.036 | 216.373 | 15.067 | 86.476 | 0.461 | 1391.030 | 6.580 | 25.6 |
| *Fejervarya kawamurai* | 32.0809 | 118.8135 | Xuanwu, Nanjing, Jiangsu | 41.400 | 0.079 | 0.003 | 0.292 | 0.025 | 167.002 | 9.530 | 89.346 | 0.320 | 1366.400 | 8.400 | 25.6 |
| *Fejervarya kawamurai* | 32.0809 | 118.8135 | Xuanwu, Nanjing, Jiangsu | 44.430 | 0.109 | 0.001 | 0.199 | 0.011 | 197.040 | 6.930 | 85.698 | 0.370 | 1292.000 | 0.000 | 25.6 |
| *Fejervarya kawamurai* | 32.0809 | 32.0809 | Xuanwu, Nanjing, Jiangsu | 42.000 | 0.102 | 0.005 | 0.155 | 0.011 | 237.7 | 7.000 | 111.300 | 4.000 | 1903.5 | 246 | 27.0 |
| *Fejervarya kawamurai* | 32.0809 | 32.0809 | Xuanwu, Nanjing, Jiangsu | 40.500 | 0.106 | 0.002 | 0.173 | 0.018 | 221.8 | 11.300 | 102.000 | 2.200 | 2696.000 | 220.1 | 26.0 |
| *Fejervarya multistriata* | 31.20416 | 121.38099 | Xujiashe,Shanghai | 38.990 | 0.203 | 0.075 | 0.104 | 0.030 | 213.701 | 2.210 | 78.625 | 0.903 | 1402.905 | 0.781 | 24.2 |
| *Fejervarya multistriata* | 31.2183778 | 121.5562527 | Xujiashe,Shanghai | 39.880 | 0.233 | 0.278 | 0.090 | 0.078 | 217.624 | 3.660 | 75.466 | 0.761 | 1487.876 | 6.276 | 23.5 |
| *Fejervarya multistriata* | 31.317475 | 121.5530 | Xujiashe,Shanghai | 41.330 | 0.328 | 0.742 | 0.085 | 0.030 | 230.512 | 7.060 | 76.792 | 0.638 | 2097.884 | 12.024 | 25.1 |
| *Fejervarya multistriata* | 31.3265306 | 121.298675 | Yangpu,  Shanghai | 40.510 | 0.209 | 0.369 | 0.086 | 0.049 | 241.626 | 3.630 | 73.005 | 0.446 | 1965.624 | 13.883 | 24.2 |
| *Fejervarya multistriata* | 31.46926 | 121.3378333 | Jiading, Shanghai | 41.180 | 0.207 | 0.218 | 0.117 | 0.117 | 215.607 | 3.680 | 69.483 | 1.281 | 1547.992 | 6.670 | 23.5 |
| *Fejervarya multistriata* | 31.1198306 | 121.229086 | Pudong (new area), Shanghai | 35.720 | 0.338 | 0.254 | 0.067 | 0.084 | 202.572 | 9.790 | 76.242 | 0.491 | 2402.628 | 11.127 | 22.3 |
| *Fejervarya multistriata* | 31.0391444 | 121.5067278 | Xuhui, Shanghai | 36.880 | 0.131 | 0.181 | 0.085 | 0.031 | 307.161 | 2.900 | 76.717 | 0.183 | 1492.674 | 3.636 | 23.5 |
| *Fejervarya multistriata* | 31.1367306 | 121.24076 | Hongkou, Shanghai | 36.920 | 0.161 | 0.130 | 0.091 | 0.054 | 266.368 | 4.010 | 76.541 | 0.283 | 2693.004 | 11.899 | 23.6 |
| *Fejervarya multistriata* | 31.050008 | 121.4870722 | Huangpu, Shanghai | 39.880 | 0.197 | 0.200 | 0.082 | 0.047 | 249.303 | 4.860 | 76.229 | 0.110 | 2162.531 | 11.714 | 23.2 |
| *Fejervarya multistriata* | 31.0883806 | 121.17945555 | Minhang, Shanghai | 38.020 | 0.258 | 0.370 | 0.076 | 0.037 | 217.646 | 4.080 | 69.493 | 0.579 | 1826.256 | 9.275 | 23.7 |
| *Fejervarya multistriata* | 31.0478944 | 121.133213 | Pudong (new area), Shanghai | 37.010 | 0.303 | 0.311 | 0.083 | 0.049 | 195.786 | 5.830 | 84.949 | 0.485 | 1910.648 | 14.339 | 26.1 |

**S14 Table. Bayes Factor comparisons performed between the final and control supermatrix topologies.** The marginal likelihood comparison and Bayes Factor were computed using path sampling method, based on curated genome-wide datasets retrieved from GenBank.

| **Supermatrix tree** | **Marginal likelihood estimation** | **bootstrap SD** | **subsample SD** | **Information** | **Standard deviation** | **Bayes Factor = e ^(ML  Final−ML Control)^** |
| --- | --- | --- | --- | --- | --- | --- |
| Control Model C1 with supermatrix genome-data | ML: -26753.096 (17.445) | 17.249 | 20.564 | 2850.937 | 16.885 | e ^-116.42^ |
| Refine Model A1 with supermatrix genome-data | ML: -26636.676 (15.463) | 16.579 | 20.338 | 2817.388 | 16.785 | e ^116.42^ |

**S15 Table.** **Divergence time estimates derived from the species tree of optimal Model A1 following refinement of calibrations and topology with genome-wide multilocus data.** The estimates include 95% Highest Posterior Density (HPD) intervals. Clades and nodes labelled from (a) to (n) correspond to those presented in the species tree (Fig. 3).

| **Calibration point** | **Speciation events** | | **support** | | **Mean (Mya)** | | **95% HPD (Mya)** | |
| --- | --- | --- | --- | --- | --- | --- | --- | --- |
| 1 | Split off between *H. chinensis* and *H. tigerinus* | 100 | | 18.544 | | 13.837-23.079 | |  |
| 0 | Split off between *F. limnocharis* in Sundaland (Clade I) and F. limnocharis in Southeast Asian mainland (Clade II) | 100 | | 15.78 | | 9.59-21.505 | |  |
| 0 | Stem clade of *F. limnocharis* complex, *F. sakishimensis*, and *F. cancrivora* complex | 17.13 | | 18.65 | | 12.70-25.18 | |  |
| 0 | Emergence of *F. cancrivora* + *F. moodiei* | 100 | | 13.74 | | 8.72-18.79.13 | |  |
| 1 | Basal emergence of *Fejervarya* in the Palearctic and Indomalaya | 100 | | 19.32 | | 12.60-25.18 | |  |
| 0 | Split off between *F. kawamurai* [Clade I] and [Clade II] | 100 | | 11.51 | | 3.73-18.05 | |  |
| 0 | *Fejervarya* clades emerged in southeastern Palearctic (containing *F. kawamurai* and *F. multistriata*) | 26.12 | | 13.36 | | 4.68-19.82 | |  |
| 0 | Split off between Clade I and Clade II of *F. multistriata* | 100 | | 6.28 | | 0 -18.45 | |  |
| 0 | Split off between Split *Q. jiulongensis* + *Q. shini* | 67 | | 27.20 | | 6.7-41.42 | |  |
| 0 | Stem clade of *Q. jiulongensis*, *Q. exilispinosa* + *Q. shini* + *Q. spinosa* | 100 | | 35.61 | | 23.30-44.16 | |  |
| 1 | Basal emergence of *Quasipaa* in East Asia | 100 | | 38.46 | | 32.20-44.36 | |  |
| 0 | Crown clade of *Q. exilispinosa* + *Q. spinosa* | 100 | | 28.13 | | 13.05-40.71 | |  |
| 0 | Crown clade of *Q. boulengeri* + *Q. yei* | 100 | | 37.06 | | N/a | |  |

**S16 Table. Multivariate (MANOVA) and univariate (ANOVA/Welch’s ANOVA) tests of acoustic traits in two cryptic and sympatric *Fejervarya* clades from the Yangtze Basin.** Comparisons are between *F. kawamurai* (Clade II, Jiangsu Plain; *N* = 6) and *F. multistriata* (Clade I, Shanghai; *N* = 11). Five call properties (call duration, call interval, call rate, pulse rate, and dominant frequency) were analysed across 17 individuals. Asterisks denote significance levels: p < 0.05 (), p < 0.001 (**). The data presented in this table correspond to the bar plots in Fig. 4.

| **Trait/model** | **Test** | **df** | **F** | **Wilks’ λ** | **p-value** | **Effect** |
| --- | --- | --- | --- | --- | --- | --- |
| Overall profile | MANOVA (Wilks) | 5,11 | 1.08 | 0.671 | 0.423 | no overall separation |
| Call duration | ANOVA | 1,15 | 4.19 | – | 0.059 | longer call in *F. multistriata* |
|  | ANOVA | 1,12.3 | 7.26 | – | 0.019 | robust difference |
| Call interval | ANOVA | 1,15 | 4.5 | – | 0.051 | shorter call in *F. multistriata* |
| Call rate | ANOVA | 1,15 | 0 | – | 0.999 | no difference |
| Pulse rate | ANOVA | 1,15 | 1.54 | – | 0.234 | no difference |
| Dominant frequency | ANOVA | 1,15 | 0.03 | – | 0.855 | no difference |

**S17 Table. ANCOVA results for call traits of *F. kawamurai* [Clade II] and *F. multistriata* [Clade I].** We tested species effects on call traits while controlling for temperature (temporal traits) and body size (dominant frequency). Significant interspecific differences were detected in call duration and call interval, with *F. multistriata* producing calls on average 0.16 s longer and 0.11 s at shorter intervals than *F. kawamurai*, independent of temperature.

| **Trait** | **Covariate** | **Species effect (β)** | **F (df)** | **p-value** | **Interpretation** |
| --- | --- | --- | --- | --- | --- |
| Call duration | Temperature | +0.16 s | F (1,12.3) = 7.26 | 0.019 | *F. multistriata* calls was 0.16 s longer, with minor temperature’s effect (+0.015 s/°C) |
| Call interval | Temperature | -0.11 s | F (1,15) = 13.12 | 0.0012 | *F. multistriata* intervals 0.11 s shorter than *F. kawamurai* with independent of temperature’s effect |
| Call rate | Temperature | N/a | N/a | 0.99 | No species effect |
| Pulse rate | Temperature | 4.4 / °C (F50.0temp) | F (1,15) = 3.54 | 0.025 (temp) | Driven by temperature (+4.40 pulses/°C) |
| Dominant frequency | Snout vent length | N/a | N/a | >0.55 | No effect of species or body size |

**S18 Table. Revised dataset of Dicroglossidae sequence (verified entries, *N* = 393) with georeferenced coordinates used for mismatch mapping in Fig. 5.** Geographic coordinates were assigned based on reported locality information, with missing values estimated using centroid-based coordinates of sampling localities. Model A1 assignments are compared with GenBank labels, match-mismatch status and associated regional classifications provided for each sample.

| **Sample ID** | **Model A1 assignment** | **Model A1 species label** | **Model A1 clade** | **GenBank original label** | **GenBank species label** | **Match status** | **Locality** | **Country** | **Latitutde** | **Longitude** |
| --- | --- | --- | --- | --- | --- | --- | --- | --- | --- | --- |
| HNFL01 | *Fejervarya kawamurai (Clade II)* | *Fejervarya kawamurai* | Clade II | *Fejervarya limnocharis* | *Fejervarya limnocharis* | mismatch | Fuluo, Hainan Island | China | 18.5988 | 108.7235 |
| HNFL02 | *Fejervarya kawamurai (Clade II)* | *Fejervarya kawamurai* | Clade II | *Fejervarya limnocharis* | *Fejervarya limnocharis* | mismatch | Fuluo, Hainan Island | China | 18.5988 | 108.7235 |
| HNFL03 | *Fejervarya kawamurai (Clade II)* | *Fejervarya kawamurai* | Clade II | *Fejervarya limnocharis* | *Fejervarya limnocharis* | mismatch | Fuluo, Hainan Island | China | 18.5988 | 108.7235 |
| HNLY01 | *Fejervarya kawamurai (Clade II)* | *Fejervarya kawamurai* | Clade II | *Fejervarya limnocharis* | *Fejervarya limnocharis* | mismatch | Luoyang, Henan | China | 34.6196 | 112.4541 |
| HNXY05 | *Fejervarya kawamurai (Clade II)* | *Fejervarya kawamurai* | Clade II | *Fejervarya limnocharis* | *Fejervarya limnocharis* | mismatch | Xinyang, Henan | China | 32.1649 | 114.0911 |
| SHLC15 | *Fejervarya kawamurai (Clade II)* | *Fejervarya kawamurai* | Clade II | *Fejervarya limnocharis* | *Fejervarya limnocharis* | mismatch | Luchao, Shanghai | China | 30.8818 | 121.8359 |
| ZJDQ05 | *Fejervarya kawamurai (Clade II)* | *Fejervarya kawamurai* | Clade II | *Fejervarya limnocharis* | *Fejervarya limnocharis* | mismatch | Hangzhou, Zhejiang | China | 30.46 | 120.45 |
| ZJDQ09 | *Fejervarya kawamurai (Clade II)* | *Fejervarya kawamurai* | Clade II | *Fejervarya limnocharis* | *Fejervarya limnocharis* | mismatch | Hangzhou, Zhejiang | China | 30.46 | 120.45 |
| ZJJX04 | *Fejervarya kawamurai (Clade II)* | *Fejervarya kawamurai* | Clade II | *Fejervarya limnocharis* | *Fejervarya limnocharis* | mismatch | Hangzhou, Zhejiang | China | 30.46 | 120.45 |
| ZJJX10 | *Fejervarya kawamurai (Clade II)* | *Fejervarya kawamurai* | Clade II | *Fejervarya limnocharis* | *Fejervarya limnocharis* | mismatch | Hangzhou, Zhejiang | China | 30.46 | 120.45 |
| ZJLX05 | *Fejervarya kawamurai (Clade II)* | *Fejervarya kawamurai* | Clade II | *Fejervarya limnocharis* | *Fejervarya limnocharis* | mismatch | Jinhua, Zhejiang | China | 29.3642 | 119.8111 |
| ZJLX06 | *Fejervarya kawamurai (Clade II)* | *Fejervarya kawamurai* | Clade II | *Fejervarya limnocharis* | *Fejervarya limnocharis* | mismatch | Jinhua, Zhejiang | China | 29.3642 | 119.8111 |
| ZJLX08 | *Fejervarya kawamurai (Clade II)* | *Fejervarya kawamurai* | Clade II | *Fejervarya limnocharis* | *Fejervarya limnocharis* | mismatch | Jinhua, Zhejiang | China | 29.3642 | 119.8111 |
| ZJLX09 | *Fejervarya kawamurai (Clade II)* | *Fejervarya kawamurai* | Clade II | *Fejervarya limnocharis* | *Fejervarya limnocharis* | mismatch | Jinhua, Zhejiang | China | 29.3642 | 119.8111 |
| ZJLX10 | *Fejervarya kawamurai (Clade II)* | *Fejervarya kawamurai* | Clade II | *Fejervarya limnocharis* | *Fejervarya limnocharis* | mismatch | Jinhua, Zhejiang | China | 29.3642 | 119.8111 |
| ZJLX12 | *Fejervarya kawamurai (Clade II)* | *Fejervarya kawamurai* | Clade II | *Fejervarya limnocharis* | *Fejervarya limnocharis* | mismatch | Jinhua, Zhejiang | China | 29.3642 | 119.8111 |
| ZJLX14 | *Fejervarya kawamurai (Clade II)* | *Fejervarya kawamurai* | Clade II | *Fejervarya limnocharis* | *Fejervarya limnocharis* | mismatch | Jinhua, Zhejiang | China | 29.3642 | 119.8111 |
| ZJLX15 | *Fejervarya kawamurai (Clade II)* | *Fejervarya kawamurai* | Clade II | *Fejervarya limnocharis* | *Fejervarya limnocharis* | mismatch | Jinhua, Zhejiang | China | 29.3642 | 119.8111 |
| ZJNB04 | *Fejervarya kawamurai (Clade II)* | *Fejervarya kawamurai* | Clade II | *Fejervarya limnocharis* | *Fejervarya limnocharis* | mismatch | Beilun, Zhejiang | China | 29.8999 | 121.8719 |
| ZJPH02 | *Fejervarya kawamurai (Clade II)* | *Fejervarya kawamurai* | Clade II | *Fejervarya limnocharis* | *Fejervarya limnocharis* | mismatch | Pinghu, Zhejiang | China | 30.6396 | 121.0488 |
| ZJPH03 | *Fejervarya kawamurai (Clade II)* | *Fejervarya kawamurai* | Clade II | *Fejervarya limnocharis* | *Fejervarya limnocharis* | mismatch | Pinghu, Zhejiang | China | 30.6396 | 121.0488 |
| ZJPH04 | *Fejervarya kawamurai (Clade II)* | *Fejervarya kawamurai* | Clade II | *Fejervarya limnocharis* | *Fejervarya limnocharis* | mismatch | Pinghu, Zhejiang | China | 30.6396 | 121.0488 |
| ZJPH05 | *Fejervarya kawamurai (Clade II)* | *Fejervarya kawamurai* | Clade II | *Fejervarya limnocharis* | *Fejervarya limnocharis* | mismatch | Pinghu, Zhejiang | China | 30.6396 | 121.0488 |
| ZJPJ01 | *Fejervarya kawamurai (Clade II)* | *Fejervarya kawamurai* | Clade II | *Fejervarya limnocharis* | *Fejervarya limnocharis* | mismatch | Jinhua, Zhejiang | China | 29.4847 | 120.0118 |
| ZJPJ02 | *Fejervarya kawamurai (Clade II)* | *Fejervarya kawamurai* | Clade II | *Fejervarya limnocharis* | *Fejervarya limnocharis* | mismatch | Jinhua, Zhejiang | China | 29.4847 | 120.0118 |
| ZJPJ03 | *Fejervarya kawamurai (Clade II)* | *Fejervarya kawamurai* | Clade II | *Fejervarya limnocharis* | *Fejervarya limnocharis* | mismatch | Jinhua, Zhejiang | China | 29.4847 | 120.0118 |
| ZJPJ04 | *Fejervarya kawamurai (Clade II)* | *Fejervarya kawamurai* | Clade II | *Fejervarya limnocharis* | *Fejervarya limnocharis* | mismatch | Jinhua, Zhejiang | China | 29.4847 | 120.0118 |
| ZJPJ05 | *Fejervarya kawamurai (Clade II)* | *Fejervarya kawamurai* | Clade II | *Fejervarya limnocharis* | *Fejervarya limnocharis* | mismatch | Jinhua, Zhejiang | China | 29.4847 | 120.0118 |
| ZJPJ09 | *Fejervarya kawamurai (Clade II)* | *Fejervarya kawamurai* | Clade II | *Fejervarya limnocharis* | *Fejervarya limnocharis* | mismatch | Jinhua, Zhejiang | China | 29.4847 | 120.0118 |
| ZJPJ11 | *Fejervarya kawamurai (Clade II)* | *Fejervarya kawamurai* | Clade II | *Fejervarya limnocharis* | *Fejervarya limnocharis* | mismatch | Jinhua, Zhejiang | China | 29.4847 | 120.0118 |
| ZJPJ15 | *Fejervarya kawamurai (Clade II)* | *Fejervarya kawamurai* | Clade II | *Fejervarya limnocharis* | *Fejervarya limnocharis* | mismatch | Jinhua, Zhejiang | China | 29.4847 | 120.0118 |
| ZSHX06 | *Fejervarya kawamurai (Clade II)* | *Fejervarya kawamurai* | Clade II | *Fejervarya limnocharis* | *Fejervarya limnocharis* | mismatch | Huangxing, Zhoushan | China | 30.1989 | 122.6412 |
| ZSKZ08 | *Fejervarya kawamurai (Clade II)* | *Fejervarya kawamurai* | Clade II | *Fejervarya limnocharis* | *Fejervarya limnocharis* | mismatch | Kuzhu,Zhoushan | China | 29.9928 | 122.2436 |
| GZBY01 | *Fejervarya kawamurai (Clade I)* | *Fejervarya kawamurai* | Clade I | *Fejervarya limnocharis* | *Fejervarya limnocharis* | mismatch | Guangzhou | China | 23.2857 | 113.3058 |
| GZBY02 | *Fejervarya kawamurai (Clade I)* | *Fejervarya kawamurai* | Clade I | *Fejervarya limnocharis* | *Fejervarya limnocharis* | mismatch | Guangzhou | China | 23.2857 | 113.3058 |
| GZBY04 | *Fejervarya kawamurai (Clade I)* | *Fejervarya kawamurai* | Clade I | *Fejervarya limnocharis* | *Fejervarya limnocharis* | mismatch | Guangzhou | China | 23.2857 | 113.3058 |
| GZBY05 | *Fejervarya kawamurai (Clade I)* | *Fejervarya kawamurai* | Clade I | *Fejervarya limnocharis* | *Fejervarya limnocharis* | mismatch | Guangzhou | China | 23.2857 | 113.3058 |
| HNXY05 | *Fejervarya kawamurai (Clade I)* | *Fejervarya kawamurai* | Clade I | *Fejervarya limnocharis* | *Fejervarya limnocharis* | mismatch | Shanghai | China | 32.1469 | 114.091 |
| ZJPH12 | *Fejervarya kawamurai (Clade I)* | *Fejervarya kawamurai* | Clade I | *Fejervarya limnocharis* | *Fejervarya limnocharis* | mismatch | Shaoxing, Zhejiang | China | 30.6396 | 121.0488 |
| ZSBQ11 | *Fejervarya kawamurai (Clade I)* | *Fejervarya kawamurai* | Clade I | *Fejervarya limnocharis* | *Fejervarya limnocharis* | mismatch | Baiquan, Zhoushan | China | 30.0567 | 122.1578 |
| ZSDD03 | *Fejervarya kawamurai (Clade I)* | *Fejervarya kawamurai* | Clade I | *Fejervarya limnocharis* | *Fejervarya limnocharis* | mismatch | Daidong, Zhoushan | China | 30.2902 | 122.1947 |
| ZSDF01 | *Fejervarya kawamurai (Clade I)* | *Fejervarya kawamurai* | Clade I | *Fejervarya limnocharis* | *Fejervarya limnocharis* | mismatch | Dongfu, Zhoushan | China | 30.1442 | 122.7575 |
| ZSDF03 | *Fejervarya kawamurai (Clade I)* | *Fejervarya kawamurai* | Clade I | *Fejervarya limnocharis* | *Fejervarya limnocharis* | mismatch | Dongfu, Zhoushan | China | 30.1442 | 122.7575 |
| ZSDH02 | *Fejervarya kawamurai (Clade I)* | *Fejervarya kawamurai* | Clade I | *Fejervarya limnocharis* | *Fejervarya limnocharis* | mismatch | Donghai, Zhoushan | China | 30.7083 | 122.4992 |
| ZSDJ12 | *Fejervarya kawamurai (Clade I)* | *Fejervarya kawamurai* | Clade I | *Fejervarya limnocharis* | *Fejervarya limnocharis* | mismatch | Daijia, Zhoushan | China | 30.0198 | 122.0332 |
| ZSGJ01 | *Fejervarya kawamurai (Clade I)* | *Fejervarya kawamurai* | Clade I | *Fejervarya limnocharis* | *Fejervarya limnocharis* | mismatch | Shaoxing, Zhejiang | China | 29.8833 | 121.9823 |
| ZSGL04 | *Fejervarya kawamurai (Clade I)* | *Fejervarya kawamurai* | Clade I | *Fejervarya limnocharis* | *Fejervarya limnocharis* | mismatch | Ganlan,Zhoushan | China | 30.1121 | 122.1305 |
| ZSGQ06 | *Fejervarya kawamurai (Clade I)* | *Fejervarya kawamurai* | Clade I | *Fejervarya limnocharis* | *Fejervarya limnocharis* | mismatch | Gouqi, Zhoushan | China | 30.7162 | 122.7724 |
| ZSHL03 | *Fejervarya kawamurai (Clade I)* | *Fejervarya kawamurai* | Clade I | *Fejervarya limnocharis* | *Fejervarya limnocharis* | mismatch | Huanglong, Zhoushan | China | 30.728 | 122.8146 |
| ZSHN01 | *Fejervarya kawamurai (Clade I)* | *Fejervarya kawamurai* | Clade I | *Fejervarya limnocharis* | *Fejervarya limnocharis* | mismatch | Huaniao, Zhoushan | China | 30.8474 | 122.6917 |
| ZSHX06 | *Fejervarya kawamurai (Clade I)* | *Fejervarya kawamurai* | Clade I | *Fejervarya limnocharis* | *Fejervarya limnocharis* | mismatch | Huangxing, Zhoushan | China | 30.1989 | 122.6412 |
| ZSJH01 | *Fejervarya kawamurai (Clade I)* | *Fejervarya kawamurai* | Clade I | *Fejervarya limnocharis* | *Fejervarya limnocharis* | mismatch | Jihu, Zhoushan | China | 30.7182 | 122.4624 |
| ZSKZ08 | *Fejervarya kawamurai (Clade I)* | *Fejervarya kawamurai* | Clade I | *Fejervarya limnocharis* | *Fejervarya limnocharis* | mismatch | Kuzhu, Zhoushan | China | 29.9928 | 122.2436 |
| ZSMZH01 | *Fejervarya kawamurai (Clade I)* | *Fejervarya kawamurai* | Clade I | *Fejervarya limnocharis* | *Fejervarya limnocharis* | mismatch | Miaozihu, Zhoushan | China | 30.1976 | 122.6792 |
| ZSMZH02 | *Fejervarya kawamurai (Clade I)* | *Fejervarya kawamurai* | Clade I | *Fejervarya limnocharis* | *Fejervarya limnocharis* | mismatch | Miaozihu, Zhoushan | China | 30.1976 | 122.6792 |
| ZSMZH03 | *Fejervarya kawamurai (Clade I)* | *Fejervarya kawamurai* | Clade I | *Fejervarya limnocharis* | *Fejervarya limnocharis* | mismatch | Miaozihu, Zhoushan | China | 30.1976 | 122.6792 |
| ZSNP03 | *Fejervarya kawamurai (Clade I)* | *Fejervarya kawamurai* | Clade I | *Fejervarya limnocharis* | *Fejervarya limnocharis* | mismatch | Nanpu, Zhoushan | China | 30.2667 | 122.1385 |
| ZSQB04 | *Fejervarya kawamurai (Clade I)* | *Fejervarya kawamurai* | Clade I | *Fejervarya limnocharis* | *Fejervarya limnocharis* | mismatch | Qingbang, Zhoushan | China | 30.1995 | 122.7069 |
| ZSQS02 | *Fejervarya kawamurai (Clade I)* | *Fejervarya kawamurai* | Clade I | *Fejervarya limnocharis* | *Fejervarya limnocharis* | mismatch | Qushan, Zhoushan, | China | 30.4371 | 122.3516 |
| ZSSN05 | *Fejervarya kawamurai (Clade I)* | *Fejervarya kawamurai* | Clade I | *Fejervarya limnocharis* | *Fejervarya limnocharis* | mismatch | Shengshan, Zhoushan | China | 30.7239 | 122.811 |
| ZSSN09 | *Fejervarya kawamurai (Clade I)* | *Fejervarya kawamurai* | Clade I | *Fejervarya limnocharis* | *Fejervarya limnocharis* | mismatch | Sannong, Zhoushan | China | 30.4454 | 122.2938 |
| ZSSS05 | *Fejervarya kawamurai (Clade I)* | *Fejervarya kawamurai* | Clade I | *Fejervarya limnocharis* | *Fejervarya limnocharis* | mismatch | Sannong, Zhoushan | China | 30.4454 | 122.2938 |
| ZSTT06 | *Fejervarya kawamurai (Clade I)* | *Fejervarya kawamurai* | Clade I | *Fejervarya limnocharis* | *Fejervarya limnocharis* | mismatch | Tiantu,Zhoushan | China | 30.4386 | 122.41 |
| ZSXYS01 | *Fejervarya kawamurai (Clade I)* | *Fejervarya kawamurai* | Clade I | *Fejervarya limnocharis* | *Fejervarya limnocharis* | mismatch | Xiaoyangshan,Zhoushan | China | 30.6411 | 122.0611 |
| ZSYD09 | *Fejervarya kawamurai (Clade I)* | *Fejervarya kawamurai* | Clade I | *Fejervarya limnocharis* | *Fejervarya limnocharis* | mismatch | Yandun,Zhoushan | China | 30.136 | 122.0198 |
| ZSZM14 | *Fejervarya kawamurai (Clade I)* | *Fejervarya kawamurai* | Clade I | *Fejervarya limnocharis* | *Fejervarya limnocharis* | mismatch | Zhanmao,Zhoushan | China | 30.0476 | 122.2564 |
| Full mtDNA | *Fejervarya kawamurai (Clade I)* | *Fejervarya kawamurai* | Clade I | *Fejervarya kawamurai* | *Fejervarya kawamurai* | match | Zunyi city, Guizhou | China | 27.7083 | 106.92 |
| ZJLX05 | *Fejervarya kawamurai (Clade II)* | *Fejervarya kawamurai* | Clade II | *Fejervarya limnocharis* | *Fejervarya limnocharis* | mismatch | Jinhua, Zhejiang | China | 29.3642 | 119.8111 |
| ZJLX06 | *Fejervarya kawamurai (Clade II)* | *Fejervarya kawamurai* | Clade II | *Fejervarya limnocharis* | *Fejervarya limnocharis* | mismatch | Zhejiang, China | China | 29.3642 | 119.8111 |
| ZJLX08 | *Fejervarya kawamurai (Clade II)* | *Fejervarya kawamurai* | Clade II | *Fejervarya limnocharis* | *Fejervarya limnocharis* | mismatch | Zhejiang, China | China | 29.3642 | 119.8111 |
| ZJLX09 | *Fejervarya kawamurai (Clade II)* | *Fejervarya kawamurai* | Clade II | *Fejervarya limnocharis* | *Fejervarya limnocharis* | mismatch | Zhejiang, China | China | 29.3642 | 119.8111 |
| ZJLX12 | *Fejervarya kawamurai (Clade II)* | *Fejervarya kawamurai* | Clade II | *Fejervarya limnocharis* | *Fejervarya limnocharis* | mismatch | Zhejiang, China | China | 29.3642 | 119.8111 |
| ZJLX14 | *Fejervarya kawamurai (Clade II)* | *Fejervarya kawamurai* | Clade II | *Fejervarya limnocharis* | *Fejervarya limnocharis* | mismatch | Zhejiang, China | China | 29.3642 | 119.8111 |
| ZJLX15 | *Fejervarya kawamurai (Clade II)* | *Fejervarya kawamurai* | Clade II | *Fejervarya limnocharis* | *Fejervarya limnocharis* | mismatch | Zhejiang, China | China | 29.3642 | 119.8111 |
| ZJPH02 | *Fejervarya kawamurai (Clade II)* | *Fejervarya kawamurai* | Clade II | *Fejervarya limnocharis* | *Fejervarya limnocharis* | mismatch | Zhejiang, China | China | 30.6396 | 121.0488 |
| ZJPH03 | *Fejervarya kawamurai (Clade II)* | *Fejervarya kawamurai* | Clade II | *Fejervarya limnocharis* | *Fejervarya limnocharis* | mismatch | Zhejiang, China | China | 30.6396 | 121.0488 |
| ZJPH04 | *Fejervarya kawamurai (Clade II)* | *Fejervarya kawamurai* | Clade II | *Fejervarya limnocharis* | *Fejervarya limnocharis* | mismatch | Zhejiang, China | China | 30.6396 | 121.0488 |
| ZJPH05 | *Fejervarya kawamurai (Clade II)* | *Fejervarya kawamurai* | Clade II | *Fejervarya limnocharis* | *Fejervarya limnocharis* | mismatch | Zhejiang, China | China | 30.6396 | 121.0488 |
| Jp1 | *Fejervarya sakishimensis* | *Fejervarya sakishimensis* | | *Fejervarya sakishimensis* | *Fejervarya sakishimensis* | match | Iriomote Island | Japan | 24.33 | 123.8 |
| CAS 235484 | *Fejervarya limnocharis (Clade II)* | *Fejervarya limnocharis* | Clade II | *Fejervarya limnocharis* | *Fejervarya limnocharis* | match | Krung Thep Mahanakhon (Bangkok) | Thailand | 13.7563 | 100.5018 |
| CAS 215116 | *Fejervarya limnocharis (Clade II)* | *Fejervarya limnocharis* | Clade II | *Fejervarya limnocharis* | *Fejervarya limnocharis* | match | Shanjiang village, just S of Liuku, Yunnan | China | 25.85 | 98.85 |
| CAS 228131 | *Fejervarya limnocharis (Clade II)* | *Fejervarya limnocharis* | Clade II | *Fejervarya limnocharis* | *Fejervarya limnocharis* | match | Shanjiang village, just S of Liuku, Yunnan | China | 25.85 | 98.85 |
| CAS 228129 | *Fejervarya limnocharis (Clade II)* | *Fejervarya limnocharis* | Clade II | *Fejervarya limnocharis* | *Fejervarya limnocharis* | match | Liuku bridge, Yunnan | China | 25.8435 | 98.8565 |
| CAS 230367 | *Fejervarya limnocharis (Clade II)* | *Fejervarya limnocharis* | Clade II | *Fejervarya limnocharis* | *Fejervarya limnocharis* | match | Township, Pidaung Wildlife Sanctuary, near Ma-Li-Hka Village | Verified | 27.6 | 97.25 |
| CAS 234814 | *Fejervarya limnocharis (Clade II)* | *Fejervarya limnocharis* | Clade II | *Fejervarya limnocharis* | *Fejervarya limnocharis* | match | Chin: Kanpatlat Township, in Kanpatlat | Myanmar | 21.19 | 94.03 |
| CAS 234815 | *Fejervarya limnocharis (Clade II)* | *Fejervarya limnocharis* | Clade II | *Fejervarya limnocharis* | *Fejervarya limnocharis* | match | Chin: Kanpatlat Township, in Kanpatlat | Myanmar | 21.19 | 94.03 |
| CAS 235276 | *Fejervarya limnocharis (Clade II)* | *Fejervarya limnocharis* | Clade II | *Fejervarya limnocharis* | *Fejervarya limnocharis* | match | Kanpatlat Township, Let Mon Village | Myanmar | 21.19 | 94.03 |
| SMF 104127 | *Fejervarya limnocharis (Clade II)* | *Fejervarya limnocharis* | Clade II | *Fejervarya limnocharis* | *Fejervarya limnocharis* | match | Mae Hong Son, Pangmapha, Ban Soppong | Thailand | 19.57 | 98.28 |
| Thai-1 | *Fejervarya limnocharis (Clade II)* | *Fejervarya limnocharis* | Clade II | *Fejervarya limnocharis* | *Fejervarya limnocharis* | match | Ranong | Thailand | 9.96 | 98.63 |
| Thai-2 | *Fejervarya limnocharis (Clade II)* | *Fejervarya limnocharis* | Clade II | *Fejervarya limnocharis* | *Fejervarya limnocharis* | match | Tha Ton | Thailand | 20.03 | 99.18 |
| Thai-3 | *Fejervarya limnocharis (Clade II)* | *Fejervarya limnocharis* | Clade II | *Fejervarya limnocharis* | *Fejervarya limnocharis* | match | Mae Hong Son | Thailand | 19.3 | 97.97 |
| Thai-4 | *Fejervarya limnocharis (Clade II)* | *Fejervarya limnocharis* | Clade II | *Fejervarya limnocharis* | *Fejervarya limnocharis* | match | Nakhon Si Thammarat | Thailand | 8.43 | 99.96 |
| SMF 104128 | *Fejervarya limnocharis (Clade II)* | *Fejervarya limnocharis* | Clade II | *Fejervarya limnocharis* | *Fejervarya limnocharis* | match | Mae Hong Son, Pangmapha, Ban Soppong | Thailand | 19.57 | 98.28 |
| SMF 104131 | *Fejervarya limnocharis (Clade II)* | *Fejervarya limnocharis* | Clade II | *Fejervarya limnocharis* | *Fejervarya limnocharis* | match | Pangmapha, near Ban Nam Rin, Thailand | Thailand | 19.6 | 98.3 |
| SMF 105119 | *Fejervarya limnocharis (Clade II)* | *Fejervarya limnocharis* | Clade II | *Fejervarya limnocharis* | *Fejervarya limnocharis* | match | Ridge north of viewpoint between Pangmapha and Pai, Thailand | Thailand | 19.46 | 98.33 |
| SMF 105120 | *Fejervarya limnocharis (Clade II)* | *Fejervarya limnocharis* | Clade II | *Fejervarya limnocharis* | *Fejervarya limnocharis* | match | Ridge north of viewpoint between Pangmapha and Pai, Thailand | Thailand | 19.46 | 98.33 |
| SMF 105121 | *Fejervarya limnocharis (Clade II)* | *Fejervarya limnocharis* | Clade II | *Fejervarya limnocharis* | *Fejervarya limnocharis* | match | Pangmapha district, at NamLang River, Thailand | Thailand | 19.58 | 98.26 |
| TZ25 | *Fejervarya limnocharis (Clade II)* | *Fejervarya limnocharis* | Clade II | *Fejervarya limnocharis* | *Fejervarya limnocharis* | match | North Vietnam | Vietnam | 21.5 | 105.8 |
| TZ98105 | *Fejervarya limnocharis (Clade II)* | *Fejervarya limnocharis* | Clade II | *Fejervarya limnocharis* | *Fejervarya limnocharis* | match | North Vietnam | Vietnam | 21.5 | 105.8 |
| sample_100 | *Fejervarya limnocharis (Clade II)* | *Fejervarya limnocharis* | Clade II | *Fejervarya limnocharis* | *Fejervarya limnocharis* | match | Northern Karsh forest, Vietnam | Vietnam | 22.8 | 105 |
| 1999.572 | *Fejervarya limnocharis (Clade II)* | *Fejervarya limnocharis* | Clade II | *Fejervarya limnocharis* | *Fejervarya limnocharis* | match | Lao Cai, Sapa | Vietnam | 22.3167 | 103.7833 |
| 2004.021 | *Fejervarya limnocharis (Clade II)* | *Fejervarya limnocharis* | Clade II | *Fejervarya limnocharis* | *Fejervarya limnocharis* | match | Phongsali, Buon Tai, Laos | Laos | 21.3833 | 101.9667 |
| 2004.031 | *Fejervarya limnocharis (Clade II)* | *Fejervarya limnocharis* | Clade II | *Fejervarya limnocharis* | *Fejervarya limnocharis* | match | Phongsali, Nathen, Laos | Laos | 21.4 | 101.9667 |
| 2006.241 | *Fejervarya limnocharis (Clade II)* | *Fejervarya limnocharis* | Clade II | *Fejervarya limnocharis* | *Fejervarya limnocharis* | match | Louangphrabang, Ban Sop Khao | Laos | 19.88 | 102.16 |
| K3037 | *Fejervarya limnocharis (Clade II)* | *Fejervarya limnocharis* | Clade II | *Fejervarya limnocharis* | *Fejervarya limnocharis* | match | Chiang Mai, Doi Chiang Dao | Thailand | 19.4 | 98.93 |
| K3085 | *Fejervarya limnocharis (Clade II)* | *Fejervarya limnocharis* | Clade II | *Fejervarya limnocharis* | *Fejervarya limnocharis* | match | Louangphrabang, Ban Sop Khao | Laos | 19.88 | 102.16 |
| FM_HN_01 | *Fejervarya kawamurai (Clade II)* | *Fejervarya kawamurai* | Clade II | *Fejervarya multistriata* | *Fejervarya multistriata* | mismatch | Northern Chinese Mainland | China | 36.5 | 112.5 |
| FM_HN_02 | *Fejervarya kawamurai (Clade II)* | *Fejervarya kawamurai* | Clade II | *Fejervarya multistriata* | *Fejervarya multistriata* | mismatch | Northern Chinese Mainland | China | 36.5 | 112.5 |
| FM_HN_03 | *Fejervarya kawamurai (Clade II)* | *Fejervarya kawamurai* | Clade II | *Fejervarya multistriata* | *Fejervarya multistriata* | mismatch | Northern Chinese Mainland | China | 36.5 | 112.5 |
| FM_HN_04 | *Fejervarya kawamurai (Clade II)* | *Fejervarya kawamurai* | Clade II | *Fejervarya multistriata* | *Fejervarya multistriata* | mismatch | Northern Chinese Mainland | China | 36.5 | 112.5 |
| FM_HN_05 | *Fejervarya kawamurai (Clade II)* | *Fejervarya kawamurai* | Clade II | *Fejervarya multistriata* | *Fejervarya multistriata* | mismatch | Northern Chinese Mainland | China | 36.5 | 112.5 |
| FM_HN_06 | *Fejervarya kawamurai (Clade II)* | *Fejervarya kawamurai* | Clade II | *Fejervarya multistriata* | *Fejervarya multistriata* | mismatch | Northern Chinese Mainland | China | 36.5 | 112.5 |
| FM_HN_07 | *Fejervarya kawamurai (Clade II)* | *Fejervarya kawamurai* | Clade II | *Fejervarya multistriata* | *Fejervarya multistriata* | mismatch | Northern Chinese Mainland | China | 36.5 | 112.5 |
| FM_HN_08 | *Fejervarya kawamurai (Clade II)* | *Fejervarya kawamurai* | Clade II | *Fejervarya multistriata* | *Fejervarya multistriata* | mismatch | Northern Chinese Mainland | China | 36.5 | 112.5 |
| FM_HN_09 | *Fejervarya kawamurai (Clade II)* | *Fejervarya kawamurai* | Clade II | *Fejervarya multistriata* | *Fejervarya multistriata* | mismatch | Northern Chinese Mainland | China | 36.5 | 112.5 |
| FM_HN_10 | *Fejervarya kawamurai (Clade II)* | *Fejervarya kawamurai* | Clade II | *Fejervarya multistriata* | *Fejervarya multistriata* | mismatch | Northern Chinese Mainland | China | 36.5 | 112.5 |
| MZ44_MZ | *Fejervarya kawamurai (Clade II)* | *Fejervarya kawamurai* | Clade II | *Fejervarya multistriata* | *Fejervarya multistriata* | mismatch | Chongming, Shanghai | China | 31.63 | 121.45 |
| ZWc_1113_BZ | *Fejervarya kawamurai (Clade II)* | *Fejervarya kawamurai* | Clade II | *Fejervarya multistriata* | *Fejervarya multistriata* | mismatch | Chongming, Shanghai | China | 31.63 | 121.45 |
| ZWc_1121_BZ | *Fejervarya kawamurai (Clade II)* | *Fejervarya kawamurai* | Clade II | *Fejervarya multistriata* | *Fejervarya multistriata* | mismatch | Chongming, Shanghai | China | 31.63 | 121.45 |
| ZWc_13111_MZ | *Fejervarya kawamurai (Clade II)* | *Fejervarya kawamurai* | Clade II | *Fejervarya multistriata* | *Fejervarya multistriata* | mismatch | Chongming, Shanghai | China | 31.63 | 121.45 |
| ZWc_1317_MZ | *Fejervarya kawamurai (Clade II)* | *Fejervarya kawamurai* | Clade II | *Fejervarya multistriata* | *Fejervarya multistriata* | mismatch | Chongming, Shanghai | China | 31.63 | 121.45 |
| ZWc_1343_MZ | *Fejervarya kawamurai (Clade II)* | *Fejervarya kawamurai* | Clade II | *Fejervarya multistriata* | *Fejervarya multistriata* | mismatch | Chongming, Shanghai | China | 31.63 | 121.45 |
| ZWc_1345_MZ | *Fejervarya kawamurai (Clade II)* | *Fejervarya kawamurai* | Clade II | *Fejervarya multistriata* | *Fejervarya multistriata* | mismatch | Chongming, Shanghai | China | 31.63 | 121.45 |
| ZWc_1346_MZ | *Fejervarya kawamurai (Clade II)* | *Fejervarya kawamurai* | Clade II | *Fejervarya multistriata* | *Fejervarya multistriata* | mismatch | Chongming, Shanghai | China | 31.63 | 121.45 |
| ZWc_1516a_MT | *Fejervarya kawamurai (Clade II)* | *Fejervarya kawamurai* | Clade II | *Fejervarya multistriata* | *Fejervarya multistriata* | mismatch | Chongming, Shanghai | China | 31.63 | 121.45 |
| ZWc_1527_MZH | *Fejervarya kawamurai (Clade II)* | *Fejervarya kawamurai* | Clade II | *Fejervarya multistriata* | *Fejervarya multistriata* | mismatch | Chongming, Shanghai | China | 31.63 | 121.45 |
| ZWc_1611b_BBY | *Fejervarya kawamurai (Clade II)* | *Fejervarya kawamurai* | Clade II | *Fejervarya multistriata* | *Fejervarya multistriata* | mismatch | Chongming, Shanghai | China | 31.63 | 121.45 |
| ZWc_1621_BBY | *Fejervarya kawamurai (Clade II)* | *Fejervarya kawamurai* | Clade II | *Fejervarya multistriata* | *Fejervarya multistriata* | mismatch | Chongming, Shanghai | China | 31.63 | 121.45 |
| ZWc_1622b_BBY | *Fejervarya kawamurai (Clade II)* | *Fejervarya kawamurai* | Clade II | *Fejervarya multistriata* | *Fejervarya multistriata* | mismatch | Chongming, Shanghai | China | 31.63 | 121.45 |
| ZWc_1643b_BBY | *Fejervarya kawamurai (Clade II)* | *Fejervarya kawamurai* | Clade II | *Fejervarya multistriata* | *Fejervarya multistriata* | mismatch | Chongming, Shanghai | China | 31.63 | 121.45 |
| ZWc_1646b_BBY | *Fejervarya kawamurai (Clade II)* | *Fejervarya kawamurai* | Clade II | *Fejervarya multistriata* | *Fejervarya multistriata* | mismatch | Chongming, Shanghai | China | 31.63 | 121.45 |
| ZWc_1819b_DP | *Fejervarya kawamurai (Clade II)* | *Fejervarya kawamurai* | Clade II | *Fejervarya multistriata* | *Fejervarya multistriata* | mismatch | Chongming, Shanghai | China | 31.63 | 121.45 |
| ZWc_1826_DP | *Fejervarya kawamurai (Clade II)* | *Fejervarya kawamurai* | Clade II | *Fejervarya multistriata* | *Fejervarya multistriata* | mismatch | Chongming, Shanghai | China | 31.63 | 121.45 |
| ZWc_BBY29_BBY | *Fejervarya kawamurai (Clade II)* | *Fejervarya kawamurai* | Clade II | *Fejervarya multistriata* | *Fejervarya multistriata* | mismatch | Chongming, Shanghai | China | 31.63 | 121.45 |
| ZWc_BHQ07_DT | *Fejervarya kawamurai (Clade II)* | *Fejervarya kawamurai* | Clade II | *Fejervarya multistriata* | *Fejervarya multistriata* | mismatch | Chongming, Shanghai | China | 31.63 | 121.45 |
| ZWc_BHQ12_DT | *Fejervarya kawamurai (Clade II)* | *Fejervarya kawamurai* | Clade II | *Fejervarya multistriata* | *Fejervarya multistriata* | mismatch | Chongming, Shanghai | China | 31.63 | 121.45 |
| ZWc_BHQ14_DT | *Fejervarya kawamurai (Clade II)* | *Fejervarya kawamurai* | Clade II | *Fejervarya multistriata* | *Fejervarya multistriata* | mismatch | Chongming, Shanghai | China | 31.63 | 121.45 |
| ZWc_BZ09_BZ | *Fejervarya kawamurai (Clade II)* | *Fejervarya kawamurai* | Clade II | *Fejervarya multistriata* | *Fejervarya multistriata* | mismatch | Chongming, Shanghai | China | 31.63 | 121.45 |
| ZWc_BZ10_BZ | *Fejervarya kawamurai (Clade II)* | *Fejervarya kawamurai* | Clade II | *Fejervarya multistriata* | *Fejervarya multistriata* | mismatch | Chongming, Shanghai | China | 31.63 | 121.45 |
| ZWc_BZ13_BZ | *Fejervarya kawamurai (Clade II)* | *Fejervarya kawamurai* | Clade II | *Fejervarya multistriata* | *Fejervarya multistriata* | mismatch | Chongming, Shanghai | China | 31.63 | 121.45 |
| ZWc_BZ23_BZ | *Fejervarya kawamurai (Clade II)* | *Fejervarya kawamurai* | Clade II | *Fejervarya multistriata* | *Fejervarya multistriata* | mismatch | Chongming, Shanghai | China | 31.63 | 121.45 |
| ZWc_DL01_DL | *Fejervarya kawamurai (Clade II)* | *Fejervarya kawamurai* | Clade II | *Fejervarya multistriata* | *Fejervarya multistriata* | mismatch | Chongming, Shanghai | China | 31.63 | 121.45 |
| ZWc_DL02_DL | *Fejervarya kawamurai (Clade II)* | *Fejervarya kawamurai* | Clade II | *Fejervarya multistriata* | *Fejervarya multistriata* | mismatch | Chongming, Shanghai | China | 31.63 | 121.45 |
| ZWc_DL03_DL | *Fejervarya kawamurai (Clade II)* | *Fejervarya kawamurai* | Clade II | *Fejervarya multistriata* | *Fejervarya multistriata* | mismatch | Chongming, Shanghai | China | 31.63 | 121.45 |
| ZWc_DL11_DL | *Fejervarya kawamurai (Clade II)* | *Fejervarya kawamurai* | Clade II | *Fejervarya multistriata* | *Fejervarya multistriata* | mismatch | Chongming, Shanghai | China | 31.63 | 121.45 |
| ZWc_DL17_DL | *Fejervarya kawamurai (Clade II)* | *Fejervarya kawamurai* | Clade II | *Fejervarya multistriata* | *Fejervarya multistriata* | mismatch | Chongming, Shanghai | China | 31.63 | 121.45 |
| ZWc_DL18_DL | *Fejervarya kawamurai (Clade II)* | *Fejervarya kawamurai* | Clade II | *Fejervarya multistriata* | *Fejervarya multistriata* | mismatch | Chongming, Shanghai | China | 31.63 | 121.45 |
| ZWc_DT12_DT | *Fejervarya kawamurai (Clade II)* | *Fejervarya kawamurai* | Clade II | *Fejervarya multistriata* | *Fejervarya multistriata* | mismatch | Chongming, Shanghai | China | 31.63 | 121.45 |
| ZWc_DT2_DT | *Fejervarya kawamurai (Clade II)* | *Fejervarya kawamurai* | Clade II | *Fejervarya multistriata* | *Fejervarya multistriata* | mismatch | Chongming, Shanghai | China | 31.63 | 121.45 |
| ZWc_DT5_DT | *Fejervarya kawamurai (Clade II)* | *Fejervarya kawamurai* | Clade II | *Fejervarya multistriata* | *Fejervarya multistriata* | mismatch | Chongming, Shanghai | China | 31.63 | 121.45 |
| ZWc_DT8_DT | *Fejervarya kawamurai (Clade II)* | *Fejervarya kawamurai* | Clade II | *Fejervarya multistriata* | *Fejervarya multistriata* | mismatch | Chongming, Shanghai | China | 31.63 | 121.45 |
| ZWc_HS14_HS | *Fejervarya kawamurai (Clade II)* | *Fejervarya kawamurai* | Clade II | *Fejervarya multistriata* | *Fejervarya multistriata* | mismatch | Chongming, Shanghai | China | 31.63 | 121.45 |
| ZWc_HS16_HS | *Fejervarya kawamurai (Clade II)* | *Fejervarya kawamurai* | Clade II | *Fejervarya multistriata* | *Fejervarya multistriata* | mismatch | Chongming, Shanghai | China | 31.63 | 121.45 |
| ZWc_HS17_HS | *Fejervarya kawamurai (Clade II)* | *Fejervarya kawamurai* | Clade II | *Fejervarya multistriata* | *Fejervarya multistriata* | mismatch | Chongming, Shanghai | China | 31.63 | 121.45 |
| ZWc_MT02_MT | *Fejervarya kawamurai (Clade II)* | *Fejervarya kawamurai* | Clade II | *Fejervarya multistriata* | *Fejervarya multistriata* | mismatch | Chongming, Shanghai | China | 31.63 | 121.45 |
| ZWc_MT09_MT | *Fejervarya kawamurai (Clade II)* | *Fejervarya kawamurai* | Clade II | *Fejervarya multistriata* | *Fejervarya multistriata* | mismatch | Chongming, Shanghai | China | 31.63 | 121.45 |
| ZWc_MT10_MT | *Fejervarya kawamurai (Clade II)* | *Fejervarya kawamurai* | Clade II | *Fejervarya multistriata* | *Fejervarya multistriata* | mismatch | Chongming, Shanghai | China | 31.63 | 121.45 |
| ZWc_MT13_MT | *Fejervarya kawamurai (Clade II)* | *Fejervarya kawamurai* | Clade II | *Fejervarya multistriata* | *Fejervarya multistriata* | mismatch | Chongming, Shanghai | China | 31.63 | 121.45 |
| ZWc_MT16_MT | *Fejervarya kawamurai (Clade II)* | *Fejervarya kawamurai* | Clade II | *Fejervarya multistriata* | *Fejervarya multistriata* | mismatch | Chongming, Shanghai | China | 31.63 | 121.45 |
| ZWc_MT18_MT | *Fejervarya kawamurai (Clade II)* | *Fejervarya kawamurai* | Clade II | *Fejervarya multistriata* | *Fejervarya multistriata* | mismatch | Chongming, Shanghai | China | 31.63 | 121.45 |
| ZWc_MZ01_MZ | *Fejervarya kawamurai (Clade II)* | *Fejervarya kawamurai* | Clade II | *Fejervarya multistriata* | *Fejervarya multistriata* | mismatch | Chongming, Shanghai | China | 31.63 | 121.45 |
| ZWc_MZ04_MZ | *Fejervarya kawamurai (Clade II)* | *Fejervarya kawamurai* | Clade II | *Fejervarya multistriata* | *Fejervarya multistriata* | mismatch | Chongming, Shanghai | China | 31.63 | 121.45 |
| ZWc_MZ06_MZ | *Fejervarya kawamurai (Clade II)* | *Fejervarya kawamurai* | Clade II | *Fejervarya multistriata* | *Fejervarya multistriata* | mismatch | Chongming, Shanghai | China | 31.63 | 121.45 |
| ZWc_MZ09_MZ | *Fejervarya kawamurai (Clade II)* | *Fejervarya kawamurai* | Clade II | *Fejervarya multistriata* | *Fejervarya multistriata* | mismatch | Chongming, Shanghai | China | 31.63 | 121.45 |
| ZWc_MZ10_MZ | *Fejervarya kawamurai (Clade II)* | *Fejervarya kawamurai* | Clade II | *Fejervarya multistriata* | *Fejervarya multistriata* | mismatch | Chongming, Shanghai | China | 31.63 | 121.45 |
| _ZWc_MZ11_MZ | *Fejervarya kawamurai (Clade II)* | *Fejervarya kawamurai* | Clade II | *Fejervarya multistriata* | *Fejervarya multistriata* | mismatch | Chongming, Shanghai | China | 31.63 | 121.45 |
| ZWc_MZ13_MZ | *Fejervarya kawamurai (Clade II)* | *Fejervarya kawamurai* | Clade II | *Fejervarya multistriata* | *Fejervarya multistriata* | mismatch | Chongming, Shanghai | China | 31.63 | 121.45 |
| ZWc_MZ14_MZ | *Fejervarya kawamurai (Clade II)* | *Fejervarya kawamurai* | Clade II | *Fejervarya multistriata* | *Fejervarya multistriata* | mismatch | Chongming, Shanghai | China | 31.63 | 121.45 |
| ZWc_MZ15_MZ | *Fejervarya kawamurai (Clade II)* | *Fejervarya kawamurai* | Clade II | *Fejervarya multistriata* | *Fejervarya multistriata* | mismatch | Chongming, Shanghai | China | 31.63 | 121.45 |
| ZWc_MZ16_MZ | *Fejervarya kawamurai (Clade II)* | *Fejervarya kawamurai* | Clade II | *Fejervarya multistriata* | *Fejervarya multistriata* | mismatch | Chongming, Shanghai | China | 31.63 | 121.45 |
| ZWc_MZ21_MZ | *Fejervarya kawamurai (Clade II)* | *Fejervarya kawamurai* | Clade II | *Fejervarya multistriata* | *Fejervarya multistriata* | mismatch | Chongming, Shanghai | China | 31.63 | 121.45 |
| ZWc_MZ22_MZ | *Fejervarya kawamurai (Clade II)* | *Fejervarya kawamurai* | Clade II | *Fejervarya multistriata* | *Fejervarya multistriata* | mismatch | Chongming, Shanghai | China | 31.63 | 121.45 |
| ZWc_MZ23_MZ | *Fejervarya kawamurai (Clade II)* | *Fejervarya kawamurai* | Clade II | *Fejervarya multistriata* | *Fejervarya multistriata* | mismatch | Chongming, Shanghai | China | 31.63 | 121.45 |
| ZWc_MZ26_MZ | *Fejervarya kawamurai (Clade II)* | *Fejervarya kawamurai* | Clade II | *Fejervarya multistriata* | *Fejervarya multistriata* | mismatch | Chongming, Shanghai | China | 31.63 | 121.45 |
| ZWc_MZ30_MZ | *Fejervarya kawamurai (Clade II)* | *Fejervarya kawamurai* | Clade II | *Fejervarya multistriata* | *Fejervarya multistriata* | mismatch | Chongming, Shanghai | China | 31.63 | 121.45 |
| ZWc_MZH15_MZH | *Fejervarya kawamurai (Clade II)* | *Fejervarya kawamurai* | Clade II | *Fejervarya multistriata* | *Fejervarya multistriata* | mismatch | Chongming, Shanghai | China | 31.63 | 121.45 |
| ZWc_QJX26_QXJ | *Fejervarya kawamurai (Clade II)* | *Fejervarya kawamurai* | Clade II | *Fejervarya multistriata* | *Fejervarya multistriata* | mismatch | Chongming, Shanghai | China | 31.63 | 121.45 |
| ZWc_QJX31_QXJ | *Fejervarya kawamurai (Clade II)* | *Fejervarya kawamurai* | Clade II | *Fejervarya multistriata* | *Fejervarya multistriata* | mismatch | Chongming, Shanghai | China | 31.63 | 121.45 |
| ZWc_QXJ37_QXJ | *Fejervarya kawamurai (Clade II)* | *Fejervarya kawamurai* | Clade II | *Fejervarya multistriata* | *Fejervarya multistriata* | mismatch | Chongming, Shanghai | China | 31.63 | 121.45 |
| ZWc_QXJ46_QXJ | *Fejervarya kawamurai (Clade II)* | *Fejervarya kawamurai* | Clade II | *Fejervarya multistriata* | *Fejervarya multistriata* | mismatch | Chongming, Shanghai | China | 31.63 | 121.45 |
| ZWd1102_BZ | *Fejervarya kawamurai (Clade II)* | *Fejervarya kawamurai* | Clade II | *Fejervarya multistriata* | *Fejervarya multistriata* | mismatch | Chongming, Shanghai | China | 31.63 | 121.45 |
| ZWd1103_BZ | *Fejervarya kawamurai (Clade II)* | *Fejervarya kawamurai* | Clade II | *Fejervarya multistriata* | *Fejervarya multistriata* | mismatch | Chongming, Shanghai | China | 31.63 | 121.45 |
| ZWd1117_BZ | *Fejervarya kawamurai (Clade II)* | *Fejervarya kawamurai* | Clade II | *Fejervarya multistriata* | *Fejervarya multistriata* | mismatch | Chongming, Shanghai | China | 31.63 | 121.45 |
| ZWd1135_BZ | *Fejervarya kawamurai (Clade II)* | *Fejervarya kawamurai* | Clade II | *Fejervarya multistriata* | *Fejervarya multistriata* | mismatch | Chongming, Shanghai | China | 31.63 | 121.45 |
| ZWd1211_BZ | *Fejervarya kawamurai (Clade II)* | *Fejervarya kawamurai* | Clade II | *Fejervarya multistriata* | *Fejervarya multistriata* | mismatch | Chongming, Shanghai | China | 31.63 | 121.45 |
| ZWd1215_DP | *Fejervarya kawamurai (Clade II)* | *Fejervarya kawamurai* | Clade II | *Fejervarya multistriata* | *Fejervarya multistriata* | mismatch | Chongming, Shanghai | China | 31.63 | 121.45 |
| ZWd1231_BZ | *Fejervarya kawamurai (Clade II)* | *Fejervarya kawamurai* | Clade II | *Fejervarya multistriata* | *Fejervarya multistriata* | mismatch | Chongming, Shanghai | China | 31.63 | 121.45 |
| ZWd1244_BZ | *Fejervarya kawamurai (Clade II)* | *Fejervarya kawamurai* | Clade II | *Fejervarya multistriata* | *Fejervarya multistriata* | mismatch | Chongming, Shanghai | China | 31.63 | 121.45 |
| ZWd1339_MZ | *Fejervarya kawamurai (Clade II)* | *Fejervarya kawamurai* | Clade II | *Fejervarya multistriata* | *Fejervarya multistriata* | mismatch | Chongming, Shanghai | China | 31.63 | 121.45 |
| ZWd1343_MZ | *Fejervarya kawamurai (Clade II)* | *Fejervarya kawamurai* | Clade II | *Fejervarya multistriata* | *Fejervarya multistriata* | mismatch | Chongming, Shanghai | China | 31.63 | 121.45 |
| ZWd1515_MT | *Fejervarya kawamurai (Clade II)* | *Fejervarya kawamurai* | Clade II | *Fejervarya multistriata* | *Fejervarya multistriata* | mismatch | Chongming, Shanghai | China | 31.63 | 121.45 |
| ZWd1516_MT | *Fejervarya kawamurai (Clade II)* | *Fejervarya kawamurai* | Clade II | *Fejervarya multistriata* | *Fejervarya multistriata* | mismatch | Chongming, Shanghai | China | 31.63 | 121.45 |
| ZWd1525_MT | *Fejervarya kawamurai (Clade II)* | *Fejervarya kawamurai* | Clade II | *Fejervarya multistriata* | *Fejervarya multistriata* | mismatch | Chongming, Shanghai | China | 31.63 | 121.45 |
| ZWd1548a_MZH | *Fejervarya kawamurai (Clade II)* | *Fejervarya kawamurai* | Clade II | *Fejervarya multistriata* | *Fejervarya multistriata* | mismatch | Chongming, Shanghai | China | 31.63 | 121.45 |
| ZWd1613_BBY | *Fejervarya kawamurai (Clade II)* | *Fejervarya kawamurai* | Clade II | *Fejervarya multistriata* | *Fejervarya multistriata* | mismatch | Chongming, Shanghai | China | 31.63 | 121.45 |
| ZWd1617b_BBY | *Fejervarya kawamurai (Clade II)* | *Fejervarya kawamurai* | Clade II | *Fejervarya multistriata* | *Fejervarya multistriata* | mismatch | Chongming, Shanghai | China | 31.63 | 121.45 |
| ZWd1619b_BBY | *Fejervarya kawamurai (Clade II)* | *Fejervarya kawamurai* | Clade II | *Fejervarya multistriata* | *Fejervarya multistriata* | mismatch | Chongming, Shanghai | China | 31.63 | 121.45 |
| ZWd1621b_BBY | *Fejervarya kawamurai (Clade II)* | *Fejervarya kawamurai* | Clade II | *Fejervarya multistriata* | *Fejervarya multistriata* | mismatch | Chongming, Shanghai | China | 31.63 | 121.45 |
| ZWd1634_BBY | *Fejervarya kawamurai (Clade II)* | *Fejervarya kawamurai* | Clade II | *Fejervarya multistriata* | *Fejervarya multistriata* | mismatch | Chongming, Shanghai | China | 31.63 | 121.45 |
| ZWd1641b_BBY | *Fejervarya kawamurai (Clade II)* | *Fejervarya kawamurai* | Clade II | *Fejervarya multistriata* | *Fejervarya multistriata* | mismatch | Chongming, Shanghai | China | 31.63 | 121.45 |
| ZWd1642b_BBY | *Fejervarya kawamurai (Clade II)* | *Fejervarya kawamurai* | Clade II | *Fejervarya multistriata* | *Fejervarya multistriata* | mismatch | Chongming, Shanghai | China | 31.63 | 121.45 |
| ZWd1647b_BBY | *Fejervarya kawamurai (Clade II)* | *Fejervarya kawamurai* | Clade II | *Fejervarya multistriata* | *Fejervarya multistriata* | mismatch | Chongming, Shanghai | China | 31.63 | 121.45 |
| ZWd1813b_DP | *Fejervarya kawamurai (Clade II)* | *Fejervarya kawamurai* | Clade II | *Fejervarya multistriata* | *Fejervarya multistriata* | mismatch | Chongming, Shanghai | China | 31.63 | 121.45 |
| ZWd1814_DP | *Fejervarya kawamurai (Clade II)* | *Fejervarya kawamurai* | Clade II | *Fejervarya multistriata* | *Fejervarya multistriata* | mismatch | Chongming, Shanghai | China | 31.63 | 121.45 |
| ZWd1814b_DP | *Fejervarya kawamurai (Clade II)* | *Fejervarya kawamurai* | Clade II | *Fejervarya multistriata* | *Fejervarya multistriata* | mismatch | Chongming, Shanghai | China | 31.63 | 121.45 |
| ZWd1818_DP | *Fejervarya kawamurai (Clade II)* | *Fejervarya kawamurai* | Clade II | *Fejervarya multistriata* | *Fejervarya multistriata* | mismatch | Chongming, Shanghai | China | 31.63 | 121.45 |
| ZWd1818b_DP | *Fejervarya kawamurai (Clade II)* | *Fejervarya kawamurai* | Clade II | *Fejervarya multistriata* | *Fejervarya multistriata* | mismatch | Chongming, Shanghai | China | 31.63 | 121.45 |
| ZWd1819sb_DP | *Fejervarya kawamurai (Clade II)* | *Fejervarya kawamurai* | Clade II | *Fejervarya multistriata* | *Fejervarya multistriata* | mismatch | Chongming, Shanghai | China | 31.63 | 121.45 |
| ZWd1842_DP | *Fejervarya kawamurai (Clade II)* | *Fejervarya kawamurai* | Clade II | *Fejervarya multistriata* | *Fejervarya multistriata* | mismatch | Chongming, Shanghai | China | 31.63 | 121.45 |
| ZWdBBY09_BBY | *Fejervarya kawamurai (Clade II)* | *Fejervarya kawamurai* | Clade II | *Fejervarya multistriata* | *Fejervarya multistriata* | mismatch | Chongming, Shanghai | China | 31.63 | 121.45 |
| ZWdBBY29_BBY | *Fejervarya kawamurai (Clade II)* | *Fejervarya kawamurai* | Clade II | *Fejervarya multistriata* | *Fejervarya multistriata* | mismatch | Chongming, Shanghai | China | 31.63 | 121.45 |
| ZWdBZ03_BZ | *Fejervarya kawamurai (Clade II)* | *Fejervarya kawamurai* | Clade II | *Fejervarya multistriata* | *Fejervarya multistriata* | mismatch | Chongming, Shanghai | China | 31.63 | 121.45 |
| ZWdBZ18_BZ | *Fejervarya kawamurai (Clade II)* | *Fejervarya kawamurai* | Clade II | *Fejervarya multistriata* | *Fejervarya multistriata* | mismatch | Chongming, Shanghai | China | 31.63 | 121.45 |
| ZWdBZ22_BZ | *Fejervarya kawamurai (Clade II)* | *Fejervarya kawamurai* | Clade II | *Fejervarya multistriata* | *Fejervarya multistriata* | mismatch | Chongming, Shanghai | China | 31.63 | 121.45 |
| ZWdCX03_CX | *Fejervarya kawamurai (Clade II)* | *Fejervarya kawamurai* | Clade II | *Fejervarya multistriata* | *Fejervarya multistriata* | mismatch | Chongming, Shanghai | China | 31.63 | 121.45 |
| ZWdCX16_CX | *Fejervarya kawamurai (Clade II)* | *Fejervarya kawamurai* | Clade II | *Fejervarya multistriata* | *Fejervarya multistriata* | mismatch | Chongming, Shanghai | China | 31.63 | 121.45 |
| ZWdCXD1033_CX | *Fejervarya kawamurai (Clade II)* | *Fejervarya kawamurai* | Clade II | *Fejervarya multistriata* | *Fejervarya multistriata* | mismatch | Chongming, Shanghai | China | 31.63 | 121.45 |
| ZWdDL01_DL | *Fejervarya kawamurai (Clade II)* | *Fejervarya kawamurai* | Clade II | *Fejervarya multistriata* | *Fejervarya multistriata* | mismatch | Chongming, Shanghai | China | 31.63 | 121.45 |
| ZWdDL04_DL | *Fejervarya kawamurai (Clade II)* | *Fejervarya kawamurai* | Clade II | *Fejervarya multistriata* | *Fejervarya multistriata* | mismatch | Chongming, Shanghai | China | 31.63 | 121.45 |
| ZWdDL06_DL | *Fejervarya kawamurai (Clade II)* | *Fejervarya kawamurai* | Clade II | *Fejervarya multistriata* | *Fejervarya multistriata* | mismatch | Chongming, Shanghai | China | 31.63 | 121.45 |
| ZWdDL07_DL | *Fejervarya kawamurai (Clade II)* | *Fejervarya kawamurai* | Clade II | *Fejervarya multistriata* | *Fejervarya multistriata* | mismatch | Chongming, Shanghai | China | 31.63 | 121.45 |
| ZWdDL09_DL | *Fejervarya kawamurai (Clade II)* | *Fejervarya kawamurai* | Clade II | *Fejervarya multistriata* | *Fejervarya multistriata* | mismatch | Chongming, Shanghai | China | 31.63 | 121.45 |
| ZWdDL14_DL | *Fejervarya kawamurai (Clade II)* | *Fejervarya kawamurai* | Clade II | *Fejervarya multistriata* | *Fejervarya multistriata* | mismatch | Chongming, Shanghai | China | 31.63 | 121.45 |
| ZWdDL15_DL | *Fejervarya kawamurai (Clade II)* | *Fejervarya kawamurai* | Clade II | *Fejervarya multistriata* | *Fejervarya multistriata* | mismatch | Chongming, Shanghai | China | 31.63 | 121.45 |
| ZWdDL17_DL | *Fejervarya kawamurai (Clade II)* | *Fejervarya kawamurai* | Clade II | *Fejervarya multistriata* | *Fejervarya multistriata* | mismatch | Chongming, Shanghai | China | 31.63 | 121.45 |
| ZWdDL18_DL | *Fejervarya kawamurai (Clade II)* | *Fejervarya kawamurai* | Clade II | *Fejervarya multistriata* | *Fejervarya multistriata* | mismatch | Chongming, Shanghai | China | 31.63 | 121.45 |
| ZWdDP12_DP | *Fejervarya kawamurai (Clade II)* | *Fejervarya kawamurai* | Clade II | *Fejervarya multistriata* | *Fejervarya multistriata* | mismatch | Chongming, Shanghai | China | 31.63 | 121.45 |
| ZWdDP18_DP | *Fejervarya kawamurai (Clade II)* | *Fejervarya kawamurai* | Clade II | *Fejervarya multistriata* | *Fejervarya multistriata* | mismatch | Chongming, Shanghai | China | 31.63 | 121.45 |
| ZWdDT2_DT | *Fejervarya kawamurai (Clade II)* | *Fejervarya kawamurai* | Clade II | *Fejervarya multistriata* | *Fejervarya multistriata* | mismatch | Chongming, Shanghai | China | 31.63 | 121.45 |
| ZWdHS001_HS | *Fejervarya kawamurai (Clade II)* | *Fejervarya kawamurai* | Clade II | *Fejervarya multistriata* | *Fejervarya multistriata* | mismatch | Chongming, Shanghai | China | 31.63 | 121.45 |
| ZWdHS037_HS | *Fejervarya kawamurai (Clade II)* | *Fejervarya kawamurai* | Clade II | *Fejervarya multistriata* | *Fejervarya multistriata* | mismatch | Chongming, Shanghai | China | 31.63 | 121.45 |
| ZWdHS12_HS | *Fejervarya kawamurai (Clade II)* | *Fejervarya kawamurai* | Clade II | *Fejervarya multistriata* | *Fejervarya multistriata* | mismatch | Chongming, Shanghai | China | 31.63 | 121.45 |
| ZWdHS17_HS | *Fejervarya kawamurai (Clade II)* | *Fejervarya kawamurai* | Clade II | *Fejervarya multistriata* | *Fejervarya multistriata* | mismatch | Chongming, Shanghai | China | 31.63 | 121.45 |
| ZWdMT02_MT | *Fejervarya kawamurai (Clade II)* | *Fejervarya kawamurai* | Clade II | *Fejervarya multistriata* | *Fejervarya multistriata* | mismatch | Chongming, Shanghai | China | 31.63 | 121.45 |
| ZWdMT03_MT | *Fejervarya kawamurai (Clade II)* | *Fejervarya kawamurai* | Clade II | *Fejervarya multistriata* | *Fejervarya multistriata* | mismatch | Chongming, Shanghai | China | 31.63 | 121.45 |
| ZWdMT08_MT | *Fejervarya kawamurai (Clade II)* | *Fejervarya kawamurai* | Clade II | *Fejervarya multistriata* | *Fejervarya multistriata* | mismatch | Chongming, Shanghai | China | 31.63 | 121.45 |
| ZWdMT09_MT | *Fejervarya kawamurai (Clade II)* | *Fejervarya kawamurai* | Clade II | *Fejervarya multistriata* | *Fejervarya multistriata* | mismatch | Chongming, Shanghai | China | 31.63 | 121.45 |
| ZWdMT13_MT | *Fejervarya kawamurai (Clade II)* | *Fejervarya kawamurai* | Clade II | *Fejervarya multistriata* | *Fejervarya multistriata* | mismatch | Chongming, Shanghai | China | 31.63 | 121.45 |
| ZWdMT18_MT | *Fejervarya kawamurai (Clade II)* | *Fejervarya kawamurai* | Clade II | *Fejervarya multistriata* | *Fejervarya multistriata* | mismatch | Chongming, Shanghai | China | 31.63 | 121.45 |
| ZWdMZ06_MZ | *Fejervarya kawamurai (Clade II)* | *Fejervarya kawamurai* | Clade II | *Fejervarya multistriata* | *Fejervarya multistriata* | mismatch | Chongming, Shanghai | China | 31.63 | 121.45 |
| ZWdMZ11_MZ | *Fejervarya kawamurai (Clade II)* | *Fejervarya kawamurai* | Clade II | *Fejervarya multistriata* | *Fejervarya multistriata* | mismatch | Chongming, Shanghai | China | 31.63 | 121.45 |
| ZWdMZ13_MZ | *Fejervarya kawamurai (Clade II)* | *Fejervarya kawamurai* | Clade II | *Fejervarya multistriata* | *Fejervarya multistriata* | mismatch | Chongming, Shanghai | China | 31.63 | 121.45 |
| ZWdMZ14_MZ | *Fejervarya kawamurai (Clade II)* | *Fejervarya kawamurai* | Clade II | *Fejervarya multistriata* | *Fejervarya multistriata* | mismatch | Chongming, Shanghai | China | 31.63 | 121.45 |
| ZWdMZ26_MZ | *Fejervarya kawamurai (Clade II)* | *Fejervarya kawamurai* | Clade II | *Fejervarya multistriata* | *Fejervarya multistriata* | mismatch | Chongming, Shanghai | China | 31.63 | 121.45 |
| ZWdMZ30_MZ | *Fejervarya kawamurai (Clade II)* | *Fejervarya kawamurai* | Clade II | *Fejervarya multistriata* | *Fejervarya multistriata* | mismatch | Chongming, Shanghai | China | 31.63 | 121.45 |
| ZWdMZ32_MZ | *Fejervarya kawamurai (Clade II)* | *Fejervarya kawamurai* | Clade II | *Fejervarya multistriata* | *Fejervarya multistriata* | mismatch | Chongming, Shanghai | China | 31.63 | 121.45 |
| ZWdMZ35_MZ | *Fejervarya kawamurai (Clade II)* | *Fejervarya kawamurai* | Clade II | *Fejervarya multistriata* | *Fejervarya multistriata* | mismatch | Chongming, Shanghai | China | 31.63 | 121.45 |
| ZWdMZ46_MZ | *Fejervarya kawamurai (Clade II)* | *Fejervarya kawamurai* | Clade II | *Fejervarya multistriata* | *Fejervarya multistriata* | mismatch | Chongming, Shanghai | China | 31.63 | 121.45 |
| ZWdMZ47_MZ | *Fejervarya kawamurai (Clade II)* | *Fejervarya kawamurai* | Clade II | *Fejervarya multistriata* | *Fejervarya multistriata* | mismatch | Chongming, Shanghai | China | 31.63 | 121.45 |
| ZWdMZH04_MZH | *Fejervarya kawamurai (Clade II)* | *Fejervarya kawamurai* | Clade II | *Fejervarya multistriata* | *Fejervarya multistriata* | mismatch | Chongming, Shanghai | China | 31.63 | 121.45 |
| ZWdMZH09_MZH | *Fejervarya kawamurai (Clade II)* | *Fejervarya kawamurai* | Clade II | *Fejervarya multistriata* | *Fejervarya multistriata* | mismatch | Chongming, Shanghai | China | 31.63 | 121.45 |
| ZWdMZH16_MZH | *Fejervarya kawamurai (Clade II)* | *Fejervarya kawamurai* | Clade II | *Fejervarya multistriata* | *Fejervarya multistriata* | mismatch | Chongming, Shanghai | China | 31.63 | 121.45 |
| ZWdQXJ43_QXJ | *Fejervarya kawamurai (Clade II)* | *Fejervarya kawamurai* | Clade II | *Fejervarya multistriata* | *Fejervarya multistriata* | mismatch | Chongming, Shanghai | China | 31.63 | 121.45 |
| ZWd1819_DP | *Fejervarya kawamurai (Clade II)* | *Fejervarya kawamurai* | Clade II | *Fejervarya multistriata* | *Fejervarya multistriata* | mismatch | Chongming, Shanghai | China | 31.63 | 121.45 |
| ZWdBHQ06_DT | *Fejervarya kawamurai (Clade II)* | *Fejervarya kawamurai* | Clade II | *Fejervarya multistriata* | *Fejervarya multistriata* | mismatch | Chongming, Shanghai | China | 31.63 | 121.45 |
| ZWdMZ36_MZ | *Fejervarya kawamurai (Clade II)* | *Fejervarya kawamurai* | Clade II | *Fejervarya multistriata* | *Fejervarya multistriata* | mismatch | Chongming, Shanghai | China | 31.63 | 121.45 |
| 15083 | *Fejervarya multistriata (Clade II)* | *Fejervarya multistriata* | Clade II | *Fejervarya limnocharis* | *Fejervarya limnocharis* | mismatch | Taiwan Province, Taichung City, Taichung | Taiwan | 31.63 | 121.45 |
| 17691 | *Fejervarya multistriata (Clade II)* | *Fejervarya multistriata* | Clade II | *Fejervarya limnocharis* | *Fejervarya limnocharis* | mismatch | Lienchiang County, Nangan Township, Fuwoling, Taiwan | Taiwan | 26.156 | 119.947 |
| 17744 | *Fejervarya multistriata (Clade II)* | *Fejervarya multistriata* | Clade II | *Fejervarya limnocharis* | *Fejervarya limnocharis* | mismatch | New Taipei City, Shimen District, Jutsao | Taiwan | 25.268 | 121.551 |
| 17620 | *Fejervarya multistriata (Clade I)* | *Fejervarya multistriata* | Clade I | *Fejervarya limnocharis* | *Fejervarya limnocharis* | mismatch | Guangdong, Shaoguan City, Juyuan County, Tianjingshan Forestry Station | China | 24.698 | 112.988 |
| 17674 | *Fejervarya multistriata (Clade I)* | *Fejervarya multistriata* | Clade I | *Fejervarya limnocharis* | *Fejervarya limnocharis* | mismatch | Fujian, Xiamen City, Xiamen University | China | 24.442 | 118.099 |
| 18178 | *Fejervarya multistriata (Clade II)* | *Fejervarya multistriata* | Clade II | *Fejervarya limnocharis* | *Fejervarya limnocharis* | mismatch | Pintung County, Jiadung Township, Hsiaputou | Taiwan | 22.4092 | 120.561 |
| 18238 | *Fejervarya multistriata (Clade II)* | *Fejervarya multistriata* | Clade II | *Fejervarya limnocharis* | *Fejervarya limnocharis* | mismatch | Yilan County, Toucheng Township, Toucheng | Taiwan | 24.893 | 121.856 |
| JL73-2-1 | *Fejervarya multistriata (Clade II)* | *Fejervarya multistriata* | Clade II | *Fejervarya limnocharis* | *Fejervarya limnocharis* | mismatch | Yilan County, Nan-ao | Taiwan | 24.4388 | 121.764 |
| JL73-2-2 | *Fejervarya multistriata (Clade II)* | *Fejervarya multistriata* | Clade II | *Fejervarya limnocharis* | *Fejervarya limnocharis* | mismatch | Yilan County, Nan-ao | Taiwan | 24.4388 | 121.764 |
| JL106 | *Fejervarya multistriata (Clade II)* | *Fejervarya multistriata* | Clade II | *Fejervarya limnocharis* | *Fejervarya limnocharis* | mismatch | Tainan City, Liouying | Taiwan | 23.261 | 120.378 |
| JL66-2 | *Fejervarya multistriata (Clade II)* | *Fejervarya multistriata* | Clade II | *Fejervarya limnocharis* | *Fejervarya limnocharis* | mismatch | Taiwan Province, Pingtung County, Sandimen | Taiwan | 22.7519 | 120.636 |
| JL-19-2 | *Fejervarya multistriata (Clade II)* | *Fejervarya multistriata* | Clade II | *Fejervarya limnocharis* | *Fejervarya limnocharis* | mismatch | Hualien County, Shoufeng Township | Taiwan | 23.8823 | 121.568 |
| G024 | *Fejervarya limnocharis (Clade II)* | *Fejervarya limnocharis* | Clade II | *Fejervarya limnocharis* | *Fejervarya limnocharis* | match | Hainan Island, China | China | 19 | 109 |
| JL02-1 | *Fejervarya limnocharis (Clade II)* | *Fejervarya limnocharis* | Clade II | *Fejervarya limnocharis* | *Fejervarya limnocharis* | match | Chubu, Shizuoka Prefecture, Central | Japan | 34.988 | 138.299 |
| NMNSF15460 | *Fejervarya limnocharis (Clade II)* | *Fejervarya limnocharis* | Clade II | *Fejervarya multistriata* | *Fejervarya multistriata* | mismatch | Pingtung County, Manjhou Township, Chiupeng | Taiwan | 22.1033 | 120.847 |
| fm1 | *Fejervarya limnocharis (Clade II)* | *Fejervarya limnocharis* | Clade II | *Fejervarya multistriata* | *Fejervarya multistriata* | mismatch | N/a, but original depositor is from Xian | China | 34.34 | 108.94 |
| TAD-P992 | *Fejervarya limnocharis (Clade II)* | *Fejervarya limnocharis* | Clade II | *Fejervarya limnocharis* | *Fejervarya limnocharis* | match | Phangnga, Namtok Raman Forest Park | Thailand | 8.45 | 98.52 |
| USNM:Herp:595381 | *Fejervarya limnocharis (Clade II)* | *Fejervarya limnocharis* | Clade II | *Fejervarya limnocharis* | *Fejervarya limnocharis* | match | south central extent of Trang An Landscape Complex | Vietnam | 20.25 | 105.9 |
| USNM:Herp:595383 | *Fejervarya limnocharis (Clade II)* | *Fejervarya limnocharis* | Clade II | *Fejervarya limnocharis* | *Fejervarya limnocharis* | match | Du Gia Forest, Bac Me, 3.2 km of Khau Ca Reserve (straight-line distance) | Vietnam | 22.8 | 105.3 |
| H001 | *Fejervarya multistriata (Clade II)* | *Fejervarya multistriata* | Clade II | *Fejervarya limnocharis* | *Fejervarya limnocharis* | mismatch | Taiwan Island | Taiwan | 23.7 | 121 |
| H002 | *Fejervarya multistriata (Clade II)* | *Fejervarya multistriata* | Clade II | *Fejervarya limnocharis* | *Fejervarya limnocharis* | mismatch | Taiwan Island | Taiwan | 23.7 | 121 |
| H005 | *Fejervarya multistriata (Clade II)* | *Fejervarya multistriata* | Clade II | *Fejervarya limnocharis* | *Fejervarya limnocharis* | mismatch | Taiwan Island | Taiwan | 23.7 | 121 |
| H011 | *Fejervarya multistriata (Clade II)* | *Fejervarya multistriata* | Clade II | *Fejervarya limnocharis* | *Fejervarya limnocharis* | mismatch | Taiwan Island | Taiwan | 23.7 | 121 |
| H012 | *Fejervarya multistriata (Clade II)* | *Fejervarya multistriata* | Clade II | *Fejervarya limnocharis* | *Fejervarya limnocharis* | mismatch | Taiwan Island | Taiwan | 23.7 | 121 |
| H013 | *Fejervarya multistriata (Clade II)* | *Fejervarya multistriata* | Clade II | *Fejervarya limnocharis* | *Fejervarya limnocharis* | mismatch | Taiwan Island | Taiwan | 23.7 | 121 |
| GZBY01 | *Fejervarya kawamurai (Clade II)* | *Fejervarya kawamurai* | Clade II | *Fejervarya multistrata* | *Fejervarya multistriata* | mismatch | Guangzhou | China | 23.13 | 113.26 |
| GZBY02 | *Fejervarya kawamurai (Clade II)* | *Fejervarya kawamurai* | Clade II | *Fejervarya multistrata* | *Fejervarya multistriata* | mismatch | Guangzhou | China | 23.13 | 113.26 |
| GZBY04 | *Fejervarya kawamurai (Clade II)* | *Fejervarya kawamurai* | Clade II | *Fejervarya multistrata* | *Fejervarya multistriata* | mismatch | Guangzhou | China | 23.13 | 113.26 |
| GZBY05 | *Fejervarya kawamurai (Clade II)* | *Fejervarya kawamurai* | Clade II | *Fejervarya multistrata* | *Fejervarya multistriata* | mismatch | Guangzhou | China | 23.13 | 113.26 |
| mtDNA | *Fejervarya multistriata (Clade I)* | *Fejervarya multistriata* | Clade I | *Fejervarya multistrata* | *Fejervarya multistriata* | match | Santai County, Mianyang City, Sichuan | China | 31.178 | 105.13 |
| ZWd1339 | *Fejervarya multistriata (Clade I)* | *Fejervarya multistriata* | Clade I | *Fejervarya multistrata* | *Fejervarya multistriata* | match | Chongming, Shanghai | China | 31.63 | 121.45 |
| MZ44_MZ | *Fejervarya multistriata (Clade I)* | *Fejervarya multistriata* | Clade I | *Fejervarya multistrata* | *Fejervarya multistriata* | match | Chongming, Shanghai | China | 31.63 | 121.45 |
| full mtDNA | *Fejervarya cancrivora* | *Fejervarya cancrivora* |  | *Fejervarya cf. cancrivora* | *Fejervarya cf. cancrivora* | mismatch | Beihai, Guangxi | China | 21.48 | 109.12 |
| MS-2009 (MS-can-Mal) | *Fejervarya cancrivora* | *Fejervarya cancrivora* |  | *Fejervarya cf. cancrivora* | *Fejervarya cf. cancrivora* | mismatch | Selangor | Malaysia | 3.07 | 101.52 |
| Jiadong-13 | *Fejervarya moodiei* | *Fejervarya moodiei* |  | *Fejervarya cancrivora* | *Fejervarya cancrivora* | mismatch | Jiadong | China | 22.85 | 113.1 |
| FCL-16S-1 | *Fejervarya cancrivora* | *Fejervarya cancrivora* |  | *Fejervarya cancrivora* | *Fejervarya cancrivora* | match | Cianjur, Java | Indonesia | -6.82 | 107.14 |
| FCL-16S-2 | *Fejervarya cancrivora* | *Fejervarya cancrivora* |  | *Fejervarya cancrivora* | *Fejervarya cancrivora* | match | West Sumatra, Padang | Indonesia | -0.95 | 100.36 |
| FCL-16S-7 | *Fejervarya cancrivora* | *Fejervarya cancrivora* |  | *Fejervarya cancrivora* | *Fejervarya cancrivora* | match | Central Java, Banyumas | Indonesia | -7.43 | 109.24 |
| IEBR A.5085 | *Fejervarya moodiei* | *Fejervarya moodiei* |  | *Fejervarya moodiei* | *Fejervarya moodiei* | match | Nam Dinh | Vietnam | 20.43 | 106.16 |
| IEBR A.5089 | *Fejervarya moodiei* | *Fejervarya moodiei* |  | *Fejervarya moodiei* | *Fejervarya moodiei* | match | Nam Dinh | Vietnam | 20.43 | 106.16 |
| IEBR A.5091 | *Fejervarya moodiei* | *Fejervarya moodiei* |  | *Fejervarya moodiei* | *Fejervarya moodiei* | match | Nam Dinh | Vietnam | 20.43 | 106.16 |
| IEBR A.5097 | *Fejervarya moodiei* | *Fejervarya moodiei* |  | *Fejervarya moodiei* | *Fejervarya moodiei* | match | Thua Thien Hue | Vietnam | 16.46 | 107.59 |
| IEBR A.5096 | *Fejervarya moodiei* | *Fejervarya moodiei* |  | *Fejervarya moodiei* | *Fejervarya moodiei* | match | Thua Thien Hue | Vietnam | 16.46 | 107.59 |
| IEBR A.5098 | *Fejervarya moodiei* | *Fejervarya moodiei* |  | *Fejervarya moodiei* | *Fejervarya moodiei* | match | Hai Phong | Vietnam | 20.85 | 106.68 |
| IEBR A.5099 | *Fejervarya moodiei* | *Fejervarya moodiei* |  | *Fejervarya moodiei* | *Fejervarya moodiei* | match | Hai Phong | Vietnam | 20.85 | 106.68 |
| IEBR A.5101 | *Fejervarya moodiei* | *Fejervarya moodiei* |  | *Fejervarya moodiei* | *Fejervarya moodiei* | match | Ca Mau | Vietnam | 9.18 | 105.15 |
| CG9CaMau | *Fejervarya moodiei* | *Fejervarya moodiei* |  | *Fejervarya moodiei* | *Fejervarya moodiei* | match | Ca Mau | Vietnam | 9.18 | 105.15 |
| full mtDNA | *Hoplobatrachus chinensis* | *Hoplobatrachus chinensis* | | *Hoplobatrachus rugulosus* | *Hoplobatrachus rugulosus* | mismatch | Jinhua, Zhejiang | China | 29.08 | 119.65 |
| full mtDNA | *Hoplobatrachus chinensis* | *Hoplobatrachus chinensis* | | *Hoplobatrachus rugulosus* | *Hoplobatrachus rugulosus* | mismatch | Anhui | China | 31.86 | 117.28 |
| full mtDNA | *Hoplobatrachus chinensis* | *Hoplobatrachus chinensis* | | *Hoplobatrachus chinensis x rugulosus* | *Hoplobatrachus chinensis x rugulosus* | mismatch | Jinhua, Zhejiang | China | 29.3608 | 119.9666 |
| JHBT-B4 | *Hoplobatrachus chinensis* | *Hoplobatrachus chinensis* | | *Hoplobatrachus rugulosus* | *Hoplobatrachus rugulosus* | mismatch | the Bendi farm in Jinhua, Zhejiang | China | 29.08 | 119.65 |
| JHBT-B3 | *Hoplobatrachus chinensis* | *Hoplobatrachus chinensis* | | *Hoplobatrachus rugulosus* | *Hoplobatrachus rugulosus* | mismatch | the Bendi farm in Jinhua, Zhejiang | China | 29.08 | 119.65 |
| JHBT-B2 | *Hoplobatrachus chinensis* | *Hoplobatrachus chinensis* | | *Hoplobatrachus rugulosus* | *Hoplobatrachus rugulosus* | mismatch | the Bendi farm in Jinhua, Zhejiang | China | 29.08 | 119.65 |
| JHBT-B1 | *Hoplobatrachus chinensis* | *Hoplobatrachus chinensis* | | *Hoplobatrachus rugulosus* | *Hoplobatrachus rugulosus* | mismatch | the Bendi farm in Jinhua, Zhejiang | China | 29.08 | 119.65 |
| FMNH 255191 | *Hoplobatrachus chinensis* | *Hoplobatrachus chinensis* | | *Hoplobatrachus rugulosus* | *Hoplobatrachus rugulosus* | mismatch | Laos: Champasak Province, Mounlapamok District, Dong Khanthung National Biodiversity Conservation Area | Laos | 14.1 | 105.85 |
| HN1 | *Hoplobatrachus chinensis* | *Hoplobatrachus chinensis* | | *Hoplobatrachus rugulosus* | *Hoplobatrachus rugulosus* | mismatch | the 'Hainan' farm in Jinhua, Zhejiang | China | 29 | 119 |
| HN2 | *Hoplobatrachus chinensis* | *Hoplobatrachus chinensis* | | *Hoplobatrachus rugulosus* | *Hoplobatrachus rugulosus* | mismatch | the 'Hainan' farm in Jinhua, Zhejiang | China | 29 | 119 |
| HN3 | *Hoplobatrachus chinensis* | *Hoplobatrachus chinensis* | | *Hoplobatrachus rugulosus* | *Hoplobatrachus rugulosus* | mismatch | the 'Hainan' farm in Jinhua, Zhejiang | China | 29 | 119 |
| HW1 | *Hoplobatrachus chinensis* | *Hoplobatrachus chinensis* | | *Hoplobatrachus rugulosus* | *Hoplobatrachus rugulosus* | mismatch | the Huwawa farm in Jinhua, Zhejiang | China | 29 | 119 |
| HW5 | *Hoplobatrachus chinensis* | *Hoplobatrachus chinensis* | | *Hoplobatrachus rugulosus* | *Hoplobatrachus rugulosus* | mismatch | the Huwawa farm in Jinhua, Zhejiang | China | 29 | 119 |
| HW6 | *Hoplobatrachus chinensis* | *Hoplobatrachus chinensis* | | *Hoplobatrachus rugulosus* | *Hoplobatrachus rugulosus* | mismatch | the Huwawa farm in Jinhua, Zhejiang | China | 29 | 119 |
| THW1 | *Hoplobatrachus chinensis* | *Hoplobatrachus chinensis* | | *Hoplobatrachus rugulosus* | *Hoplobatrachus rugulosus* | mismatch | the Huwawa farm in Jinhua, Zhejiang | China | 29 | 119 |
| NW1 | *Hoplobatrachus chinensis* | *Hoplobatrachus chinensis* | | *Hoplobatrachus rugulosus* | *Hoplobatrachus rugulosus* | mismatch | the Niuwa farm in Jinhua, Zhejiang | China | 29.05 | 119.5 |
| NW2 | *Hoplobatrachus chinensis* | *Hoplobatrachus chinensis* | | *Hoplobatrachus rugulosus* | *Hoplobatrachus rugulosus* | mismatch | the Niuwa farm in Jinhua, Zhejiang | China | 29.05 | 119.5 |
| JHBT-W7 | *Hoplobatrachus chinensis* | *Hoplobatrachus chinensis* | | *Hoplobatrachus rugulosus* | *Hoplobatrachus rugulosus* | mismatch | the Wu farm in Jinhua, Zhejiang | China | 29.1 | 119.4 |
| JHBT-W8 | *Hoplobatrachus chinensis* | *Hoplobatrachus chinensis* | | *Hoplobatrachus rugulosus* | *Hoplobatrachus rugulosus* | mismatch | the Wu farm in Jinhua, Zhejiang | China | 29.1 | 119.4 |
| WZ2 | *Hoplobatrachus chinensis* | *Hoplobatrachus chinensis* | | *Hoplobatrachus rugulosus* | *Hoplobatrachus rugulosus* | mismatch | Jinhua, Zhejiang | China | 29.3 | 119 |
| WZ3 | *Hoplobatrachus chinensis* | *Hoplobatrachus chinensis* | | *Hoplobatrachus rugulosus* | *Hoplobatrachus rugulosus* | mismatch | Jinhua, Zhejiang | China | 29.3 | 119 |
| full mtDNA | *Hoplobatrachus tigerinus* | *Hoplobatrachus tigerinus* | | *Hoplobatrachus tigerinus* | *Hoplobatrachus tigerinus* | match | Mymensingh | Bangladesh | 24.75 | 90.4 |
| KIZ021611 | *Quasipaa spinosa* | *Quasipaa spinosa* |  | *Quasipaa spinosa* | *Quasipaa spinosa* | match | Yuanbaoshan, Guangxi | China | 25.62 | 110.2 |
| KIZ021613 | *Quasipaa shini* | *Quasipaa shini* |  | *Quasipaa shini* | *Quasipaa shini* | match | Yuanbaoshan, Guangxi | China | 25.62 | 110.2 |
| KIZ022191 | *Quasipaa spinosa* | *Quasipaa spinosa* |  | *Quasipaa spinosa* | *Quasipaa spinosa* | match | Fulong, Guangxi | China | 22.55 | 110.15 |
| KIZ02340 | *Quasipaa yei* | *Quasipaa yei* |  | *Quasipaa yei* | *Quasipaa yei* | match | Shangcheng, Henan | China | 31.8 | 115.4 |
| KIZ02412 | *Quasipaa yei* | *Quasipaa yei* |  | *Quasipaa yei* | *Quasipaa yei* | match | Shangcheng, Henan | China | 31.8 | 115.4 |
| KIZ03107 | *Quasipaa boulengeri* | *Quasipaa boulengeri* |  | *Quasipaa boulengeri* | *Quasipaa boulengeri* | match | Youyang, Chongqing | China | 28.84 | 108.77 |
| KIZ04714 | *Quasipaa spinosa* | *Quasipaa spinosa* |  | *Quasipaa spinosa* | *Quasipaa spinosa* | match | Huizhou, Guangdong | China | 23.11 | 114.41 |
| KIZ08918 | *Quasipaa jiulongensis* | *Quasipaa jiulongensis* |  | *Quasipaa jiulongensis* | *Quasipaa jiulongensis* | match | Wuyishan, Fujian | China | 27.76 | 118.03 |
| KIZ08932 | *Quasipaa exilispinosa* | *Quasipaa exilispinosa* |  | *Quasipaa exilispinosa* | *Quasipaa exilispinosa* | match | Wuyishan, Fujian | China | 27.76 | 118.03 |
| KIZ08997 | *Quasipaa spinosa* | *Quasipaa spinosa* |  | *Quasipaa spinosa* | *Quasipaa spinosa* | match | Daiyunshan, Fujian | China | 25.43 | 118.24 |
| KIZ09096 | *Quasipaa spinosa* | *Quasipaa spinosa* |  | *Quasipaa spinosa* | *Quasipaa spinosa* | match | Qixingzhen, Guangdong | China | 23.03 | 114.41 |
| KIZ09282 | *Quasipaa spinosa* | *Quasipaa spinosa* |  | *Quasipaa spinosa* | *Quasipaa spinosa* | match | Chongzuo, Guangxi | China | 22.4 | 107.37 |
| KIZ09706 | *Quasipaa spinosa* | *Quasipaa spinosa* |  | *Quasipaa spinosa* | *Quasipaa spinosa* | match | Yangjiang, Guangdong | China | 21.86 | 111.97 |
| KIZJX0709008 | *Quasipaa spinosa* | *Quasipaa spinosa* |  | *Quasipaa spinosa* | *Quasipaa spinosa* | match | Lushan, Jiangxi | China | 29.55 | 115.98 |
| KIZYPX18607 | *Quasipaa spinosa* | *Quasipaa spinosa* |  | *Quasipaa spinosa* | *Quasipaa spinosa* | match | Xinying, Guangdong | China | 23.73 | 114.68 |
| KIZ01575 | *Quasipaa exilispinosa* | *Quasipaa exilispinosa* |  | *Quasipaa exilispinosa* | *Quasipaa exilispinosa* | match | Damaoshan, Hongkong | Hong Kong SAR of China | 22.5 | 114.2 |
| KIZ07219 | *Quasipaa exilispinosa* | *Quasipaa exilispinosa* |  | *Quasipaa exilispinosa* | *Quasipaa exilispinosa* | match | Xunwu, Jiangxi | China | 24.54 | 115.64 |
| KIZ08934 | *Quasipaa exilispinosa* | *Quasipaa exilispinosa* |  | *Quasipaa exilispinosa* | *Quasipaa exilispinosa* | match | Wuyishan, Fujian | China | 27.76 | 118.03 |
| KIZ08935 | *Quasipaa exilispinosa* | *Quasipaa exilispinosa* |  | *Quasipaa exilispinosa* | *Quasipaa exilispinosa* | match | Wuyishan, Fujian | China | 27.76 | 118.03 |
| KIZ08936 | *Quasipaa exilispinosa* | *Quasipaa exilispinosa* |  | *Quasipaa exilispinosa* | *Quasipaa exilispinosa* | match | Wuyishan, Fujian | China | 27.76 | 118.03 |
| KIZ08938 | *Quasipaa exilispinosa* | *Quasipaa exilispinosa* |  | *Quasipaa exilispinosa* | *Quasipaa exilispinosa* | match | Wuyishan, Fujian | China | 27.76 | 118.03 |
| KIZ08920 | *Quasipaa jiulongensis* | *Quasipaa jiulongensis* |  | *Quasipaa jiulongensis* | *Quasipaa jiulongensis* | match | Wuyishan, Fujian | China | 27.76 | 118.03 |
| KIZ02397 | *Quasipaa shini* | *Quasipaa shini* |  | *Quasipaa shini* | *Quasipaa shini* | match | Kaili, Guizhou | China | 26.58 | 107.97 |
| KIZ09282 | *Quasipaa spinosa* | *Quasipaa spinosa* |  | *Quasipaa spinosa* | *Quasipaa spinosa* | match | Chongzuo, Guangxi | China | 22.4 | 107.37 |
| KIZC21 | *Quasipaa spinosa* | *Quasipaa spinosa* |  | *Quasipaa spinosa* | *Quasipaa spinosa* | match | Huangshanpo, Anhui | China | 30.3 | 118.27 |
| KIZ021611 | *Quasipaa spinosa* | *Quasipaa spinosa* |  | *Quasipaa spinosa* | *Quasipaa spinosa* | match | China: Yuanbaoshan, Guangxi | China | 25.62 | 110.2 |
| HiroshimaShiga | *Fejervarya kawamurai (Clade I)* | *Fejervarya kawamurai* | Clade I | *Fejervarya kawamurai* | *Fejervarya kawamurai* | match | Shiga | Japan | 35 | 136 |
| Karasuma | *Fejervarya kawamurai (Clade I)* | *Fejervarya kawamurai* | Clade I | *Fejervarya kawamurai* | *Fejervarya kawamurai* | match | Shiga | Japan | 35 | 136 |
| JL02_1 | *Fejervarya kawamurai (Clade I)* | *Fejervarya kawamurai* | Clade I | *Fejervarya limnocharis* | *Fejervarya limnocharis* | mismatch | Chubu, Shizuoka Prefecture, Central Region, Shizuoka City, Shizuoka | Japan | 34.9884 | 138.299 |
| Okin | *Fejervarya kawamurai (Clade I)* | *Fejervarya kawamurai* | Clade I | *Fejervarya limnocharis* | *Fejervarya limnocharis* | mismatch | Okinawa | Japan | 26.5 | 127.9 |
| Okin_1_ | *Fejervarya kawamurai (Clade I)* | *Fejervarya kawamurai* | Clade I | *Fejervarya limnocharis* | *Fejervarya limnocharis* | mismatch | Okinawa | Japan | 26.5 | 127.9 |
| Okin_2_ | *Fejervarya kawamurai (Clade I)* | *Fejervarya kawamurai* | Clade I | *Fejervarya limnocharis* | *Fejervarya limnocharis* | mismatch | Okinawa | Japan | 26.5 | 127.9 |
| japo_higa | *Fejervarya kawamurai (Clade I)* | *Fejervarya kawamurai* | Clade I | *Fejervarya limnocharis* | *Fejervarya limnocharis* | mismatch | Higasihiroshima, Kagamiyama | Japan | 34.4 | 132.74 |
| japo_hiro | *Fejervarya kawamurai (Clade I)* | *Fejervarya kawamurai* | Clade I | *Fejervarya limnocharis* | *Fejervarya limnocharis* | mismatch | Hiroshima | Japan | 34.39 | 132.46 |
| Hiroshima_1 | *Fejervarya kawamurai (Clade I)* | *Fejervarya kawamurai* | Clade I | *Fejervarya sp.* | *Fejervarya sp.* | mismatch | Hiroshima | Japan | 34.39 | 132.46 |
| G024 | *Fejervarya multistriata (Clade II)* | *Fejervarya multistriata* | Clade II | *Fejervarya limnocharis* | *Fejervarya limnocharis* | mismatch | Hainan Island | China | 19.2 | 109.7 |
| HS12001 | *Fejervarya multistriata (Clade II)* | *Fejervarya multistriata* | Clade II | *Fejervarya multistriata* | *Fejervarya multistriata* | match | Santai County, Mianyang City | China | 31.1782 | 105.1305 |
| Husa | *Fejervarya multistriata (Clade II)* | *Fejervarya multistriata* | Clade II | *Fejervarya multistriata* | *Fejervarya multistriata* | match | Husa | China | 24.8 | 98.3 |
| zjbj3 | *Fejervarya multistriata (Clade II)* | *Fejervarya multistriata* | Clade II | *Fejervarya multistriata* | *Fejervarya multistriata* | match | Jian city, Jiangxi | China | 27.12 | 114.98 |
| 16SF15 | *Fejervarya limnocharis (Clade I)* | *Fejervarya limnocharis* | Clade I | *Fejervarya limnocharis* | *Fejervarya limnocharis* | match | West Kalimantan | Indonesia | 0.5 | 111 |
| 16SSG18 | *Fejervarya limnocharis (Clade I)* | *Fejervarya limnocharis* | Clade I | *Fejervarya limnocharis* | *Fejervarya limnocharis* | match | Sabah, Borneo | Malaysia | 5.5 | 117 |
| FMNH 261984 | *Fejervarya limnocharis (Clade I)* | *Fejervarya limnocharis* | Clade I | *Fejervarya limnocharis* | *Fejervarya limnocharis* | match | *No exact locality but verified ito be originated from Laos | Laos | 18 | 103 |
| MVZ226347 | *Fejervarya limnocharis (Clade I)* | *Fejervarya limnocharis* | Clade I | *Fejervarya limnocharis* | *Fejervarya limnocharis* | match | Southern Chinese Mainland | China | 30 | 112 |
| Malay | *Fejervarya limnocharis (Clade I)* | *Fejervarya limnocharis* | Clade I | *Fejervarya limnocharis* | *Fejervarya limnocharis* | match | Peninsular Malaysia | Malaysia | 4 | 102 |
| NA0083 | *Fejervarya limnocharis (Clade I)* | *Fejervarya limnocharis* | Clade I | *Fejervarya limnocharis* | *Fejervarya limnocharis* | match | Sarawak | Malaysia | 2.5 | 113 |
| NA0084 | *Fejervarya limnocharis (Clade I)* | *Fejervarya limnocharis* | Clade I | *Fejervarya limnocharis* | *Fejervarya limnocharis* | match | Sarawak | Malaysia | 2.5 | 113 |
| Rano | *Fejervarya limnocharis (Clade I)* | *Fejervarya limnocharis* | Clade I | *Fejervarya limnocharis* | *Fejervarya limnocharis* | match | Ranong | Thailand | 9.96 | 98.63 |
| SCUM04H001 | *Fejervarya limnocharis (Clade I)* | *Fejervarya limnocharis* | Clade I | *Fejervarya limnocharis* | *Fejervarya limnocharis* | match | Hainan Island | China | 19.2 | 109.7 |
| SH0034 | *Fejervarya limnocharis (Clade I)* | *Fejervarya limnocharis* | Clade I | *Fejervarya limnocharis* | *Fejervarya limnocharis* | match | Borneo | Malaysia | 1 | 114 |
| SNP0261 | *Fejervarya limnocharis (Clade I)* | *Fejervarya limnocharis* | Clade I | *Fejervarya limnocharis* | *Fejervarya limnocharis* | match | Sarawak | Malaysia | 2.5 | 113 |
| Thai 1 | *Fejervarya limnocharis (Clade I)* | *Fejervarya limnocharis* | Clade I | *Fejervarya limnocharis* | *Fejervarya limnocharis* | match | Nakhon Si Thammarat | Thailand | 8.43 | 99.96 |
| Thai 5 | *Fejervarya limnocharis (Clade I)* | *Fejervarya limnocharis* | Clade I | *Fejervarya limnocharis* | *Fejervarya limnocharis* | match | Sanam Chaiket | Thailand | 13.62 | 101.45 |
| Thai 6 | *Fejervarya limnocharis (Clade I)* | *Fejervarya limnocharis* | Clade I | *Fejervarya limnocharis* | *Fejervarya limnocharis* | match | Bangkok | Thailand | 13.7563 | 100.5018 |
| Thai 7 | *Fejervarya limnocharis (Clade I)* | *Fejervarya limnocharis* | Clade I | *Fejervarya limnocharis* | *Fejervarya limnocharis* | match | Sara Buri | Thailand | 14.53 | 100.91 |
| SH13_027 | *Fejervarya limnocharis (Clade I)* | *Fejervarya limnocharis* | Clade I | *Fejervarya limnocharis* | *Fejervarya limnocharis* | match | Borneo | Indonesia | 1 | 114 |
| lim 1 | *Fejervarya limnocharis (Clade I)* | *Fejervarya limnocharis* | Clade I | *Fejervarya cf. limnocharis* | *Fejervarya cf. limnocharis* | mismatch | Java | Indonesia | -7.5 | 110 |
| lim 10 | *Fejervarya limnocharis (Clade I)* | *Fejervarya limnocharis* | Clade I | *Fejervarya cf. limnocharis* | *Fejervarya cf. limnocharis* | mismatch | Sapa, | Vietnam | 22.34 | 103.84 |
| lim 11 | *Fejervarya limnocharis (Clade I)* | *Fejervarya limnocharis* | Clade I | *Fejervarya cf. limnocharis* | *Fejervarya cf. limnocharis* | mismatch | Phongsaly | Laos | 21.68 | 102.11 |
| lim 12 | *Fejervarya limnocharis (Clade I)* | *Fejervarya limnocharis* | Clade I | *Fejervarya cf. limnocharis* | *Fejervarya cf. limnocharis* | mismatch | Cambodia | Cambodia | 12.57 | 104.99 |
| lim 2 | *Fejervarya limnocharis (Clade I)* | *Fejervarya limnocharis* | Clade I | *Fejervarya cf. limnocharis* | *Fejervarya cf. limnocharis* | mismatch | Java | Indonesia | -7.5 | 110 |
| lim 3 | *Fejervarya limnocharis (Clade I)* | *Fejervarya limnocharis* | Clade I | *Fejervarya cf. limnocharis* | *Fejervarya cf. limnocharis* | mismatch | Kuala Lumpur | Malaysia | 3.14 | 101.69 |
| lim 4 | *Fejervarya limnocharis (Clade I)* | *Fejervarya limnocharis* | Clade I | *Fejervarya cf. limnocharis* | *Fejervarya cf. limnocharis* | mismatch | Kuala Lumpur | Malaysia | 3.14 | 101.69 |
| lim 5 | *Fejervarya limnocharis (Clade I)* | *Fejervarya limnocharis* | Clade I | *Fejervarya cf. limnocharis* | *Fejervarya cf. limnocharis* | mismatch | Ranong | Thailand | 9.96 | 98.63 |
| lim 6 | *Fejervarya limnocharis (Clade I)* | *Fejervarya limnocharis* | Clade I | *Fejervarya cf. limnocharis* | *Fejervarya cf. limnocharis* | mismatch | Tha Ton | Thailand | 20.03 | 99.18 |
| lim 8 | *Fejervarya limnocharis (Clade I)* | *Fejervarya limnocharis* | Clade I | *Fejervarya cf. limnocharis* | *Fejervarya cf. limnocharis* | mismatch | Phang Nga | Thailand | 8.45 | 98.53 |
| lim 9 | *Fejervarya limnocharis (Clade I)* | *Fejervarya limnocharis* | Clade I | *Fejervarya cf. limnocharis* | *Fejervarya cf. limnocharis* | mismatch | Phang Nga | Thailand | 8.45 | 98.53 |
| limno | *Fejervarya limnocharis (Clade I)* | *Fejervarya limnocharis* | Clade I | *Fejervarya cf. limnocharis* | *Fejervarya cf. limnocharis* | mismatch | Java | Indonesia | -7.5 | 110 |

**References**

1.	van Dijk PP, Iskandar D, Inger RF, Lau MWN, Ermi Z, Baorong G, et al. *Fejervarya limnocharis*, Asian Grass Frog. IUCN Red List Threat Species. 2004; e.T58275A86154107. Available: http://dx.doi.org/10.2305/IUCN.UK.2004.RLTS.T58275A11747569.en

2.	Auliya M, Altherr S, Nithart C, Hughes A, Bickford D. Numerous uncertainties in the multifaceted global trade in frogs’ legs with the EU as the major consumer. Nat Conserv. 2023;51: 71–135. doi:10.3897/natureconservation.51.93868

3.	Yodthong S, Stuart BL, Aowphol A. Species delimitation of crab-eating frogs (*Fejervarya cancrivora* complex) clarifies taxonomy and geographic distributions in mainland Southeast Asia. Zookeys. 2019; 119–153. doi:10.3897/zookeys.883.37544

4.	Yu D, Zhang J, Li P, Zheng R, Shao C. Do cryptic species exist in *Hoplobatrachus rugulosus*? An examination using four nuclear genes, the Cyt b gene and the complete MT genome. PLoS One. 2015;10: e0124825. doi:10.1371/journal.pone.0124825

5.	Djong HT, Matsui M, Kuramoto M, Nishioka M, Djong HT, Matsui M, et al. A New Species of the *Fejervarya limnocharis* Complex from Japan (Anura, Dicroglossidae). Zoolog Sci. 2011;28: 922–929. doi:10.2108/zsj.28.922

6.	Dufresnes C, Litvinchuk SN. Diversity, distribution and molecular species delimitation in frogs and toads from the Eastern Palaearctic. Zool J Linn Soc. 2022;195: 695–760. doi:10.1093/zoolinnean/zlab083

7.	Borzée A. Continental Northeast Asian amphibians: Origins, behavioural ecology, and conservation. 1st ed. Academic Press Elsevier; 2024.

8.	IUCN. The IUCN Red List of Threatened Species. Version 2024-1. 2024 [cited 16 May 2021]. Available: https://www.iucnredlist.org

9.	Tong H, Wang K, Xu J, Guo J, Guo Y, Shi S, et al. A new species in the *Fejervarya limnocharis* species complex (Amphibia: Dicroglossidae). Zootaxa. 2025;5660: 529–546. doi:10.11646/zootaxa.5660.4.4

10.	Köhler G, Mogk L, Khaing KPP, Than NL. The genera *Fejervarya* and *Minervarya* in Myanmar: Description of a new species, new country records, and taxonomic notes (Amphibia, Anura, Dicroglossidae). Vertebr Zool. 2019;69: 183–226. doi:10.26049/VZ69-2-2019-05

11.	Bouckaert R, Vaughan TG, Barido-Sottani J, Duchêne S, Fourment M, Gavryushkina A, et al. BEAST 2.5: An advanced software platform for Bayesian evolutionary analysis. PLoS Comput Biol. 2019;15: e1006650. doi:10.1371/journal.pcbi.1006650

12.	Bouckaert RR, Drummond AJ. bModelTest: Bayesian phylogenetic site model averaging and model comparison. BMC Evol Biol. 2017;17: 42. doi:10.1186/s12862-017-0890-6

13.	Ogilvie HA, Bouckaert RR, Drummond AJ. StarBEAST2 brings faster species tree inference and accurate estimates of substitution rates. Mol Biol Evol. 2017;34: 2101–2114. doi:10.1093/molbev/msx126

14.	Skilling J. Nested sampling for general Bayesian computation. Bayesian Anal. 2006;1: 833–860. doi:10.1214/06-BA127

15.	Maturana P, Brewer BJ, Klaere S, Bouckaert RR. Model selection and parameter inference in phylogenetics using nested sampling. Syst Biol. 2019;68: 219–233. doi:10.1093/sysbio/syy050

16.	Chen S, Zhou Y, Chen Y, Gu J. Fastp: An ultra-fast all-in-one FASTQ preprocessor. Bioinformatics. 2018;34: i884–i890. doi:10.1093/bioinformatics/bty560

17.	Li H, Durbin R. Fast and accurate short read alignment with Burrows-Wheeler transform. Bioinformatics. 2009;25: 1754–1760. doi:10.1093/bioinformatics/btp324

18.	Catchen J, Hohenlohe PA, Bassham S, Amores A, Cresko WA. Stacks: An analysis tool set for population genomics. Mol Ecol. 2013;22: 3124–3140. doi:10.1111/mec.12354

19.	McKenna A, Hanna M, Banks E, Sivachenko A, Cibulskis K, Kernytsky A, et al. The Genome Analysis Toolkit: A MapReduce framework for analyzing next-generation DNA sequencing data. Genome Res. 2010;20: 1297–1303. doi:10.1101/gr.107524.110.20

20.	Li H, Handsaker B, Wysoker A, Fennell T, Ruan J, Homer N, et al. The Sequence Alignment/Map format and SAMtools. Bioinformatics. 2009;25: 2078–2079. doi:10.1093/bioinformatics/btp352

21.	Broad Institute. Picard Toolkit. In: Github repository [Internet]. 2025. Available: https://broadinstitute.github.io/picard/

22.	Kim D, Paggi JM, Park C, Bennett C, Salzberg SL. Graph-based genome alignment and genotyping with HISAT2 and HISAT-genotype. Nat Biotechnol. 2019;37: 907–915. doi:10.1038/s41587-019-0201-4

23.	Danecek P, Auton A, Abecasis G, Albers CA, Banks E, DePristo MA, et al. The variant call format and VCFtools. Bioinformatics. 2011;27: 2156–2158. doi:10.1093/bioinformatics/btr330

24.	Danecek P, Bonfield JK, Liddle J, Marshall J, Ohan V, Pollard MO, et al. Twelve years of SAMtools and BCFtools. Gigascience. 2021;10: 1–4. doi:10.1093/gigascience/giab008

25.	Li H. Tabix: Fast retrieval of sequence features from generic TAB-delimited files. Bioinformatics. 2011;27: 718–719. doi:10.1093/bioinformatics/btq671

26.	Leaché AD, Banbury BL, Felsenstein J, De Oca ANM, Stamatakis A. Short tree, long tree, right tree, wrong tree: New acquisition bias corrections for inferring SNP phylogenies. Syst Biol. 2015;64: 1032–1047. doi:10.1093/sysbio/syv053

27.	Harvey MG, Smith BT, Glenn TC, Faircloth BC, Brumfield T, Rouge B, et al. Sequence capture versus Restriction Site Associated DNA sequencing for shallow systematics. Syst Biol. 2016;65: 910–924. Available: https://doi.org/10.1093/sysbio/syw036

28.	Ortiz EM. vcf2phylip v2.0: convert a VCF matrix into several matrix formats for phylogenetic analysis. In: Zenodo. 2019 p. https://doi.org/10.5281/zenodo.2540861.

29.	Chen Z, Li H, Zhu Y, Feng Q, He Y, Chen X. Molecular phylogeny of the family Dicroglossidae (Amphibia: Anura) inferred from complete mitochondrial genomes. Biochem Syst Ecol. 2017;71: 1–9. doi:10.1016/j.bse.2017.01.006

30.	Feng YJ, Blackburn DC, Liang D, Hillis DM, Wake DB, Cannatella DC, et al. Phylogenomics reveals rapid, simultaneous diversification of three major clades of Gondwanan frogs at the Cretaceous–Paleogene boundary. Proc Natl Acad Sci U S A. 2017;114: E5864–E5870. doi:10.1073/pnas.1704632114

31.	Othman SN, Litvinchuk SN, Maslova I, Dahn H, Messenger KR, Andersen D, et al. From Gondwana to the Yellow Sea, evolutionary diversifications of true toads *Bufo* sp. in the Eastern Palearctic and a revisit of species boundaries for Asian lineages. Elife. 2022;11: e70494. doi:10.7554/eLife.70494

32.	Matthews KJ, Maloney KT, Zahirovic S, Williams SE, Seton M, Müller RD. Global plate boundary evolution and kinematics since the late Paleozoic. Glob Planet Change. 2016;146: 226–250. doi:10.1016/j.gloplacha.2016.10.002

33.	QGIS Development Team. QGIS Geographic Information System. Open Source Geospatial Foundation Project. http://qgis.osgeo.org. 2024. Available: https://www.qgis.org

34.	Yan F, Zhou W, Zhao H, Yuan Z, Wang Y, Jiang K, et al. Geological events play a larger role than Pleistocene climatic fluctuations in driving the genetic structure of *Quasipaa boulengeri* (Anura: Dicroglossidae). Mol Ecol. 2013;22: 1120–1133. doi:10.1111/mec.12153

35.	Heath TA, Huelsenbeck JP, Stadler T. The fossilized birth-death process for coherent calibration of divergence-time estimates. Proc Natl Acad Sci U S A. 2014;111: E2957–E2966. doi:10.1073/pnas.1319091111

36.	Rambaut A, Drummond AJ, Xie D, Baele G, Suchard MA. Posterior summarization in Bayesian phylogenetics using Tracer 1.7. Syst Biol. 2018;67: 901–904. doi:10.1093/sysbio/syy032

37.	Baele G, Lemey P, Vansteelandt S. Make the most of your samples: Bayes factor estimators for high-dimensional models of sequence evolution. BMC Bioinformatics. 2013;14: 1–18. doi:10.1186/1471-2105-14-85

38.	Fan Y, Wu R, Chen MH, Kuo L, Lewis PO. Choosing among partition models in Bayesian phylogenetics. Mol Biol Evol. 2011;28: 523–532. doi:10.1093/molbev/msq224

39.	Xu Z, Wu H, Abulike K, Othman SN, Borzée A, Wang J. Assessing the effectiveness of remote sensing variables for coarse-scale amphibian distribution modeling: A case study of *Fejervarya multistriata* in China. J Environ Manage. 2025;392: 126809. doi:10.1016/j.jenvman.2025.126809

40.	Borzée A, Andersen D, Jang Y. Population trend inferred from aural surveys for calling anurans in Korea. PeerJ. 2018; e5568. doi:10.7717/peerj.5568

41.	Kramer-Schadt S, Niedballa J, Pilgrim JD, Schröder B, Lindenborn J, Reinfelder V, et al. The importance of correcting for sampling bias in MaxEnt species distribution models. Divers Distrib. 2013;19: 1366–1379. doi:10.1111/ddi.12096

42.	ESRI. ArcGIS Desktop: Release 10. In: Redlands, CA: Environmental Systems Research Institute. 2011.

43.	Fick SE, Hijmans RJ. WorldClim 2: new 1-km spatial resolution climate surfaces for global land areas. Int J Climatol. 2017;37: 4302–4315. doi:10.1002/joc.5086

44.	Abrams M, Crippen R, Fujisada H. ASTER Global Digital Elevation Model (GDEM) and ASTER Global Water Body Dataset (ASTWBD). Remote Sens. 2020;12: 1156. doi:10.3390/rs12071156

45.	Merow C, Smith MJ, Silander JA. A practical guide to MaxEnt for modeling species’ distributions: What it does, and why inputs and settings matter. Ecography (Cop). 2013;36: 1058–1069. doi:10.1111/j.1600-0587.2013.07872.x

46.	R Core Team. R: A language and environment for statistical computing. URL https://www.R-project.org/. R Foundation for Statistical Computing. Vienna, Austria; 2024. Available: https://www.r-project.org/.

47.	Phillips SB, Aneja VP, Kang D, Arya SP. Maximum entropy modeling of species geographic distributions. Ecol Modell. 2006;190: 231–259. doi:10.1016/j.ecolmodel.2005.03.026

48.	Mandrekar JN. Receiver operating characteristic curve in diagnostic test assessment. J Thorac Oncol. 2010;5: 1315–1316. doi:10.1097/JTO.0b013e3181ec173d

49.	Allouche O, Tsoar A, Kadmon R. Assessing the accuracy of species distribution models: Prevalence, kappa and the true skill statistic (TSS). J Appl Ecol. 2006;43: 1223–1232. doi:10.1111/j.1365-2664.2006.01214.x

50.	Liu C, Newell G, White M. On the selection of thresholds for predicting species occurrence with presence-only data. Ecol Evol. 2016;6: 337–348. doi:10.1002/ece3.1878

51.	Liu C, White M, Newell G. Selecting thresholds for the prediction of species occurrence with presence-only data. J Biogeogr. 2013;40: 778–789. doi:10.1111/jbi.12058

52.	Liu C, Berry PM, Dawson TP, Pearson RG. Selecting thresholds of occurrence in the prediction of species distributions. Ecography (Cop). 2005;28: 385–393. doi:10.1111/j.0906-7590.2005.03957.x

53.	Cai YT, Ma L, Xu CJ, Li P, Zhang JY, Storey KB, et al. The complete mitochondrial genome of the hybrid of *Hoplobatrachus chinensis* (♀)× *H. rugulosus* (♂) and its phylogeny. Mitochondrial DNA Part B Resour. 2018;3: 344–345. doi:10.1080/23802359.2018.1450661

54.	Osbeck P. Reise nach Ostindien und China. Nebst O. Toreens Reise nach Suratte und C. G. Ekebergs nachricht von der Landwirthschaft der Chinesen. Aus dem Schwedischen Übersetzt von J. G. Georgi. Rostock: J. C. Koppe. 1765.

55.	Wiegmann AFA. “Amphibien,” in Reise um die Erde ausfeführt auf dem Königlich Preussischen Seehandlungs-Schiffe Prinzes Louise, comandiert von Captain W. Wendt, in den Jahren 1830, 1831 und 1832 Dritter Theil. von Dr. F. J. F. Meyen. In: Meyen FJF, editor. Zoologisher Bericht. Berlin: Sander’schen Buchhandlung (C.W. Eichhoff); 1834. p. Pp. 512.

56.	Dubois A. It is high time that taxonomists follow the Code. 4. Amphibia of Indochina. Bionomina. 2021;24: 38–50.

57.	Yang K, Wo Y, Shao G, Liao P, Tong H, Brown RP, et al. Phylogenetic relationships among Chinese Rice Frogs within the *Fejervarya limnocharis* species complex (Amphibia: Dicroglossidae). Asian Herpetol Res. 2022;13: 232–241. doi:10.16373/j.cnki.ahr.210050

58.	Liu Q, Feng H, Jin L, Mi ZP, Zhou ZM, Bo Liao W. Latitudinal variation in body size in *Fejervarya limnocharis supports* the inverse of Bergmann’s rule. Anim Biol. 2018;68: 113–128. doi:10.1163/15707563-17000129

59.	Zhao N, Ma Z, Jiang Y, Shi Y, Xie Y, Wang Y, et al. Geographical patterns of *Fejervarya limnocharis* gut microbiota by latitude along mainland China’s coastline. Front Microbiol. 2022;13: 1062302. doi:10.3389/fmicb.2022.1062302

60.	Wu Z, Li Y, Murray BR. Insular shifts in body size of rice frogs in the Zhoushan Archipelago, China. J Anim Ecol. 2006;75: 1071–1080. doi:10.1111/j.1365-2656.2006.01126.x

61.	Sumida M, Kotaki M, Islam MM, Djong TH, Igawa T, Kondo Y, et al. Evolutionary relationships and reproductive isolating mechanisms in the rice frog (*Fejervarya limnocharis*) species complex from Sri Lanka, Thailand, Taiwan and Japan, inferred from mtDNA gene sequences, allozymes, and crossing experiments. Zoolog Sci. 2007;24: 547–562. doi:10.2108/zsj.24.547

62.	Chang CW, Huang BH, Lin SM, Huang CL, Liao PC. Changes of diet and dominant intestinal microbes in farmland frogs. BMC Microbiol. 2016;16: 1–13. doi:10.1186/s12866-016-0660-4

63.	Toda M, Nishida M, Matsui M, Wu GF, Ota H. Allozyme variation among East Asian populations of the Indian rice frog, *Rana limnocharis* (Amphibia: Anura). Biochem Syst Ecol. 1997;25: 143–159. doi:10.1016/S0305-1978(96)00098-1

64.	Dubois A, Ohler A. Systematics of *Fejervarya limnocharis* (Gravenhorst, 1829) (Amphibia, Anura, Ranidae) and related species. 1. Nomenclatural status and type-specimens of the nominal species *Rana limnocharis* Gravenhorst, 1829. Alytes. 2000;18: 15–50.

65.	Iskandar D. The amphibians of Java and Bali. Bandung, Indonesia: Research and Development Centre for Biology- LIP! and GEF- Biodiversity Collections Project, Bogor; 1998.

66.	Taylor EH. Philippine Amphibia. Philipp J Sci. 1920;16: 213–359.

67.	McCoy CJ, Richmond ND. Herpetological type specimens in Carnegie Museum. Ann Carnegie Museum Pittsburgh. 1966;38: 233–264.

68.	Chandramouli SR, Ankaiah D, Prasad KVD, Arul V. On the identity of two *Fejervarya* Frog (Dicroglossidae) species from the Andaman and Nicobar Archipelago. Taprobanica. 2020;09: 194–204. doi:10.47605/tapro.v9i2.231

69.	Chung H Van, Cuong PT, Truong NQ. Additional records and phylogenetic relationships of *Fejervarya moodiei* (Taylor, 1920) (Anura: Dicroglossidae) from Vietnam. J For Sci Technol. 2022; 3–13. doi:10.55250/jo.vnuf.2022.14.003-013

70.	Sah AK, Gautam B, Bhattarai S. Journal of Animal Diversity. J Anim Divers. 2021;3. doi:10.29252/JAD.2020.2.2.2

71.	Dittrich C, Struck U, Rödel MO. Stable isotope analyses-A method to distinguish intensively farmed from wild frogs. Ecol Evol. 2017;7: 2525–2534. doi:10.1002/ece3.2878

72.	Zheng Y, Zhang S, Lu Q, Zhang S, Wang L, Hong M, et al. Population genetic patterns of a mangrove-associated frog reveal its colonization history and habitat connectivity. Divers Distrib. 2021;27: 1584–1600. doi:10.1111/ddi.13304

73.	Kurniawan N, Djong TH, Islam MM, Nishizawa T, Belabut DM, Sen YH, et al. Taxonomic status of three types of *Fejervarya cancrivora* from Indonesia and other Asian countries based on morphological observations and crossing experiments. Zoolog Sci. 2011;28: 12–24. doi:10.2108/zsj.28.12

74.	Li J, Wang X, Lan T, Lu Y, Hong M, Ding L, et al. CDK5/NFAT5‐regulated transporters involved in osmoregulation in *Fejervarya cancrivora*. Biology (Basel). 2022;11: 858. doi:10.3390/biology11060858

75.	Pratomo H, Susilo A, Rosadi B. Invertebrate diversity in gastrointestinal *Fejervarya cancrivora* rice fields in Ciomas, Bogor Regency. E3S Web Conf. 2024;483: 01007.

76.	Zhong J, Liu ZQ, Wang YQ. Phylogeography of the rice frog, *Fejervarya multistriata* (Anura: Ranidae), from China based on mtDNA D-loop sequences. Zoolog Sci. 2008;25: 811–820. doi:10.2108/zsj.25.811

77.	Zhang H, Yan J, Zhang G, Zhou K. Phylogeography and demographic history of Chinese black-spotted frog populations (*Pelophylax nigromaculata*): evidence for independent refugia expansion and secondary contact. BMC Evol Biol. 2008;8: 21.

78.	Sumida M, Kondo Y, Kanamori Y, Nishioka M. Inter- and intraspecific evolutionary relationships of the rice frog *Rana limnocharis* and the allied species *R. cancrivora* inferred from crossing experiments and mitochondrial DNA sequences of the *12S* and *16S* rRNA genes. Mol Phylogenet Evol. 2002;25: 293–305. doi:10.1016/S1055-7903(02)00243-9

79.	Kotaki M, Kurabayashi A, Matsui M, Khonsue W, Djong TH, Tandon M, et al. Genetic divergences and phylogenetic relationships among the *Fejervarya limnocharis* complex in Thailand and neighboring countries revealed by mitochondrial and nuclear genes. Zoolog Sci. 2008;25: 381–390. doi:10.2108/zsj.25.381

80.	Bossuyt F, Milinkovitch MC. Convergent adaptive radiations in Madagascan and Asian ranid frogs reveal covariation between larval and adult traits. Proc Natl Acad Sci U S A. 2000;97: 6585–6590. doi:10.1073/pnas.97.12.6585

81.	Yang X, Luo W, Xia Y, Zeng X. Using sex-linked markers via Genotyping-by-Sequencing to identify XX/XY Sex chromosomes in the Spiny frog (*Quasipaa boulengeri*). Genes (Basel). 2022;13. doi:10.3390/genes13040575

82.	Shao Y, Wang LJ, Zhong L, Hong ML, Chen HM, Murphy RW, et al. Transcriptomes reveal the genetic mechanisms underlying ionic regulatory adaptations to salt in the crab-eating frog. Sci Rep. 2015;5: 17551. doi:10.1038/srep17551

83.	Lau Q, Igawa T, Kosch TA, Dharmayanthi AB, Berger L, Skerratt LF, et al. Conserved evolution of MHC supertypes among Japanese frogs suggests selection for Bd resistance. Animals. 2023;13: 2121. doi:10.3390/ani13132121

84.	Tang Y, Chen JY, Ding GH, Lin ZH. Analyzing the gonadal transcriptome of the frog *Hoplobatrachus rugulosus* to identify genes involved in sex development. BMC Genomics. 2021;22: 552. doi:10.1186/s12864-021-07879-6

85.	Wang X, Xie Y, Hu W, Wei Z, Wei X, Yuan H, et al. Transcriptome characterization and SSR discovery in the giant spiny frog *Quasipaa spinosa*. Gene. 2022;842: 146793. doi:10.1016/j.gene.2022.146793
