# Supplementary material for: Model-based multilocus, genomic, and phenotypic integration reveals species assignment inconsistencies and sampling blind spots across Asian ecozones in Dicroglossidae
Source: PLoS One. 2026 Oct 23;21(10):e0360243. doi: 10.1371/journal.pone.0360243 (PMC13693946; doi:10.1371/journal.pone.0360243)
Supplement: Supplementary file 2 [file pone.0360243.s003.docx]

PONE-D-26-21504

Model-based multilocus, genomic, and phenotypic integration reveals species assignment inconsistencies and sampling blind spots across Asian ecozones in Dicroglossidae

PLOS One

Dear Dr. Borzée,

Thank you for submitting your manuscript to PLOS ONE. After careful consideration, we feel that it has merit but does not fully meet PLOS ONE’s publication criteria as it currently stands. Therefore, we invite you to submit a revised version of the manuscript that addresses the points raised during the review process.

The manuscript was evaluated by two expert reviewers. While one reviewer provided only minor comments, the other reviewer has raised major concerns that need to be carefully considered and thoroughly discussed in the revised version. In summary, these concerns relate to data sampling, morphological and biological evidence considered, analytical issues, and interpretation and discussion of some results.

Please submit your revised manuscript by Aug 25 2026 11:59PM. If you will need more time than this to complete your revisions, please reply to this message or contact the journal office at [plosone@plos.org](mailto:plosone@plos.org). When you're ready to submit your revision, log on to <https://www.editorialmanager.com/pone/> and select the 'Submissions Needing Revision' folder to locate your manuscript file.

Please include the following items when submitting your revised manuscript:

- A letter that responds to each point raised by the academic editor and reviewer(s). You should upload this letter as a separate file labeled 'Response to Reviewers'.
- A marked-up copy of your manuscript that highlights changes made to the original version. You should upload this as a separate file labeled 'Revised Manuscript with Track Changes'.
- An unmarked version of your revised paper without tracked changes. You should upload this as a separate file labeled 'Manuscript'.

If you would like to make changes to your financial disclosure, please include your updated statement in your cover letter. Guidelines for resubmitting your figure files are available below the reviewer comments at the end of this letter.

If applicable, we recommend that you deposit your laboratory protocols in [protocols.io](http://protocols.io/) to enhance the reproducibility of your results. Protocols.io assigns your protocol its own identifier (DOI) so that it can be cited independently in the future. For instructions see: [https://journals.plos.org/plosone/s/submission-guidelines#loc-laboratory-protocols](https://track.editorialmanager.com/CL0/https:%2F%2Fjournals.plos.org%2Fplosone%2Fs%2Fsubmission-guidelines%23loc-laboratory-protocols/1/010f019f07327bde-d420bfdf-d6ef-449b-9734-24b129d77121-000000/jh8hZ7S49DncJxykzuj4jB48fB6UcGxK-jsBhY0oUKo=258). Additionally, PLOS ONE offers an option for publishing peer-reviewed Lab Protocol articles, which describe protocols hosted on [protocols.io](http://protocols.io/). Read more information on sharing protocols at [https://plos.org/protocols?utm_medium=editorial-email&utm_source=authorletters&utm_campaign=protocols](https://track.editorialmanager.com/CL0/https:%2F%2Fplos.org%2Fprotocols%3Futm_medium=editorial-email%26utm_source=authorletters%26utm_campaign=protocols/1/010f019f07327bde-d420bfdf-d6ef-449b-9734-24b129d77121-000000/K1fgtbf-m6pdl989LTUCmN4pJuRE9IeZzTjzfxIgP2U=258).

As the corresponding author, your ORCID iD is verified in the submission system and will appear in the published article. PLOS supports the use of ORCID, and we encourage all coauthors to register for an ORCID iD and use it as well. Please encourage your coauthors to verify their ORCID iD within the submission system before final acceptance, as unverified ORCID iDs will not appear in the published article. *Only* the individual author can complete the verification step; PLOS staff *cannot* verify ORCID iDs on behalf of authors.

We look forward to receiving your revised manuscript.

Kind regards,

Martín O. Pereyra
Academic Editor
PLOS One

A: We thank the Academic Editor and both reviewers for their constructive comments. We have carefully addressed the comments from both reviewers and substantially revised the manuscript. In addition to the revisions addressing Reviewer 1’s comments, the major concerns raised by Reviewer 2 regarding sampling, biological evidence, genomic data processing, analytical robustness, and interpretation prompted further clarification and additional analyses.

For sampling, we have clarified that the study primarily uses publicly available molecular and genomic data across broad geographic and taxonomic scales, complemented by our targeted field sampling at key geographic boundaries. We have also clarified the processing and limitations of the heterogeneous genomic datasets and conducted an additional missing-data and locus-recovery analysis, now presented in S2 Fig and S10 Table. The SRA-derived genomic data are now explicitly presented as complementary evidence within the total-evidence framework. Finally, we strengthened the treatment of morphological and bioacoustic evidence, sampling limitations, and geographic gaps, while moderating our conclusions to better reflect the scope of the available data. Detailed point-by-point responses to Reviewers 1 and 2 are provided below, with the corresponding revisions indicated by line numbers in the tracked-changes version of the manuscript.

**Journal Requirements:**

When submitting your revision, we need you to address these additional requirements.

1. Please ensure that your manuscript meets PLOS ONE's style requirements, including those for file naming. The PLOS ONE style templates can be found at

[https://journals.plos.org/plosone/s/file?id=wjVg/PLOSOne_formatting_sample_main_body.pdf](https://track.editorialmanager.com/CL0/https:%2F%2Fjournals.plos.org%2Fplosone%2Fs%2Ffile%3Fid=wjVg%2FPLOSOne_formatting_sample_main_body.pdf/1/010f019f07327bde-d420bfdf-d6ef-449b-9734-24b129d77121-000000/d9uVQSPFm9CMzjN-6UELZ0Obc2WDN6eLk0PED9GmrAM=258) and

[https://journals.plos.org/plosone/s/file?id=ba62/PLOSOne_formatting_sample_title_authors_affiliations.pdf](https://track.editorialmanager.com/CL0/https:%2F%2Fjournals.plos.org%2Fplosone%2Fs%2Ffile%3Fid=ba62%2FPLOSOne_formatting_sample_title_authors_affiliations.pdf/1/010f019f07327bde-d420bfdf-d6ef-449b-9734-24b129d77121-000000/WeKi12VH9PfbGFjlq2o6QNANtfY5sRQe6OYMjzJOZSo=258)

2. To comply with PLOS One submissions requirements, in your Methods section, please provide additional information regarding the experiments involving animals and ensure you have included details on (1) methods of sacrifice, (2) methods of anesthesia and/or analgesia, and (3) efforts to alleviate suffering.

A: We have added clarification regarding animal handling in the Methods. All specimens used for tissue sampling were collected after being found dead as roadkills; therefore, no live animals were euthanised for tissue collection, and anesthesia, analgesia, and procedures to alleviate suffering were not applicable (Lines 138–140). For the live individuals used for bioacoustic sampling, DNA samples were obtained non-lethally by swabbing the frogs, after which individuals were released at the sampling site (Lines 150–152).

3. We note that the grant information you provided in the ‘Funding Information’ and ‘Financial Disclosure’ sections do not match.

When you resubmit, please ensure that you provide the correct grant numbers for the awards you received for your study in the ‘Funding Information’ section.

4. Please note that funding information should not appear in any section or other areas of your manuscript. We will only publish funding information present in the Funding Statement section of the online submission form. Please remove any funding-related text from the manuscript.

A: We have removed all funding-related information from the manuscript. Funding information is provided only in the Funding Statement section of the online submission form.

5. We note that Figures 2, 3,and 4 in your submission contain copyrighted images. All PLOS content is published under the Creative Commons Attribution License (CC BY 4.0), which means that the manuscript, images, and Supporting Information files will be freely available online, and any third party is permitted to access, download, copy, distribute, and use these materials in any way, even commercially, with proper attribution. For more information, see our copyright guidelines: <http://journals.plos.org/plosone/s/licenses-and-copyright>.

We require you to either (1) present written permission from the copyright holder to publish these figures specifically under the CC BY 4.0 license, or (2) remove the figures from your submission:

1. You may seek permission from the original copyright holder of Figures 2, 3, and 4 to publish the content specifically under the CC BY 4.0 license.

We recommend that you contact the original copyright holder with the Content Permission Form (<http://journals.plos.org/plosone/s/file?id=7c09/content-permission-form.pdf>) and the following text:

“I request permission for the open-access journal PLOS ONE to publish XXX under the Creative Commons Attribution License (CCAL) CC BY 4.0 (<http://creativecommons.org/licenses/by/4.0/>). Please be aware that this license allows unrestricted use and distribution, even commercially, by third parties. Please reply and provide explicit written permission to publish XXX under a CC BY license and complete the attached form.”

Please upload the completed Content Permission Form or other proof of granted permissions as an "Other" file with your submission.

In the figure caption of the copyrighted figure, please include the following text: “Reprinted from [ref] under a CC BY license, with permission from [name of publisher], original copyright [original copyright year].”

2. If you are unable to obtain permission from the original copyright holder to publish these figures under the CC BY 4.0 license or if the copyright holder’s requirements are incompatible with the CC BY 4.0 license, please either i) remove the figure or ii) supply a replacement figure that complies with the CC BY 4.0 license. Please check copyright information on all replacement figures and update the figure caption with source information. If applicable, please specify in the figure caption text when a figure is similar but not identical to the original image and is therefore for illustrative purposes only.

6. We note that Figures 2, 3, 4, and 5 in your submission contain map images which may be copyrighted. All PLOS content is published under the Creative Commons Attribution License (CC BY 4.0), which means that the manuscript, images, and Supporting Information files will be freely available online, and any third party is permitted to access, download, copy, distribute, and use these materials in any way, even commercially, with proper attribution. For these reasons, we cannot publish previously copyrighted maps or satellite images created using proprietary data, such as Google software (Google Maps, Street View, and Earth). For more information, see our copyright guidelines: <http://journals.plos.org/plosone/s/licenses-and-copyright>.

We require you to either (1) present written permission from the copyright holder to publish these figures specifically under the CC BY 4.0 license, or (2) remove the figures from your submission:

1. You may seek permission from the original copyright holder of Figures 2, 3, 4, and 5 to publish the content specifically under the CC BY 4.0 license.

We recommend that you contact the original copyright holder with the Content Permission Form (<http://journals.plos.org/plosone/s/file?id=7c09/content-permission-form.pdf>) and the following text:

“I request permission for the open-access journal PLOS ONE to publish XXX under the Creative Commons Attribution License (CCAL) CC BY 4.0 (<http://creativecommons.org/licenses/by/4.0/>). Please be aware that this license allows unrestricted use and distribution, even commercially, by third parties. Please reply and provide explicit written permission to publish XXX under a CC BY license and complete the attached form.”

Please upload the completed Content Permission Form or other proof of granted permissions as an "Other" file with your submission.

In the figure caption of the copyrighted figure, please include the following text: “Reprinted from [ref] under a CC BY license, with permission from [name of publisher], original copyright [original copyright year].”

2. If you are unable to obtain permission from the original copyright holder to publish these figures under the CC BY 4.0 license or if the copyright holder’s requirements are incompatible with the CC BY 4.0 license, please either i) remove the figure or ii) supply a replacement figure that complies with the CC BY 4.0 license. Please check copyright information on all replacement figures and update the figure caption with source information. If applicable, please specify in the figure caption text when a figure is similar but not identical to the original image and is therefore for illustrative purposes only.

The following resources for replacing copyrighted map figures may be helpful:

USGS National Map Viewer (public domain): <http://viewer.nationalmap.gov/viewer/>

The Gateway to Astronaut Photography of Earth (public domain): <http://eol.jsc.nasa.gov/sseop/clickmap/>

Maps at the CIA (public domain): <https://www.cia.gov/library/publications/the-world-factbook/index.html> and <https://www.cia.gov/library/publications/cia-maps-publications/index.html>

NASA Earth Observatory (public domain): <http://earthobservatory.nasa.gov/>

Landsat: <http://landsat.visibleearth.nasa.gov/>

USGS EROS (Earth Resources Observatory and Science (EROS) Center) (public domain): [http://eros.usgs.gov/#](http://eros.usgs.gov/)

Natural Earth (public domain): [http://www.naturalearthdata.com/](http://track.editorialmanager.com/CL0/http:%2F%2Fwww.naturalearthdata.com%2F/1/010f019f07327bde-d420bfdf-d6ef-449b-9734-24b129d77121-000000/EJVOxNl70aA6E4I6XOFY2fIgdeVhK00QX7hhn-XpoSs=258)

A: We clarify that the maps in Figures 2,3,4 and 5 were generated by the authors in ArcGIS Pro from geographic and elevation data obtained through public domain, DIVA-GIS. The maps are original figures generated for this study and do not reproduce or adapt previously published maps or proprietary map imagery such as Google Maps, Google Earth, or Street View. The elevation data were derived from the Shuttle Radar Topography Mission (SRTM; Farr *et al.*, 2007). Major river layers were derived from public domain, Natural Earth 1:110m physical vector data (Natural Earth, 2009), whereas the Huai River was derived from the China 1:250,000 National Fundamental Geographic Database (National Geomatics Center of China, 2016).

For the frog photographs used in Figures 2–5, we further clarify that all photographs are original photographs taken by the authors and do not reproduce any third-party copyrighted material. Particularly, we have revised Figure 2 by replacing the previous frog images with photographs of specimens from our in-house collection. As clarification, photograph of *F. multistriata* was taken by the first author, S.N.O., whereas the remaining frog photographs were taken by the corresponding author, A.B. The authors agree to the publication of these photographs as part of the article under the CC BY 4.0 license.

We have also revised the captions of Figures 2–5 to explicitly identify the sources of the underlying geographic data and the copyright ownership of the photographs.

References

Farr TG, Rosen PA, Caro E, Crippen R, Duren R, Hensley S, *et al.* (2007). The shuttle radar topography mission. *Rev Geophys*: RG2004.

National Geomatics Center of China (2016). 1:250,000 National Fundamental Geographic Information Database of China. *Beijing, China Natl Geomatics Cent China*.

Natural Earth (2009). Rivers, Lake Centerlines. *Nat Earth*.

7. Please include captions for your Supporting Information files at the end of your manuscript, and update any in-text citations to match accordingly. Please see our Supporting Information guidelines for more information: [http://journals.plos.org/plosone/s/supporting-information](http://track.editorialmanager.com/CL0/http:%2F%2Fjournals.plos.org%2Fplosone%2Fs%2Fsupporting-information/1/010f019f07327bde-d420bfdf-d6ef-449b-9734-24b129d77121-000000/7aWgXai0kQF25n_DpQ-xWnFOikkOGF_p6jqRHSF2YzA=258).

A: We have revised the Supporting Information presentation accordingly. All supplementary methods, results, discussion, figures, and tables are compiled into a single Supporting Information file (S1 File), containing S1–S3 Figs and S1–S18 Tables. We have added captions for all Supporting Information figures and tables at the end of the main manuscript and updated all corresponding in-text citations to follow the PLOS ONE Supporting Information format.

8. If the reviewer comments include a recommendation to cite specific previously published works, please review and evaluate these publications to determine whether they are relevant and should be cited. There is no requirement to cite these works unless the editor has indicated otherwise.

A: We have reviewed the references recommended by the reviewers, evaluated their relevance to the present study, and added the relevant references to the revised manuscript accordingly.

[Note: HTML markup is below. Please do not edit.]

Reviewers' comments:

Reviewer's Responses to Questions

**Comments to the Author**

1. Is the manuscript technically sound, and do the data support the conclusions?

The manuscript must describe a technically sound piece of scientific research with data that supports the conclusions. Experiments must have been conducted rigorously, with appropriate controls, replication, and sample sizes. The conclusions must be drawn appropriately based on the data presented.

Reviewer #1: Yes

Reviewer #2: Partly

2. Has the statistical analysis been performed appropriately and rigorously?

Reviewer #1: Yes

Reviewer #2: Yes

3. Have the authors made all data underlying the findings in their manuscript fully available?

The [PLOS Data policy](http://track.editorialmanager.com/CL0/http:%2F%2Fwww.plosone.org%2Fstatic%2Fpolicies.action%23sharing/1/010f019f07327bde-d420bfdf-d6ef-449b-9734-24b129d77121-000000/zm2Zy7nLYPubM0DV619Z3Vb0LpywNnpNc_xJUjUrArs=258) requires authors to make all data underlying the findings described in their manuscript fully available without restriction, with rare exception (please refer to the Data Availability Statement in the manuscript PDF file). The data should be provided as part of the manuscript or its supporting information, or deposited to a public repository. For example, in addition to summary statistics, the data points behind means, medians and variance measures should be available. If there are restrictions on publicly sharing data—e.g. participant privacy or use of data from a third party—those must be specified.

Reviewer #1: Yes

Reviewer #2: Yes

4. Is the manuscript presented in an intelligible fashion and written in standard English?

PLOS ONE does not copyedit accepted manuscripts, so the language in submitted articles must be clear, correct, and unambiguous. Any typographical or grammatical errors should be corrected at revision, so please note any specific errors here.

Reviewer #1: Yes

Reviewer #2: Yes

5. Review Comments to the Author

Please use the space provided to explain your answers to the questions above. You may also include additional comments for the author, including concerns about dual publication, research ethics, or publication ethics. (Please upload your review as an attachment if it exceeds 20,000 characters)

Reviewer #1:

A: We appreciate Reviewer 1’s positive assessment of the technical soundness, statistical analyses, data availability, and overall presentation of the study, as well as the specific comments provided to further improve the manuscript. We have addressed each comment point by point below. All corresponding revisions are indicated by line numbers referring to the tracked-changes version of the revised manuscript.

Line 37 “Hoplobatracus” – Italics

A: We have italicized *Hoplobatrachus* (Line 38).

72 Reference 16 – Add recent data

A: We have updated Reference 16 using the most recent information available from the Amphibian Species of the World database and updated the access date accordingly. We also corrected the number of currently recognized Fejervarya species from 14 to 15, including the recently described *F. zhoushanensis*.

89 “Dufresnes et al” – Change to reference number

A: The reference has been updated to the numbering form. (Line 95)

99 “Dufresnes et al., 2019; Magalhães et al., 2024” – Change to reference number

A: The references have been updated to the numbering form. (Line 105).

151 Change as “Using the modified protocol of Chellex-100 Method [38]”

A: Corrected as suggested. The reference Dong et al. 2020 is updated to numbering form. (Line 167).

181 “RLC; Ogilvie et al., 2017” – change as reference number [39]

A: The reference Ogilvie et al., 2017 is updated to numbering form. (Line 198).

229 “Dicroglossidae in East Asia, based on published estimates [46]”

A: We have changed the citation from “Chen et al. (2017)” to the corresponding numbered citation. (Line 277).

265 ‘Xu et al. (2025)” – change to reference number [51]

A: In the text we have rephrased the sentence and cite Xu et al. (2025) in numbering form: *“we used a validated occurrence dataset integrating our field survey data with vetted GBIF records and remote-sensing variables [50]”* (Lines 304-305).

280 “We obtained 19 bioclimatic variables” – change to reference number [55]

A: We have changed the citation of Fick and Hijmans (2017) to the corresponding numbered reference format for consistency with the journal style. (Line 321).

289 Full stop not provided after “split [59]”

A: Corrected as suggested.

289 “Allouche et al. 2006” – Reference missing

A: The reference has been added. (Line 329).

307 Change as ‘Following previous observations [64]’

A: We have changed the text accordingly (Lines 347-348).

330 “Fejervarya” – Italics

A: We have corrected it as suggested.

350 “multistriata” – Italics

A: We have corrected it as suggested.

315 “Komac et al. 2016” – change to reference number [71]

A: We have changed Komac et al. 2016 to numbering format. (Line 457).

483 “Limnocharis” – italics

A: We have corrected it as suggested.

518 “Zhong et al., (2008)” – change to reference number [74]

*A: In the text we have rephrased the sentence as: “Contrary to the previously reported dominance of F. multistriata in the East Asian mainland [75]” (Lines 563-564).*

564 “Cai et al. 2018” – change to reference number [78]; 564 “Auliya et al. 2023” - Reference missing

A: We have reformatted the two references to their corresponding numbered citation format (Line 611).

568 “Christy et al. 2007” – Change to reference number [79]

A: We have reformatted the reference to the numbered citation format (Line 615).

583 Change highlighted to [51]

A: Corrected as suggested.

696 Change highlighted to lowercase

A: Corrected as suggested.

744 Add recent data

A: The Frost’s database reference has been updated to the recent data.

746 “Hoplobatracus rugulosus” – Italics

A: We have corrected it as suggested.

760 “Rana cancrivora” – Italics

A: We have corrected the reference as suggested.

778,779 Change highlighted to lowercase

A: The text is corrected as suggested.

950 “Fejervarya limnocharis” – Italics

A: We have corrected it as suggested.

Reviewer #2: This study reevaluates species boundaries and cryptic phylogenetic diversity in three genera of Dicroglossidae distributed across East and Southeast Asia. By integrating mitochondrial DNA, nuclear DNA, genome-derived SNPs, morphology, bioacoustics, and ecological niche modeling, the authors investigate patterns of lineage diversification, particularly in mainland China near the boundary between the Palearctic and Indo-Malayan realms.

This study demonstrates the taxonomic complexity of Asian Dicroglossidae and provides a valuable framework for integrating multilocus, genomic, ecological, and acoustic data in species delimitation analyses. In particular, the identification of previously unrecognized lineages within Fejervarya is an important contribution. However, several conclusions extend beyond what is directly supported by the available evidence, and a more cautious interpretation of species boundaries and taxonomic status is warranted.
For this reason, I consider this paper to be a major revision.

A: We appreciate the reviewer’s assessment. In response to the concerns raised, we have revised the manuscript to more clearly distinguish between the relative support for alternative species-delimitation models, the identification of evolutionary lineages, and formal taxonomic conclusions. We have also moderated interpretations of species boundaries and taxonomic status where appropriate. Specific changes and additional analyses are addressed point-by-point below, with line numbers provided throughout the responses referring to the tracked-changes version of the revised manuscript.

As major comments on this paper,
Although this study aims to reevaluate the taxonomy across a broad region of Asia, only 11 individuals were newly sampled, and most of the conclusions rely on publicly available databases (GenBank, SRA). There is insufficient independent field sampling to support broad-scale taxonomic revision or definitive conclusions regarding species boundaries.

A: We agree that 11 newly sampled individuals alone would be insufficient to support a broad-scale formal taxonomic revision. However, this was not the design of the present study. The core of our study is a hierarchical, scenario-based species-delimitation framework tested primarily using publicly available molecular datasets and complemented by targeted new data. The main multilocus dataset comprised up to 517 taxon entries, from which we developed and explicitly compared 18 alternative species-delimitation and assignment models. To avoid this misconception, we have revised the scope and strength of our conclusions to reflect the available evidence.

We highlight that the 11 newly sampled individuals represent targeted samples distributed at a key ecozones boundary (southeastern Palearctic and northern Indomalaya) rather than the sampling basis for our Dicroglossidae inference across Asia.

(1) We reframed objectives that previously implied definitive resolution of species boundaries to emphasise the evaluation of alternative species-delimitation hypotheses using available multilocus, genomic, morphological, and ecological evidence. The revised goals in the text now reads:

*“(1) to evaluate alternative lineage-assignment and species delimitation hypotheses within three Dicroglossid genera across the Eastern Indomalaya and Southeastern Palearctic using integrative species delimitation approaches, and genome-informed data (Lines 110-111).*

*(2) To further assess the lineage boundaries within the widespread and sympatric Fejervarya species complexes in East Asia (F. kawamurai, F. multistriata, and F. limnocharis) through comparative phylogeographic analysis, complemented by ecological niche modelling and bioacoustics evidence.*” (Lines 116 - 118).

(2) In Methods, we re-clarified the roles of public data and our field sampling. We stressed that publicly available molecular datasets provide the primary range-wide sampling, whereas our 11 newly collected individuals provide targeted, complementary sampling at the focal ecozone boundary.

“*To construct a geographically broad framework for species delimitation simulation, we assembled publicly available molecular datasets as the primary data source for a geographically broad, scenario-based species delimitation framework, complemented by targeted field sampling at a key ecozone boundary.* “ (Lines 127-130).

(3) We moderated statements that could imply stronger species-delimitation conclusions than directly supported by our analyses. For example, we replaced statements describing the “superiority” of Model A1 and the “robustness” of the proposed species tree with language describing relative support for Model A1 compared with the GenBank-based Model C1. We similarly refer to the recovered groups as evolutionary lineages where the available evidence does not justify formal species recognition.
(4) We also revised the ecological interpretation. We now describe differences in predicted habitat associations while explicitly acknowledging ecological overlap, rather than treating ENM results as direct evidence of taxonomic separation. The revised text reads:

*“Focusing on a sympatric lineage pair at the Yangtze-Huai ecozone boundary, ecological niche and acoustic analyses provided complementary evidence for differentiation between lineages despite geographic overlap (Fig. 4).* “ (Line 374).

Although deep genetic differentiation is evident within each Fejervarya complex, morphological support is limited and evidence for reproductive isolation is lacking. At present, these clades are more appropriately discussed as independent evolutionary lineages rather than distinct species.

A: We agree that in the absence of consistent morphological evidence or direct evidence of reproductive isolation, the recovered genetic differentiation should not be interpreted as definitive species-level separation.

We now refer to newly recovered subdivisions within the Fejervarya species complexes more cautiously as “independent evolutionary lineages” rather than distinct species. “Clade I” represents the type-locality lineage, while “Clade II” represents the divergent lineage within each complex.

In the text, we have tone down the wording to avoid implying definitive species-level resolution: *“to improve species-level resolution”* was revised to *“to refine lineage resolution”* (Lines 213-214).

The statement that ecological niche differences were *“supporting their taxonomic separation”* was removed, and the ENM results are now restricted to describing differences in predicted habitat associations and ecological overlap between lineages (Lines 452–456).

We have also moderated the wording that implying our results provided a definitive species delimitation. *“Fine-scale delimitation”* was revised to *“fine-scale assessment of lineage structure,”* and *“clarifies”* was revised to *“further characterises”* the overlapping ranges of the two Fejervarya lineages (Lines 584–587).

We have further moderated the taxonomic interpretation in the Discussion by revising *“clarify species boundaries”* to *“clarify lineage assignments”* in the context of conservation (Lines 666–667).

The SNP analysis integrates data derived from GBS, RNA-seq, and WGS. However, the study does not provide sufficient assessment regarding the effects of missing data rates and mapping biases arising from the use of different datatypes. Although Nanorana parkeri was used as the reference genome for SNP mapping, there is a potential for bias given the phylogenetic distance from the target genus. Detailed information on mapping success, missing-data rates, and potential reference bias should be provided.

A: We thank the reviewer for raising the potential effects of heterogeneous genomic data, missingness, and reference-genome bias. We primarily based species delimitation on the eight-locus multilocus dataset and subsequently incorporated the SRA-derived genomic loci with the multilocus and morphological data as complementary evidence in the total-evidence analysis of the best-supported A1 model. Thus, we did not use the SRA-derived genomic data independently to define species boundaries.

The public GBS, RNA-seq, and WGS datasets differed in sequencing strategy and did not consistently provide restriction-enzyme or library-preparation information. We therefore could not apply a uniform de novo RAD-style assembly or restrict the analysis to a common enzyme pair. Instead, we processed all SRA datasets against the same Nanorana parkeri reference and applied consistent filtering criteria to standardize sequence recovery across these heterogeneous public datasets. This strategy is compatible with the use of genomic-locus alignments in StarBEAST2, which accommodates sequence alignments derived from genomic loci such as RAD-seq loci and exons (Ogilvie et al., 2017), and has also been applied to transcriptome-derived loci in empirical phylogenomic analyses (McGuire et al., 2023).

We added this clarification to the Methods in the text as:

*“Given the public genomic datasets comprised GBS, RNA-seq and WGS/ WCS data generated using different sequencing strategies, restriction-enzyme and library-preparation information was not consistently available across datasets. Consequently, a uniform de novo assembly could not be applied. We therefore used a common reference-based framework to standardise sequence recovery across the heterogenous datasets. Because the genomic analysis was conducted within a single Asian Dicroglossidae phylogenetic framework, we aligned the reads to the Nanorana parkeri genome (GCF_000935625.1), which was used as a common family-level reference across the focal genera, and provides a chromosome-level assembly with high scaffold continuity. We aligned reads with reference genome using BWA-MEM v.0.7.17 [43]. For GBS datasets, SNP calling was performed using the Stacks v2.66 pipeline [44]. For Rna-seq and WGS/WCS datasets, we processed alignments using the GATK v.4.6 germline variant discovery pipeline [45]. Resulted VCF files were subsequently converted into NEXUS format for downstream species tree inference in BEAST pipeline (see loci information in S9 Table). This reference-based approach also generated genomic-locus alignments compatible with the StarBEAST2 framework, which accommodates sequence alignments derived from independent genomic loci such as RAD-seq and exons [41].”* (Lines 224-242).

Nevertheless, we agree that differences among sequencing strategies and the phylogenetic distance from the reference may influence initial locus recovery. We therefore conducted an additional assessment of missing or ambiguous characters (N and ?) directly from the genomic alignments retained in the A1 analysis. We identified 33 samples across three retained locus blocks: 17 RNA-seq samples with 70 loci, 10 GBS samples with 64 loci, and six WGS/WCS samples with 13 loci. All samples contained the full set of retained loci within their respective blocks. Sequence-level missingness was generally low, although some samples in the 64-locus block showed higher missingness (refer to S10 Table; and newly added S2 Fig.), both added to the S1 File. Supporting Information.

We added this clarification to the Methods in the text as:

*“Following genomic processing and filtering, we assessed data completeness directly from the genomic alignments retained for Model A1. For each sample, we recorded the number of retained loci and quantified the number and proportion of missing or ambiguous characters (N and ?) relative to the total alignment length. To evaluate variation among the heterogenous public datasets, we summarised these metrics at the sample and locus-recovery levels, and recorded the original sequencing data source (GBS, RNA-seq, or WGS /WCS). Although locus recovery differed among datasets (S10 Table), with 70 loci retained for RNA-seq samples, 64 for GBS samples, and 13 for WGS/WCS samples, median sequence-level missingness was 1.54% (S2 Fig.). All 33 genomic samples were retained because each sample contained data across the full set of loci retained for its respective dataset.”* (Lines 245-254).

Finally, we acknowledge that this assessment cannot eliminate potential reference bias during initial locus recovery. We therefore treat the SRA-derived loci as complementary genomic evidence rather than independent evidence defining species limits. Importantly, the multilocus analyses established support for A1 before we evaluated this model under the total-evidence framework.

We added this clarification to the Discussion as follows:

*“The genomic component also has limitations associated with the heterogeneous public datasets. The use of a common family-level reference may have influenced initial locus recovery because of the phylogenetic distance between Nanorana parkeri and the focal taxa. Accordingly, we interpret the SRA-derived genomic loci as complementary evidence rather than independent evidence defining species limits. Support for Model A1 was established from the multilocus analyses before its evaluation under the total-evidence framework.”* (Lines 705–711).

The manuscript frequently interprets Model A1 as representing the most likely species boundaries. However, the analyses only demonstrate that Model A1 fits the available data better than alternative scenarios. Additional evidence would be required before treating this model as a definitive representation of species limits.

A: We agree that Model A1 should be interpreted as the best-supported model among the alternative delimitation scenarios tested, rather than as a definitive representation of species limits. We recognise that some statements in the original manuscript regarding “species boundaries” and their resolution did not sufficiently maintain this distinction and could be interpreted as treating the lineages recovered under Model A1 as definitive species boundaries.

Accordingly, conclusions that could imply definitive species recognition have been toned-down to refer instead to the relative support among alternative delimitation models and the lineages recovered under them.

Importantly, we highlight that our final aim is to use the mismatches between recovered lineage assignments and existing geographic records with GenBank non updated assignment, to identify regions where revision or additional of sampling is particularly needed (Fig. 5). This provides a framework for prioritising future genomic sampling of key lineages and geographic areas before formal taxonomic reassessment.

Lastly, in the opening of the Discussion, we have clarified the role of our study as a model-based evaluation of competing lineage-assignment and delimitation hypotheses, rather than a formal taxonomic revision.

*“This multi-layered approach allowed us to evaluate the concordance between diverse lines of evidence and existing annotations, providing a broad baseline for future taxonomic revisions in these ecozones.”* (Lines 540-542).

at Line 682, we replaced *“the proposed taxonomic clarification”* with *“the lineage interpretation recovered under Model A1.”*

*“Accordingly, we interpret the SRA-derived genomic loci as complementary evidence rather than independent evidence defining species limits. Support for Model A1 was established from the multilocus analyses before its evaluation under the total-evidence framework.”* (Lines 707-710).

Finally, explicit taxonomic suggestions are retained separately in the Supporting Information (S1 File) for readers’ reference rather than presented as formal taxonomic conclusions in the main text.

Comments for ENM and bioacoustics analysis
Since the MANOVA results for vocalization analysis were not statistically significant, the resulting acoustic differences provide only limited evidence for behavioral differentiation or reproductive isolation.

A: We agree and have moderated the interpretation of the bioacoustic results accordingly. We retain the observed differences in call duration and interval, but no longer interpret these differences as direct evidence of reproductive isolation. Instead, we present them as acoustic differentiation that consistent with previous observations in the *F. limnocharis* complex.

In Discussion we have rephrased the wording as:

*“Bioacoustic analyses revealed that F. kawamurai calls are shorter in duration and longer in interval compared to F. multistriata (Fig. 4), showing acoustic differentiation similar to those reported between the F. limnocharis clades in Indomalaya [83].* (Lines 622-625).

The vocal differences between F. kawamurai and F. multistriata are interesting, but further sampling is needed to rule out the effects of regional and individual variations.

A: We agree that the current acoustic sampling cannot fully distinguish lineage-level differences from regional or individual variation. We have therefore revised the Discussion so that the observed call differences no longer confirm behavioural divergence, and we explicitly acknowledge the need for broader geographic and individual sampling.

*“However, although the overall acoustic difference was not significant, call duration and call interval differed between the two species (Fig. 4C), suggesting differences in specific acoustic variables, and a potential role of mating-call differences in reducing interspecific competition and facilitating coexistence [84]. Further geographic and individual sampling will be required to determine the consistency of these differences across populations.”* (Lines 650–657).

Although the MaxEnt analysis shows substantial overlap between the niches of the two lineages, the results seem to emphasize ecological divergence somewhat. The extent of this overlap should also be discussed in equal detail.

A: We agree that the original text placed disproportionate emphasis on differences in predicted habitat suitability. We have revised the Results and Discussion to give equal emphasis to the substantial overlap between the two lineages. Under the minPred threshold, the models predicted 1,870,774.17 km^2^ of suitable habitat for *F. kawamurai* and 2,091,013.23 km^2^ for *F. multistriata*, with 1,570,405.79 km^2^ of predicted overlap. We therefore no longer interpret these results as evidence of strong ecological divergence, but as differences in predicted habitat associations within broadly overlapping distributions (see Abstract: Lines 43–44; Results: Lines 478–480; Discussion: Lines 651–653).

A more detailed evaluation is needed regarding the impact of geographical bias in the GBIF data on the model results.

A: We had already implemented several measures to reduce potential bias in the occurrence data (S1 File. Supporting information, Lines 229–294). We curated questionable occurrences and applied 10-km spatial thinning to reduce spatial sampling bias, reducing 2,076 and 1,427 initial GBIF records to 286 and 597 occurrences for *F. kawamurai* and *F. multistriata*, respectively.

We also evaluated model performance using a 75:25 training-testing split, 10,000 background points, 10 replicate runs, and both AUC and TSS, and evaluated predictions under both minPred and the more restrictive MTSS threshold. Nevertheless, these procedures cannot fully eliminate geographical variation in survey effort and reporting intensity within GBIF. We have therefore expanded the Discussion to acknowledge residual sampling bias and now interpret fine-scale differences in predicted suitability cautiously, particularly in under-sampled regions.

Divergence-time estimation is not essential for the primary objective of species delimitation and taxonomic reassessment. Given the reliance on calibration by a secondary calibration point at basal position, temporal interpretations linking lineage divergence to specific geological or climatic events should be presented more cautiously.

A: We agree that divergence-time estimation is not essential for selecting among alternative species-delimitation models. We nevertheless retain the analysis because it provides complementary temporal context for interpreting the lineage structure (such as type-locality versus diverging clades) recovered under the best-supported model. Therefore, we have clarified that divergence times are not used as independent evidence for species boundaries in the Results.

*“The F. multistriata complex originated c. 6.29 Mya (Clade h; Fig. 3), with subsequent diversification c. 2.00–3.00 Mya (Fig. 2), temporally overlapping with Pleistocene climatic fluctuations in the Palearctic, although a direct association was not tested here.”* (Lines 445-447).

*“The Fejervarya assemblage emerged in the Early Miocene (c. 19.32 Mya; Fig. 3), followed by diversification across the biogeographic transition between the Indomalayan and Southern Palearctic regions.”* (Lines 424-425)

We acknowledge the uncertainty associated with secondary calibration and have therefore interpreted the divergence-time estimates cautiously in the Discussion. We describe allopatry, ecological associations, and anthropogenic influences as potential interpretations of the recovered patterns, rather than as directly demonstrated drivers.

*“Allopatric divergence, cryptic diversity and anthropogenic activities may contribute to the patterns of taxonomic and geographic structure in Dicroglossidae frogs in the Southeastern Palearctic and Eastern Indomalaya.”* (Lines 545-547)

*“Splitting and clustering patterns in the topology of the best-supported and refined Model A1 (Fig. 3) are consistent with patterns of both allopatry and cryptic sympatry.”* (Lines 553-555).

Finally, we added regional context for the divergence-time estimates (Lines 559–560), noting that the recovered divergence times, particularly within *Fejervarya*, are broadly consistent with the regional temporal patterns of diversification that recently being synthesised for East Asia anurans (Othman and Borzée, 2026).

Ref: Othman SN, Borzée A (2026). From Paleogene to Anthropocene: phylogeography, geographic patterns of traits, and chronology of evolutionary drivers in northeast Asian anurans. Heredity (Edinb) 135: 497–524.

Several interpretations in the Discussion, including references to species boundaries, reproductive isolation, and anthropogenic translocation, appear stronger than the evidence presented in the Results. The authors should more clearly distinguish between supported findings and speculative hypotheses.

A: Throughout the Discussion, we have moderated statements concerning species boundaries, reproductive isolation, ecological differentiation, divergence history, and anthropogenic translocation.

We clarify that the SRA-derived genomic loci are treated as complementary rather than independent evidence defining species limits, and that support for Model A1 was established from the multilocus analyses before its evaluation under the total-evidence framework (Lines 675–676, 704–707).

We treat the acoustic differences as limited complementary evidence and no longer interpret them as demonstrating reproductive isolation. Specifically, we clarify that the overall acoustic difference was not significant, while differences were detected in specific acoustic variables, and acknowledge that further geographic and individual sampling is required to determine their consistency across populations (Lines 650–657).

We have also revised statements concerning anthropogenic translocation to avoid implying a direct causal relationship where this is not supported by the present data. For example, in the Discussion we stated: *“Unlike the other two Dicroglossidae genera, the genetic structure of East Asian Hoplobatrachus clades may have been influenced by anthropogenic factors.”* (Lines 583–584).

6. PLOS authors have the option to publish the peer review history of their article ([what does this mean?](https://track.editorialmanager.com/CL0/https:%2F%2Fjournals.plos.org%2Fplosone%2Fs%2Feditorial-and-peer-review-process%23loc-peer-review-history/1/010f019f07327bde-d420bfdf-d6ef-449b-9734-24b129d77121-000000/xt_Yv4VWrIv3hK02rOtNOO9XyEC4cpRENKCLJv9aNzA=258)). If published, this will include your full peer review and any attached files.

If you choose “no”, your identity will remain anonymous but your review may still be made public.

**Do you want your identity to be public for this peer review?** For information about this choice, including consent withdrawal, please see our [Privacy Policy](https://track.editorialmanager.com/CL0/https:%2F%2Fwww.plos.org%2Fprivacy-policy/1/010f019f07327bde-d420bfdf-d6ef-449b-9734-24b129d77121-000000/PT53HHaxuo_thTrEeqg0k5XzSjMu9GwChBXtWHKitEA=258).

Reviewer #1: No

Reviewer #2: No

[NOTE: If reviewer comments were submitted as an attachment file, they will be attached to this email and accessible via the submission site. Please log into your account, locate the manuscript record, and check for the action link "View Attachments". If this link does not appear, there are no attachment files.]

To ensure your figures meet our technical requirements, please review our figure guidelines: <https://journals.plos.org/plosone/s/figures>

You may also use PLOS’s free figure tool, NAAS, to help you prepare publication quality figures: <https://journals.plos.org/plosone/s/figures#loc-tools-for-figure-preparation>.

NAAS will assess whether your figures meet our technical requirements by comparing each figure against our figure specifications.
