# Supplementary material for: Model-based multilocus, genomic, and phenotypic integration reveals species assignment inconsistencies and sampling blind spots across Asian ecozones in Dicroglossidae
Source: PLoS One. 2026 Oct 23;21(10):e0360243. doi: 10.1371/journal.pone.0360243 (PMC13693946; doi:10.1371/journal.pone.0360243)
Supplement: Peer review history

# Peer review: Decision Letter 0

**DOI:** 10.1371/journal.pone.0360243.r001

**Authors:** Martín O. Pereyra

26 Jun 2026

Dear Dr. Borzée,

Thank you for submitting your manuscript to PLOS ONE. After careful consideration, we feel that it has merit but does not fully meet PLOS ONE’s publication criteria as it currently stands. Therefore, we invite you to submit a revised version of the manuscript that addresses the points raised during the review process.

The manuscript was evaluated by two expert reviewers. While one reviewer provided only minor comments, the other reviewer has raised major concerns that need to be carefully considered and thoroughly discussed in the revised version. In summary, these concerns relate to data sampling, morphological and biological evidence considered, analytical issues, and interpretation and discussion of some results.

Please submit your revised manuscript by Aug 25 2026 11:59PM. If you will need more time than this to complete your revisions, please reply to this message or contact the journal office at plosone@plos.org. When you're ready to submit your revision, log on to https://www.editorialmanager.com/pone/ and select the 'Submissions Needing Revision' folder to locate your manuscript file.

- A letter that responds to each point raised by the academic editor and reviewer(s). You should upload this letter as a separate file labeled 'Response to Reviewers'.
- A marked-up copy of your manuscript that highlights changes made to the original version. You should upload this as a separate file labeled 'Revised Manuscript with Track Changes'.
- An unmarked version of your revised paper without tracked changes. You should upload this as a separate file labeled 'Manuscript'.

If you would like to make changes to your financial disclosure, please include your updated statement in your cover letter. Guidelines for resubmitting your figure files are available below the reviewer comments at the end of this letter.

If applicable, we recommend that you deposit your laboratory protocols in protocols.io to enhance the reproducibility of your results. Protocols.io assigns your protocol its own identifier (DOI) so that it can be cited independently in the future. For instructions see: https://journals.plos.org/plosone/s/submission-guidelines#loc-laboratory-protocols. Additionally, PLOS ONE offers an option for publishing peer-reviewed Lab Protocol articles, which describe protocols hosted on protocols.io. Read more information on sharing protocols at https://plos.org/protocols?utm_medium=editorial-email&utm_source=authorletters&utm_campaign=protocols.

As the corresponding author, your ORCID iD is verified in the submission system and will appear in the published article. PLOS supports the use of ORCID, and we encourage all coauthors to register for an ORCID iD and use it as well. Please encourage your coauthors to verify their ORCID iD within the submission system before final acceptance, as unverified ORCID iDs will not appear in the published article. *Only*  the individual author can complete the verification step; PLOS staff *cannot*  verify ORCID iDs on behalf of authors.

We look forward to receiving your revised manuscript.

Kind regards,

Martín O. Pereyra

Academic Editor

PLOS One

**Journal Requirements:**

When submitting your revision, we need you to address these additional requirements.

1. Please ensure that your manuscript meets PLOS ONE's style requirements, including those for file naming. The PLOS ONE style templates can be found at

https://journals.plos.org/plosone/s/file?id=wjVg/PLOSOne_formatting_sample_main_body.pdf and

https://journals.plos.org/plosone/s/file?id=ba62/PLOSOne_formatting_sample_title_authors_affiliations.pdf

2. To comply with PLOS One submissions requirements, in your Methods section, please provide additional information regarding the experiments involving animals and ensure you have included details on (1) methods of sacrifice, (2) methods of anesthesia and/or analgesia, and (3) efforts to alleviate suffering.

3. We note that the grant information you provided in the ‘Funding Information’ and ‘Financial Disclosure’ sections do not match.

When you resubmit, please ensure that you provide the correct grant numbers for the awards you received for your study in the ‘Funding Information’ section.

4. Please note that funding information should not appear in any section or other areas of your manuscript. We will only publish funding information present in the Funding Statement section of the online submission form. Please remove any funding-related text from the manuscript.

5. We note that Figures 2, 3,and 4 in your submission contain copyrighted images. All PLOS content is published under the Creative Commons Attribution License (CC BY 4.0), which means that the manuscript, images, and Supporting Information files will be freely available online, and any third party is permitted to access, download, copy, distribute, and use these materials in any way, even commercially, with proper attribution. For more information, see our copyright guidelines: http://journals.plos.org/plosone/s/licenses-and-copyright.

We require you to either (1) present written permission from the copyright holder to publish these figures specifically under the CC BY 4.0 license, or (2) remove the figures from your submission:

1. You may seek permission from the original copyright holder of Figures 2, 3, and 4 to publish the content specifically under the CC BY 4.0 license.

We recommend that you contact the original copyright holder with the Content Permission Form (http://journals.plos.org/plosone/s/file?id=7c09/content-permission-form.pdf) and the following text:

“I request permission for the open-access journal PLOS ONE to publish XXX under the Creative Commons Attribution License (CCAL) CC BY 4.0 (http://creativecommons.org/licenses/by/4.0/). Please be aware that this license allows unrestricted use and distribution, even commercially, by third parties. Please reply and provide explicit written permission to publish XXX under a CC BY license and complete the attached form.”

Please upload the completed Content Permission Form or other proof of granted permissions as an "Other" file with your submission.

In the figure caption of the copyrighted figure, please include the following text: “Reprinted from [ref] under a CC BY license, with permission from [name of publisher], original copyright [original copyright year].”

2. If you are unable to obtain permission from the original copyright holder to publish these figures under the CC BY 4.0 license or if the copyright holder’s requirements are incompatible with the CC BY 4.0 license, please either i) remove the figure or ii) supply a replacement figure that complies with the CC BY 4.0 license. Please check copyright information on all replacement figures and update the figure caption with source information. If applicable, please specify in the figure caption text when a figure is similar but not identical to the original image and is therefore for illustrative purposes only.

6. We note that Figures 2, 3, 4, and 5 in your submission contain map images which may be copyrighted. All PLOS content is published under the Creative Commons Attribution License (CC BY 4.0), which means that the manuscript, images, and Supporting Information files will be freely available online, and any third party is permitted to access, download, copy, distribute, and use these materials in any way, even commercially, with proper attribution. For these reasons, we cannot publish previously copyrighted maps or satellite images created using proprietary data, such as Google software (Google Maps, Street View, and Earth). For more information, see our copyright guidelines: http://journals.plos.org/plosone/s/licenses-and-copyright.

We require you to either (1) present written permission from the copyright holder to publish these figures specifically under the CC BY 4.0 license, or (2) remove the figures from your submission:

1. You may seek permission from the original copyright holder of Figures 2, 3, 4, and 5 to publish the content specifically under the CC BY 4.0 license.

We recommend that you contact the original copyright holder with the Content Permission Form (http://journals.plos.org/plosone/s/file?id=7c09/content-permission-form.pdf) and the following text:

“I request permission for the open-access journal PLOS ONE to publish XXX under the Creative Commons Attribution License (CCAL) CC BY 4.0 (http://creativecommons.org/licenses/by/4.0/). Please be aware that this license allows unrestricted use and distribution, even commercially, by third parties. Please reply and provide explicit written permission to publish XXX under a CC BY license and complete the attached form.”

Please upload the completed Content Permission Form or other proof of granted permissions as an "Other" file with your submission.

In the figure caption of the copyrighted figure, please include the following text: “Reprinted from [ref] under a CC BY license, with permission from [name of publisher], original copyright [original copyright year].”

2. If you are unable to obtain permission from the original copyright holder to publish these figures under the CC BY 4.0 license or if the copyright holder’s requirements are incompatible with the CC BY 4.0 license, please either i) remove the figure or ii) supply a replacement figure that complies with the CC BY 4.0 license. Please check copyright information on all replacement figures and update the figure caption with source information. If applicable, please specify in the figure caption text when a figure is similar but not identical to the original image and is therefore for illustrative purposes only.

The following resources for replacing copyrighted map figures may be helpful:

USGS National Map Viewer (public domain): http://viewer.nationalmap.gov/viewer/

The Gateway to Astronaut Photography of Earth (public domain): http://eol.jsc.nasa.gov/sseop/clickmap/

Maps at the CIA (public domain): https://www.cia.gov/library/publications/the-world-factbook/index.html and https://www.cia.gov/library/publications/cia-maps-publications/index.html

NASA Earth Observatory (public domain): http://earthobservatory.nasa.gov/

Landsat: http://landsat.visibleearth.nasa.gov/

USGS EROS (Earth Resources Observatory and Science (EROS) Center) (public domain): http://eros.usgs.gov/#

Natural Earth (public domain): http://www.naturalearthdata.com/

7. Please include captions for your Supporting Information files at the end of your manuscript, and update any in-text citations to match accordingly. Please see our Supporting Information guidelines for more information: http://journals.plos.org/plosone/s/supporting-information.

8. If the reviewer comments include a recommendation to cite specific previously published works, please review and evaluate these publications to determine whether they are relevant and should be cited. There is no requirement to cite these works unless the editor has indicated otherwise.

[Note: HTML markup is below. Please do not edit.]

Reviewers' comments:

Reviewer's Responses to Questions

**Comments to the Author**

1. Is the manuscript technically sound, and do the data support the conclusions?

Reviewer #1: Yes

Reviewer #2: Partly

**********

2. Has the statistical analysis been performed appropriately and rigorously?

Reviewer #1: Yes

Reviewer #2: Yes

**********

3. Have the authors made all data underlying the findings in their manuscript fully available?

The PLOS Data policy

Reviewer #1: Yes

Reviewer #2: Yes

**********

4. Is the manuscript presented in an intelligible fashion and written in standard English?

Reviewer #1: Yes

Reviewer #2: Yes

**********

Reviewer #1: Line 37 “Hoplobatracus” - Italics

72 Reference 16 – Add recent data

89 “Dufresnes et al” – Change to reference number

99 “Dufresnes et al., 2019; Magalhães et al., 2024” – Change to reference number

151 Change as “Using the modified protocol of Chellex-100 Method [38]”

181 “RLC; Ogilvie et al., 2017” – change as reference number [39]

229 “Dicroglossidae in East Asia, based on published estimates [46]”

265 ‘Xu et al. (2025)” – change to reference number [51]

280 “We obtained 19 bioclimatic variables” – change to reference number [55]

289 Full stop not provided after “split [59]”

289 “Allouche et al. 2006” – Reference missing

307 Change as ‘Following previous observations [64]’

330 “Fejervarya” - Italics

350 “multistriata” - Italics

315 “Komac et al. 2016” – change to reference number [71]

483 “Limnocharis” - italics

518 “Zhong et al., (2008)” – change to reference number [74]

564 “Cai et al. 2018” – change to reference number [78]

564 “Auliya et al. 2023” - Reference missing

568 “Christy et al. 2007” – Change to reference number [79]

583 Change highlighted to [51]

696 Change highlighted to lowercase

744 Add recent data

746 “Hoplobatracus rugulosus” - Italics

760 “Rana cancrivora” - Italics

778,779 Change highlighted to lowercase

950 “Fejervarya limnocharis” - Italics

Reviewer #2: This study reevaluates species boundaries and cryptic phylogenetic diversity in three genera of Dicroglossidae distributed across East and Southeast Asia. By integrating mitochondrial DNA, nuclear DNA, genome-derived SNPs, morphology, bioacoustics, and ecological niche modeling, the authors investigate patterns of lineage diversification, particularly in mainland China near the boundary between the Palearctic and Indo-Malayan realms.

This study demonstrates the taxonomic complexity of Asian Dicroglossidae and provides a valuable framework for integrating multilocus, genomic, ecological, and acoustic data in species delimitation analyses. In particular, the identification of previously unrecognized lineages within Fejervarya is an important contribution. However, several conclusions extend beyond what is directly supported by the available evidence, and a more cautious interpretation of species boundaries and taxonomic status is warranted.

For this reason, I consider this paper to be a major revision.

As major comments on this paper,

Although this study aims to reevaluate the taxonomy across a broad region of Asia, only 11 individuals were newly sampled, and most of the conclusions rely on publicly available databases (GenBank, SRA). There is insufficient independent field sampling to support broad-scale taxonomic revision or definitive conclusions regarding species boundaries.

Although deep genetic differentiation is evident within each Fejervarya complex, morphological support is limited and evidence for reproductive isolation is lacking. At present, these clades are more appropriately discussed as independent evolutionary lineages rather than distinct species.

The SNP analysis integrates data derived from GBS, RNA-seq, and WGS. However, the study does not provide sufficient assessment regarding the effects of missing data rates and mapping biases arising from the use of different datatypes.

Although Nanorana parkeri was used as the reference genome for SNP mapping, there is a potential for bias given the phylogenetic distance from the target genus. Detailed information on mapping success, missing-data rates, and potential reference bias should be provided.

The manuscript frequently interprets Model A1 as representing the most likely species boundaries. However, the analyses only demonstrate that Model A1 fits the available data better than alternative scenarios. Additional evidence would be required before treating this model as a definitive representation of species limits.

Comments for ENM and bioacoustics analysis

Since the MANOVA results for vocalization analysis were not statistically significant, the resulting acoustic differences provide only limited evidence for behavioral differentiation or reproductive isolation.

The vocal differences between F. kawamurai and F. multistriata are interesting, but further sampling is needed to rule out the effects of regional and individual variations.

Although the MaxEnt analysis shows substantial overlap between the niches of the two lineages, the results seem to emphasize ecological divergence somewhat. The extent of this overlap should also be discussed in equal detail.

A more detailed evaluation is needed regarding the impact of geographical bias in the GBIF data on the model results.

Divergence-time estimation is not essential for the primary objective of species delimitation and taxonomic reassessment. Given the reliance on calibration by a secondary calibration point at basal position, temporal interpretations linking lineage divergence to specific geological or climatic events should be presented more cautiously.

Several interpretations in the Discussion, including references to species boundaries, reproductive isolation, and anthropogenic translocation, appear stronger than the evidence presented in the Results. The authors should more clearly distinguish between supported findings and speculative hypotheses.

**********

what does this mean?). If published, this will include your full peer review and any attached files.

If you choose “no”, your identity will remain anonymous but your review may still be made public.

**Do you want your identity to be public for this peer review?**  For information about this choice, including consent withdrawal, please see our Privacy Policy

Reviewer #1: No

Reviewer #2: No

**********

[NOTE: If reviewer comments were submitted as an attachment file, they will be attached to this email and accessible via the submission site. Please log into your account, locate the manuscript record, and check for the action link "View Attachments". If this link does not appear, there are no attachment files.]

To ensure your figures meet our technical requirements, please review our figure guidelines: https://journals.plos.org/plosone/s/figures

You may also use PLOS’s free figure tool, NAAS, to help you prepare publication quality figures: https://journals.plos.org/plosone/s/figures#loc-tools-for-figure-preparation.

NAAS will assess whether your figures meet our technical requirements by comparing each figure against our figure specifications.

# Author comment: Author response to Decision Letter 1

**DOI:** 10.1371/journal.pone.0360243.r002

3 Sep 2026

Thank you for considering our submission for publication, we have addressed all comments in the answer letter we have uploaded

Attachment Submitted filename: Response to Reviewers.docx

# Peer review: Decision Letter 1

**DOI:** 10.1371/journal.pone.0360243.r003

**Authors:** Martín O. Pereyra

25 Sep 2026

Model-based multilocus, genomic, and phenotypic integration reveals species assignment inconsistencies and sampling blind spots across Asian ecozones in Dicroglossidae

PONE-D-26-21504R1

Dear Dr. Borzée,

We’re pleased to inform you that your manuscript has been judged scientifically suitable for publication and will be formally accepted for publication once it meets all outstanding technical requirements.

Within one week, you’ll receive an e-mail detailing the required amendments. When these have been addressed, you’ll receive a formal acceptance letter and your manuscript will be scheduled for publication.

An invoice will be generated when your article is formally accepted. Please note, if your institution has a publishing partnership with PLOS and your article meets the relevant criteria, all or part of your publication costs will be covered. Please make sure your user information is up-to-date by logging into Editorial Manager at Editorial Manager® and clicking the ‘Update My Information' link at the top of the page. For questions related to billing, please contact billing support.

If your institution or institutions have a press office, please notify them about your upcoming paper to help maximize its impact. If they’ll be preparing press materials, please inform our press team as soon as possible -- no later than 48 hours after receiving the formal acceptance. Your manuscript will remain under strict press embargo until 2 pm Eastern Time on the date of publication. For more information, please contact onepress@plos.org.

Kind regards,

Martín O. Pereyra

Academic Editor

PLOS One

Additional Editor Comments (optional):

Reviewers' comments:

Reviewer's Responses to Questions

**Comments to the Author**

Reviewer #2: All comments have been addressed

**********

2. Is the manuscript technically sound, and do the data support the conclusions?

Reviewer #2: Yes

**********

3. Has the statistical analysis been performed appropriately and rigorously?

Reviewer #2: Yes

**********

4. Have the authors made all data underlying the findings in their manuscript fully available?

The PLOS Data policy

Reviewer #2: Yes

**********

5. Is the manuscript presented in an intelligible fashion and written in standard English?

Reviewer #2: Yes

**********

Reviewer #2: I have reviewed the authors' responses to the comments from the previous round of peer review, as well as the revised manuscript.

I believe the authors have addressed my comments thoroughly and appropriately in their revisions. They also politely pointed out where I had misunderstood certain points and made revisions to clarify those areas.

Most of the concerns I previously held regarding the manuscript have been resolved, and I consider it to be of a quality suitable for publication. I recommend accepting the manuscript.

**********

what does this mean?). If published, this will include your full peer review and any attached files.

If you choose “no”, your identity will remain anonymous but your review may still be made public.

**Do you want your identity to be public for this peer review?**  For information about this choice, including consent withdrawal, please see our Privacy Policy

Reviewer #2: No

**********

# Editor report: Acceptance letter

**DOI:** 10.1371/journal.pone.0360243.r004

**Authors:** Martín O. Pereyra

PONE-D-26-21504R1

PLOS One

Dear Dr. Borzée,

I'm pleased to inform you that your manuscript has been deemed suitable for publication in PLOS One. Congratulations! Your manuscript is now being handed over to our production team.

At this stage, our production department will prepare your paper for publication. This includes ensuring the following:

* All references, tables, and figures are properly cited

* All relevant supporting information is included in the manuscript submission,

* There are no issues that prevent the paper from being properly typeset

You will receive further instructions from the production team, including instructions on how to review your proof when it is ready. Please keep in mind that we are working through a large volume of accepted articles, so please give us a few days to review your paper and let you know the next and final steps.

Lastly, if your institution or institutions have a press office, please let them know about your upcoming paper now to help maximize its impact. If they'll be preparing press materials, please inform our press team within the next 48 hours. Your manuscript will remain under strict press embargo until 2 pm Eastern Time on the date of publication. For more information, please contact onepress@plos.org.

You will receive an invoice from PLOS for your publication fee after your manuscript has reached the completed accept phase. If you receive an email requesting payment before acceptance or for any other service, this may be a phishing scheme. Learn how to identify phishing emails and protect your accounts at https://explore.plos.org/phishing.

If we can help with anything else, please email us at customercare@plos.org.

Thank you for submitting your work to PLOS One and supporting open access.

Kind regards,

PLOS One Editorial Office Staff

on behalf of

Dr. Martín O. Pereyra

Academic Editor

PLOS One
